# Evolution of *Oryza* chloroplast genomes promoted adaptation to diverse ecological habitats

**DOI:** 10.1038/s42003-019-0531-2

**Published:** 2019-07-26

**Authors:** Li-Zhi Gao, Yun-Long Liu, Dan Zhang, Wei Li, Ju Gao, Yuan Liu, Kui Li, Chao Shi, Yuan Zhao, You-Jie Zhao, Jun-Ying Jiao, Shu-Yan Mao, Cheng-Wen Gao, Evan E. Eichler

**Affiliations:** 1 Plant Germplasm and Genomics Research Center, Germplasm Bank of Wild Species in Southwest China, Kunming Institute of Botany, Chinese Academy of Sciences, 650204 Kunming, China; 2 Institution of Genomics and Bioinformatics, South China Agricultural University, 510642 Guangzhou, China; 3 The Ministry of Education Key Laboratory for Agricultural Biodiversity and Pest Management, Faculty of Plant Protection, Yunnan Agricultural University, 650204 Kunming, China; 4 Southwest China Forestry University, 650224 Kunming, China; 5 Howard Hughes Medical Institute, University of Washington, Seattle, WA 98195 USA

**Keywords:** Computational biology and bioinformatics, Ecology, Evolution, Genetics, Plant sciences

## Abstract

The course, tempo and mode of chloroplast genome evolution remain largely unknown, resulting in limited knowledge about how plant plastome gene and genome evolve during the process of recent plant speciation. Here, we report the complete plastomes of 22 closely related *Oryza* species in chronologically ordered stages and generate the first precise map of genomic structural variation, to our knowledge. The occurrence rapidity was estimated on average to be ~7 insertions and ~15 deletions per Myr. Relatively fewer deletions than insertions result in an increased repeat density that causes the observed growth of *Oryza* chloroplast genome sizes. Genome-wide scanning identified 14 positively selected genes that are relevant to photosynthesis system, eight of which were found independently in shade-tolerant or sun-loving rice species. *psaA* seemed positively selected in both shade-tolerant and sun-loving rice species. The results show that adaptive evolution of chloroplast genes makes rice species adapt to diverse ecological habitats related to sunlight preferences.

## Abstract (web summary)

Gao, Liu, Zhang, Li et al. report the complete plastid genome assemblies for 22 closely related species of rice, generating a map of genomic structural variation. They show that plastid gene evolution has helped rice evolve to diverse habitats with different levels of sunlight.

## Introduction

Chloroplasts were derived from endosymbiosis between independent living cyanobacteria and a non-photosynthetic host^1^. Each has its own genome that is usually nonrecombinant and uniparentally inherited ^2^. Chloroplast DNA (cpDNA) have increasingly been employed for resolving the deep phylogeny of plants because of their low rates of nucleotide substitutions and decelerated structural variation compared with nuclear genomic sequences^3–5^. Thus, it has been commonly recognized that the chloroplast genomes are prominently conserved in terms of genome size, genomic structure, and gene content among the majority of flowering plant species^4,6,7^. However, the course, tempo and mode in the evolution and genome architecture changes in the context of the recent speciation remain largely unknown, resulting in limited knowledge about the micro-evolution of chloroplast genes and genomes in flowering plants. The deep comparisons of closely related plant species in chronologically ordered different stages are able to dramatically improve the sensitivity of evolutionary inferences with genomic structural knowledge to thoroughly elucidate the mechanisms, rates, or directionality of chloroplast genome evolution. Such an effort could precisely generate more convincing results needed to examine patterns of genomic architectures during the speciation process than single chloroplast genome analysis can offer. With this regard, we know little about broad-scale patterns of evolutionary dynamics, discrepancy, and consequences that will provide in-depth insights into microstructural genome diversification and unravel mechanisms underlying the maintenance or disruption of microstructural organization. The availability of high-quality complete chloroplast genome sequences will offer an unprecedented opportunity to address these questions. It is required to initiate comparative analyses among multiple chloroplast genomes of very closely related plant species that span the speciation continuum under a well-resolved phylogenetic framework.

The genus *Oryza* is one of ideal groups to understand the diversity and evolution of chloroplast genes and genomes in flowering plants. It belongs to the grass family (Poaceae) and consists of more than 20 wild species and two cultivated species^8,9^. These rice species have been assigned as 10 genome types (AA, BB, CC, EE, FF, GG, BBCC, CCDD, HHJJ, and HHKK)^10–12^, representing an enormous gene pool for genetic improvement of modern rice cultivars. Molecular phylogenetic and phylogenomic studies in this genus have well-resolved evolutionary relationships and reconstructed the phylogeny of different rice genome types and species^10,12–18^. Besides cpDNA sequences that have been applied to determining phylogenetic relationships among *Oryza* species^12,19–23^, the completion of 10 *Oryza* plastomes (*O. australiensis*, *O. barthii*, *O. glaberrima*, *O. glumaepatula*, *O. longistaminata*, *O. meridionalis*, *O. nivara*, *O. officinalis*, *O. rufipogon*, and *O. sativa*)^24–30^, and for example, the fully sequenced rice chloroplast genomes^24,31^ have been extensively used to phylogenetic and population genetic studies in cultivated rice and wild relatives^32–34^. The genus *Oryza* particularly becomes an unparalleled system for studying comparative and evolutionary genomics in plants owning to available nuclear genomes of the two cultivated rice subspecies^35–37^and the other six *Oryza* species having been sequenced recently^18,38^. These species with well-defined phylogenetic framework and splitting times may undoubtedly serve as an important model that deserves endeavors for obtaining an in-depth view of chloroplast gene and genome evolution.

Next-generation sequencing techniques have revolutionized DNA sequencing via high-throughput capabilities and low costs^39^, making it now fairly convenient to obtain multiple high-quality plastome sequences. Here, we present the complete plastome sequences of 21 *Oryza* species and *Leersia japonica* from the tribe *Oryzeae* as outgroup. Our sampling representation allows us to infer the tempo and mode in *Oryza* plastome gene and genome evolution. To this end, we performed phylogenomic analysis based on whole-plastome sequences of almost all *Oryza* species to clarify evolutionary relationships among rice species towards obtaining overall insights into the rice chloroplast genome evolution. Understanding phylogenetic relationships of rice species will greatly enhance our efforts to search for favorable genes. Besides, maternally inherited nature of organelle genomes makes them potentially useful tools to identify diploid donor of the polyploidy species. Thus, another objective of this study was to elucidate the evolutionary relationships of the *Oryza* chloroplast genomes with emphasis on the origin and evolution of the allotetraploid rice species. In addition, all these cp genomes were pairwise compared to genome-wide identified structural variants, and such a comprehensive map of genomic variation across *Oryza* plastomes permits, to our knowledge, to gain the first vision about the tempos of the InDels that came into and/or removed from the chloroplast genomes during recent speciation. Specifically, we investigated the evolutionary dynamics of repeat sequences by means of assessing the pace of the occurrences and turnover as well as the magnitude of conservation since the generation along the timetabled *Oryza* phylogeny. We also evaluated how far-reaching structural variation has affected the evolution of chloroplast protein-coding genes; and finally, we scanned for chloroplast rice genes that evolve under Darwinian selection. These candidate genes of wild rice in particular are expected to play an important role in adaptations to their ecological niches in Asia, South America, Africa, and Australia, and become a valuable database of functional variation for the future rice improvement programs.

## Results

### Genome size, gene content and structure of *Oryza* plastomes

We sequenced and assembled plastomes of a total of 21 *Oryza* species and one closely related species, *Leersia japonica*, from the tribe *Oryzeae* (Supplementary Table 1). Illumina paired-end reads, after excluding non-chloroplast genome reads, were first mapped to the *O. sativa* ssp. *japonica* chloroplast genome (NC_001320) (AA- genome) as a reference, and these 21 chloroplast genomes (cp genomes) were generated with genome sizes varying from 134,074 bp (*L. japonica*) to 136,133 bp (*O. meyeriana*) (GG- genome; Supplementary Table 2). These 22 newly sequenced cp genomes in this study together with two additional *O. sativa* cp genomes (AA- genome; *O. sativa* ssp. *japonica*, NC_001320; *O. sativa* ssp. *indica*, NC_008155)^24,31^ exhibited well-maintained quadripartite structure; they consists of two inverted repeat regions (IRa and IRb) separated by large (LSC) and small (SSC) single-copy regions, typically resembling to the majority of flowering plant cp genomes^40^ (Supplementary Fig. 1). They displayed similar GC contents of ~39%. In comparisons with the relatively conserved sequences in the coding or IR regions, non-coding regions of the investigated cp genomes were apparently variable (Supplementary Fig. 2).

All *Oryza* cp genomes displayed a conserved gene content and commonly harbored an identical set of 111 annotated unique genes, including 77 unique protein-coding genes, 30 tRNA genes, and four rRNA genes (Supplementary Fig. 1**;** Supplementary Table 2), representing typical features of land plant cp genomes^7^^,^^41,42^. All four rRNA genes were located in the IRs, 21 tRNA genes were found in the single-copy region, and the others were positioned in the IRs. Of the 15 genes that contained introns, a total of the 14 genes (*trnK-UUU*, *rps16*, *atpF*, *trnL-UAA*, *trnV-UAC*, *petB*, *petD*, *rpl16*, *rpl2*, *ndhB*, *trnI-GAU*, *trnA-UGC*, *ndhA*, and *rps12*) had one intron with the exception of *ycf3* with two introns. *rps12* was trans-spliced with one of its exons located in the LSC regions (5′_end) and the other existed in the IR regions (3′_end), which were separated by a long intron. A pair of the *ndhH* genes was annotated, which stretched across the IR/SSC junctions with the 5′ end of *ndhH* located in SSC.

The occurrences of gene transfer and loss have been commonly observed in plant cp genomes^43–46^. *accD*, *ycf1*, *ycf2*, *rpl23*, *rpl22*, *infA*, *rps16*, and *ycf4* were reported partially or completely lost from the legume chloroplast genomes^47,48^, of these, some genes such as *infA* even have been lost for multiple times. To our knowledge, this is the first effort using such an extensively representative cp genomes to further understanding of patterns of gene loss among the closely related plants; in this case, we observed that *ycf1*, *ycf2*, and *ycf15* were lost from these sequenced *Oryza* cp genomes. *accD*, coding for a subunit of acetyl-CoA carboxylase, was absent from the *T. subterraneum* cp genome^48^. Sequence alignments of all annotated *accD* revealed a remarkably different gene structure and protein-coding sequences that were unlike those in other angiosperms (Supplementary Fig. 3), which may be suggestive of a novel gene structure. Raubeson et al.^49^ proposed that *ycf68* is a pseudogene in *Ranunculus*. In the genus *Oryza*, one SNV and two InDels were found within the *ycf68* gene at the sites of 49 bp, 140 bp, and 289 bp, respectively (Supplementary Fig. 4), resulting in frameshift mutations and premature termination codes in the functional Ycf68 protein (Supplementary Fig. 5).

In addition to an overall conservation of gene content, the sequenced *Oryza* cp genomes were structurally conserved and displayed an almost uniform order of the same set of genes with slight differences at boundary junctions (Supplementary Fig. 1). This is not exceptional as a slight IR expansion/contraction was also reported in other cp genomes^40,50,51^. In the junctions between IRs and SSCs, for example, *ndhH* genes marginally extended into the IR regions; the distances of *rps19* from the junction of LSC/IRb varied from 40 bp in *O. sativa* ssp. *indica* to 48 bp in *O. australiensis* (EE- genome); a trivial difference in junction positions was also observed among the 22 *Oryza* and *L. japonica* chloroplast genomes (Supplementary Fig. 6).

### Phylogenomic analysis based upon whole plastome sequences and *Oryza* chloroplast genome evolution

**Fig. 1:**
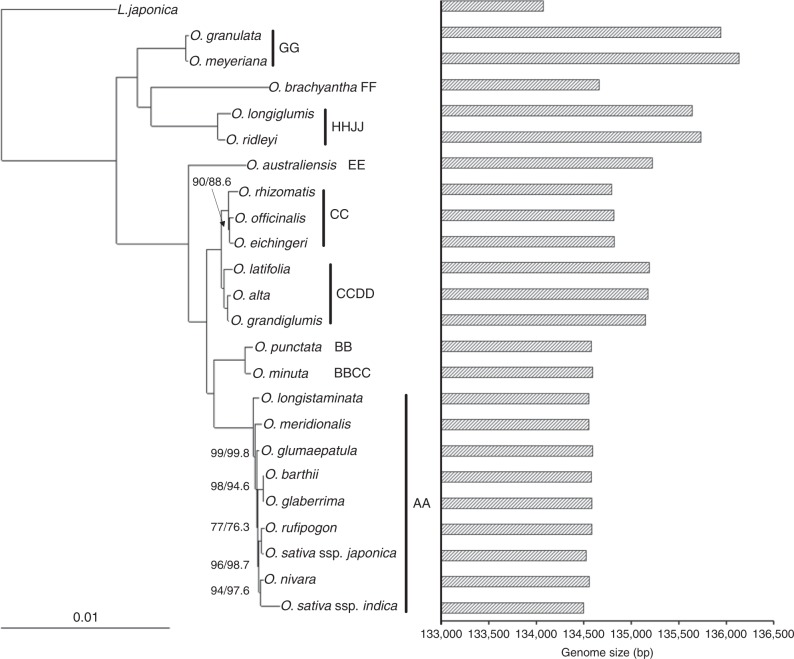
Phylogenomic relationships of the genus *Oryza* based on whole plastome sequences and the variation of rice chloroplast genome sizes. ML phylogeny of the *Oryza* species inferred from the whole-genome sequences of chloroplasts. Numbers near branches are bootstrap values of ML and MP, respectively. MP and BI inferences generated the same topology as ML tree (see Supplementary Figs. 7 and 8). The branches without numbers indicate 100% bootstrap supports. Chloroplast genome sizes are individually displayed and compared among the studied *Oryza* species

One of advantages to have sequenced chloroplast genomes of almost all *Oryza* species is to explore evolutionary relationships that allow a full picture of the rice plastome genome evolution. We concatenated the chloroplast whole-genome sequences into an alignment dataset comprising of 139,206 bp in length after removing all indels across the studied species. Of them, variable and phylogenetically informative sites were 5917 bp (4.25%) and 2766 bp (1.99%), respectively (Supplementary Table 3). Using *L. japonica* as outgroup, maximum likelihood (ML), maximum parsimony (MP), and Bayesian inference (BI) methods yielded the fully resolved phylogenetic trees that are nearly identical to each other with almost 100% bootstrap support values or 1.00 posterior probabilities (Fig. 1; Supplementary Figs. 7 and 8). In the resulting phylogenies, almost all *Oryza* species were grouped into clusters corresponding to different genome types, and the studied species formed genome type-based monophyletic clades. The genus *Oryza* was divided into the two main clades of which one branch comprised AA-, BB-, BBCC-, CC-, CCDD-, and EE- genome species, while the other included FF-, GG-, and HHJJ- species. The ML phylogenetic trees were further reconstructed using non-coding genomic sequences (Supplementary Fig. 9) and a total of 77 protein-coding genes (Supplementary Fig. 10), respectively. They were consistent to each other and largely congruent with the above-described topology based on the entire chloroplast genome sequences, strongly supporting the previous recognition of different genome types in both rice diploids and tetraploids by studying the meiotic pairing of hybrids between *Oryza* species^52,53^ and phylogenetic analysis^12^. The topological differences were merely observed when using protein-coding gene sequences, indicating that *O. brachyantha* (FF- genome) independently formed a monophyletic and failed to group with the GG- and HHJJ- genome species. This robust phylogeny is generally consistent with the phylogeny of *Oryza* species in earlier studies^12,18^, further providing solid grounds for performing evolutionary and comparative analyses. We further reconstructed phylogenetic trees using genomic indels (Supplementary Fig. 11) and indels presented in protein-coding genes (Supplementary Fig. 12). Compared to several topological differences (e.g., *O. nivara* and *O. glumapaetula*) of the phylogeny using genomic SVs (Supplementary Fig. 11), the SV phylogeny reconstructed using indels presented in protein-coding genes is quite consistent with the above-described SNV phylogeny topology with almost 100% bootstrap support (Supplementary Fig. 12).

Whole chloroplast genomes provide a wealth of information to determine the phylogenetic relationships of the eight AA- genome *Oryza* species using *L. japonica* as outgroup (Fig. 1; Supplementary Figs. 7–10). It displayed the same topology supported by high bootstrap values or posterior probabilities as using *O. punctata* (BB-genome) as outgroup. The results are in strong support of the resolution of evolutionary relationships of the eight AA- genome *Oryza* species as revealed by 53 nuclear singleton fragments and 16 intergenic genomic regions^17^. However, the most unexpected finding is that, in comparison to *O. meridionalis*, *O. longistaminata* appear to be distantly related to other AA- genome species, the basal lineage with high bootstrap values or posterior probabilities.

The genus *Oryza* constitutes more than one-third of rice species that are natural allotetraploids originated from two distinct diploid donors through hybridization events coupled with doubled chromosome numbers. Due to the maternally inherited nature of plant chloroplasts, obtaining the chloroplast phylogenomic tree that represents a maternal genealogy enables us to determine the maternal parents of allotetraploid species. To infer origins of allopolyploid rice species, the phylogenomic tree was reconstructed based on complete chloroplast genome sequences of the majority of *Oryza* species (Fig. 1; Supplementary Figs. 7–10). The three allotetraploid (CCDD) species, *O. alta, O. grandiglumis,* and *O. latifolia*, which formed a monophelytic clade, were closely related to the three diploid CC genome species, that is, *O. officinalis*, *O. rhizomatis*, and *O. eichingeri*, with 100% bootstrap support values or 1.00 posterior probabilities. Complete chloroplast genome-based results convincingly demonstrate the hypothesis that the CCDD genome originated from a single hybridization event^12,14,19,54–56^. Although it was well documented that the CC genome species (*O. officinalis*, *O. rhizomatis*, and *O. eichingeri*) served as the maternal parent of the CCDD genome tetraploids, their evolutionary relationships have long been in dispute. Wang et al.^14^ proposed that *O. eichingeri* from Africa is the closest to the CCDD genome tetraploids, while Bao and Ge^19^ suggested that *O. officinalis* and *O. rhizomatis* other than *O. eichingeri* were more closely related to the CCDD genome species. The chloroplast phylogenomic trees, however, indicates that *O. officinalis*, *O. rhizomatis*, and *O. eichingeri* almost similarly served as maternal parents of the CCDD genome tetraploids. Besides supporting a single origin of the CCDD genome species, the present study demonstrates that *O. alta* and *O. grandiglumis* are more closely related to each other than to *O. latifolia* with 100% bootstrap support values or 1.00 posterior probabilities, suggesting a significant divergence between the former two species and *O. latifolia* during the process of speciation and subsequent evolution. This result is seemingly in good agreement with the taxonomic treatment of Roschevicz^57^ that these American tetraploids as two separate species (*O. grandiglumis* and *O. latifolia*) (CCDD- genome) but multiple characters are needed by morphological, biogeographical, cytological, and genomic evidences. In addition, our results reveal that the allotetraploid *O. minuta* (BBCC- genome) presented a closer relationship with the diploid *O. puctata* (BB- genome), suggesting that the BB genome was the maternal donor during the formation of the BBCC genome species. Nevertheless, the BBCC genome species (*O. eichingeri*) revealed a different origin with maternal parent being the CC genome^12^. A robust reconstruction of origins of all allotetraploid rice species requires to complete chloroplast genome sequences of remaining tetraploids including *O. schlechteri* and *O. coarctata* and put extra efforts to ensure the diploid species with the DD, HH, JJ, or KK genomes that are either undiscovered or disappeared.

### A genome-wide map of genomic structural variation across *Oryza* plastomes

**Fig. 2:**
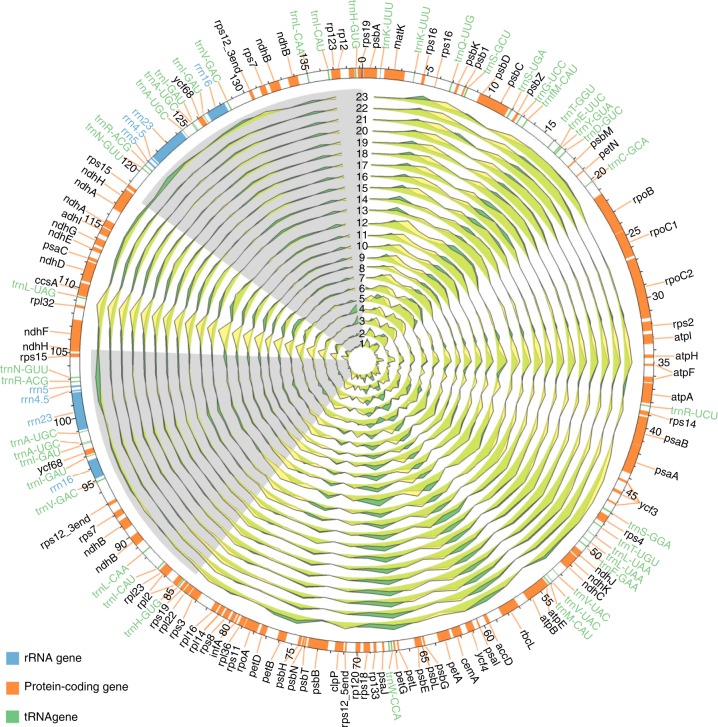
An overview of chloroplast genome variation across the *Oryza* plastomes. The InDel density of 2Kb was measured for the 23 *Oryza* chloroplast genomes using *L. japonica* as outgroup. In the quadripartite structure of these chloroplast genomes, the two IR regions (IR_A_ and IR_B_) are shown with gray background, while the large and small single-copy regions (LSC and SSC) are displayed with blank background. The deletions are filled with green ridges while the insertions are occupied with yellow ridges. The studied *Oryza* species are indicated as below: 1. *O. sativa* ssp. *japonica*; 2. *O. rufipogon*; 3. *O. sativa* ssp. *indica*; 4. *O. nivara*; 5. *O. glaberrima*; 6. *O. barthii*; 7. *O. glumaepatula*; 8. *O. meridionalis*; 9. *O. longistaminata*; 10. *O. punctate*; 11. *O. minuta*; 12. *O. officinalis*; 13. *O. eichingeri*; 14. *O. rhizomatis*; 15. *O. alta*; 16. *O. grandiglumis*; 17. *O. latifolia*; 18. *O. australiensis*; 19. *O. longiglumis*; 20. *O. ridleyi*; 21. *O. brachyantha*; 22. *O. granulata*; 23. *O. meyeriana*; and 24. *L. japonica*

Global sequence alignments of the 23 *Oryza* and *L. japonica* chloroplast genomes identified several variants, indicative of evolutionary processes in rice. With *L. japonica* as outgroup, we characterized genomic variation including SNVs, insertions, and deletions (InDels) in the *Oryza* chloroplast genomes, revealing that they varied from one species to another (Supplementary Tables 4 and 5). On the whole-genome level, the numbers of SNVs, deletions, and insertions per kb varied from one to another with average values of 16.22, 1.15, and 1.26, respectively. In terms of these three types of genomic variation, the IR regions presented the fewest numbers per kb with average values of 3.16, 0.43, and 0.41, respectively. The SSC regions exhibited the utmost numbers of SNVs and insertions per kb with average values of 26.43 and 1.84, respectively. Meanwhile, the number of deletions per kb was the largest in the LSC region with an average value of 1.34. These results together make evident that the IR regions were more conserved than single-copy regions, and that chloroplast genes were more conserved than the intergenic regions (Supplementary Fig. 2). Figure 2, Supplementary Figs. 2 and 13 provide an overview of genomic structural variation across *Oryza* plastomes, ascertaining trivial variation but overall conservation of genome architecture across these rice species.

**Fig. 3:**
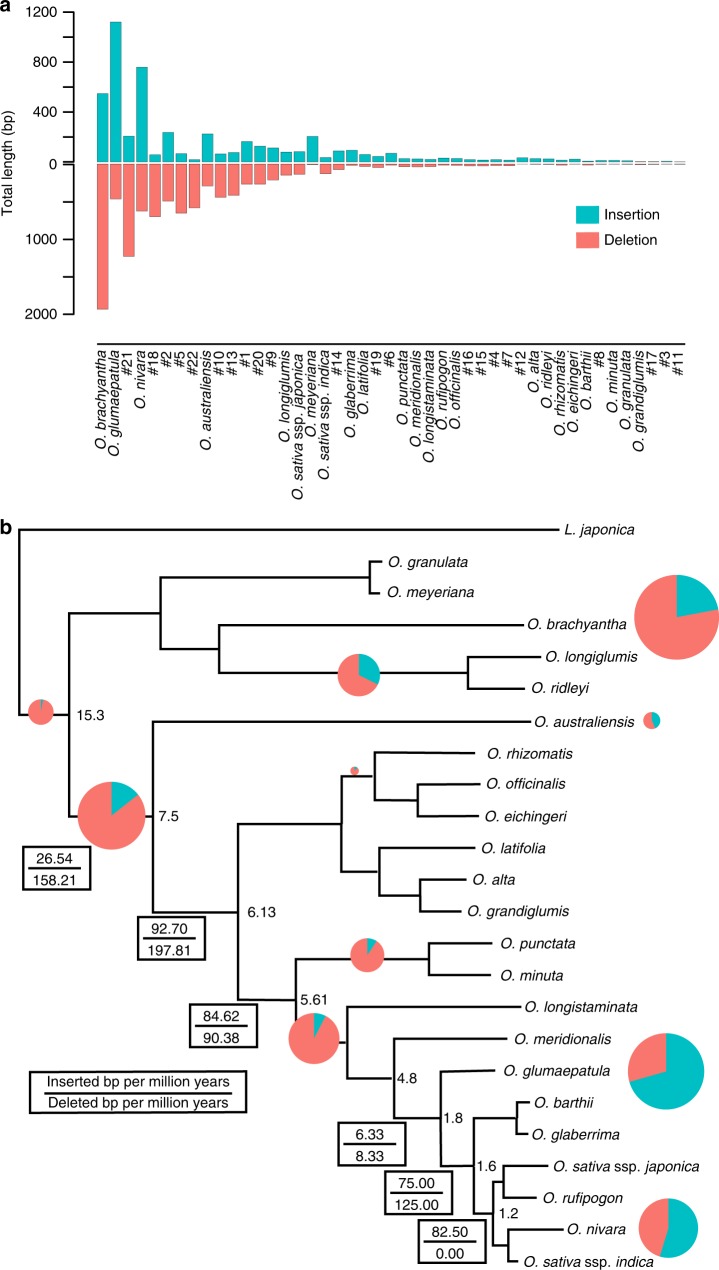
The occurrence rapidity of chloroplast genomic variation during speciation in the genus *Oryza*. **a** Accumulation rates of insertion and deletion lengths every million years along branches of the *Oryza* phylogeny. Pie of top 10 branches are scaled proportionally to InDel lengths. Divergence times (Myr) of major branches are shown by following estimates by Zou et al.^60^, Zhu et al.^17^ and Zhang et al.^18^; **b** Total lengths of insertion and deletion for different lineages of the genus *Oryza*. Detailed information of these lineages is shown in Supplementary Fig. 12C

To examine the occurrences of genomic structural variants along different lineages that account for the evolution of *Oryza* plastomes, we mapped all genomic structural variants onto the phylogenetic tree of the 23 *Oryza* chloroplast genomes using *L. japonica* as outgroup. These InDels were inferred by aligning 24 chloroplast genome sequences using a phylogeny-aware algorithm PRANK_+F_^58^, which has been proven to outperform traditional methods with regard to the accuracy in InDel-rich sequences. A total of 730 insertions were identified that varied from 1 to 234 bp in length. This summed to 4908 bp of insertions, compared to 994 deletions, ranging from 1 to 608 bp in length that affect 12,592 bp (Supplementary Tables 6 and 7; Supplementary Fig. 14A; Fig. 3a). The majority of detected InDels were small in size; ~99% of all InDels were <100 bp, whereas ~82% were <10 bp. The size distribution of structural variations is consistent with findings in plant nuclear genomes^18,59^ that longer variants were less abundant (Supplementary Fig. 14B).

To estimate the pace of these InDels in the *Oryza* plastomes, we dated the occurrences of structural variants by locating them at the timetabled phylogenetic nodes. The results showed that, during the past ~15.3 Mya in the middle Miocene^60^, the 24 lineages as well as their common ancestral branches, on average, had ~7 insertions and ~15 deletions per Myr, respectively (Supplementary Fig. 14A). We estimated insertion and removal rates of InDels based on the previously estimated times^17,18,60^ (Fig. 3b**;** Supplementary Fig. 14C). Our results further revealed that insertions and deletions remarkably varied across different lineages. Nevertheless, the estimated rates of either insertions or deletions were mostly constant along these lineages except for, for examples, *O. brachyantha* (FF- genome), *O. glumaepatula* (AA- genome), and *O. nivara* (AA- genome) with elevated rates and *O. barthi* (AA- genome), *O. alta* (CCDD- genome), and *O. officinalis* (CC- genome) with lowered rates (Fig. 3b**;** Supplementary Fig. 14C). The finding is consistent to the result that the numbers of genomic structural variants observed along different branches were largely proportional to branch lengths (Supplementary Fig. 14D). This is well consistent to the observation in the six AA- genome *Oryza* nuclear genomes that the occurrences of structural variation events are closely related to their phylogenetic positions and divergence times^18^.

The corresponding genomic positions of these identified InDels were mapped and located into the 23 *Oryza* plastomes, displaying that 92% of the predicted InDels were found in intronic (14%) and intergenic regions (78%) (Supplementary Table 8**;** Supplementary Fig. 15A). As expected, InDels were scarce in exons of protein-coding genes (4%) and RNA genes (4%). The uneven distribution of genomic structural variants throughout the plastomes suggests that they are likely to have negative impacts and thus easily eliminated by purifying selection.

Far-reaching structural variation may have affected not only genomic architectural heterogeneities but also the evolution of protein-coding genes in the *Oryza* chloroplast genomes. To examine whether this holds true, we characterized the length distribution of insertions and deletions within chloroplast protein-coding genes. Our results showed that, of the 71 InDels, there were merely 27 with lengths that are multiples of three (Supplementary Fig. 15B). This finding suggests that negative selection on frameshift InDels may not really affect the chloroplast protein-coding genes, which is sharply against the observation in the six nuclear genomes of *Oryza*^18^ and other flowering plants^38,59^. The prevalence of genomic structural variants within chloroplast genes were further characterized by mapping them into the exons of protein-coding and RNA genes of the *Oryza* chloroplast genomes (Supplementary Tables 9 and 10**;** Supplementary Fig. 15C**;** Supplementary Figs. 16–18), indicating that InDels serve as an important driver of rice chloroplast gene evolution.

Evolutionary patterns and tempo of cp genes and genomes have long been studied on a large scale, obtaining the knowledge of macro-evolution of cp genes and genomes in angiosperms^4,6,61^. With this regard, this is the first case study, to our knowledge, providing insight into the micro-evolution of cp genes and genomes in the course of recent speciation in flowering plants.

### Evolutionary dynamics of plastid repetitive DNA and the growth of *Oryza* plastomes

**Fig. 4:**
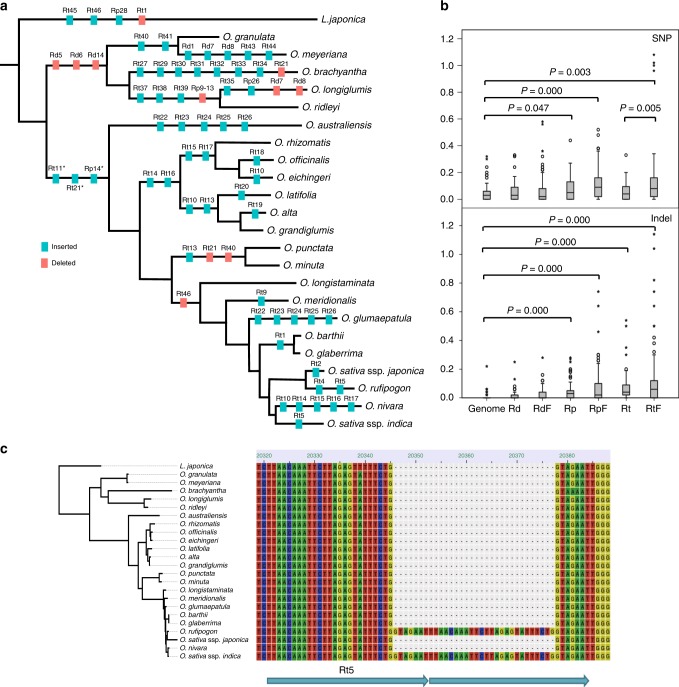
Evolutionary dynamics of repeat DNA sequences across *Oryza* plastomes. **a** Occurrences of repeat sequences that are mapped onto different lineages of the *Oryza* phylogeny using *L. japonica* as outgroup. Insertions and deletions of palindromic (Rp), dispersed (Rd) and tandem (Rt) repeats are colored in blue and red, respectively. Repeats in the common ancestor of *Oryza* and *L. japonica* were listed on the bottom left form. * indicates the shared repeats with SNV**;** ** shows the repeats located at the same genomic positions; **b** Repeats are enriched for indels and SNVs compared to equivalently sized regions randomly sampled from genome. *P*-value is given only when the difference is significant. SNV SNV count per site, Indel InDel count per site, Genome randomly sampled genome-wide regions, Rd Rd repeats, RdF Rd flanking regions (including upstream and downstream 50 bp, respectively), Rp Rp repeats, RpF Rp flanking regions (including upstream and downstream 50 bp, respectively), Rt Rt repeats, RtF Rt flanking regions (including upstream and downstream 50 bp, respectively), **c** Multiple sequence alignment of Rt5 and flanking sequences of *Oryza* species using *L. japonica* as outgroup to show independent occurrence of Rt5 in *O. sativa* ssp. *indica* and *O. rufipogon*. Rt5 repeats are indicated by using blue horizontal arrows

Although the remarkable feature of a chloroplast genome is the conservation of its most prominent structure and genome size^62^, our results showed that there was a gradual growth of rice chloroplast genomes in size with an increase in genetic distance from cultivated rice in the genus *Oryza* (Fig. 1**;** Supplementary Table 2). To determine the contribution of repetitive DNA sequences to genome variation and evolution, we investigated their evolutionary dynamics across these closely related chloroplast genomes. In total, we identified 112 repeats in the 23 *Oryza* and *L. japonica* cp genomes that are under the three categories, namely palindromic (Rp), dispersed (Rd), and tandem (Rt) repeats (Supplementary Table 11). Among them, the shortest 61 repeats that ranged from 15 to 29 bp in length occurred most frequently (Supplementary Fig. 19A). Although the vast majority of repeats were located within non-coding regions, the rest were found in a number of protein-coding or tRNA genes (Supplementary Fig. 19B). Comparative genomic analyses revealed that evolutionary behaviors of the three types of repeat sequences (Rp, Rd, and Rt) were contrastingly different across *Oryza* cp genomes. Here we define repetitive DNA sequences that locate in orthologous genomic regions with similar lengths as shared repeats. In contrast to Rd and Rp repeats that mainly generated in the common ancestor and have subsequently preserved afterwards during the past ~15.3 Myr^60^, Rt repeats were fairly evolutionarily dynamic, representing the abundance of copy number variation of chloroplast repeats across *Oryza* lineages (Fig. 4a**;** Supplementary Table 12). Our visual examination of the conserved Rd and Rp repeats shared by all 24 *Oryzea*e cp genomes accurately documented numerous single or multiple independent SNVs and/or deletions across *Oryzeae* lineages, providing convincing evidence for that Rp evolve faster than Rd repeats within these rice plastomes (Fig. 4a**;** Supplementary Table 12). Comparisons of genomic variation levels (SNVs and InDels) within Rt, Rp, Rd, and their flanking regions with genome background revealed that Rp and flanking regions exhibited high levels of both SNVs and InDels, Rt and flanking regions only exhibited high levels of InDels, and Rd and flanking regions showed no difference in both SNVs and InDels (Fig. 4b). The findings together suggest that, in comparison the conserved Rd repeat, Rt and Rp repeats are highly variable that may serve as important but different forces to trigger rice chloroplast genome variation and evolution.

Although molecular mechanisms of how a novel repeated sequence *de novo* generates in a chloroplast genome remain largely unresolved, our results indicate that the tempos of repeat insertions are quite different among these closely related rice species while they evolve (Fig. 4a). The majority of repeat insertion events occurred at differently timetabled nodes, that is, they may be well illuminated by their phylogenetic relationships; Rt5 may represent a case that occurred independently in *O. sativa* ssp. *indica* and *O. rufipogon* (Fig. 4c). To look into the rapidity of repeats inserted into or removed from the *Oryza* plastomes, we approximately mapped the occurrences at the timetabled phylogenetic tree and estimated the speed of their insertion into and/or removal from the *Oryza* plastomes (Fig. 4a). The detection of 54 insertion and 10 deletion events suggests the accumulation of repeat sequences in rice plastomes during the past ~15.3 Myr^60^.

In sharp contrast of recent and rapid evolution of transposable elements observed in *Oryza* nuclear genomes^18,38,63^, the majority of chloroplast Rp and Rd repeats possess fairly slow turnover rates since the generation in the common ancestor of these *Oryza* species, except for Rt repeats that evolve quickly. Of the three repeat types, Rt, accounting for 40.71% of total repeats, stood out as the most common one, while Rp and Rd were 36.28% and 23.01% of overall repeats, respectively (Supplementary Fig. 19C). These findings reveal an increased density of chloroplast repeats (e.g., Rt in particular) in *Oryza* chloroplast genomes as a result of relatively fewer deletions than insertions after they came into chloroplast genomes, may partially account for the growth of chloroplast genomes in the genus *Oryza* (Fig. 1**;** Supplementary Table 2).

The availability of the 24 *Oryzeae* complete chloroplast genomes makes it also possible to study the molecular evolution of rice chloroplast SSRs (Supplementary Table 13; Supplementary Fig. 20). Genome-wide characterization of SSRs showed that numbers of the four types of SSRs, including dinucleotide, trinucleotide, tetranucleotide, and pentanucleotide motifs, ranged from 70 to 81 with an average density of ~0.55 SSRs per kb. Numbers of all SSRs varied from 5 to 13, and the average density was 0.07 SSRs per Kb across the 24 genomes. Among the four motifs of SSRs, trinucleotide SSRs were the most abundant with the occurrence frequencies of ~52.7–59.21%, while pentanucleotide SSRs were the rarest (0–1.39%; Supplementary Table 14; Supplementary Fig. 20). Comparison analyses of *Oryza* cp genomes detected a total of 17 specific SSRs that are indicative of their incessant generation although tempos were quite different among these closely related rice species. Of the 30 SSRs that were shared by at least two chloroplast genomes, 17 SSRs were shared by more than 14 *Oryzea*e cp genomes, suggesting their relatively early insertions, the majority of which may be well illuminated by their phylogenetic relationships. Note that these conserved SSRs varied by repeat length polymorphisms together with SNVs and InDels (Supplementary Table 14), indicating the nature of evolutionary dynamics and potential applications of highly conserved polymorphic chloroplast SSRs to genetics studies of rice and closely related species.

### Genome-wide scanning for footprints of positively selected chloroplast genes and adaptive evolution of sun-loving and shade-tolerant *Oryza* species

Non-synonymous (*dN*), synonymous (*dS*) nucleotide substitution rates and non-synonymous/synonymous rate ratio (*ω* = *dN*/*dS*) are widely used as indicators of accelerated evolution and positive selection or adaptive evolution. The elevation of ω ratios can be due to either by adaptive evolution or relaxation of selective constraints. To examine molecular evolution of chloroplast protein-coding genes in the genus *Oryza*, we first calculated *dN*, *dS*, and *ω* along different lineages for all 77 rice chloroplast protein-coding genes. We observed that *ω* values slightly varied among these *Oryza* lineages, which is in a good agreement with the phylogenetic relationships (Fig. 1; Supplementary Figs. 7–10). Along the terminal branches, the basal lineages of *O. granulata* (GG- genome; 0.1843) and *O. meyeriana* (GG- genome; 0.1826) had the largest *ω* estimates, *O. brachyantha* (FF- genome) (0.1417) showed the smallest while the remainder had intermediate values (Supplementary Table 15).

To identify the positively selected chloroplast genes that may be beneficial in rice breeding programs, we performed CodeML and a series of LRTs to calculate the ratio of *dS* and *dN* changes at each codon on particular branches or clades of interest in the *Oryza* phylogeny for all 77 chloroplast genes. Applying the likelihood method and a *P*-value of 5% for statistical significance (FDR < 0.05), we convincingly obtained a total of 14 non-redundant positively selected genes (PSGs) in all tests (*accD*, *matK*, *ndhD*, *ndhF*, *ndhH*, *psaA*, *psbB*, *psbD*, *psbH*, *rbcL*, *rpl16*, *rpoA*, *rpoC2*, and *ycf68*; Supplementary Table 16), accounting for ~17.95% of all examined chloroplast genes. Note that the 14 PSGs (*accD*, *matK*, *ndhD*, *ndhF*, *ndhH*, *psaA*, *psbB*, *psbD*, *psbH*, *rbcL*, *rpl16*, *rpoA*, *rpoC2*, and *ycf68*) detected by using branch/clade-specific LRTs covered all eight PSGs (*accD*, *matK*, *ndhF*, *ndhH*, *psbH*, *rbcL*, *rpoC2*, and *ycf68*) with site model tests for all branches (Supplementary Table 16). A total of 10, 2, and 2 PSGs were found in the LSC, SSC, and IR regions, respectively (Supplementary Table 17), suggesting that rice chloroplast genes located in the LSC region is prone to being under positive selection. These 14 PSGs were functionally assigned as three self-replication genes, eight photosynthesis genes, one other gene, and two genes with unknown functions, indicating that the majority of positively selected genes are relevant to photosynthesis system (Supplementary Table 18). These proteins-coding genes often function chloroplast protein synthesis, gene transcription, energy transformation and regulation, and particularly photosynthesis (Supplementary Table 19).

**Fig. 5:**
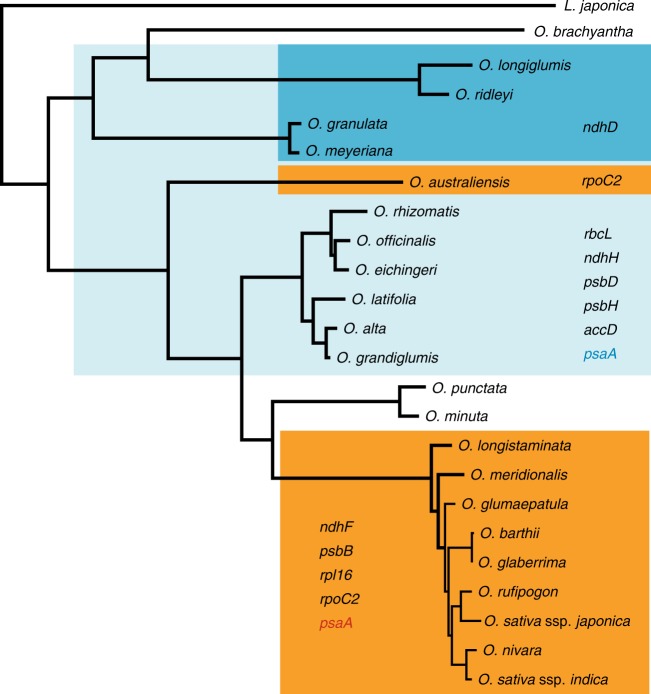
Genome-wide scanning for positively selected chloroplast genes in the lineages of the genus *Oryza* and adaptive evolution of sun-loving and shade-tolerant or shade-loving rice species. Branches in rectangle represent rice lineages where genes are significantly under positive selection (*P* < 0.05; FDR < 0.05). Sun-loving rice species are indicated in red, while shade-tolerant and shade-loving rice species are designated in light blue and dark blue, respectively. *psaA* is under adaptive evolution in sun-loving rice species (in red) and shade-tolerant rice species (in blue)

The genus *Oryza* includes both sun-loving and shade-tolerant rice species, providing an ideal model to understand the role of natural selection in driving the chloroplast genome evolution and identify key chloroplast genes underlying adaptive evolution of very closely related plant species. Strikingly, the photosynthesis gene *ndhD* has shown signatures of positive selection in typically shade-tolerant rice species, including *O. longiglumis* (HHJJ- genome), *O. ridleyi* (HHJJ- genome), *O. meyeriana* (GG- genome), and *O. granulata* (GG- genome; Fig. 5; Supplementary Table 20**;** Supplementary Fig. 21). Moreover, the four other photosynthesis genes, *rbcL, ndhH*, *psbD*, *psbH* and/or unknown functional gene *accD* were found to be under positive selection in either the above-mentioned shade-tolerant species or other rice species that naturally prefer to grow in shade habitats, including *O. officinalis* (CC- genome), *O. rhizomatis* (CC- genome), *O. eichingeri* (CC- genome), *O. alta* (CCDD- genome)*, O. grandiglumis* (CCDD- genome), and *O. latifolia* (CCDD- genome) (Fig. 5). Remarkably, photosynthesis genes (*ndhF*, *psbB*) and self-replication genes (*rpoC2*, *rpl16*) were under strong positive selection in sun-loving rice species, such as Asian cultivated rice *O. sativa*, African cultivated rice *O. glaberrima*, and other AA- genome species (Fig. 5; Supplementary Table 20**;** Supplementary Fig. 21). Besides these lineage-specific PSGs that are relevant to ecological adaptation to sun or shade habitats, the photosynthesis gene *psaA* appeared positively selected in both shade-tolerant and sun-loving rice species, probably displaying adaptive evolution under differently ecological conditions. Furthermore, species-specific tests unambiguously detected the four PSGs (*ndhD, ndhF, psbB*, and *rpoC2;* Fig. 5; Supplementary Table 21), strongly supporting that they are under independent natural selection. Of them, *ndhF* and *psbB* were under positive selection exclusively in *O. sativa* ssp. *japonica*, *ndhD* was positively selected in *O. longiglumis* (HHJJ- genome), and *rpoC2* was positively selected in *O. autraliensis* (EE- genome). These chloroplast functional genes may have played key roles in the adaptation of rice species to ecological habitats related to sunlight preferences. Knowledge of chloroplast genes under positive selection may provide valuable clues for understanding how these rice species adapt to diverse ecological niches in Asia, South America, Africa, and Australia. Moreover, the detected genes or sites targeted by selection will offer more opportunities for further functional and evolutionary analyses.

In conclusion, we generated complete chloroplast genomes of 22 closely related *Oryza* species in chronologically ordered stages. We performed deep comparisons that permit us, to our knowledge, to make the first accurate map of genomic structural variation in the course of recent plant speciation. We estimated the occurrence rate on average to be ~7 insertions and ~15 deletions per Myr in the genus *Oryza*. We surprisingly found the swelling of *Oryza* chloroplast genome sizes, mainly due to the relatively fewer deletions than insertions that result in an increased repeat density. Comparative genomic analysis of repeat sequences further suggests that evolutionary dynamics of palindromic, dispersed, and tandem repeats exhibited remarkably dissimilar, probably due to either the diverged turnover tempos or evolutionary rates of SNVs and/or deletions after initial insertions. We accomplished genome-wide scanning and convincingly detected a total of 14 cp genes with strong signatures of positive selection, which are mainly related to photosynthesis system. We observed that eight of them were separately selected in shade-tolerant (*ndhD*, *rbcL, ndhH*, *psbD*, *psbH*, and *accD*) or sun-loving (*ndhF* and *psbB*) *Oryza* species, while *psaA* was positively selected in both shade-tolerant and sun-loving rice species. Such a comparative genomic analysis of the *Oryza* cp genomes offers us an unprecedented opportunity to reveal footprints of adaptive evolution of chloroplast genes that makes rice species adapt to diverse ecological habitats related to sunlight preferences. Thus, this study provides new insights into the course, tempo and mode of sequence evolution and architectural variations that will greatly further our understanding about the micro-evolution of cp genes and genomes in recent plant speciation.

## Materials and methods

### Plant materials

A total of 22 species were used for chloroplast genome sequencing, including 20 *Oryza* species, corresponding to the nine genome types (AA, BB, CC, EE, FF, GG, BBCC, CCDD, and HHJJ), and *L. japonica* as outgroup. Detailed information of the sampled species is given in Supplementary Table 2, including their scientific names, accession numbers or vouchers, and countries of geographical origins. They were harvested from plants grown at the greenhouse of Germplasm Bank of Wild Species in Southwest China, Kunming Institute of Botany, Chinese Academy of Sciences, China. Voucher herbarium specimens (8045673-8045694) were deposited at the Herbarium of Kunming Institute of Botany (KUN), Kunming, China. The chloroplast genome sequences of *O. sativa* L. ssp. *japonica* (NC_001320)^64^ and *O. sativa* L. ssp. *indica* (NC_008155)^31^ were directly downloaded from NCBI (http://www.ncbi.nlm.nih.gov/genomes/GenomesGroup.cgi? taxid = 2759&opt = plastid).

### Chloroplast DNA isolation, amplification, and sequencing

We collected 50–100 g of fresh leaves from each studied *Oryza* species and *L. japonica* to isolate the purified cpDNA with an improved high salt method as reported previously^65^. They were amplified using a total of 14 primer pairs (Supplementary Table 22), which were designed according to the chloroplast sequence of *O. nivara* (NC_005973)^25^, and then PCR products were mixed for sequencing. The isolated cpDNA of the studied species was sequenced using Illumina sequencing platform. We used 40 µg of cpDNA for the fragmentation by nebulization with compressed nitrogen gas, and constructed short-insert (500 bp) libraries following the manufacturer’s protocol. cpDNA from the different species was indexed by tags and pooled together in one lane of Illumina’s Genome Analyzer, and sequence reads of 2 × 100 bp were obtained at Germplasm Bank of Wild Species in Southwest China, Kunming Institute of Botany, Chinese Academy of Sciences, China.

### Genome assembly and annotation

To determine the proportion of cpDNA, we mapped sequence reads to the cp genome of *O. sativa* ssp. *japonica* (NC_001320) using Bowtie^64^ with paired-end alignment and a maximum of three mismatches (-*v* = 3) as raw sequence reads may include non-cp DNA. Subsequently, we assembled raw sequence reads into contigs with a minimum length of 100 bp using SOAPdenovo^66^ with an overlap length of 27 bp. The contigs were aligned to the reference genome using BLAST (http://blast.ncbi.nlm.nih.gov/), and aligned contigs (**≥**90% similarity and query coverage) were ordered according to the reference cp genome, gaps between the *de novo* contigs were replaced with consensus sequences of raw reads mapped to the reference genome. Also based on the reference genome, we designed primer pairs for closing gaps and low-coverage regions, respectively. PCR products were sequenced using standard Sanger protocols on ABI 3730 xl instruments. Sanger sequences and assembled genomes were aligned with ClustalW 1.83 program^67^ to determine if there were any differences. To check the accuracy of sequence assembly, we verified regions with ambiguous read mapping (conflicted reads mapped to the same genomic region) and low coverage (≤2 reads) by PCR amplifications and Sanger sequencing. Here, 1–20 primer pairs (Supplementary Table 23) were used to close the gaps and verify the assembly accuracy.

The chloroplast genomes were annotated using DOGMA (Dual Organellar GenoMe Annotator^68^, http://bugmaster.jgi-psf.org/dogma/). Both tRNAs and rRNAs are identified by BLASTN searches against the same database of chloroplast genomes. Chloroplast genome of *O. sativa* L. ssp. *japonica* (NC_001320)^64^ and *O. sativa* L. ssp. *indica* (NC_001320)^31^ were again annotated. These chloroplast genomes were drawn using OGDRAW v1.1^69^. Full alignments with annotations were visualized by the VISTA viewer (Supplementary Fig. 1).

To detect and characterize genomic variation (SNVs and InDels) the *Oryza* chloroplast genomes were compared with *L. japonica* using the program MAFFT v5^70^, following by manual examination. The distribution of genomic variation among the chloroplast genomes was calculated and visualized using the Circos-0.64^71^.

### Phylogenomic analyses

Phylogenomic analyses were performed based on the complete cp genome sequences, a set of 77 common protein-coding genes and non-coding DNA sequences from 22 *Oryza* species including two rice subspecies (*O. sativa* L. ssp. *indica* and *O. sativa* L. ssp. *japonica*) using *L. japonica* as outgroup. All of the cp genome sequences were aligned using the program MAFFT version 5^70^ and adjusted manually where necessary. MP analyses were performed with PAUP*4.0b10 ^72^. Heuristic tree searches were conducted with 1000 random-taxon-addition replicates and tree bisection-reconnection (TBR) branch swapping with multrees option. Non-parametric bootstrap analysis was executed under 1000 replicates with TBR branch swapping. Maximum likelihood (ML) analyses were implemented in RAxML version 7.2.6^73^. RAxML searches relied on the general time reversible (GTR) model of nucleotide substitution with the gamma model of rate heterogeneity. Non-parametric bootstrapping as implemented in the fast bootstrap algorithm of RAxML used 1000 replicates. Bayesian analyses were completed using the program MrBayes version 3.1.2^74^. The best-fitting models were determined using the Akaike Information Criterion as implemented in the program Modeltest 3.7^75^. The Markov Chain Monte Carlo (MCMC) algorithm was run for 200,000 generations with trees sampled every 10 generations for each data partition. The first 25% of trees from all runs were discarded as burn-in, and the remaining trees were used to construct majority-rule consensus tree. *L. japonica* was set as outgroup and all gaps introduced by the alignment were excluded. InDels were mapped onto the phylogenetic trees determined by ML analyses of whole cp genome alignment using the ClustalW1.83 program^67^.

### Genome-wide analyses of genomic structural variants

The sequences of 23 rice complete chloroplast genomes allowed a comprehensive assessment of genome-wide structural variation. All datasets were aligned by a phylogeny-aware algorithm PRANK_+F_^58^, which has been shown to outperform traditional methods with regard to the accuracy in indel-rich sequences. In PRANK_+F,_ we invoked the _+F_ option to fix already inferred InDels at their places and avoid another indel to be inferred in an overlapping position during the second recursion of the algorithm. This reflects the notion that InDels introduced along one branch of the phylogeny are from an indel event another branch, even if they overlap, and thus produces more gappy but potentially more accurate alignments.

### Repeat sequence analyses

The repeat sequences were divided into three categories: dispersed, tandem, and palindromic repeats. The minimal copy sizes investigated were 30 bp for dispersed, 15 bp for tandem, and 20 bp for palindromic repeats, respectively. We determined these three repeat types by first applying the program DNAMAN Version 6.0.3.99 (Lynnon Biosoft, Vaudreuil, Quebec, Canada) and then manually filtering the redundant output. The chloroplast simple sequence repeats (SSRs) were searched by the SSRHunter software (http://www.biosoft.net)^76^. The number of dinucleotide ≥8 bp, trinucleotide ≥9 bp, tetranucleotide ≥12 bp, and pentanucleotide ≥15 bp loci were counted, respectively.

### Accelerated evolution of protein-coding genes

We investigated the molecular evolution of all 77 chloroplast genes in the genus *Oryza*. All the orthologous genes were aligned by ClustalW^67^, and then used to the detection of positive selection and evolutionary rate analyses. We analyzed this set of well-aligned 1:1 orthologous gene to obtain and compare the average evolutionary rates of protein-coding genes along lineages and clades of the 23 species phylogeny. *dN*, *dS*, and *ω* were estimated using the codonML^77^ program in the PAML software (version 4.4)^78^. The two models with (1) a single *ω* estimated for all branches and (2) branch-specific *ω* for each branch were compared in a likelihood ratio test (LRT). The experiments were replicated 10 times.

### Genome-wide scan for positive selection

Positive selection was tested for all 77 chloroplast genes of the genus *Oryza* using the widely employed codon-based substitution models and LRTs implemented in the program PAML version 4.4^78^. In all measurements, codon frequencies were estimated from nucleotide frequencies at each codon position (model F3 × 4). Briefly speaking, our LRT for selection on any branch of the phylogenetic tree was compared site model 1a (nearly neutral) against 2a (selection)^79^, while the branch/clade-specific LRTs were based on branch-site models^80,81^, which compared the modified model A with the corresponding null model with *ω*_2_ = 1 fixed (fix_omega = 1 and omega = 1). In the M1a-M2a comparison, the degree of freedom df *=* 2 was used, with the critical values to be 5.99 and 9.21 at 5 and 1% significance levels, respectively. For the lineage-specific and clade-specific LRTs, *P*-values were computed assuming that the null distribution was a 50:50 mixture of point mass 0 and χ^2^_df*=1*_. Identifying sites under positive selection was achieved by using the Bayes empirical Bayes (BEB)^82^ to calculate the posterior probabilities for site classes. Multiple comparisons were performed by following the method^83^ of to estimate the appropriate *P*-value threshold for a false discovery rate (FDR) of <0.05.

### Reporting summary

Further information on research design is available in the Nature Research Reporting Summary linked to this article.

## Supplementary information

Supplementary Information

Reporting Summary

## Data Availability

The data that support the findings of this study are available from the corresponding author upon reasonable request. The obtained 22 chloroplast genomes are publicly available in NCBI GenBank under accession numbers (KF359901-KF359922).

## References

[1] Dyall SD, Brown MT, Johnson PJ (2004). Ancient invasions: from endosymbionts to organelles. Science.

[2] Birky CW (1995). Uniparental inheritance of mitochondrial and chloroplast genes: mechanisms and evolution. Proc. Natl Acad. Sci. USA.

[3] Soltis DE (2004). Genome-scale data, angiosperm relationships, and “ending incongruence”, a cautionary tale in phylogenetics. Trends Plant Sci..

[4] Jansen RK (2007). Analysis of 81 genes from 64 plastid genomes resolves relationships in angiosperms and identifies genome-scale evolutionary patterns. Proc. Natl Acad. Sci. USA.

[5] Moore MJ, Soltis PS, Bell CD, Burleigh JG, Soltis DE (2010). Phylogenetic analysis of 83 plastid genes further resolves the early diversification of eudicots. Proc. Natl Acad. Sci. USA.

[6] Palmer JD (1985). Comparative organization of chloroplast genomes. Annu. Rev. Genet..

[7] Sugiura M (1992). The chloroplast genome. Plant Mol. Biol..

[8] Vaughan, D. A. *Wild Relatives of Rice: A Genetic Resource Handbook* 3–8 (Int. Rice Res. Inst., Manila, 1994).

[9] Khush GS (1997). Origin, dispersal, cultivation and variation of rice. Plant Mol. Biol..

[10] Nayar NM (1973). Origin and cytogenetics of rice. Adv. Genet..

[11] Aggarwal RK, Brar DS, Khush GS (1997). Two new genomes in the *Oryza* complex identified on the basis of molecular divergence analysis using total genomic DNA hybridization. Mol. Gen. Genet..

[12] Ge S, Sang T, Lu BR, Hong DY (1999). Phylogeny of rice genomes with emphasis on origins of allotetraploid species. Proc. Natl Acad. Sci. USA.

[13] Second, G. in *Advances in chromosome and cell genetics* (eds Sharma A. K. & Sharma A.) 45–48 (Oxford and IBH, New Delhi, 1985).

[14] Wang ZY, Second G, Tanksley SD (1992). Polymorphism and phylogenetic relationships among species in the genus *Oryza* as determined by analysis of nuclear RFLPs. Theor. Appl. Genet..

[15] Mullins I, Hilu K (2002). Sequence variation in the gene encoding the 10-kDa prolamin in *Oryza* (Poaceae). I. Phylogenetic implications. Theor. Appl. Genet..

[16] Zou XH (2008). Analysis of 142 genes resolves the rapid diversification of the rice genus. Genome Biol..

[17] Zhu T (2013). Phylogenetic relationships and genome divergence among the AA- genome species of the genus *Oryza* as revealed by 53 nuclear genes and 16 intergenic regions. Mol. Phylogenet. Evol..

[18] Zhang QJ (2014). Rapid diversification of five Oryza AA genomes associated with rice adaptation. Proc. Natl Acad. Sci. USA.

[19] Bao Y, Ge S (2004). Origin and phylogeny of *Oryza* species with the CD genome based on multiple-gene sequence data. Plant Syst. Evol..

[20] Guo YL, Ge S (2005). Molecular phylogeny of *Oryzeae* (Poaceae) based on DNA sequences from chloroplast, mitochondrial, and nuclear genomes. Am. J. Bot..

[21] Nishikawa T, Vaughan AD, Kadowaki K (2005). Phylogenetic analysis of *Oryza* species, based on simple sequence repeats and their flanking nucleotide sequences from the mitochondrial and chloroplast genomes. Theor. Appl. Genet..

[22] Kumagai M, Wang L, Ueda S (2010). Genetic diversity and evolutionary relationships in genus *Oryza* revealed by using highly variable regions of chloroplast DNA. Gene.

[23] Tang L (2010). Phylogeny and biogeography of the rice tribe (*Oryzeae*): evidence from combined analysis of 20 chloroplast fragments. Mol. Phylogenet. Evol..

[24] Hiratsuka J (1989). The complete sequence of the rice (*Oryza sativa*) chloroplast genome: Intermolecular recombination between distinct tRNA genes accounts for a major plastid DNA inversion during the evolution of the cereals. Mol. Gen. Genet..

[25] Masood MS (2004). The complete nucleotide sequence of wild rice (*Oryzanivara*) chloroplast genome: first genome wide comparative sequence analysis of wild and cultivated rice. Gene.

[26] Waters DLE, Nock CJ, Ishikawa R, Rice N, Henry RJ (2012). Chloroplast genome sequence confirms distinctness of Australian and Asian wild rice. Ecol. Evol..

[27] Wu Z, Ge S (2014). The whole chloroplast genome of wild rice (*Oryza australiensis*). Mitochondrial. DNA.

[28] Mariac C (2014). Cost-effective enrichment hybridization capture of chloroplast genomes at deep multiplexing levels for population genetics and phylogeography studies. Mol. Ecol. Resour..

[29] Kim K (2015). Complete chloroplast and ribosomal sequences for 30 accessions elucidate evolution of *Oryza* AA genome species. Sci. Rep..

[30] Wambugu PW, Brozynska M, Furtado A, Waters DL, Henry RJ (2015). Relationships of wild and domesticated rices (*Oryza* AA genome species) based upon whole chloroplast genome sequences. Sci. Rep..

[31] Tang JB (2004). A comparison of rice chloroplast genomes. Plant Physiol..

[32] Provan J (1996). DNA fingerprints of rice (*Oryza sativa*) obtained from hypervariable simple sequence repeats. Proc. R. Soc. Lond. Ser. B.

[33] Kawakami S (2007). Genetic variation in the chloroplast genome suggests multiple domestication ofcultivated Asian rice (*Oryza sativa* L.). Genome.

[34] Wei X (2012). Origin of *Oryza sativa* in China inferred by nucleotide polymorphisms of organelle DNA. PLoS One.

[35] Goff SA (2002). A draft sequence of the rice genome (*Oryza sativa*L. ssp. *japonica*). Science.

[36] Yu J, Yang H (2002). A draft sequence of the rice genome (*Oryza sativa* L. ssp. *indica*). Science.

[37] International Rice Genome Sequencing Project. (2005). The map-based sequence of the rice genome. Nature.

[38] Chen J (2013). Whole-genome sequencing of *Oryza brachyantha* reveals mechanisms underlying *Oryza* genome evolution. Nat. Commun..

[39] Shendure J, Ji H (2008). Next-generation DNA sequencing. Nat. Biotechnol..

[40] Yue F, Cui L, DePamphilis CW, Moret BME, Tang JJ (2008). Gene rearrangement analysis from chloroplast genomes with inverted repeat. BMC Genomics.

[41] Jansen RK (2005). Methods for obtaining and analyzing whole chloroplast genome sequences. Methods Enzymol..

[42] Raubeson, L. A., Jansen, R. K. in *Diversity and Evolution of Plants; Genotypic and Phenotypic Variation in Higher Plants* (ed. Henry R.) 45–68 (CABI Publishing, London, 2005).

[43] Martin W (2002). Evolutionary analysis of *Arabidopsis*, cyanobacterial, and chloroplast genomes reveals plastid phylogenyand thousands of cyanobacterial genes in the nucleus. Proc. Natl Acad. Sci. USA.

[44] Bock R (2005). Extranuclear inheritance: gene transfer out of plastids. Prog. Bot..

[45] Matsuo M, Ito Y, Yamauchi R, Obokata J (2005). The rice nuclear genome continuously integrates, shuffles, and eliminates the chloroplast genome to cause chloroplast-nuclear DNA flux. Plant Cell.

[46] Stegemann S, Bock R (2006). Experimental reconstruction of functional gene transfer from the tobaccoplastid genome to the nucleus. Plant Cell.

[47] Millen RS (2001). Many parallel losses of infA from chloroplast DNA during angiosperm evolution with multiple independent transfers to the nucleus. Plant Cell.

[48] Magee AM (2010). Localized hypermutation and associated gene losses in legume chloroplast genomes. Genome Res..

[49] Raubeson LA, Peery R, Chumley TW (2007). Comparative chloroplast genomics: analyses including new sequences from the angiosperms *Nuphar advena* and *Ranunculus macranthus*. BMC Genomics.

[50] Lee HL, Jansen RK, Chumley TW, Kim KJ (2007). Gene relocations withinchloroplast genomes of *Jasminum* and *Menodora* (Oleaceae) are due to multipleoverlapping inversions. Mol. Biol. Evol..

[51] Yi DK, Kim KJ (2012). Complete chloroplast genome sequences of important oilseed crop *Sesamum indicum L*. PLoS One.

[52] Morinaga T, Nakajima K, Yumen T (1939). The size and form of rice caryopses, and their mode of inheritance. Genes Genet. Syst..

[53] Morinagam T, Magamatu T, Kawahara E (1943). New linkage relations in rice. Genes Genet. Syst..

[54] Jena KK, Kochert G (1991). Restriction fragment length polymorphism analysis of CCDD genome species of the genus *Oryza* L. Plant Mol. Biol..

[55] Aggarwal RK, Brar DS, Nandi S, Huang N, Khush GS (1999). Phylogenetic relationships among *Oryza* species revealed by AFLP markers. Theor. Appl. Genet..

[56] Buso GSC, Range PH, Ferreira ME (2001). Analysis of random and specific sequences of nuclear and cytoplasmic DNA in diploid and tetraploid American wild rice species (*Oryza* spp.). Genome.

[57] Roschevicz RI (1931). A contribution to the study of rice. Bull. Appl. Bot. Genet. Plant Breed..

[58] Löytynoja A, Goldman N (2008). A model of evolution and structure for multiple sequence alignment. Philos. Trans. R. Soc. B.

[59] Hu TT (2011). The *Arabidopsislyrata* genome sequence and the basis of rapid genome size change. Nat. Genet..

[60] Zou XH, Yang Z, Doyle JJ, Ge S (2013). Multilocus estimation of divergence times and ancestral effective population sizes of *Oryza* species and implications for the rapid diversification of the genus. New Phytol..

[61] Wolfe KH, Li WH, Sharp PM (1987). Rates of nucleotide substitution vary greatly among plant mitochondrial, chloroplast and nuclear DNA. Proc. Natl Acad. Sci. USA.

[62] Palmer JD, Nugent JM, Herbon LA (1987). Unusual structure of geranium chloroplast DNA: a triple-sized inverted repeat, extensive gene duplications, multiple inversions, and two repeat families. Proc. Natl Acad. Sci. USA.

[63] Piegu B (2006). Doubling genome size without polyploidization: dynamics of retrotransposition-driven genomic expansions in *Oryza australiensis*, awild relative of rice. Genome Res..

[64] Shimada H, Sugiura M (1991). Fine structural features of the chloroplast genome: comparison of the sequenced chloroplast genomes. Nucleic Acids Res..

[65] Shi C (2012). An improved chloroplast DNA extraction procedure for whole plastid genome sequencing. PLoS One.

[66] Li RQ (2010). De novo assembly of human genomes with massively parallel short read sequencing. Genome Res..

[67] Thompson JD, Higgins DG, Gibson TJ (1994). CLUSTAL W: improving the sensitivity of progressive multiple sequence alignment through sequence weighting, position-specific gap penalties and weight matrix choice. Nucleic Acids Res..

[68] Wyman SK, Jansen RK, Boore JL (2004). Automatic annotation of organellar genomes with DOGMA. Bioinformatics.

[69] Lohse M, Drechsel O, Bock R (2007). Organellar Genome DRAW (OGDRAW): a tool for the easy generation of high-quality custom graphical maps of plastid and mitochondrial genomes. Curr. Genet..

[70] Katoh K, Kuma K, Toh H, Miyata T (2005). MAFFT version 5: improvement in accuracy of multiple sequence alignment. Nucleic Acids Res..

[71] Krzywinski M (2009). Circos: an information aesthetic for comparative genomics. Genome Res..

[72] Swofford, D. L. PAUP: phylogenetic analysis using parsimony (and other methods) ver.4.0. (Sinauer Associates, Sunderland, 2003).

[73] Stamatakis A (2006). RAxML-VI-HPC: maximum likelihood-based phylogenetic analyses with thousands of taxa and mixed models. Bioinformatics.

[74] Ronquist F, Huelsenbeck JP (2003). MrBayes 3: Bayesian phylogenetic inference under mixed models. Bioinformatics.

[75] Posada D, Crandall KA (1998). MODELTEST: testing the model of DNA substitution. Bioinformatics.

[76] Li Q, Wan JM (2005). SSRHunter: development of a local searching software for SSR sites. Yi Chuan.

[77] Yang Z (1998). Likelihood ratio tests for detecting positive selection and application to primate lysozyme evolution. Mol. Biol. Evol..

[78] Yang Z (2007). PAML 4: phylogenetic analysis by maximum likelihood. Mol. Biol. Evol..

[79] Nielsen R, Yang Z (1998). Likelihood models for detecting positively selected amino acid sites and applications to the HIV-1 envelope gene. Genetics.

[80] Yang Z, Nielsen R (2002). Codon-substitution models for detecting molecular adaption at individual sites along specific lineages. Mol. Biol. Evol..

[81] Zhang J, Nielsen R, Yang Z (2005). Evaluation of an improved branch-site likelihood method for detecting positive selection at the molecular level. Mol. Biol. Evol..

[82] Yang Z, Wong WS, Nielsen R (2005). Bayes empirical Bayes inference of amino acid sites under positive selection. Mol. Biol. Evol..

[83] Benjamini Y, Hochberg Y (1995). Controlling the false discovery rate: a practical and powerful approach to multiple testing. J. R. Stat. Soc. B.

