# Supplementary material for: Evolution of Oryza chloroplast genomes promoted adaptation to diverse ecological habitats
Source: Commun Biol. 2019 Jul 26;2:278. doi: 10.1038/s42003-019-0531-2 (PMC6659635; doi:10.1038/s42003-019-0531-2)
Supplement: Supplementary file 1 — Supplementary Information [file 42003_2019_531_MOESM1_ESM.pdf]

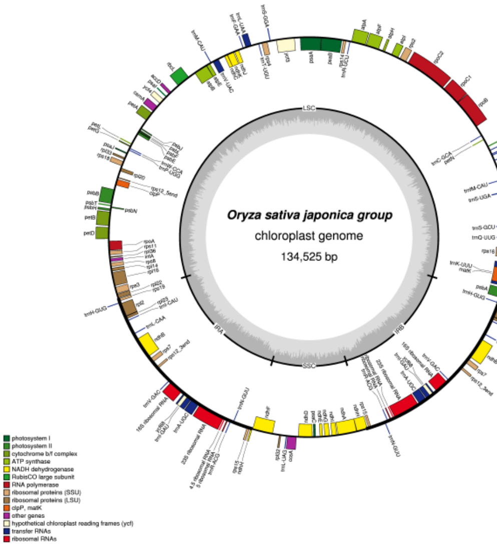
Oryza sativa japonica group
chloroplast genome
134,525 bp
photosystem I
photosystem II
cytochrome b/f complex
ATP synthase
NADH dehydrogenase
RubisCO large subunit
RNA polymerase
ribosomal proteins (SSU)
ribosomal proteins (LSU)
clpP, matK
other genes
hypothetical chloroplast reading frames (ycf)
transfer RNAs
ribosomal RNAs

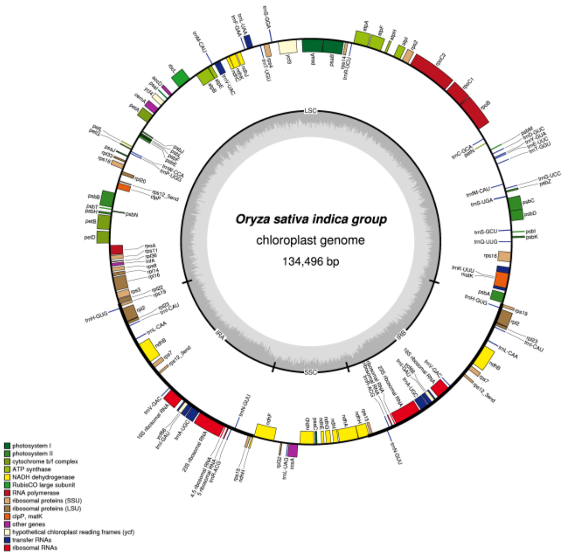
Oryza sativa indica group
chloroplast genome
134,496 bp
photosystem I
photosystem II
cytochrome b/f complex
ATP synthase
NADH dehydrogenase
RubisCO large subunit
RNA polymerase
ribosomal proteins (SSU)
ribosomal proteins (LSU)
clpP, matK
other genes
hypothetical chloroplast reading frames (ycf)
transfer RNAs
ribosomal RNAs

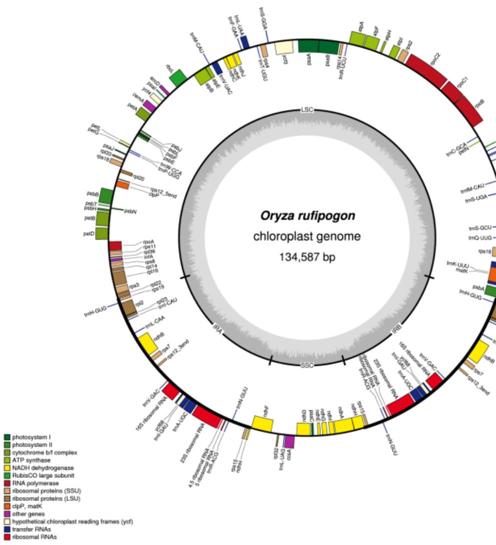
Oryza rufipogon
chloroplast genome
134,587 bp
photosystem I
photosystem II
cytochrome b/f complex
ATP synthase
NADH dehydrogenase
RubisCO large subunit
RNA polymerase
ribosomal proteins (SSU)
ribosomal proteins (LSU)
clpP, matK
other genes
hypothetical chloroplast reading frames (ycf)
transfer RNAs
ribosomal RNAs

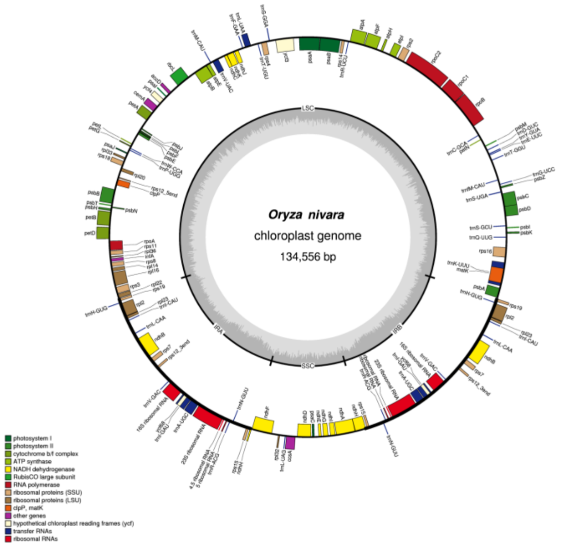
Oryza nivara
chloroplast genome
134,556 bp
photosystem I
photosystem II
cytochrome b/f complex
ATP synthase
NADH dehydrogenase
RubisCO large subunit
RNA polymerase
ribosomal proteins (SSU)
ribosomal proteins (LSU)
clpP, matK
other genes
hypothetical chloroplast reading frames (ycf)
transfer RNAs
ribosomal RNAs

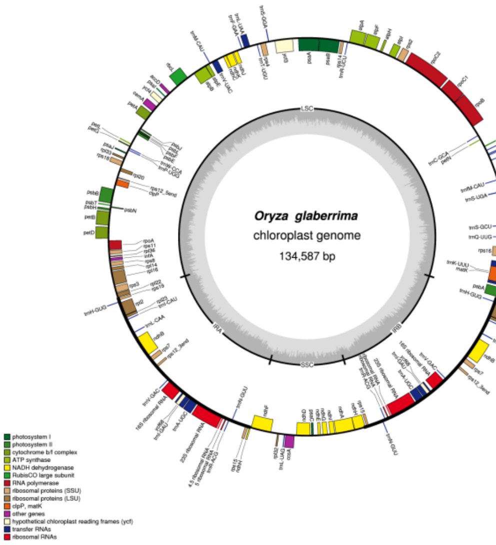
Oryza glaberrima
chloroplast genome
134,587 bp
photosystem I
photosystem II
cytochrome b/f complex
ATP synthase
NADH dehydrogenase
RubisCO large subunit
RNA polymerase
ribosomal proteins (SSU)
ribosomal proteins (LSU)
clpP, matK
other genes
hypothetical chloroplast reading frames (ycf)
transfer RNAs
ribosomal RNAs

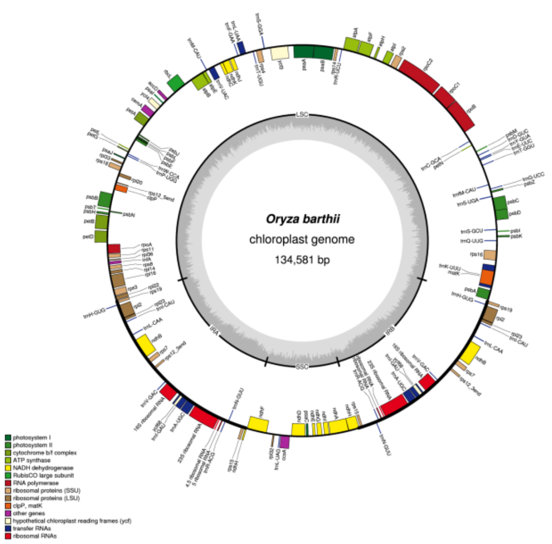
Oryza barthii
chloroplast genome
134,581 bp
photosystem I
photosystem II
cytochrome b/f complex
ATP synthase
NADH dehydrogenase
RubisCO large subunit
RNA polymerase
ribosomal proteins (SSU)
ribosomal proteins (LSU)
clpP, matK
other genes
hypothetical chloroplast reading frames (ycf)
transfer RNAs
ribosomal RNAs

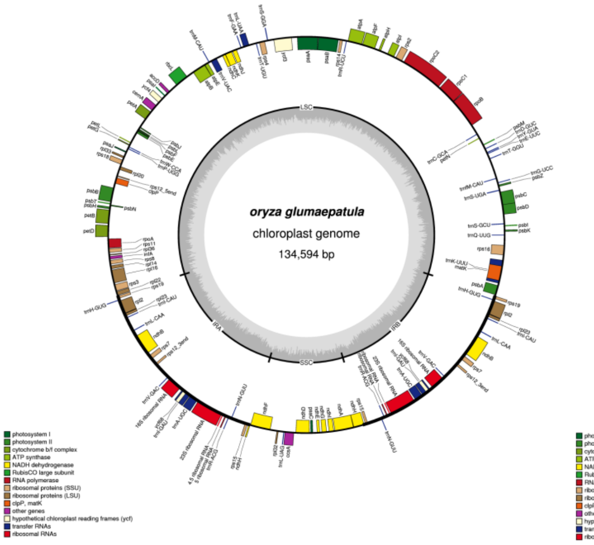
oryza glumaepatula
chloroplast genome
134,594 bp
photosystem I
photosystem II
cytochrome b/f complex
ATP synthase
NADH dehydrogenase
RubisCO large subunit
RNA polymerase
ribosomal proteins (SSU)
ribosomal proteins (LSU)
clpP, matK
other genes
hypothetical chloroplast reading frames (ycf)
transfer RNAs
ribosomal RNAs

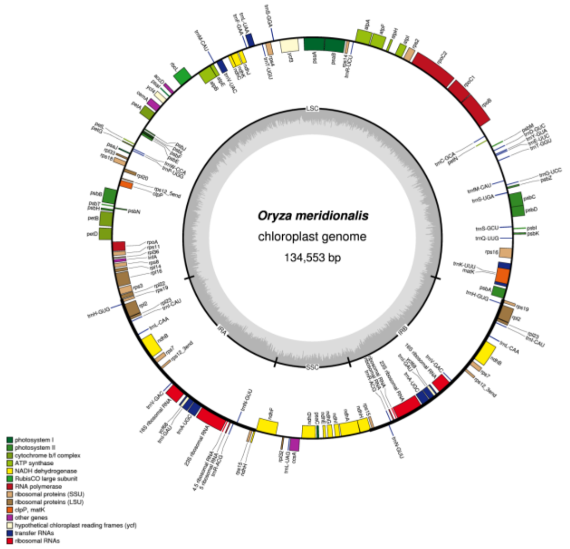
Oryza meridionalis
chloroplast genome
134,553 bp
photosystem I
photosystem II
cytochrome b/f complex
ATP synthase
NADH dehydrogenase
RubisCO large subunit
RNA polymerase
ribosomal proteins (SSU)
ribosomal proteins (LSU)
clpP, matK
other genes
hypothetical chloroplast reading frames (ycf)
transfer RNAs
ribosomal RNAs

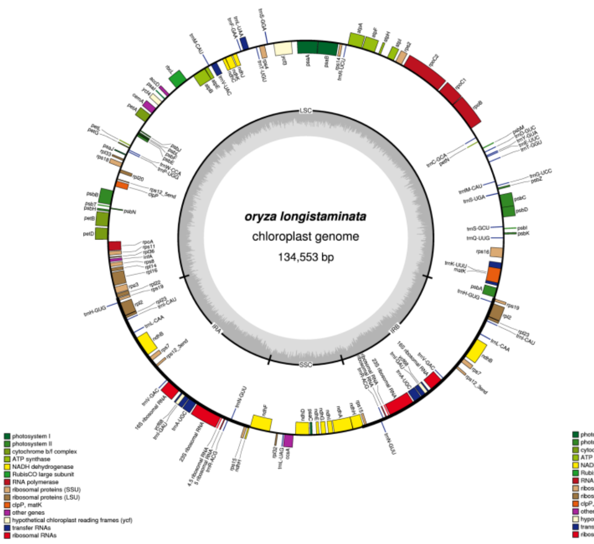
oryza longistaminata
chloroplast genome
134,553 bp
photosystem I
photosystem II
cytochrome b/f complex
ATP synthase
NADH dehydrogenase
RubisCO large subunit
RNA polymerase
ribosomal proteins (SSU)
ribosomal proteins (LSU)
clpP, matK
other genes
hypothetical chloroplast reading frames (ycf)
transfer RNAs
ribosomal RNAs

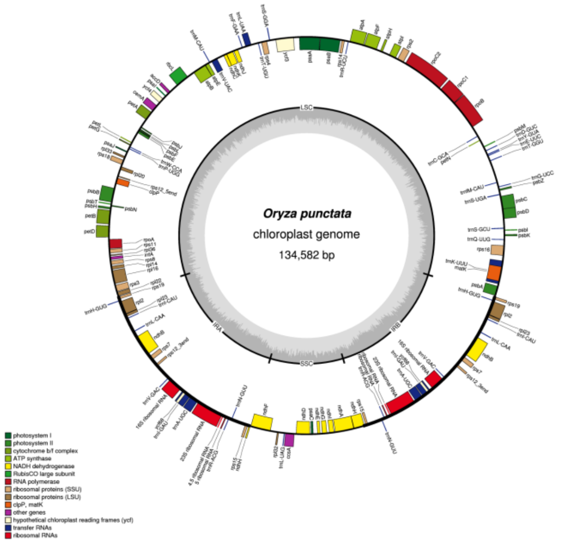
Oryza punctata
chloroplast genome
134,582 bp
photosystem I
photosystem II
cytochrome b/f complex
ATP synthase
NADH dehydrogenase
RubisCO large subunit
RNA polymerase
ribosomal proteins (SSU)
ribosomal proteins (LSU)
clpP, matK
other genes
hypothetical chloroplast reading frames (ycf)
transfer RNAs
ribosomal RNAs

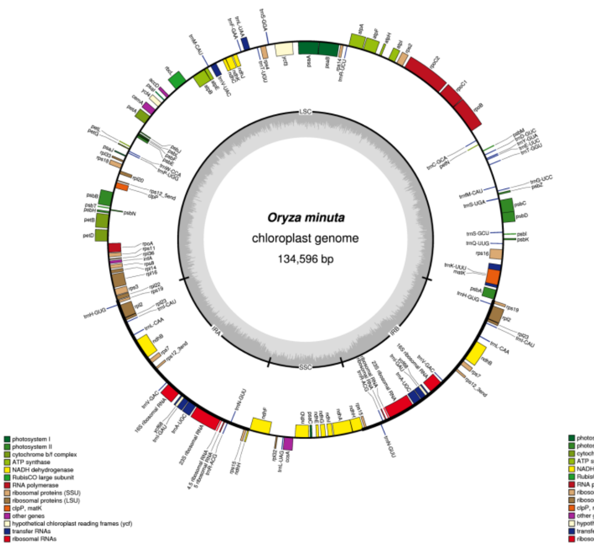
Oryza minuta
chloroplast genome
134,596 bp
photosystem I
photosystem II
cytochrome b/f complex
ATP synthase
NADH dehydrogenase
RubisCO large subunit
RNA polymerase
ribosomal proteins (SSU)
ribosomal proteins (LSU)
clpP, matK
other genes
hypothetical chloroplast reading frames (ycf)
transfer RNAs
ribosomal RNAs

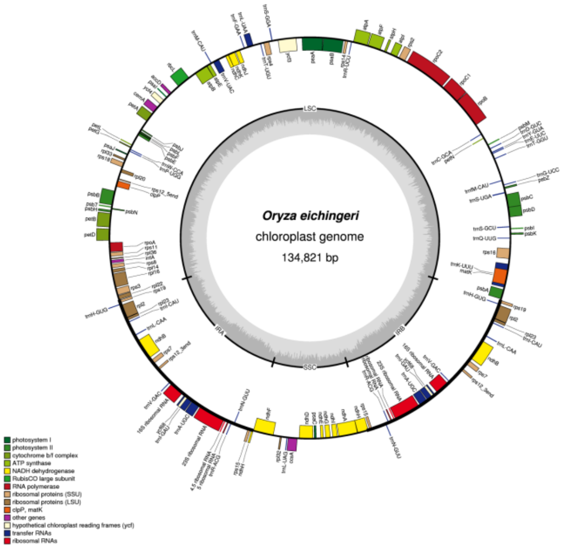
Oryza eichingeri
chloroplast genome
134,821 bp
photosystem I
photosystem II
cytochrome b/f complex
ATP synthase
NADH dehydrogenase
RubisCO large subunit
RNA polymerase
ribosomal proteins (SSU)
ribosomal proteins (LSU)
clpP, matK
other genes
hypothetical chloroplast reading frames (ycf)
transfer RNAs
ribosomal RNAs

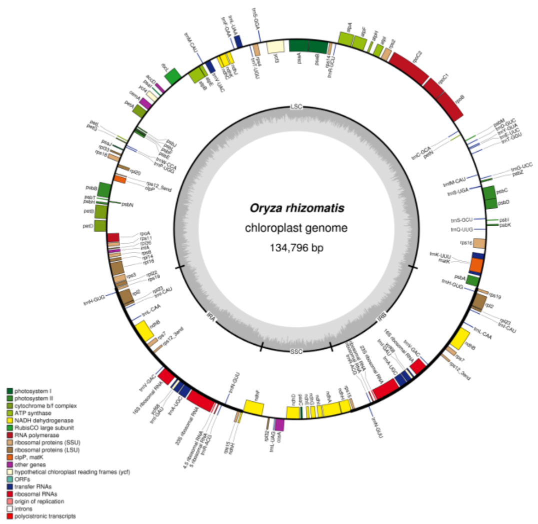
Oryza rhizomatis
chloroplast genome
134,796 bp
LSC
IRA
SSC
IRB
photosystem I
photosystem II
cytochrome b/f complex
ATP synthase
NADH dehydrogenase
RubisCO large subunit
RNA polymerase
ribosomal proteins (SSU)
ribosomal proteins (LSU)
clpP, matK
other genes
hypothetical chloroplast reading frames (ycf)
ORFs
transfer RNAs
ribosomal RNAs
origin of replication
introns
polycistronic transcripts

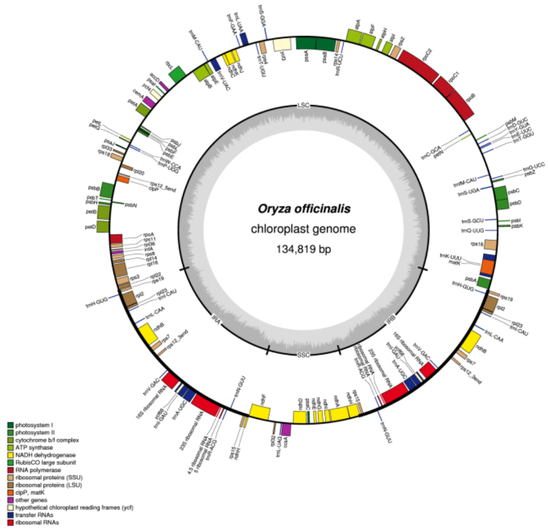
Oryza officinalis
chloroplast genome
134,819 bp
LSC
IRA
SSC
IRB
photosystem I
photosystem II
cytochrome b/f complex
ATP synthase
NADH dehydrogenase
RubisCO large subunit
RNA polymerase
ribosomal proteins (SSU)
ribosomal proteins (LSU)
clpP, matK
other genes
hypothetical chloroplast reading frames (ycf)
transfer RNAs
ribosomal RNAs

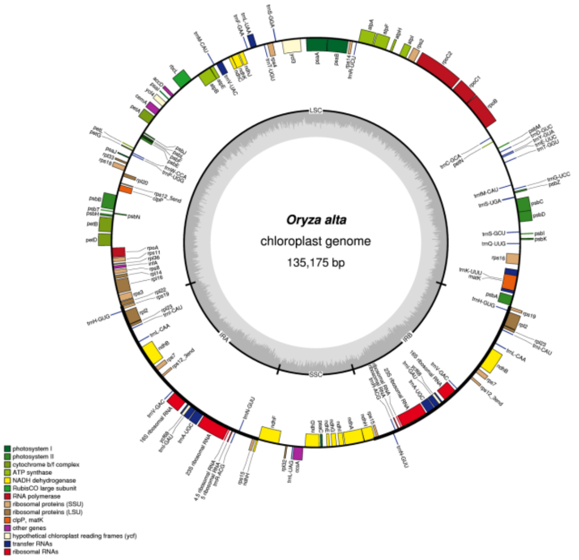
Oryza alta
chloroplast genome
135,175 bp
LSC
IRA
SSC
IRB
photosystem I
photosystem II
cytochrome b/f complex
ATP synthase
NADH dehydrogenase
RubisCO large subunit
RNA polymerase
ribosomal proteins (SSU)
ribosomal proteins (LSU)
clpP, matK
other genes
hypothetical chloroplast reading frames (ycf)
transfer RNAs
ribosomal RNAs

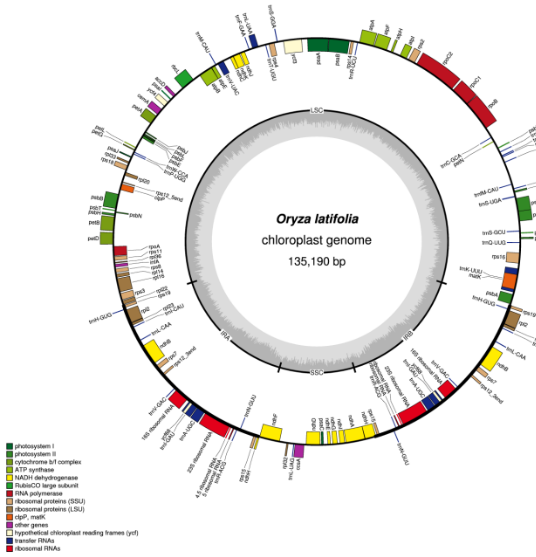
Oryza latifolia
chloroplast genome
135,190 bp
LSC
IRA
SSC
IRB
photosystem I
photosystem II
cytochrome b/f complex
ATP synthase
NADH dehydrogenase
RubisCO large subunit
RNA polymerase
ribosomal proteins (SSU)
ribosomal proteins (LSU)
clpP, matK
other genes
hypothetical chloroplast reading frames (ycf)
transfer RNAs
ribosomal RNAs

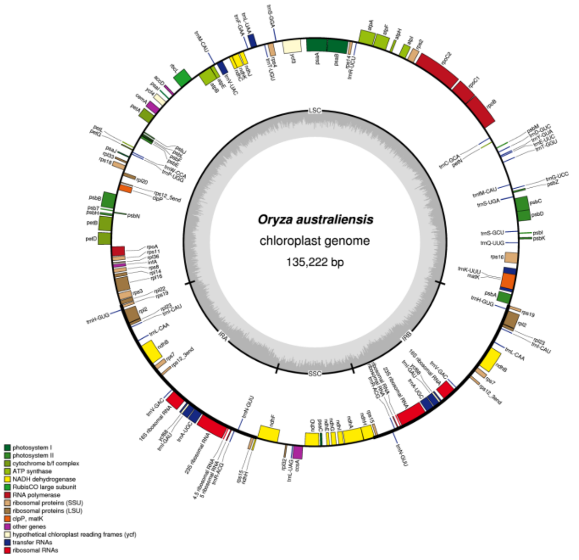
Oryza australiensis
chloroplast genome
135,222 bp
LSC
IRA
SSC
IRB
photosystem I
photosystem II
cytochrome b/f complex
ATP synthase
NADH dehydrogenase
RubisCO large subunit
RNA polymerase
ribosomal proteins (SSU)
ribosomal proteins (LSU)
clpP, matK
other genes
hypothetical chloroplast reading frames (ycf)
transfer RNAs
ribosomal RNAs

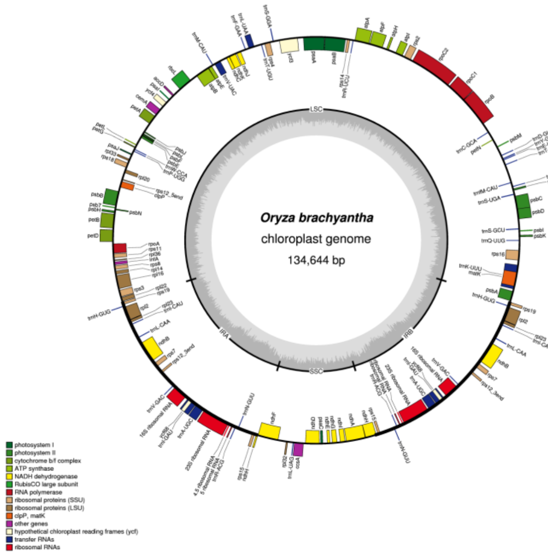
Oryza brachyantha
chloroplast genome
134,644 bp
LSC
IRA
SSC
IRB
photosystem I
photosystem II
cytochrome b/f complex
ATP synthase
NADH dehydrogenase
RubisCO large subunit
RNA polymerase
ribosomal proteins (SSU)
ribosomal proteins (LSU)
clpP, matK
other genes
hypothetical chloroplast reading frames (ycf)
transfer RNAs
ribosomal RNAs

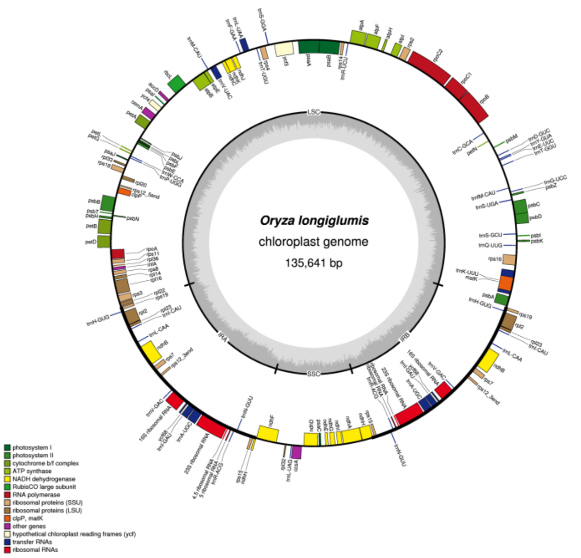
Oryza longiglumis
chloroplast genome
135,641 bp
photosystem I
photosystem II
cytochrome b/f complex
ATP synthase
NADH dehydrogenase
RubisCO large subunit
RNA polymerase
ribosomal proteins (SSU)
ribosomal proteins (LSU)
clpP, matK
other genes
hypothetical chloroplast reading frames (ycf)
transfer RNAs
ribosomal RNAs

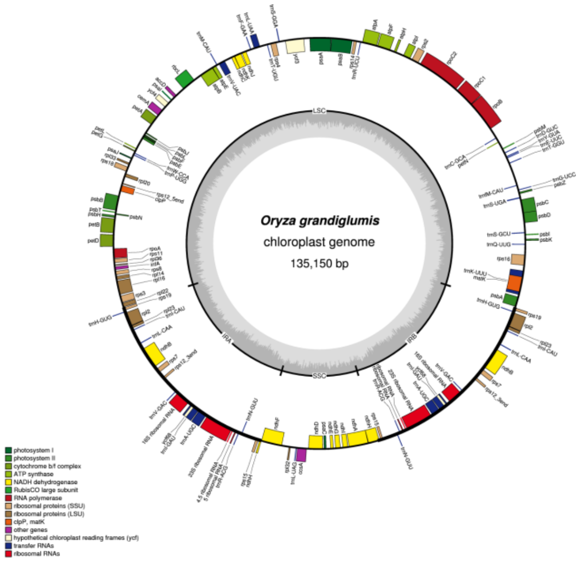
Oryza grandiglumis
chloroplast genome
135,150 bp
photosystem I
photosystem II
cytochrome b/f complex
ATP synthase
NADH dehydrogenase
RubisCO large subunit
RNA polymerase
ribosomal proteins (SSU)
ribosomal proteins (LSU)
clpP, matK
other genes
hypothetical chloroplast reading frames (ycf)
transfer RNAs
ribosomal RNAs

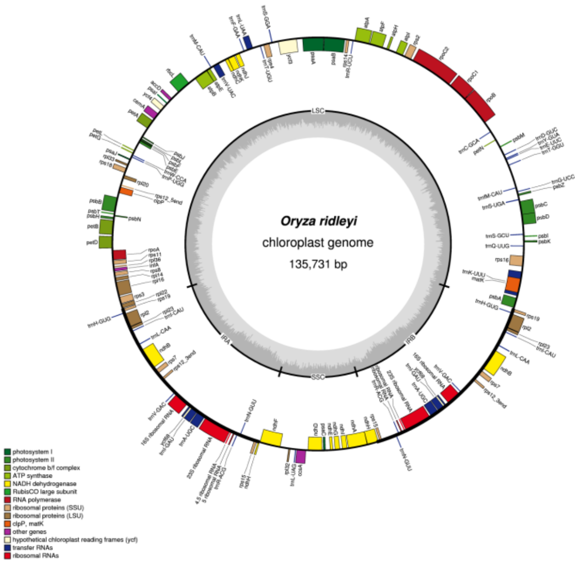
Oryza ridleyi
chloroplast genome
135,731 bp
photosystem I
photosystem II
cytochrome b/f complex
ATP synthase
NADH dehydrogenase
RubisCO large subunit
RNA polymerase
ribosomal proteins (SSU)
ribosomal proteins (LSU)
clpP, matK
other genes
hypothetical chloroplast reading frames (ycf)
transfer RNAs
ribosomal RNAs

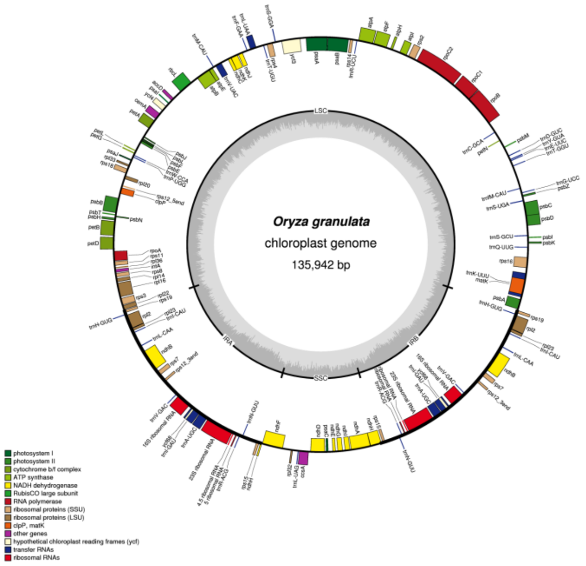
Oryza granulata
chloroplast genome
135,942 bp
photosystem I
photosystem II
cytochrome b/f complex
ATP synthase
NADH dehydrogenase
RubisCO large subunit
RNA polymerase
ribosomal proteins (SSU)
ribosomal proteins (LSU)
clpP, matK
other genes
hypothetical chloroplast reading frames (ycf)
transfer RNAs
ribosomal RNAs

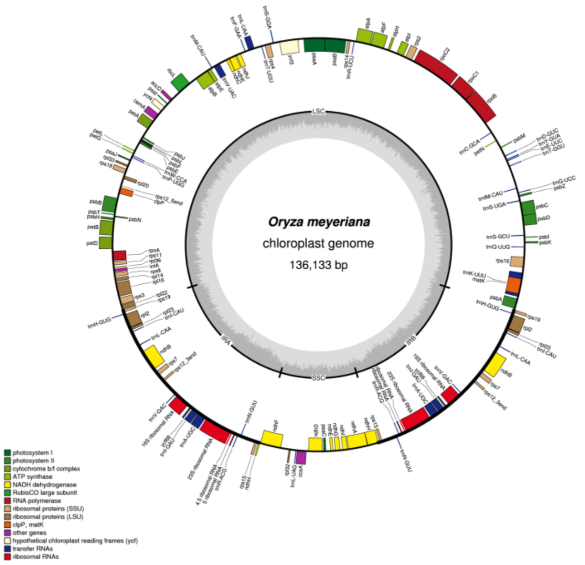
Oryza meyeriana
chloroplast genome
136,133 bp
photosystem I
photosystem II
cytochrome b/f complex
ATP synthase
NADH dehydrogenase
RubisCO large subunit
RNA polymerase
ribosomal proteins (SSU)
ribosomal proteins (LSU)
clpP, matK
other genes
hypothetical chloroplast reading frames (ycf)
transfer RNAs
ribosomal RNAs

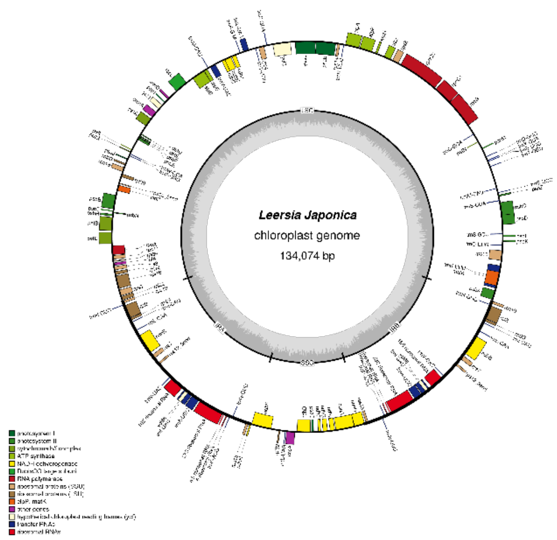
Leersia Japonica
chloroplast genome
134,074 bp

Supplementary Figure 1. Gene maps of the twenty-two *Oryza* species and *L. japonica* chloroplast genomes. The inner circle shows quadripartite structure of the genome, including the two copies of the inverted repeat (IR_A and IR_B) and the large and small single-copy regions (LSC and SSC). Genes inside the circle are transcribed in a clockwise direction, and genes outside of the circle are transcribed counterclockwise. Genes belonging to different functional groups are marked in different colors. Dashed area in the inner circle indicates GC content of the chloroplast genome.

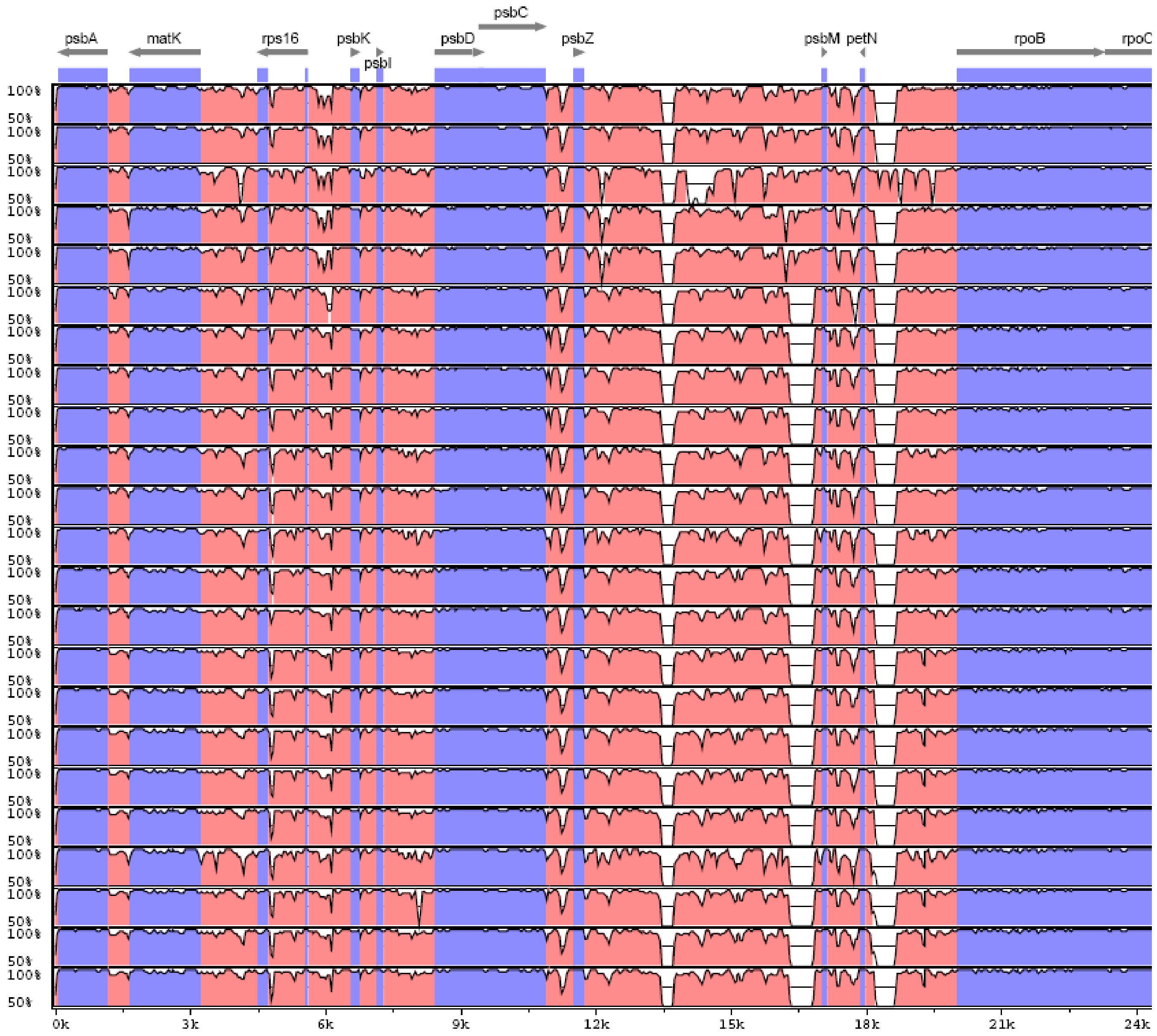
psbA
matK
rps16
psbK
psbI
psbD
psbC
psbZ
psbM
petN
rpoB
rpoC
100%
50%
0k
3k
6k
9k
12k
15k
18k
21k
24k

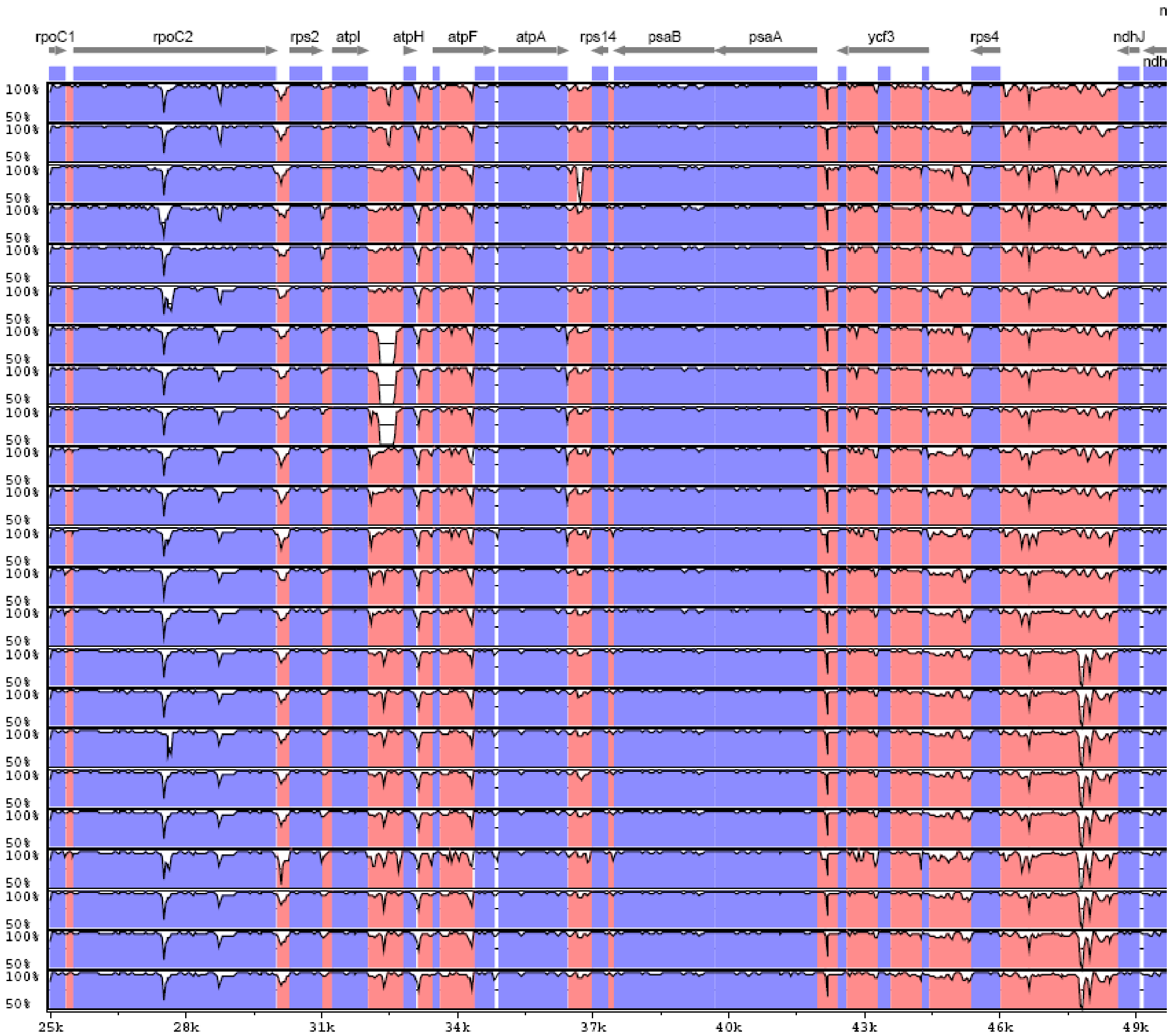

rpoC1
rpoC2
rps2
atpI
atpH
atpF
atpA
rps14
psaB
psaA
ycf3
rps4
ndhJ
ndh
n
100%
50%
25k
28k
31k
34k
37k
40k
43k
46k
49k

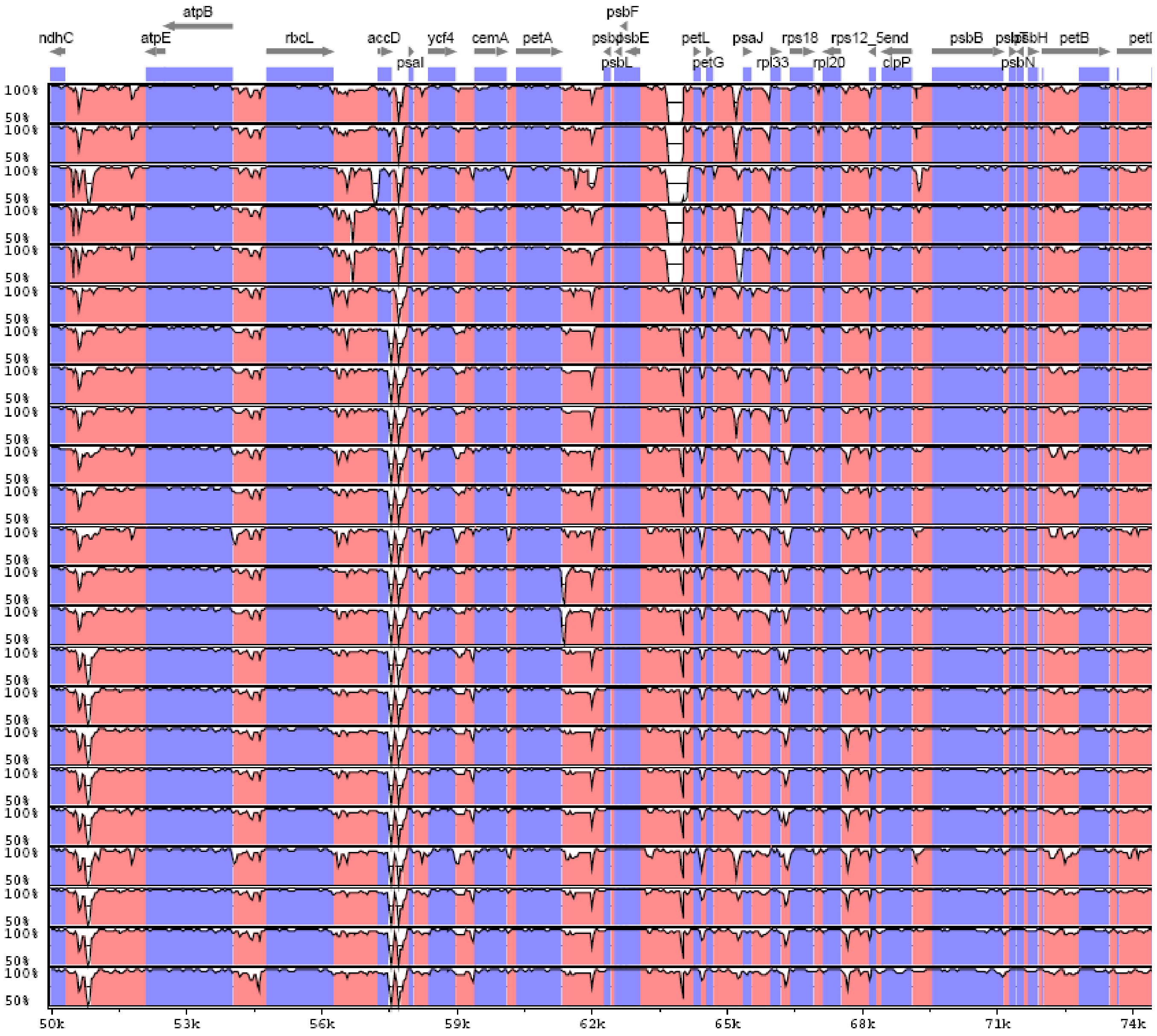

atpB
psbF
ndhC
atpE
rbcL
accD
ycf4
cemA
petA
psbJ
psbE
petL
psaJ
rps18
rps12_5end
psbB
psbH
petB
psaI
psbL
petG
rpl33
rpl20
clpP
psbN
100%
50%
50k
53k
56k
59k
62k
65k
68k
71k
74k

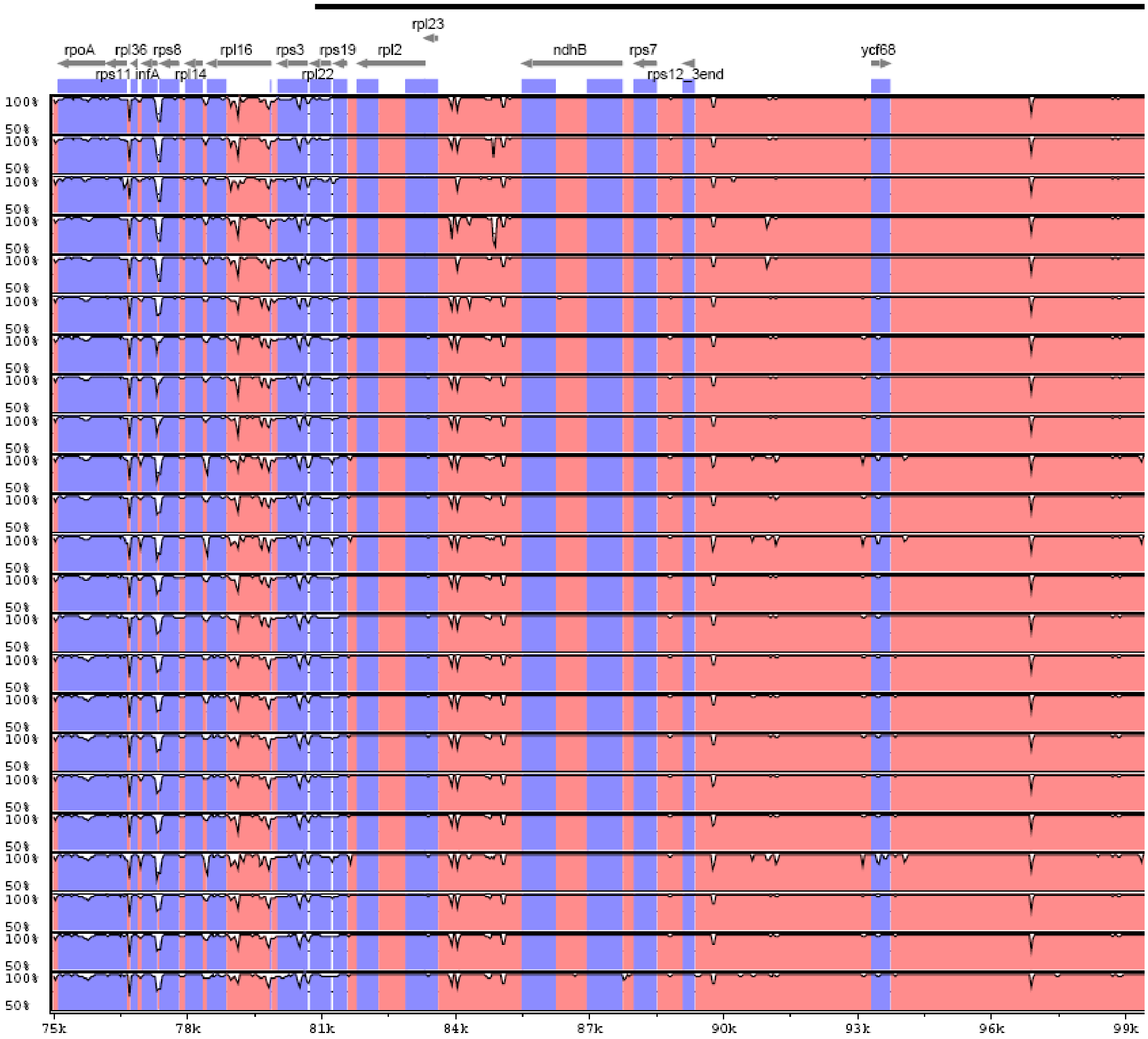

rpl23
rpoA
rpl36
rps8
rpl16
rps3
rps19
rpl2
ndhB
rps7
ycf68
rps11
infA
rpl14
rpl22
rps12_3end
100%
50%
75k
78k
81k
84k
87k
90k
93k
96k
99k

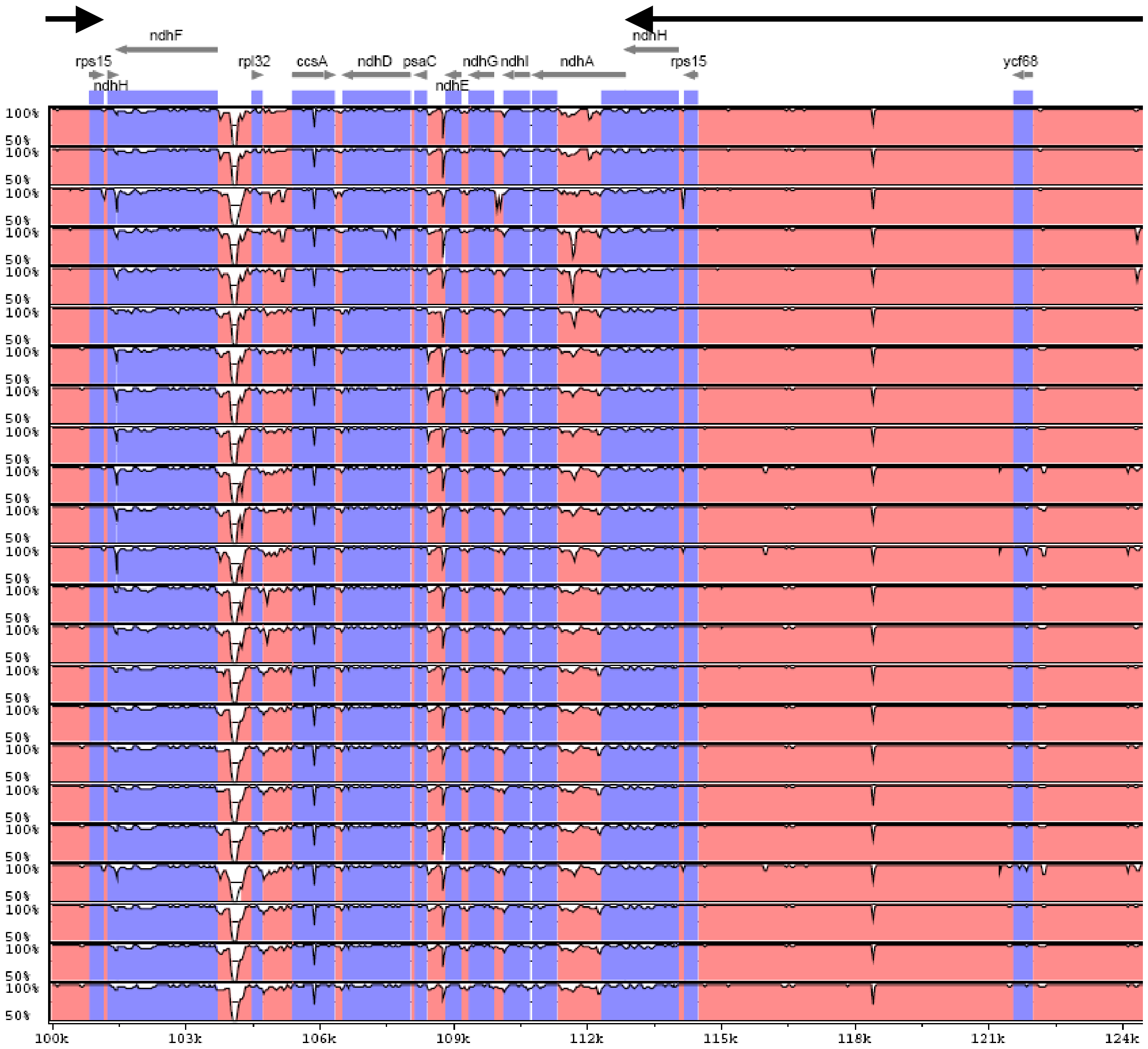

ndhF
ndhH
rps15
rpl32
ccsA
ndhD
psaC
ndhG
ndhI
ndhA
ndhE
ycf68
100%
50%
100k
103k
106k
109k
112k
115k
118k
121k
124k

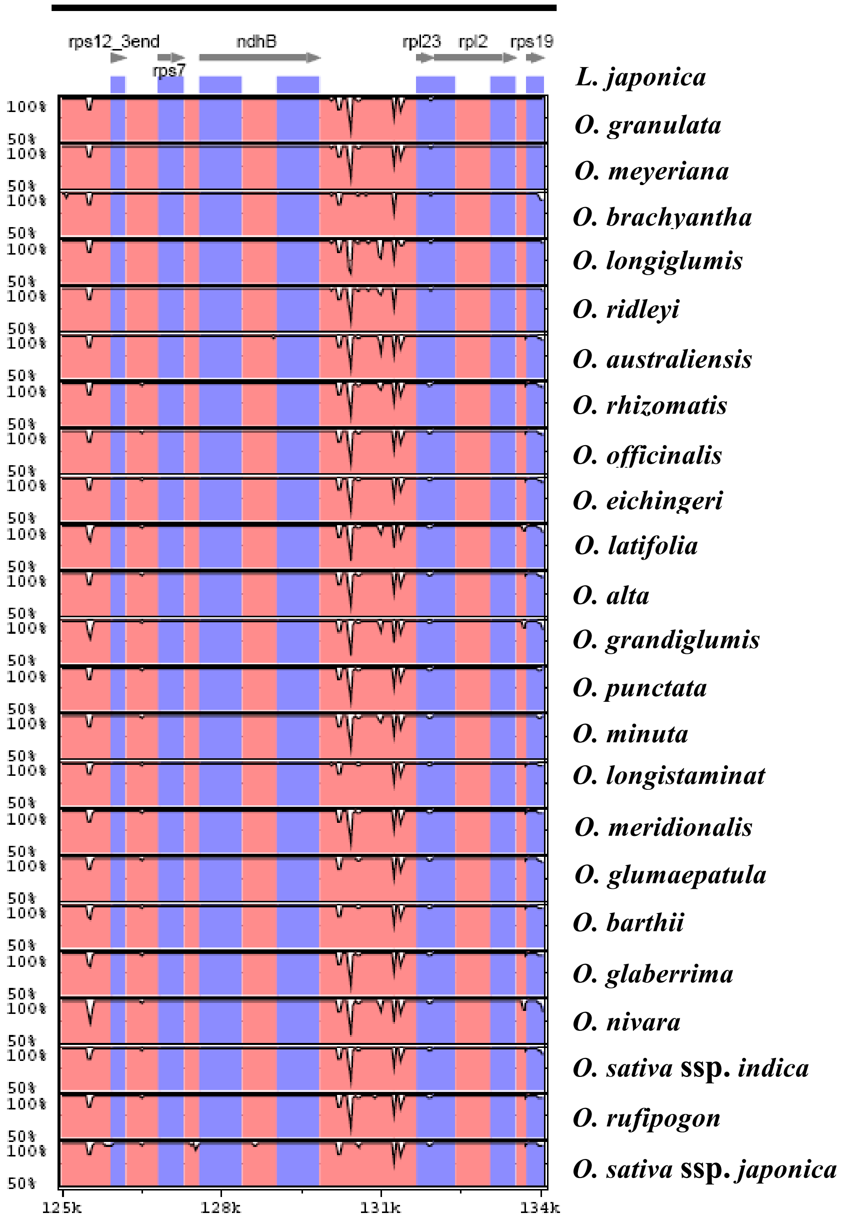

Supplementary Figure 2. Visualization of sequence alignment of the 24 chloroplast genomes. VISTA-based identity plots exhibit sequence identities between the 22 sequenced chloroplast genomes in this study and the two published rice chloroplast genomes (*O. sativa* ssp. *indica* and *O.sativa* ssp. *japonica*), with *L. japonica* as a reference. Genome regions are color coded as protein coding or noncoding sequences, while thick black lines show the inverted repeats (IRs) regions.

Pinus ---------------------------------------MSIKEWFEDKRKITALLKNSV
Cycas ---------------------------------------MSLKEWFEERRKISGSIEDLI
Typha -------------------------------MEKWWFNSMFSNEKLEHRCGLSKSMESLD
Wolffia --
Ceratophyllum --------------MTIHLLYFQSNRGQEGSMEKWWFNLILSNEELEHRCRLSKSMARPR
Piper -------------------MYCIFIQIRKGSMEGRRFDSVLSYGKLEYWRGLSKSMGSFS
Nicotiana --------------MTIHLLYFHANRGQENSMERWWFNSMLFKKEFERRCGLNKSMGSLG
Eleutherococcus --------------MTIHLLYFHTNKGQENSMERWWFNSILFKKEFEHRCGLSKSTGSLG
Coffea --------------MTIHLLYFHANRRQEKSMERWWFNSMLFKKEFECRCGLNKSTESLG
Spinacia MLIRAGNVQIYFHRMTIHQFYFHANRGQESSMKKWWFNSMLSKKELEHGCGLSKSMDSLG
Vitis --------------MTIHLLYFNVNRGQESSMEKWWFNSMLSNEKLEYRCGLSKSTDSPD
Arabidopsis -------------------------------MEKSWFNFMFSKGELEYRGELSKAMDSFA
Amborella -------------------------------MEKCGFNSMLSNEELEHKCELSKSMDSLG
O. punctata --
O. nivara --
O. longistaminata --
O. rhizomatis --
Potamophila --
O. australiensis --
L. japonica --
O. brachyantha --
O. longiglumis --

Pinus ---ERDSKD--ANETE--
Cycas ---ERTSKDYIVNEMD--
Typha -SIGHTSGSEEPILNDTKKKIPGWSDSV--SYSFNN---VDYLFDIRDMWSFISDDT---
Wolffia ----------------MDKNVSSWNESG--SCNFSN---VDQFLDIIDIWSFISDDT---
Ceratophyllum -PIGNTNGSQDPSINDRDKNG---SDSG--NYSFSN---LDHLFDVKDNLSFIYDDT---
Piper SPIGNISGSGDSTINDRDKTIENWSDRS--SYSCPN---VDYLFGVMDILGLVSDDT---
Nicotiana -PIENTNE--DP--NRKVKNIHSWRNRD--NSSCSN---VDYLFGVKDIRNFISDDT---
Eleutherococcus -PIENTSESEDPNINDMKKNIHSWGGRD--NSNYSN---VDHLFGVKDIRNFISDET---
Coffea -SIENASEGDEPDIKDRTKNIQSWGGRD--DSSYSK---VDHLFVVKDIRNFISDDT---
Spinacia -PIENTSTKEDPSLNDPEKQIHSSRNSE--SSSYSN---VNHLVRGIDIQNFISDDT---
Vitis -PIENTSGSEDRVINNTDKNINNWSQIK--SSSYSN---VDHLFGIRDIRNFISDDT---
Arabidopsis -PGEKTTISQDRFIYDMDKNFYGWDERSSYSSSYSNN--VDLLVSSKDIRNFISDDT---
Amborella -LIGNTGAGGGPTLIFDVRDILSLIYDDTFKLKDRNGDSYSVYFDIDNKIFEIDDDSDSL
O. punctata --
O. nivara --
O. longistaminata --
O. rhizomatis --
Potamophila --
O. australiensis --
L. japonica --
O. brachyantha --

```
O.longiglumis        ------------------------------------------------------------

Pinus                -----KNKNKS-------------------------------------------------
Cycas                -----KNKNIK-------------------------------------------------
Typha                --FLVKDKNGDSYSVY--------FDMENLSFEIDNDSSFLSELGSFFS-----TFLNR-
Wolffia              --FLVRDSNGDSFSIY--------FDSENQIFEIDNESTFITELERSFSSSPNSSYMNS-
Ceratophyllum        --FLVRDSNGDSYSIY--------FDIENLIFEIDNDSFFLSKLESSFS-----NYLNS-
Piper                --FFVRDGSGDTYSIY--------FDVESQIFEIDNDSSFLSELESSFS-----SYQNSR
Nicotiana            --FLVSDRNGDSYSIY--------FDIENHIFEIDNDHSFLSELESSFYSYRNSNYRNNG
Eleutherococcus      --FLVRDSNGNSYSIY--------FDIENHIFEIDNGHSFQSELESSFYSYRNSSYLNNG
Coffea               --FLVKDSNGDNFSIY--------FDIENQIFEIDNNHSFRSELKN---TYWNSSYLNNG
Spinacia             --FLLIDTKGDSYSIY--------FDIENQIFEIDNDHSFLSKLQSSFSNYWNSSYLNSR
Vitis                --FLVRDRNGVSYFIY--------FDIENQIFEIDNDQSFLSELESFFYSYRNSSYLNNV
Arabidopsis          --FFVRDSNKNSYSIF--------FDKKKKIFEIDND---FSDLEKFFYSYCSSSYLNNR
Amborella            SGLDIENQNQIFFDIYNDSDSLSELDSENKNFEIHNESDFMSKLESSFFSYLTYSCLKRR
O.punctata           ------------------------------------------------------------
O.nivara             ------------------------------------------------------------
O.longistaminata     ------------------------------------------------------------
O.rhizomatis         ------------------------------------------------------------
Potamophila          ------------------------------------------------------------
O.australiensis      ------------------------------------------------------------
L.japonica           ------------------------------------------------------------
O.brachyantha        ------------------------------------------------------------
O.longiglumis        ------------------------------------------------------------

Pinus                ------------------------------------------------------------
Cycas                ------------------------------------------------------------
Typha                -----------------GSKKNNHYYYHYMYDTQSRWNN--HINSCIDSYLRFEVSINSS
Wolffia              -----------------GSKNTSRYSYRYMYDTQSSWNN--HINSCIDSYLRYEISSESY
Ceratophyllum        -----------------GSKNYNRYYDSYMYDTKYSWNN--HINSYIDSYLCSEIRIDSY
Piper                F----------------GPKDDNRDYYCYMYDTKYSWNT--HINSCIDSYLRSEISIDSY
Nicotiana            ------------------FRGEDPYYNSYMYDTQYSWNN--HINSCIDSYLQSQICIDTS
Eleutherococcus      ------------------SNSDDPHYDHYMYDTQYSWNN--HINSCIDNYLQSQIFIETD
Coffea               ------------------STSEDTYYNHYMYDTQYSWNN--HINSCIDSYLQSQIWIETS
Spinacia             ------------------SKNGDTYNGHSLYYTNDSWNN--HINNCIDSYLHSQIRSDSS
Vitis                NSSYLN----------NVSKSNHPHYDRYMYDTKYSWNK--HINSCINNY----------
Arabidopsis          S-----------------KGDNDLHYDPYIKDTKYNCTN--HINSCIDSYFRSYICIDNN
Amborella            SSFSSYPTSLTSSNLNSGSKSYNPYYDPYMHDTRYSWNNDININSCIDSYIRCEID---S
O.punctata           ------------------------------------------------------------
O.nivara             ------------------------------------------------------------
O.longistaminata     ------------------------------------------------------------
O.rhizomatis         ------------------------------------------------------------
Potamophila          ------------------------------------------------------------
```

O. australiensis	--
L. japonica	--
O. brachyantha	--
O. longiglumis	--

Pinus	--
Cycas	--
Typha	ILGGTDNSSDCYIYNFICT-ENVSGSESGSSSIRSSSIRTSK---NGSKFSIRGRSND--
Wolffia	ISGETHNYSDNYVYNFISN-ESISVSESSNS-----TIRTSI---NASDFNIRGRSND--
Ceratophyllum	ISSGIYNYSENYIYSYVWNGENVSTIKSRSS-----SIRTSA---NSSDINLKGRYND--
Piper	ISSSSDYYNDSYIYTFIFS-ESVNDSESGSS-----GIKTIT---NDHDSNIREKSNDND
Nicotiana	IISGSENYGDSYIYRAVCGGESRNSSENEGS-----SRRTRT---KGSDLTIRESSND--
Eleutherococcus	IVSGSDNYSNSYIYSSICG-------EIEGS-----GIRTST---NGSDLTIRESSND--
Coffea	IVSGGDNYSDSYIYSSICG-ESRNNSEGEVS-----DIQTHV---KGSDFTIIESSND--
Spinacia	ILSGNDSYILSYIFNESGN-------RSESF-----SKRSIT---NGSNLTRRESSHN--
Vitis	ILSGSNNYSDSYIYSYICG-QSRTRSEHGSS-----SKQTST---NGSDLK---SSNV--
Arabidopsis	FLIDSNNFNESYIYNFICS-ESGKIRESKNY-----KIRTNR---NRSNLI---SSKD--
Amborella	ISSGSDNCSDSYIYSYICS-EGVSYSDNGSS-----SIRTRTSTSSGSSYHIRGRSNN--
O. punctata	--
O. nivara	--
O. longistaminata	--
O. rhizomatis	--
Potamophila	--
O. australiensis	--
L. japonica	--
O. brachyantha	--
O. longiglumis	--

Pinus	IDYAK-IKKLWAQCDNCENLLYLRFLRENQSVCKECGYYLQMNSSDRIELPIDRDTWRPM
Cycas	IDFN---NRLWVQCDNRENLLYMKYLRQNKSVCEECGYHLQMSSSDRIELSIDHGTWHPM
Typha	LDINKKYRHLWVQCENCYGLNYKKFFRLKMNICEQCGYHLKMSSSDRIGVLIDPGTWDPM
Wolffia	LDINKKYRHLWVQCENCYGLNYKKFFRSRLNICEHCGYHLKMSSSERIELLIDPGTWDPM
Ceratophyllum	FDINIKYRHLWVQCDNCYGLNYKKIFSSKMNICEQCGYHLKMSSSERIELSIDSGTWDPM
Piper	FDINKKYRHLWVQCENCYGLNYKKFFQSKMNICEQCGYHLKMSSSDRIELSIDPGTWDPM
Nicotiana	LEVTQKYRHLWVQCENCYGLNYKKFLKSKMNICEQCGYHLKMSSSDRIELLIDPGTWDPM
Eleutherococcus	LDVTQKYRHLWVQCENCYGLNYKKFFKSKMNLCEQCGYHLKMSSSERIELLIDPGTWDPM
Coffea	LDVTQKYRHLWVQCENCYGLNYKKFLKSKMNICEQCGYHLKMNSSERIELSIDPGTWDPM
Spinacia	LDVTQKYRHLWVQCESCYALNYKKLLKSKMGICEQCGYHLKISSSDRIELLIDPGTWNPM
Vitis	LYVAQKYRHLWIQCENCYGLNYKKNLKSKINICEQCGYHLKMSSSDRIELSIDPGTWDPV
Arabidopsis	FDITQNYNQLWIQCDNCYGLMYK---KVKMNVCEQCGHYLKMSSSERIELSIDPGTWNPM
Amborella	FEKNKKLKKLWVQCENCYALNYNKLFRSKMNVCEQCGYHLKMSSSDRIELSIDPGTWHPM
O. punctata	--
O. nivara	--

O. longistaminata	--
O. rhizomatis	--
Potamophila	--
O. australiensis	--
L. japonica	--
O. brachyantha	--
O. longiglumis	--

Pinus	DEDMYTLDVLQFYSENEPSHSDNLNSEDESYKDHITFYQIETGLTDAIQTGIGQLNGLTI
Cycas	DEDMAAPDPIQFHFEDEP------------HIDRITSCQIRTGLTDAVQTGIGRPNGIPI
Typha	DEDMVSMDPIEFHSEEEP------------YRDRIDFYQRKTGLTEAVQTGIGQLNGISI
Wolffia	DENMVSTDPIEFHSEEEP------------YRDRIDSYQKKTGLTDAVQTGIGQLNGIPI
Ceratophyllum	NEDMVSTDPIEFHSEEEP------------YRDRIDSYQIKTGLTEAVQTGIGQLNGMPI
Piper	DEDMVSIDPIEFHSEEEP------------YKDRLDSYQRKTGLTEAVQTGVGQLNGIPV
Nicotiana	DEDMVSLDPIEFHSEEEP------------YKDRIDSYQRKTGLTEAVQTGIGQLNGIPV
Eleutherococcus	DEDMVSLDPIEFHSEEEP------------YKDRIDSYQRKTGLTEAVQTGIGQLNSIPV
Coffea	DEDMASLDPIEFHSEEEP------------YKDRIDSYQKKTGLTEAVQTGIGQLNGIPV
Spinacia	DDDMVSMDPIGFHSEEEA------------YKDRIDSYQIKTGLTEAVQTGIGQLNGIPV
Vitis	DEDMVSLDPIEFHSGEEP------------YKERIDFYQRKTGLTEAVQTGTGQLNGIPV
Arabidopsis	DEDMVSADPIKFHSKEEP------------YKNRIDSAQKTTGLTDAVQTGTGQLNGIPV
Amborella	DEDMVSTDPIEFHSEEEP------------YRDRIDSYQRQAGLTDAVQTGIGQLEGIPI
O. punctata	--
O. nivara	--
O. longistaminata	--
O. rhizomatis	--
Potamophila	--
O. australiensis	--
L. japonica	--
O. brachyantha	--
O. longiglumis	--

Pinus	ALGVMDFQFMGGSMGSVVGEKITRLIERATAESLPLIMVCASGGARMQEGSFSLMQMAKI
Cycas	AIGVMDFQFMGGSMGSVVGEKITRLIEYATKQFLPVIMVCASGGARMQEGSFSLMQMAKI
Typha	AIGVMDFQFMGGSMGSVVGEKITRLIEYATNRSLPVIIVCASGGARMQEGSLSLMQMAKI
Wolffia	AIGVMDFQFMGGSMGSVVGEKITRLIEYATNKSLPVIIVCASGGARMQEGSLSLMQMAKI
Ceratophyllum	AIGVMDFQFMGGSMGSVVGEKITRLIEYATNRSLPVIIVCASGGARMQEGSLSLMQMAKI
Piper	AIGVMDFQFMGGSMGSVVGEKITRLIEYATNRSLPVIMVCASGGARMQEGSLSLMQMAKI
Nicotiana	AIGVMDFQFMGGSMGSVVGEKITRLIEYAANQILPLIIVCASGGARMQEGSLSLMQMAKI
Eleutherococcus	AIGVMDFQFMGGSMGSVVGEKITRLIEYATKKFLPLIIVCASGGARMQEGSLSLMQMAKI
Coffea	AVGVMDFQFMGGSMGSVVGEKITRLIEYATKELLPLIIVCASGGARMQEGSLSLMQMAKI
Spinacia	AIGVMDFQFMGGSMGSVVGEKITRLIEYASNKFIPLIIVCASGGARMQEGSLSLMQMAKI
Vitis	AIGVMDFQFMGGSMGSVVGEKITRLIEYATNEFLPLILVCSSGGARMQEGSLSLMQMAKI
Arabidopsis	ALGVMDFRFMGGSMGSVVGEKITRLIEYATNQCLPLILVCSSGGARMQEGSLSLMQMAKI

```
Amborella            AIGVMDFQFMGGSMGSVVGEKITRLIEYAIDRSLPVVIVCASGGARMQEGGLSLMQMAKI
O. punctata          ----MALQSLRGSMRSVVGKRICPLIEYAIFPPLPRIIVYASRRARMQRGNYSLIKKPKK
O. nivara            ----MALQSLRGSMRSVVGKRICPLIEYAIFPPLPRIIVYASRRARMQRGNYSLIKKPKK
O. longistaminata    ----MALQSLRGSMRSVVGKRICPLIEYAIFPPLPRIIVYASRRARMQRGNYSLIKKPKK
O. rhizomatis        ----MALQSLRGSMRSVVGKRICPLIEYAIFPPLPHIIVYASRRARMQRGNYSLIKKPKK
Potamophila          ----MALQSLRGSMRSVVGKRICPLIEYAIFPPLPRIIVCASRGARMQGGNYSLIKKPKK
O. australiensis     ----MALQSLRGSMRSVVDKRICPLIEYAIFPPLPRIIVYASRRARMQRGNYSLIKKPKK
L. japonica          ----MALQSLRRSMRSLVGKRICPLIEYAIFPPLPCIIICASRGTRMQRGNYSLIKKPKK
O. brachyantha       ----MALQSLRGSMRSVVGKRICPLIEYAIFPPLPRIIVCASRGARMQRGNYSLIKSLKS
O. longiglumis       ----MALQSLRGSMRSVVGKRICPLIEYAIFPPLPRIIVCASRGARMQRGNYSLIKKPKK
                         * :: :  ** *:*.::*  *** *    :* ::: :*  :*** *. **::  *

Pinus                ASALYIHQKEKKLLYISILTSPTTGGVTASFGMLGDIIIAEPKAYIAFAGKRVIEQTLGQ
Cycas                STALYIHQLEKKLLYVSILTYPTTGGVTASFGMLGDIIIAEPKAYIAFAGRRVIEQTSGQ
Typha                SSALYNYQSTKKLFYVSILTSPTTGGVTASFGMLGDVIIAEPNAYIAFAGKRVIEQTLNK
Wolffia              SSALYNYQLNKKLFYVPILTSPTTGGVTASFGMLGDIIIAEPNAYIAFAGKRVIEQTLNK
Ceratophyllum        SSALYNYQLNKKLFYVSILTSPTTGGVTASFGMLGDIIIAEPNAYIAFAGKRVIEQTLNK
Piper                SSVSYDYQSNQKLFYVSILTSPTTGGVTASFGMLGDIIIAEPNAYIAFAGKRVIEQTLNK
Nicotiana            SSALYDYQLNKKLFYVSILTSPTTGGVTASFGMLGDIIIAEPNAYIAFAGKRVIEQTLNK
Eleutherococcus      SSALYDYQSKKKLFYVPILTSPTTGGVTASFGMLGDIIIAEPNAYIAFAGKRVIEQTLNK
Coffea               SSALYDYQSNKKLFYVSILTSPTTGGVTASFGMLGDIIIAEPNAYIAFAGKRVIEQTLNT
Spinacia             SSVLYDYQSNKKLFYVSILTSPTTGGVTASFGMLGDIIIAEPNAYIAFAGKRVIEQTLNK
Vitis                SSALYDYQSNKKLFYVSILTSPTTGGVTASFGMLGDIIIAEPNSYIAFAGKRVIEQTLKK
Arabidopsis          SSVLCDYQSSKKLFYISILTSPTTGGVTASFGMLGDIIIAEPYAYIAFAGKRVIEQTLKK
Amborella            SSASYNYQSNKKLFYVSILTSPTTGGVTASFGMLGDIIIAEPNAYVAFAGKRVIEETLNK
O. punctata          VSTLRQYQSTKSPMYQSFQRICGVR------EWLNKYCMWKEVDEKDFG-----------
O. nivara            VSTLRQYQSTKSPMYQCLQRICGVR------EWLNKYCMWKEVDEKDFG-----------
O. longistaminata    VSTLRQYQSTKSPMYQSLQRICGVR------EWLNKYCMWKEVDEKDFG-----------
O. rhizomatis        VSTLRQYQSTKSPMYQSLQRICGVR------EWLNKYGMWKEVDEKDFG-----------
Potamophila          VSTLWQYQSTKSPMYQSLQRVCGVR------EWLNKYCMWKEVDENDFG-----------
O. australiensis     VSTLRQYQSTKSPMYQSLQRIG--------------------------------------
L. japonica          VSTLWQYQSTKKPCVSILT-TGRIR------VSVC-------------------------
O. brachyantha       FNFT-------AISIN--------------------------------------------
O. longiglumis       FQLYGNINQLKALCINPYSGSGKSF------GMLGDILISEPDMHLAFV-----------


Pinus                KVIEDFQVTEHLFGHGLFDLIVPRNLLKGVLSELFWFYVLR-------------------
Cycas                KVPDGLQVAEHLFDHGSFDLIVPRSLLKGVLSEPFQLYGLIPCEKERTLGLVSCNEQQFS
Typha                TVPDGSQAAEYLFHKGLFDPIVPRNPLKGVLSELFQLHGFF----------------PLN
Wolffia              TVPDGSQAAEYLFQKGLFDLIVPRNLLKGVLAELFQLHGFF----------------PLT
Ceratophyllum        TVPEGSQAAEYSFHKGLFDSIVPRNLLKGALSELLQLHGFF----------------PLN
Piper                TVPEGSQAAEYLFHKGLFDLIVPRNPLKSVLSEFFQLHGFF----------------PLN
Nicotiana            TVPEGSQAAEYLFQKGLFDLIVPRNLLKSVLSELFKLHAFF----------------PLN
Eleutherococcus      TVPEGSQAAEYLFQKGLFDLIVPRNPLKSVLSELFQLHAFF----------------PLN
Coffea               TVPEGSQAAEYLFQKGLFDLIVPRNLLKSVLSELFKFHAFV----------------PLN
```

```
Spinacia             TVPEGSQAAEFLFHKGLFDPIVPRNLLKGVLSELFELHAFF----------------PLN
Vitis                TVPEGSQAAEYLFHKGLFDPIVPRNTLKGVLSELFQLHAFF----------------PLN
Arabidopsis          AVPEGSQAAESLLRKGLLDAIVPRNLLKGVLSELFQLHAFF----------------PLN
Amborella            KIPDGLQVAEYSFHKGLFDSIVPRNPLKGVPSELFQLHGFFSSTF-----------HPLK
O.punctata           ------------FEIGAFD-----------------------------------------
O.nivara             ------------FEIGAFD-----------------------------------------
O.longistaminata     ------------FEIGTFD-----------------------------------------
O.rhizomatis         ------------FEIGAFD-----------------------------------------
Potamophila          ------------FEIGAFD-----------------------------------------
O.australiensis      ------------------------------------------------------------
L.japonica           ------------------------------------------------------------
O.brachyantha        ------------------------------------------------------------
O.longiglumis        ------------GSESG-------------------------------------------


Pinus                SSL-------------------------------
Cycas                SSAPRNESDNDTVPSHSENRKNQLQRIVPHLSFF
Typha                QNSKNESIAPD-----------------------
Wolffia              ----------------------------------
Ceratophyllum        HNSQVKR---------------------------
Piper                SNSKH-----------------------------
Nicotiana            QKSSKIK---------------------------
Eleutherococcus      QNSIKEH---------------------------
Coffea               QNETEH----------------------------
Spinacia             QNKGED----------------------------
Vitis                PKKIK-----------------------------
Arabidopsis          TN--------------------------------
Amborella            SNKVKR----------------------------
O.punctata           ----------------------------------
O.nivara             ----------------------------------
O.longistaminata     ----------------------------------
O.rhizomatis         ----------------------------------
Potamophila          ----------------------------------
O.australiensis      ----------------------------------
L.japonica           ----------------------------------
O.brachyantha        ----------------------------------
O.longiglumis        ----------------------------------
```

Supplementary Figure 3. Alignment of amino acid sequences of *AccD* of Oryzeae compared with other plants.

O. punctata	ATGGCGTACTCCTCCTGTTTGAATCGGAGTTTGAAACCAAACAAACTTCTCCTCAGGAGG
O. rhizomatis	ATGGCGTACTCCTCCTGTTTGAATCGGAGTTTGAAACCAAACAAACTTCTCCTCAGGAGG
O. officinalis	ATGGCGTACTCCTCCTGTTTGAATCGGAGTTTGAAACCAAACAAACTTCTCCTCAGGAGG
O. minuta	ATGGCGTACTCCTCCTGTTTGAATCGGAGTTTGAAACCAAACAAACTTCTCCTCAGGAGG
O. meridionalis	ATGGCGTACTCCTCCTGTTTGAATCGGAGTTTGAAACCAAACAAACTTCTCCTCAGGAGG
O. longistaminata	ATGGCGTACTCCTCCTGTTTGAATCGGAGTTTGAAACCAAACAAACTTCTCCTCAGGAGG
O. latifolia	ATGGCGTACTCCTCCTGTTTGAATCGGAGTTTGAAACCAAACAAACTTCTCCTCAGGAGG
O. grandiglumis	ATGGCGTACTCCTCCTGTTTGAATCGGAGTTTGAAACCAAACAAACTTCTCCTCAGGAGG
O. eichingeri	ATGGCGTACTCCTCCTGTTTGAATCGGAGTTTGAAACCAAACAAACTTCTCCTCAGGAGG
O. alta	ATGGCGTACTCCTCCTGTTTGAATCGGAGTTTGAAACCAAACAAACTTCTCCTCAGGAGG
O. barthii	ATGGCGTACTCCTCCTGTTTGAATCGGAGTTTGAAACCAAACAAACTTCTCCTCAGGAGG
O. glaberrima	ATGGCGTACTCCTCCTGTTTGAATCGGAGTTTGAAACCAAACAAACTTCTCCTCAGGAGG
O. glumaepatula	ATGGCGTACTCCTCCTGTTTGAATCGGAGTTTGAAACCAAACAAACTTCTCCTCAGGAGG
O. sativa ssp *indica*	ATGGCGTACTCCTCCTGTTTGAATCGGAGTTTGAAACCAAACAAACTTCTCCTCAGGAGG
O. sativa ssp *japonica*	ATGGCGTACTCCTCCTGTTTGAATCGGAGTTTGAAACCAAACAAACTTCTCCTCAGGAGG
O. nivara	ATGGCGTACTCCTCCTGTTTGAATCGGAGTTTGAAACCAAACAAACTTCTCCTCAGGAGG
O. rufipogon	ATGGCGTACTCCTCCTGTTTGAATCGGAGTTTGAAACCAAACAAACTTCTCCTCAGGAGG
O. australiensis	ATGGCGTACTCCTCCTGTTTGAATCGGAGTTTGAAACCAAACAAACTTCTCCTCAGGAGG
O. brachyantha	ATGGCGTACTCCTCCTGTTTGAATCGGAGTTTGAAACCAAACAAACTTCTCCTCAGGAGG
O. longiglumis	ATGGCGTACTCCTCCTGTTTGAATCGGAGTTTGAAACCAAACAAACTTATCCTCAGGAGG
O. ridleyi	ATGGCGTACTCCTCCTGTTTGAATCGGAGTTTGAAACCAAACAAACTTATCCTCAGGAGG
O. granulata	ATGGCGTACTCCTCCTGTTTGAATCGGAGTTTGAAACCAAACAAACTTCTCCTCAGGAGG

O. meyeriana ATGGCGTACTCCTCCTGTTTGAATCGGAGTTTGAAACCAAACAAACTTCTCCTCAGGAGG

L. japonica ATGGCGTACTCCTCCTGTTTGAATCGGAGTTTGAAACCAAACAAACTTCTCCTCAGGAGG

** ***********

O. punctata ATAGATGGGGCGATTCAGGTGAGATCCCATGTAGATCTAACTTTCTATTCACTCGTGGGA
O. rhizomatis ATAGATGGGGCGATTCAGGTGAGATCCCATGTAGATCTAACTTTCTATTCACTCGTGGGA
O. officinalis ATAGATGGGGCGATTCAGGTGAGATCCCATGTAGATCTAACTTTCTATTCACTCGTGGGA
O. minuta ATAGATGGGGCGATTCAGGTGAGATCCCATGTAGATCTAACTTTCTATTCACTCGTGGGA
O. meridionalis ATAGATGGGGCGATTCAGGTGAGATCCCATGTAGATCTAACTTTCTATTCACTCGTGGGA
O. longistaminata ATAGATGGGGCGATTCAGGTGAGATCCCATGTAGATCTAACTTTCTATTCACTCGTGGGA
O. latifolia ATAGATGGGGCGATTCAGGTGAGATCCCATGTAGATCTAACTTTCTATTCACTCGTGGGA
O. grandiglumis ATAGATGGGGCGATTCAGGTGAGATCCCATGTAGATCTAACTTTCTATTCACTCGTGGGA
O. eichingeri ATAGATGGGGCGATTCAGGTGAGATCCCATGTAGATCTAACTTTCTATTCACTCGTGGGA
O. alta ATAGATGGGGCGATTCAGGTGAGATCCCATGTAGATCTAACTTTCTATTCACTCGTGGGA
O. barthii ATAGATGGGGCGATTCAGGTGAGATCCCATGTAGATCTAACTTTCTATTCACTCGTGGGA
O. glaberrima ATAGATGGGGCGATTCAGGTGAGATCCCATGTAGATCTAACTTTCTATTCACTCGTGGGA
O. glumaepatula ATAGATGGGGCGATTCAGGTGAGATCCCATGTAGATCTAACTTTCTATTCACTCGTGGGA
O. sativa ssp *indica* ATAGATGGGGCGATTCAGGTGAGATCCCATGTAGATCTAACTTTCTATTCACTCGTGGGA
O. sativa ssp *japonica* ATAGATGGGGCGATTCAGGTGAGATCCCATGTAGATCTAACTTTCTATTCACTCGTGGGA
O. nivara ATAGATGGGGCGATTCAGGTGAGATCCCATGTAGATCTAACTTTCTATTCACTCGTGGGA
O. rufipogon ATAGATGGGGCGATTCAGGTGAGATCCCATGTAGATCTAACTTTCTATTCACTCGTGGGA
O. australiensis ATAGATGGGGCGATTCAGGTGAGATCCCATGTAGATCTAACTTTCTATTCACTCGTGGGA
O. brachyantha ATAGATGGGGCGATTCAGGTGAGATCCCATGTAGATCTAACTTTCTATTCACTCGTGGGA
O. longiglumis ATAGATGGGGCGATTCAGGTGAGATCCCATGTAGATCTAACTTTCTATTCACTCGTGGGA
O. ridleyi ATAGATGGGGCGATTCAGGTGAGATCCCATGTAGATCTAACTTTCTATTCACTCGTGGGA
O. granulata ATAGATGGGGCGATTCAGGTGAGATCCCATGTAGATCTAACTTTCTATTCACTCGTGGGA
O. meyeriana ATAGATGGGGCGATTCAGGTGAGATCCCATGTAGATCTAACTTTCTATTCACTCGTGGGA
L. japonica ATAGATGGGGCGATTCAGGTGAGATCCCATGTAGATCTAACTTTCTATTCACTCGTGGGA
****

O. punctata TCCGGGCGGTCCGGGGGGGG--CACTACGGCTCCTCTCTTCTCGAGAATCCATACATCCC
O. rhizomatis TCCGGGCGGTCCGGGGGGGG--CACTACGGCTCCTCTCTTCTCGAGAATCCATACATCCC
O. officinalis TCCGGGCGGTCCGGGGGGGG--CACTACGGCTCCTCTCTTCTCGAGAATCCATACATCCC
O. minuta TCCGGGCGGTCCGGGGGGGG--CACTACGGCTCCTCTCTTCTCGAGAATCCATACATCCC
O. meridionalis TCCGGGCGGTCCGGGGGGGG--CACTACGGCTCCTCTCTTCTCGAGAATCCATACATCCC
O. longistaminata TCCGGGCGGTCCGGGGGGGG--CACTACGGCTCCTCTCTTCTCGAGAATCCATACATCCC
O. latifolia TCCGGGCGGTCCGGGGGGGG--CACTACGGCTCCTCTCTTCTCGAGAATCCATACATCCC
O. grandiglumis TCCGGGCGGTCCGGGGGGGG--CACTACGGCTCCTCTCTTCTCGAGAATCCATACATCCC
O. eichingeri TCCGGGCGGTCCGGGGGGGG--CACTACGGCTCCTCTCTTCTCGAGAATCCATACATCCC
O. alta TCCGGGCGGTCCGGGGGGGG--CACTACGGCTCCTCTCTTCTCGAGAATCCATACATCCC
O. barthii TCCGGGCGGTCCGGGGGGGG--CACTACGGCTCCTCTCTTCTCGAGAATCCATACATCCC
O. glaberrima TCCGGGCGGTCCGGGGGGGG--CACTACGGCTCCTCTCTTCTCGAGAATCCATACATCCC
O. glumaepatula TCCGGGCGGTCCGGGGGGGG--CACTACGGCTCCTCTCTTCTCGAGAATCCATACATCCC

O. sativa ssp *indica* TCCGGGCGGTCCGGGGGGGG--CACTACGGCTCCTCTCTTCTCGAGAATCCATACATCCC
O. sativa ssp *japonica* TCCGGGCGGTCCGGGGGGGG--CACTACGGCTCCTCTCTTCTCGAGAATCCATACATCCC
O. nivara TCCGGGCGGTCCGGGGGGGG--CACTACGGCTCCTCTCTTCTCGAGAATCCATACATCCC
O. rufipogon TCCGGGCGGTCCGGGGGGGG--CACTACGGCTCCTCTCTTCTCGAGAATCCATACATCCC
O. australiensis TCCGGGCGGTCCGGGGGGGGG-CACTACGGCTCCTCTCTTCTCGAGAATCCATACATCCC
O. brachyantha TCCGGGCGGTCCGGGGGGGGGGCACCACGGCTCCTCTCTTCTCGAGAATCCATACATCCC
O. longiglumis TCCGGGCGGTCCGGGGGGGGG-CACCACGGCTCCTCTCTTCTCGAGAATCCATACATCCC
O. ridleyi TCCGGGCGGTCCGGGGGGGGG-CACCACGGCTCCTCTCTTCTCGAGAATCCATACATCCC
O. granulata TCCGGGCGGTCCGGGGGGGGG-CACCACGGCTCCTCTCTTCTCGAGAATCCATACATCCC
O. meyeriana TCCGGGCGGTCCGGGGGGGGG-CACCACGGCTCCTCTCTTCTCGAGAATCCATACATCCC
L. japonica TCCGGGCGGTCCGGGGGGGGGGCACCACGGCTCCTCTCTTCTCGAGAATCCATACATCCC
******************** *** **********************************

O. punctata TTATCAGTGTATGGAGAGCTATCTCTCGAGCACAGGTTGAGGTTCGTCCTCAATGGGAAA
O. rhizomatis TTATCAGTGTATGGAGAGCTATCTCTCGAGCACAGGTTGAGGTTCGTCCTCAATGGGAAA
O. officinalis TTATCAGTGTATGGAGAGCTATCTCTCGAGCACAGGTTGAGGTTCGTCCTCAATGGGAAA
O. minuta TTATCAGTGTATGGAGAGCTATCTCTCGAGCACAGGTTGAGGTTCGTCCTCAATGGGAAA
O. meridionalis TTATCAGTGTATGGAGAGCTATCTCTCGAGCACAGGTTGAGGTTCGTCCTCAATGGGAAA
O. longistaminata TTATCAGTGTATGGAGAGCTATCTCTCGAGCACAGGTTGAGGTTCGTCCTCAATGGGAAA
O. latifolia TTATCAGTGTATGGAGAGCTATCTCTCGAGCACAGGTTGAGGTTCGTCCTCAATGGGAAA
O. grandiglumis TTATCAGTGTATGGAGAGCTATCTCTCGAGCACAGGTTGAGGTTCGTCCTCAATGGGAAA
O. eichingeri TTATCAGTGTATGGAGAGCTATCTCTCGAGCACAGGTTGAGGTTCGTCCTCAATGGGAAA
O. alta TTATCAGTGTATGGAGAGCTATCTCTCGAGCACAGGTTGAGGTTCGTCCTCAATGGGAAA
O. barthii TTATCAGTGTATGGAGAGCTATCTCTCGAGCACAGGTTGAGGTTCGTCCTCAATGGGAAA
O. glaberrima TTATCAGTGTATGGAGAGCTATCTCTCGAGCACAGGTTGAGGTTCGTCCTCAATGGGAAA
O. glumaepatula TTATCAGTGTATGGAGAGCTATCTCTCGAGCACAGGTTGAGGTTCGTCCTCAATGGGAAA
O. sativa ssp *indica* TTATCAGTGTATGGAGAGCTATCTCTCGAGCACAGGTTGAGGTTCGTCCTCAATGGGAAA
O. sativa ssp *japonica* TTATCAGTGTATGGAGAGCTATCTCTCGAGCACAGGTTGAGGTTCGTCCTCAATGGGAAA
O. nivara TTATCAGTGTATGGAGAGCTATCTCTCGAGCACAGGTTGAGGTTCGTCCTCAATGGGAAA
O. rufipogon TTATCAGTGTATGGAGAGCTATCTCTCGAGCACAGGTTGAGGTTCGTCCTCAATGGGAAA
O. australiensis TTATCAGTGTATGGAGAGCTATCTCTCGAGCACAGGTTGA--------------------
O. brachyantha TTATCAGTGTATGGAGAGCTATCTCTCGAGCACAGGTTGAGGTTCGTCCTCAATGGGAAA
O. longiglumis TTATCAGTGTATGGAGAGCTATCTCTCGAGCACAGGTTGA--------------------
O. ridleyi TTATCAGTGTATGGAGAGCTATCTCTCGAGCACAGGTTGA--------------------
O. granulata TTATCAGTGTATGGAGAGCTATCTCTCGAGCACAGGTTGA--------------------
O. meyeriana TTATCAGTGTATGGAGAGCTATCTCTCGAGCACAGGTTGA--------------------
L. japonica TTATCAGTGTATGGAGAGCTATCTCTCGAGCACAGGTTGAGGTTCGTCCTCAATGGGAAA
****

O. punctata	ATGGAGCACCTAACAACGCATCTTCACAGACCAAGAACTACGAGATCACCCCTTTCATTC
O. rhizomatis	ATGGAGCACCTAACAACGCATCTTCACAGACCAAGAACTACGAGATCACCCCTTTCATTC
O. officinalis	ATGGAGCACCTAACAACGCATCTTCACAGACCAAGAACTACGAGATCACCCCTTTCATTC
O. minuta	ATGGAGCACCTAACAACGCATCTTCACAGACCAAGAACTACGAGATCACCCCTTTCATTC
O. meridionalis	ATGGAGCACCTAACAACGCATCTTCACAGACCAAGAACTACGAGATCACCCCTTTCATTC
O. longistaminata	ATGGAGCACCTAACAACGCATCTTCACAGACCAAGAACTACGAGATCACCCCTTTCATTC
O. latifolia	ATGGAGCACCTAACAACGCATCTTCACAGACCAAGAACTACGAGATCACCCCTTTCATTC
O. grandiglumis	ATGGAGCACCTAACAACGCATCTTCACAGACCAAGAACTACGAGATCACCCCTTTCATTC
O. eichingeri	ATGGAGCACCTAACAACGCATCTTCACAGACCAAGAACTACGAGATCACCCCTTTCATTC
O. alta	ATGGAGCACCTAACAACGCATCTTCACAGACCAAGAACTACGAGATCACCCCTTTCATTC
O. barthii	ATGGAGCACCTAACAACGCATCTTCACAGACCAAGAACTACGAGATCACCC-TTTCATTC
O. glaberrima	ATGGAGCACCTAACAACGCATCTTCACAGACCAAGAACTACGAGATCACCC-TTTCATTC
O. glumaepatula	ATGGAGCACCTAACAACGCATCTTCACAGACCAAGAACTACGAGATCACCC-TTTCATTC
O. sativa ssp *indica*	ATGGAGCACCTAACAACGCATCTTCACAGACCAAGAACTACGAGATCACCC-TTTCATTC
O. sativa ssp *japonica*	ATGGAGCACCTAACAACGCATCTTCACAGACCAAGAACTACGAGATCACCC-TTTCATTC
O. nivara	ATGGAGCACCTAACAACGCATCTTCACAGACCAAGAACTACGAGATCACCC-TTTCATTC
O. rufipogon	ATGGAGCACCTAACAACGCATCTTCACAGACCAAGAACTACGAGATCACCC-TTTCATTC
O. australiensis	--
O. brachyantha	ATGGAGCACCTAACAACGCATCTTCACAGACCAAGAACTACGAGATCACCCCTTTCATTC
O. longiglumis	--
O. ridleyi	--
O. granulata	--
O. meyeriana	--
L. japonica	ATGGAGCACCTAACAACGCATCTTCACAGACCAAGAACTACGAGATCACCCCTTTCATTC

O. punctata	TGGGGTGA--
O. rhizomatis	TGGGGTGA--
O. officinalis	TGGGGTGA--
O. minuta	TGGGGTGA--
O. meridionalis	TGGGGTGA--
O. longistaminata	TGGGGTGA--
O. latifolia	TGGGGTGA--

O. grandiglumis	TGGGGTGA--
O. eichingeri	TGGGGTGA--
O. alta	TGGGGTGA--
O. barthii	TGGGGTGACGGAGGGATCGTACCATTCGAGCCTTTTTTTCATGCTTTTCCCGGCGGTCTG
O. glaberrima	TGGGGTGACGGAGGGATCGTACCATTCGAGCCTTTTTTTCATGCTTTTCCCGGCGGTCTG
O. glumaepatula	TGGGGTGACGGAGGGATCGTACCATTCGAGCCTTTTTTTCATGCTTTTCCCGGCGGTCTG
O. sativa ssp *indica*	TGGGGTGACGGAGGGATCGTACCATTCGAGCCTTTTTTTCATGCTTTTCCCGGCGGTCTG
O. sativa ssp *japonica*	TGGGGTGACGGAGGGATCGTACCATTCGAGCCTTTTTTTCATGCTTTTCCCGGCGGTCTG
O. nivara	TGGGGTGACGGAGGGATCGTACCATTCGAGCCTTTTTTTCATGCTTTTCCCGGCGGTCTG
O. rufipogon	TGGGGTGACGGAGGGATCGTACCATTCGAGCCTTTTTTTCATGCTTTTCCCGGCGGTCTG
O. australiensis	--
O. brachyantha	TGGGGTGACGGAGGGATCGTACCATTCGAGCCTTTTTTTCATGCTTTTCCCGGCGGTCTG
O. longiglumis	--
O. ridleyi	--
O. granulata	--
O. meyeriana	--
L. japonica	TGGGGTGACGGAGGGATCGTACCATTCGAGCCTTTTTTTCATGCTTTTCCCGGCGGTCTG

O. punctata	---
O. rhizomatis	---
O. officinalis	---
O. minuta	---
O. meridionalis	---
O. longistaminata	---
O. latifolia	---
O. grandiglumis	---
O. eichingeri	---
O. alta	---
O. barthii	GAGAAAGCAGCAATCAATAGGACTTCCCTAATCCTCCCTTCCTGA
O. glaberrima	GAGAAAGCAGCAATCAATAGGACTTCCCTAATCCTCCCTTCCTGA
O. glumaepatula	GAGAAAGCAGCAATCAATAGGACTTCCCTAATCCTCCCTTCCTGA
O. sativa ssp *indica*	GAGAAAGCAGCAATCAATAGGACTTCCCTAATCCTCCCTTCCTGA
O. sativa ssp *japonica*	GAGAAAGCAGCAATCAATAGGACTTCCCTAATCCTCCCTTCCTGA
O. nivara	GAGAAAGCAGCAATCAATAGGACTTCCCTAATCCTCCCTTCCTGA
O. rufipogon	GAGAAAGCAGCAATCAATAGGACTTCCCTAATCCTCCCTTCCTGA
O. australiensis	---
O. brachyantha	GAGAAAGCAGCAATCAATAGGACTTCCCTAATCCTCCCTTCCTGA
O. longiglumis	---
O. ridleyi	---
O. granulata	---
O. meyeriana	---
L. japonica	GAGAAAGCAGCAATCAATAGGACTTCCTTAATCCTCCCTTCCTGA

Supplementary Figure 4. Alignment of nucleotide sequences of *ycf68* in the twenty-two *Oryza* species and *L. japonica*. Allelic variants were marked in red and blue, respectively.

O. ridleyi	MAYSSCLNRSLKPNKLILRRIDGAIQVRSHVDLTFYSLVGSGRSGGGHHG
O. longiglumis	MAYSSCLNRSLKPNKLILRRIDGAIQVRSHVDLTFYSLVGSGRSGGGHHG
O. granulata	MAYSSCLNRSLKPNKLLLRRIDGAIQVRSHVDLTFYSLVGSGRSGGGHHG
O. meyeriana	MAYSSCLNRSLKPNKLLLRRIDGAIQVRSHVDLTFYSLVGSGRSGGGHHG
O. australiensis	MAYSSCLNRSLKPNKLLLRRIDGAIQVRSHVDLTFYSLVGSGRSGGGHYG
O. minuta	MAYSSCLNRSLKPNKLLLRRIDGAIQVRSHVDLTFYSLVGSGRSGGGTTA
O. latifolia	MAYSSCLNRSLKPNKLLLRRIDGAIQVRSHVDLTFYSLVGSGRSGGGTTA
O. grandiglumis	MAYSSCLNRSLKPNKLLLRRIDGAIQVRSHVDLTFYSLVGSGRSGGGTTA
O. eichingeri	MAYSSCLNRSLKPNKLLLRRIDGAIQVRSHVDLTFYSLVGSGRSGGGTTA
O. officinalis	MAYSSCLNRSLKPNKLLLRRIDGAIQVRSHVDLTFYSLVGSGRSGGGTTA
O. longistaminata	MAYSSCLNRSLKPNKLLLRRIDGAIQVRSHVDLTFYSLVGSGRSGGGTTA
O. punctata	MAYSSCLNRSLKPNKLLLRRIDGAIQVRSHVDLTFYSLVGSGRSGGGTTA
O. meridionalis	MAYSSCLNRSLKPNKLLLRRIDGAIQVRSHVDLTFYSLVGSGRSGGGTTA
O. rhizomatis	MAYSSCLNRSLKPNKLLLRRIDGAIQVRSHVDLTFYSLVGSGRSGGGTTA
O. alta	MAYSSCLNRSLKPNKLLLRRIDGAIQVRSHVDLTFYSLVGSGRSGGGTTA
O. barthii	MAYSSCLNRSLKPNKLLLRRIDGAIQVRSHVDLTFYSLVGSGRSGGGTTA
O. glumaepatula	MAYSSCLNRSLKPNKLLLRRIDGAIQVRSHVDLTFYSLVGSGRSGGGTTA
O. rufipogon	MAYSSCLNRSLKPNKLLLRRIDGAIQVRSHVDLTFYSLVGSGRSGGGTTA
O. nivara	MAYSSCLNRSLKPNKLLLRRIDGAIQVRSHVDLTFYSLVGSGRSGGGTTA
O. sativa ssp *japonica*	MAYSSCLNRSLKPNKLLLRRIDGAIQVRSHVDLTFYSLVGSGRSGGGTTA
O. glaberrima	MAYSSCLNRSLKPNKLLLRRIDGAIQVRSHVDLTFYSLVGSGRSGGGTTA
O. sativa ssp *indica*	MAYSSCLNRSLKPNKLLLRRIDGAIQVRSHVDLTFYSLVGSGRSGGGTTA
O. brachyantha	MAYSSCLNRSLKPNKLLLRRIDGAIQVRSHVDLTFYSLVGSGRSGGGAPR
L. japonica	MAYSSCLNRSLKPNKLLLRRIDGAIQVRSHVDLTFYSLVGSGRSGGGAPR
	****************:******************************

O. ridleyi	-------SSLL------------------EN--PYIPYQCMESYLSSTG-
O. longiglumis	-------SSLL------------------EN--PYIPYQCMESYLSSTG-
O. granulata	-------SSLL------------------EN--PYIPYQCMESYLSSTG-
O. meyeriana	-------SSLL------------------EN--PYIPYQCMESYLSSTG-
O. australiensis	-------SSLL------------------EN--PYIPYQCMESYLSSTG-
O. minuta	PLFSRIHTSLISVWRAISRAQ-VEVRPQWENGAPNNASSQTKNYEITPFI
O. latifolia	PLFSRIHTSLISVWRAISRAQ-VEVRPQWENGAPNNASSQTKNYEITPFI
O. grandiglumis	PLFSRIHTSLISVWRAISRAQ-VEVRPQWENGAPNNASSQTKNYEITPFI
O. eichingeri	PLFSRIHTSLISVWRAISRAQ-VEVRPQWENGAPNNASSQTKNYEITPFI
O. officinalis	PLFSRIHTSLISVWRAISRAQ-VEVRPQWENGAPNNASSQTKNYEITPFI
O. longistaminata	PLFSRIHTSLISVWRAISRAQ-VEVRPQWENGAPNNASSQTKNYEITPFI
O. punctata	PLFSRIHTSLISVWRAISRAQ-VEVRPQWENGAPNNASSQTKNYEITPFI
O. meridionalis	PLFSRIHTSLISVWRAISRAQ-VEVRPQWENGAPNNASSQTKNYEITPFI
O. rhizomatis	PLFSRIHTSLISVWRAISRAQ-VEVRPQWENGAPNNASSQTKNYEITPFI
O. alta	PLFSRIHTSLISVWRAISRAQ-VEVRPQWENGAPNNASSQTKNYEITPFI
O. barthii	PLFSRIHTSLISVWRAISRAQ-VEVRPQWENGAPNNASSQTKNYEITLSF
O. glumaepatula	PLFSRIHTSLISVWRAISRAQ-VEVRPQWENGAPNNASSQTKNYEITLSF
O. rufipogon	PLFSRIHTSLISVWRAISRAQ-VEVRPQWENGAPNNASSQTKNYEITLSF

```
O. nivara                  PLFSRIHTSLISVWRAISRAQ-VEVRPQWENGAPNNASSQTKNYEITLSF
O. sativa ssp japonica     PLFSRIHTSLISVWRAISRAQ-VEVRPQWENGAPNNASSQTKNYEITLSF
O. glaberrima              PLFSRIHTSLISVWRAISRAQ-VEVRPQWENGAPNNASSQTKNYEITLSF
O. sativa ssp indica       PLFSRIHTSLISVWRAISRAQ-VEVRPQWENGAPNNASSQTKNYEITLSF
O. brachyantha             LLSSRESIHPLSVYGELSLEHRLRFVLNGKMEHLTTHLHRPRTTRSPLSF
L. japonica                LLSSRESIHPLSVYGELSLEHRLRFVLNGKMEHLTTHLHRPRTTRSPLSF
                                     :                  :           ..   .
```

```
O. ridleyi                 ----------------------------------
O. longiglumis             ----------------------------------
O. granulata               ----------------------------------
O. meyeriana               ----------------------------------
O. australiensis           ----------------------------------
O. minuta                  LG--------------------------------
O. latifolia               LG--------------------------------
O. grandiglumis            LG--------------------------------
O. eichingeri              LG--------------------------------
O. officinalis             LG--------------------------------
O. longistaminata          LG--------------------------------
O. punctata                LG--------------------------------
O. meridionalis            LG--------------------------------
O. rhizomatis              LG--------------------------------
O. alta                    LG--------------------------------
O. barthii                 WG--------------------------------
O. glumaepatula            WGDGGIVPFEPFFHAFPGGLEKAAINRTSLILPS
O. rufipogon               WGDGGIVPFEPFFHAFPGGLEKAAINRTSLILPS
O. nivara                  WGDGGIVPFEPFFHAFPGGLEKAAINRTSLILPS
O. sativa ssp japonica     WGDGGIVPFEPFFHAFPGGLEKAAINRTSLILPS
O. glaberrima              WGDGGIVPFEPFFHAFPGGLEKAAINRTSLILPS
O. sativa ssp indica       WGDGGIVPFEPFFHAFPGGLEKAAINRTSLILPS
O. brachyantha             WGDGGIVPFEPFFHAFPGGLEKAAINRTSLILPS
L. japonica                WGDGGIVPFEPFFHAFPGGLEKAAINRTSLILPS
```

Supplementary Figure 5. Alignment of amino acid sequences of *ycf68* in the twenty-two *Oryza* species and *L. japonica*.

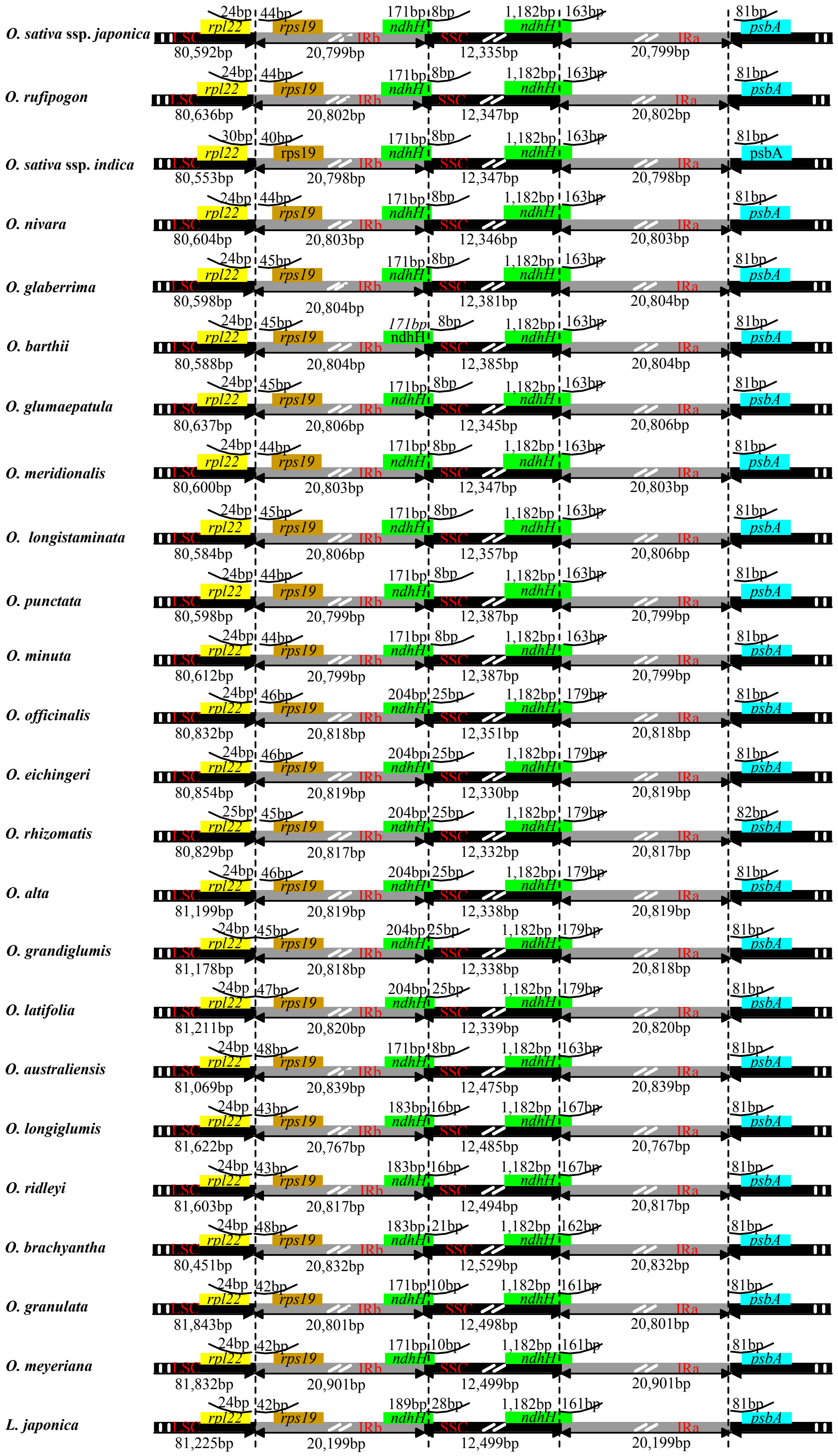

Supplementary Figure 6. Chloroplast genome IR junctions of the twenty-two *Oryza* species and *L. japonica*.

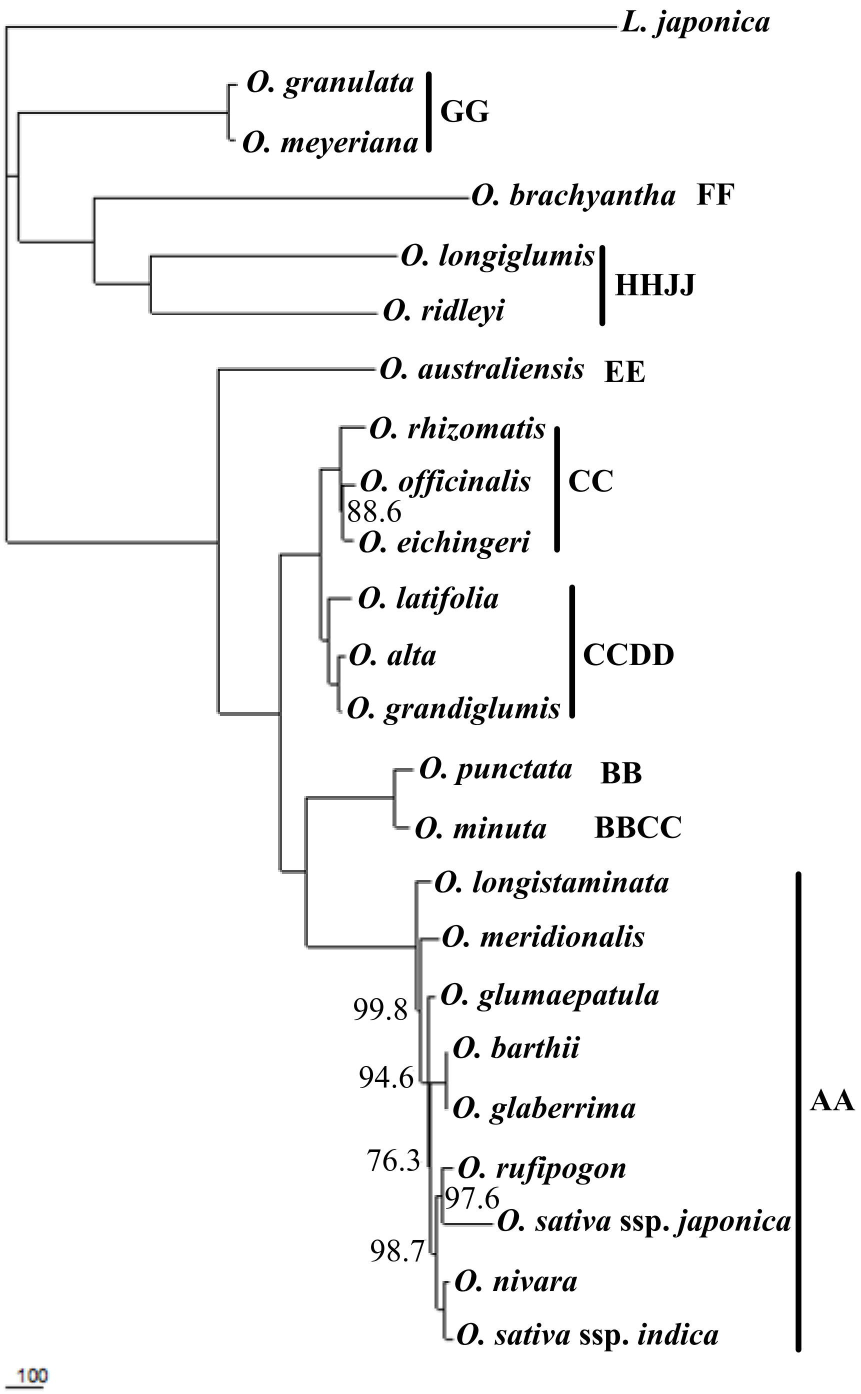

Supplementary Figure 7. MP phylogeny of *Oryza* inferred from the whole-genome sequences of chloroplasts using *L. japonica* as outgroup. Numbers near branches indicate bootstrap percentages, and the branches without numbers show 100% bootstrap support.

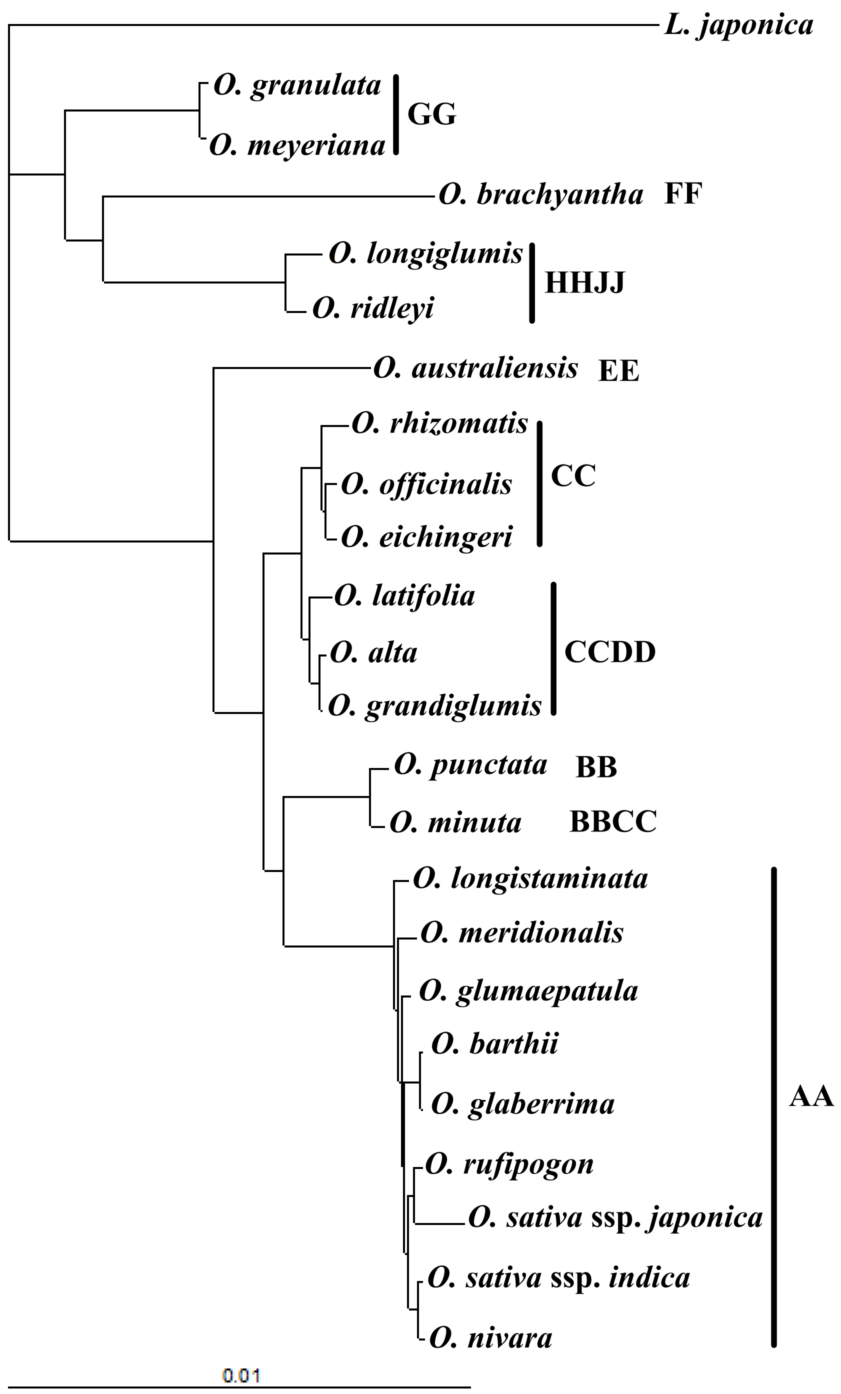

Supplementary Figure 8. BI phylogeny of *Oryza* inferred from the whole-genome sequences of chloroplasts using *L. japonica* as outgroup. The branches without numbers indicate 1.0 bootstrap supports.

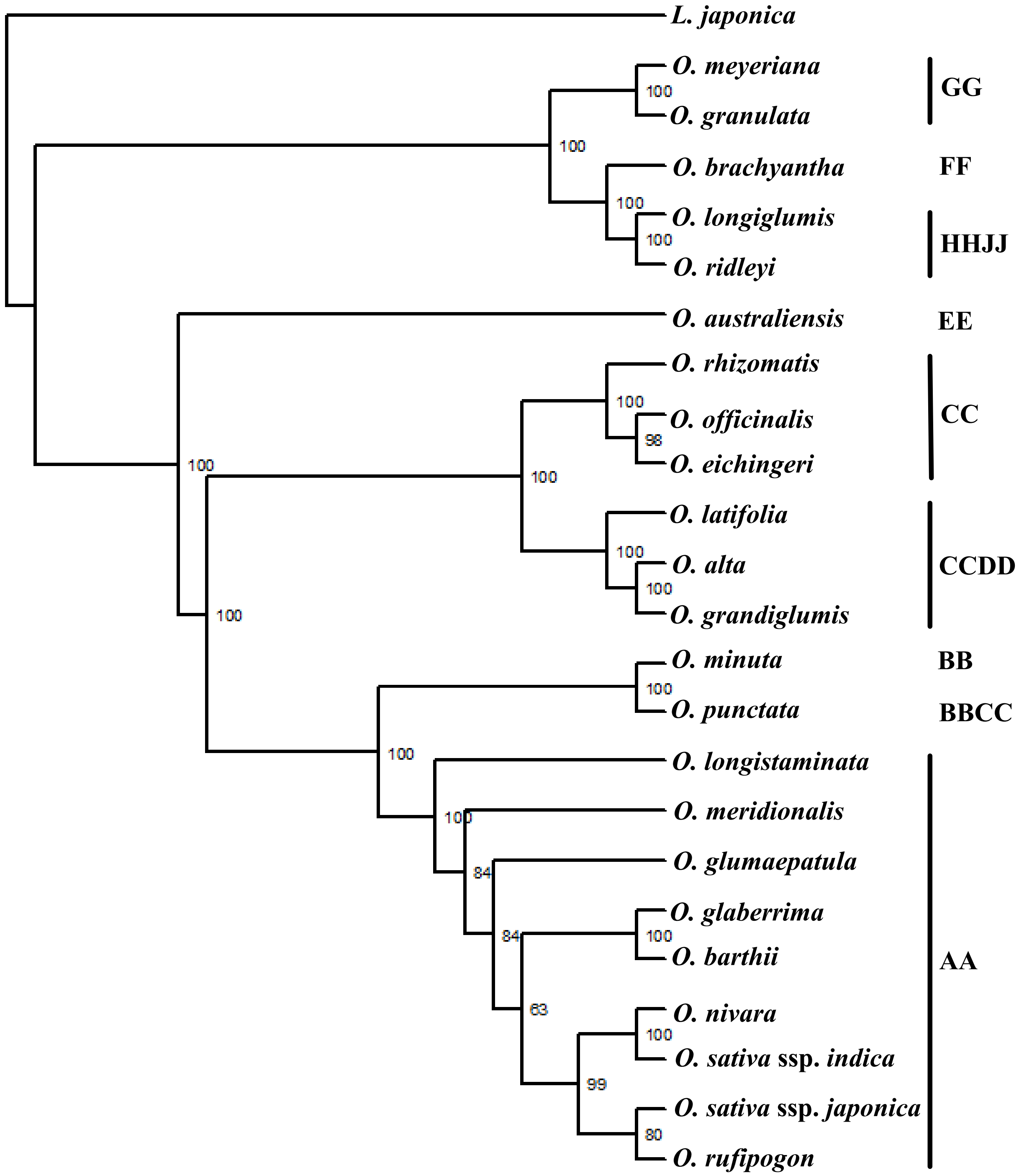

Supplementary Figure 9. ML phylogeny of *Oryza* inferred from the concatenated sequences of noncoding sequences of the chloroplast genome using *L. japonica* as outgroup.

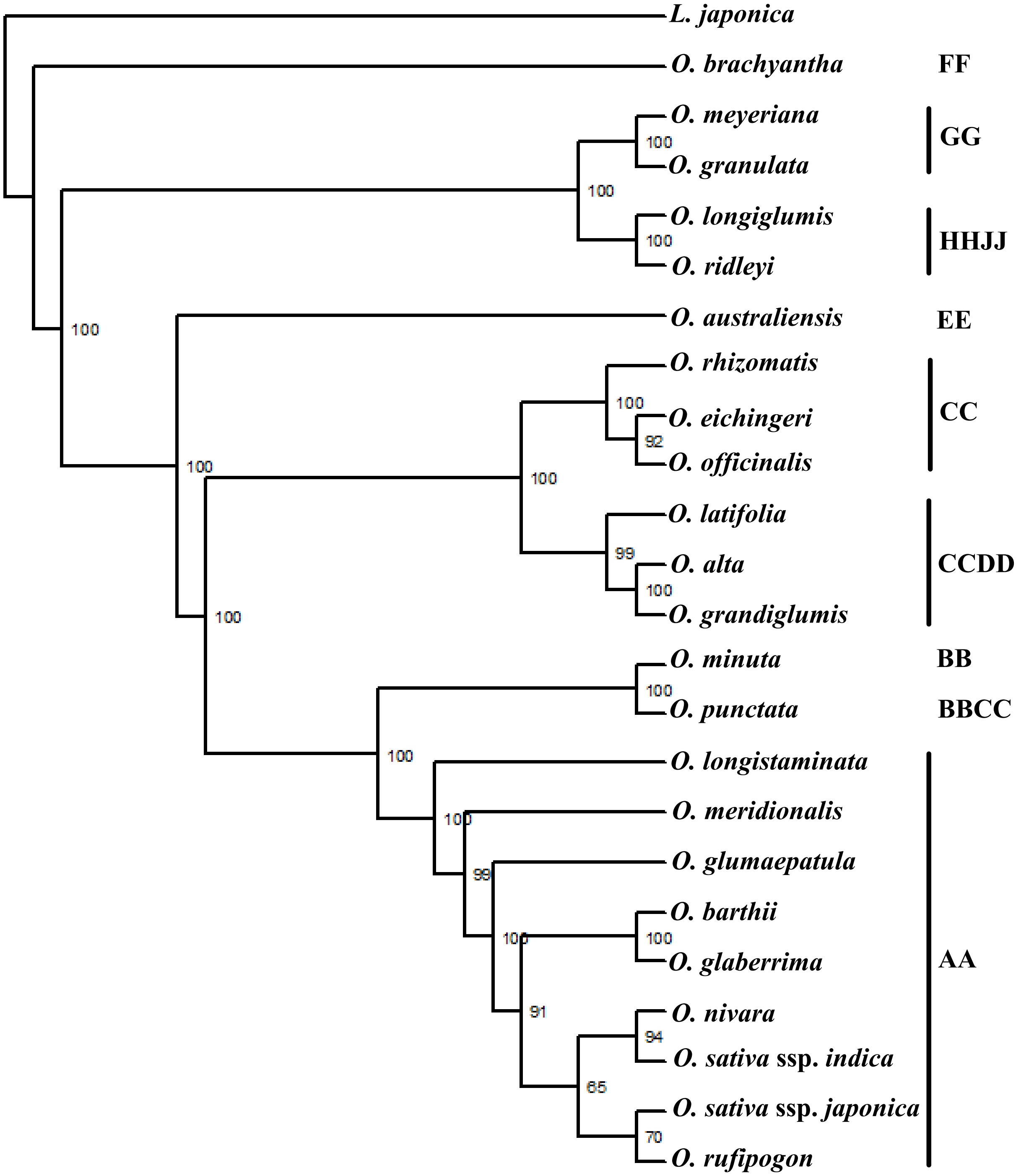

Supplementary Figure 10. ML phylogeny of *Oryza* inferred from the concatenated sequences of 77 protein-coding genes using *L. japonica* as outgroup.

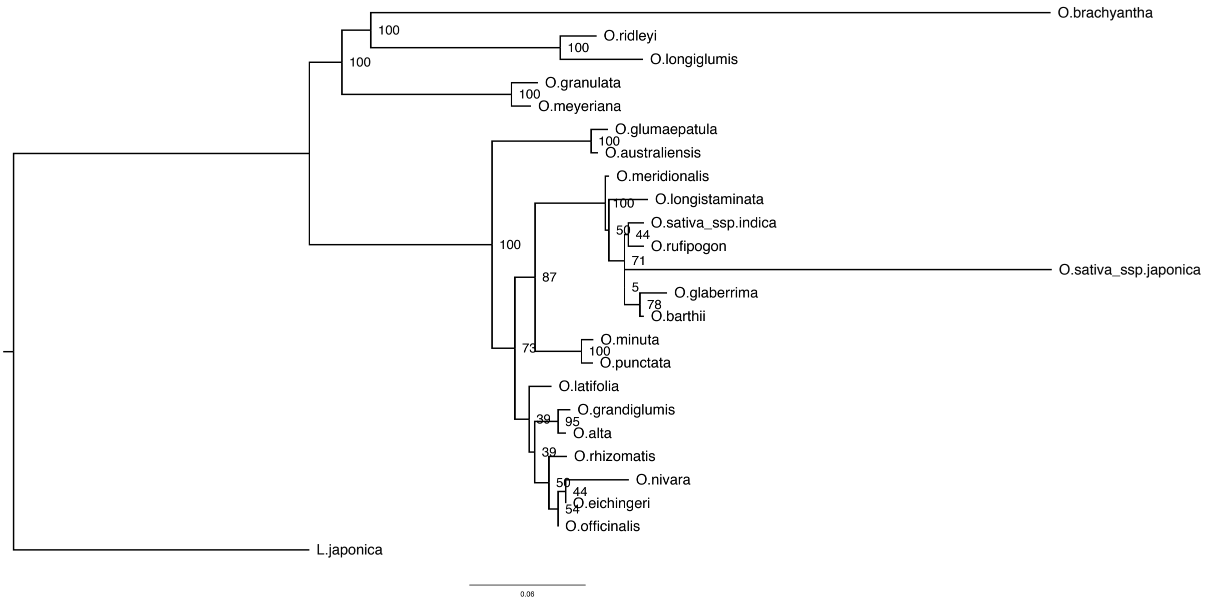

Supplementary Figure 11. Phylogeny of *Oryza* inferred from ML analysis of genomic indels as simple indel coding (SIC) from MSAs produced by MAFFT and filtered by the RELINDEL method.

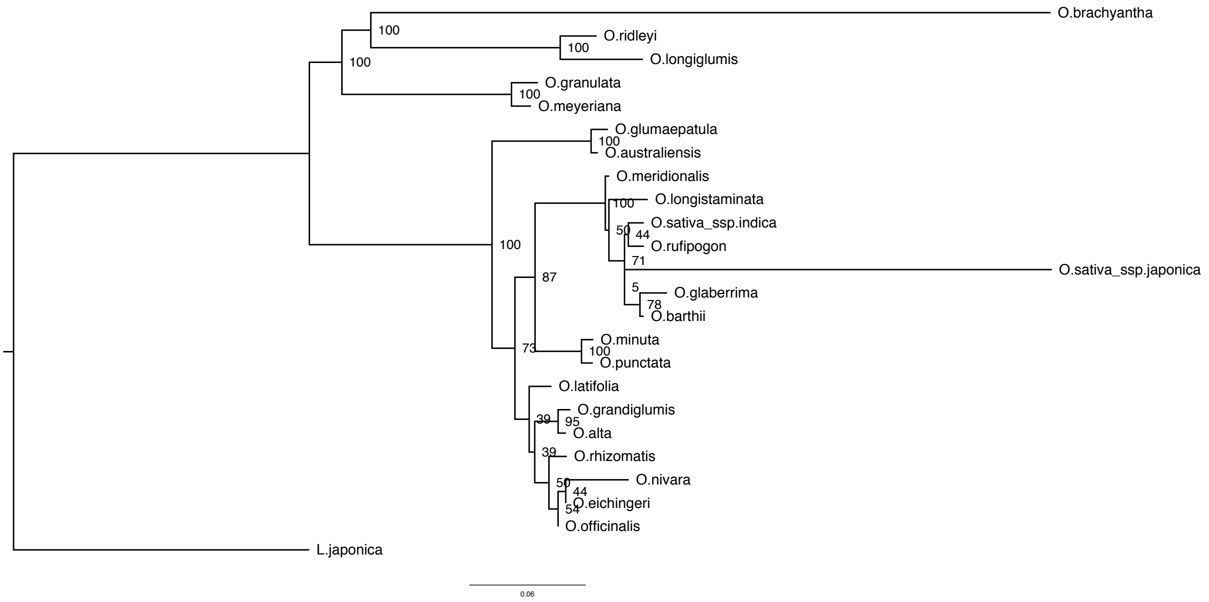

Supplementary Figure 12. Phylogeny of *Oryza* inferred from MP analysis of indels from protein-coding genes as fifth state (5th state) from MSAs produced by PRANK+F and filtered by the Gblock method.

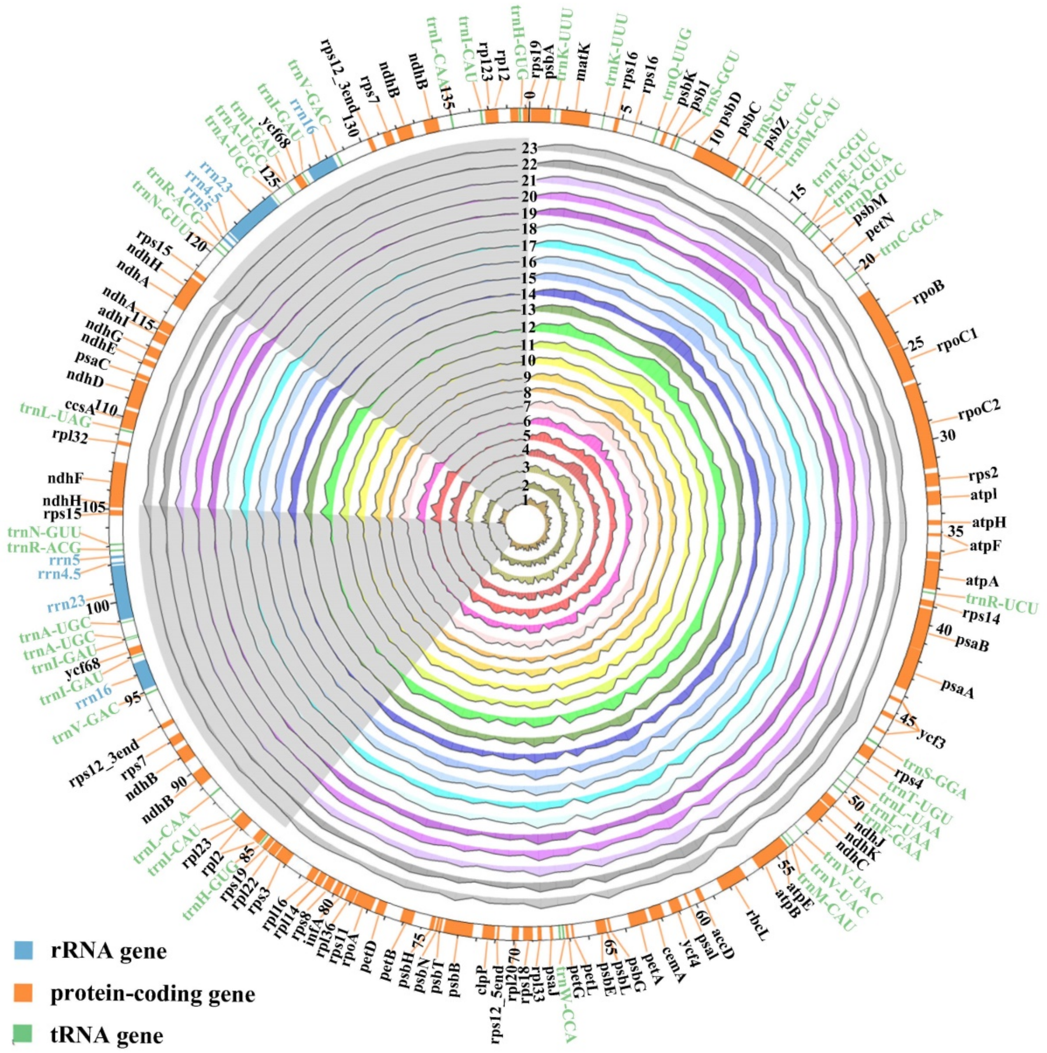

Supplementary Figure 13. A map of chloroplast nucleotide variation across the *Oryza* plastomes. The SNV density of 1Kb was measured for the twenty-three *Oryza* chloroplast genomes using *L. japonica* as outgroup. In the quadripartite structure of these chloroplast genomes, the two IR regions (IR_A and IR_B) are shown with grey background, while the large and small single-copy regions (LSC and SSC) are displayed with blank background. For each *Oryza* chloroplast genome, the levels of SNVs are genome-wide presented in different colors. The studied *Oryza* species are indicated as below: 1. *O. sativa* ssp. *japonica*; 2. *O. rufipogon*; 3. *O. sativa* ssp. *indica*; 4. *O. nivara*; 5. *O. glaberrima*; 6. *O. barthii*; 7. *O. glumaepatula*; 8. *O. meridionalis*; 9. *O. longistaminata*; 10. *O. punctate*; 11. *O. minuta*; 12. *O. officinalis*; 13. *O. eichingeri*; 14. *O. rhizomatis*; 15. *O. alta*; 16. *O. grandiglumis*; 17. *O. latifolia*; 18. *O. australiensis*; 19. *O. longiglumis*; 20. *O. ridleyi*; 21. *O. brachyantha*; 22. *O. granulata*; 23. *O. meyeriana*; 24. *L. japonica*.)

A

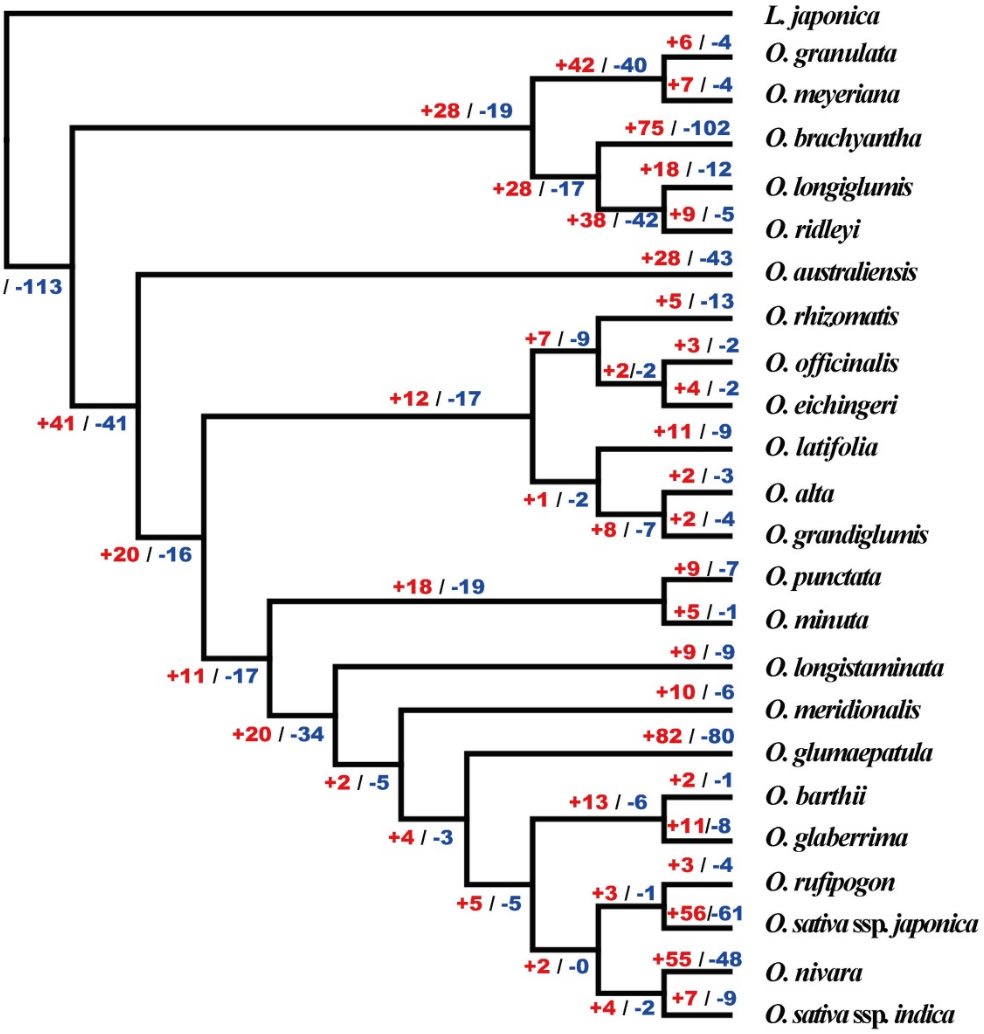

L. japonica
O. granulata
O. meyeriana
O. brachyantha
O. longiglumis
O. ridleyi
O. australiensis
O. rhizomatis
O. officinalis
O. eichingeri
O. latifolia
O. alta
O. grandiglumis
O. punctata
O. minuta
O. longistaminata
O. meridionalis
O. glumaepatula
O. barthii
O. glaberrima
O. rufipogon
O. sativa ssp. japonica
O. nivara
O. sativa ssp. indica
+6 / -4
+7 / -4
+42 / -40
+28 / -19
+75 / -102
+18 / -12
+9 / -5
+28 / -17
+38 / -42
+28 / -43
+2 / -113
+5 / -13
+7 / -9
+3 / -2
+2/-2
+4 / -2
+12 / -17
+11 / -9
+41 / -41
+2 / -3
+1 / -2
+2 / -4
+8 / -7
+20 / -16
+9 / -7
+18 / -19
+5 / -1
+9 / -9
+11 / -17
+10 / -6
+20 / -34
+82 / -80
+2 / -5
+2 / -1
+13 / -6
+11/-8
+4 / -3
+3 / -4
+3 / -1
+5 / -5
+56/-61
+55 / -48
+2 / -0
+7 / -9
+4 / -2

B

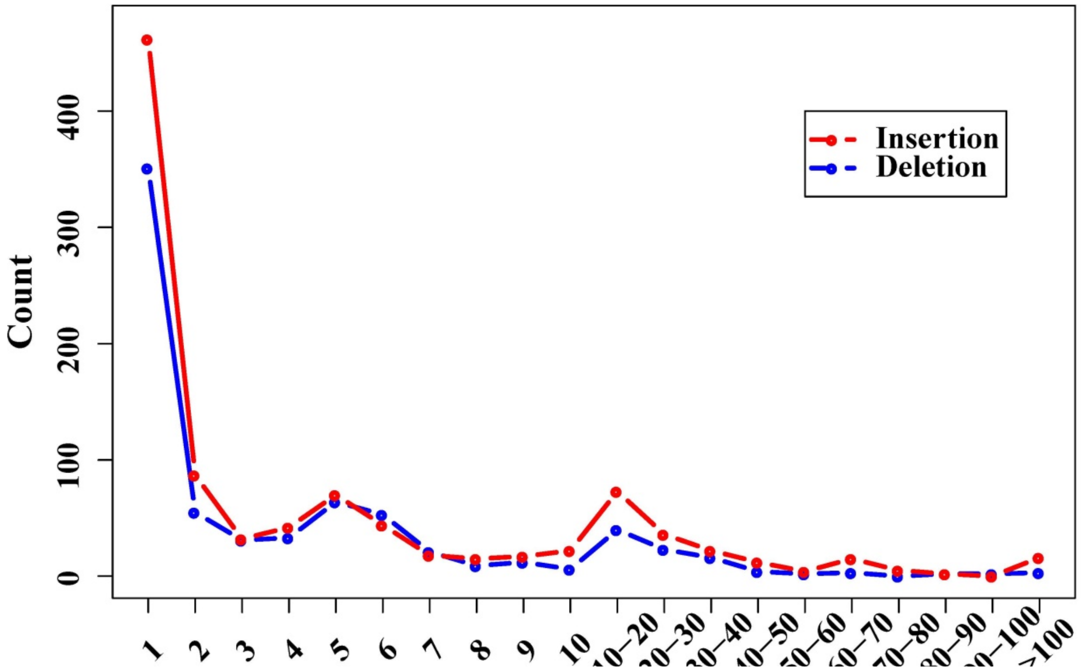

Insertion
Deletion
Count
0
100
200
300
400
1
2
3
4
5
6
7
8
9
10

C

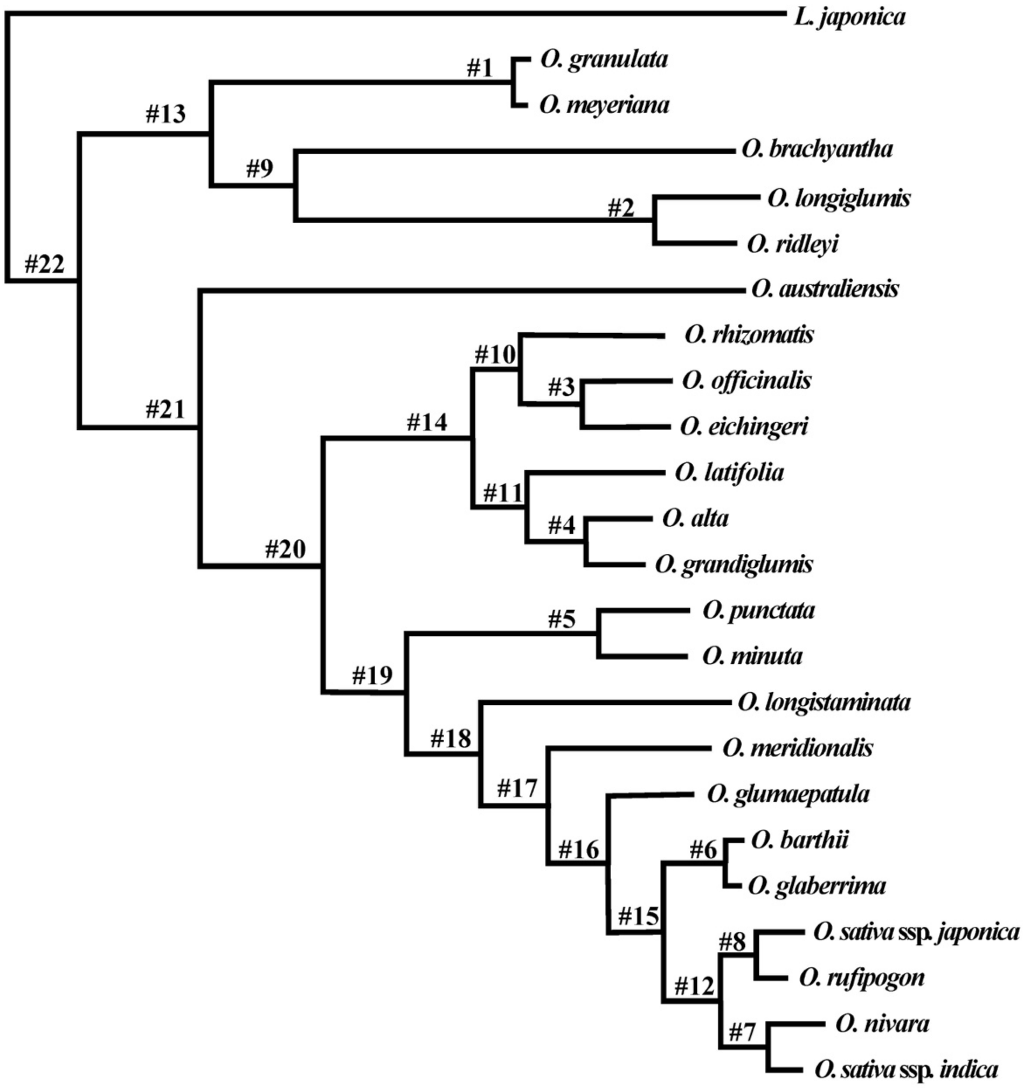
L. japonica
#1
O. granulata
O. meyeriana
#13
O. brachyantha
#9
#2
O. longiglumis
O. ridleyi
#22
O. australiensis
O. rhizomatis
#10
#3
O. officinalis
O. eichingeri
#21
#14
O. latifolia
#11
#4
O. alta
O. grandiglumis
#20
#5
O. punctata
O. minuta
#19
O. longistaminata
#18
O. meridionalis
#17
O. glumaepatula
#16
#6
O. barthii
O. glaberrima
#15
#8
O. sativa ssp. japonica
O. rufipogon
#12
#7
O. nivara
O. sativa ssp. indica

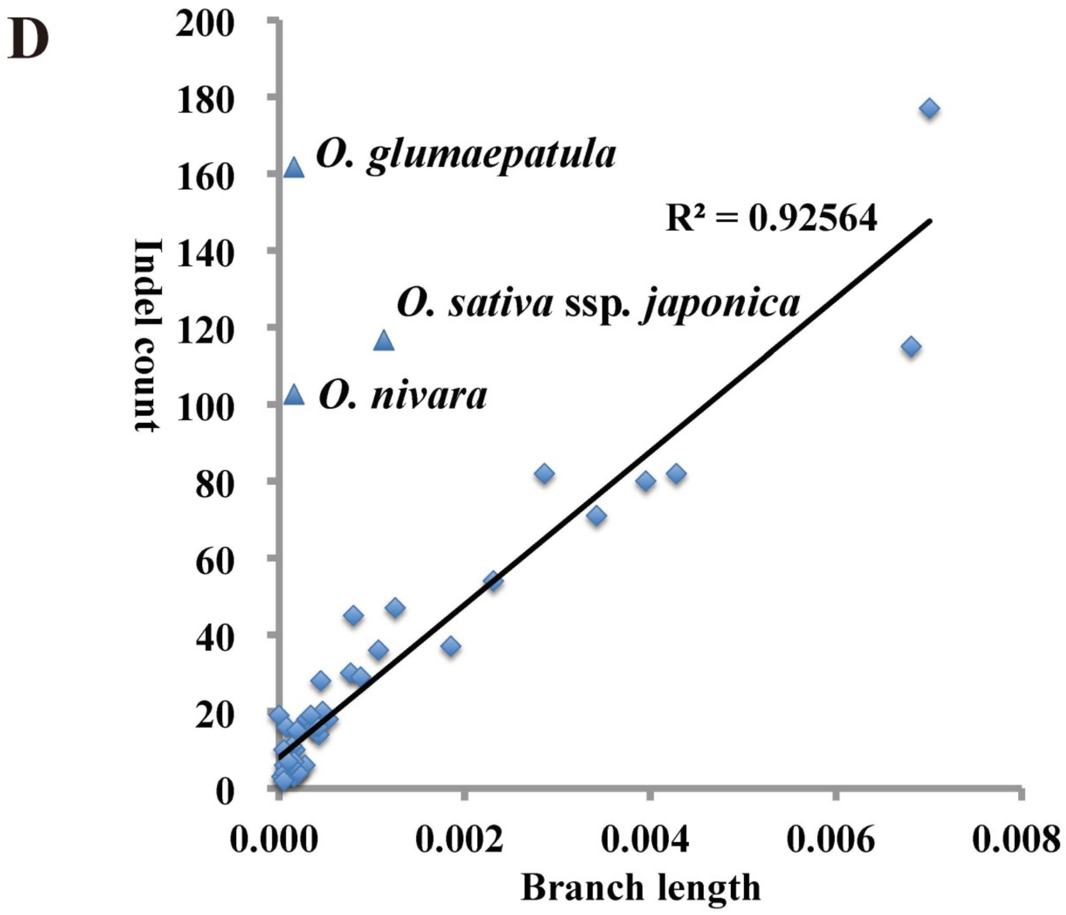

Supplementary Figure 14. The rate of chloroplast genomic variation in the genus *Oryza*. (A) The detection of insertion and deletion events occurred in different *Oryza* plastomes; (B) overall length distribution of insertions and deletions across the twenty-three *Oryzeae* chloroplast genomes; (C) numbers of all *Oryza* branches to characterize the occurrence of chloroplast genomic variation; (D)correlation between branch lengths and indel numbers for the twenty-three *Oryza* and *L. japonica* chloroplast genomes.**)**

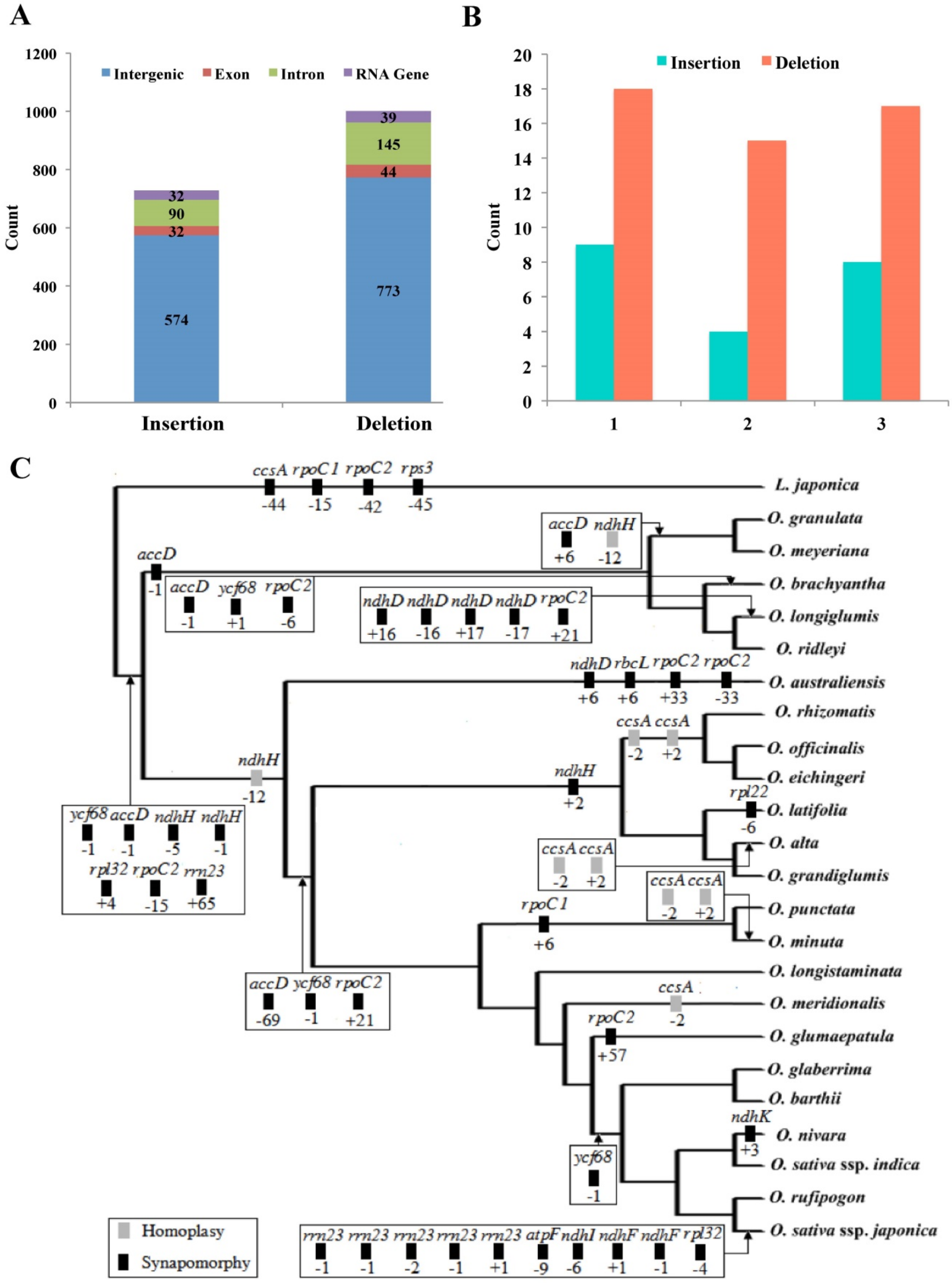

Supplementary Figure 15. InDels located within the *Oryza* chloroplast genes. (A) Numbers of insertions and deletions located within intergenic genomic regions, exons and introns of protein-coding genes and RNA genes; (B) length distribution of indels that influence the frameshift of the resided protein-coding genes. Numbers of insertions and deletions are given in terms of indel length of indels that are multiples of three (I) and not (II);

(C) phylogenetic distribution of indels within exons of chloroplast genes in the genus *Oryza* using *L. japonica* as outgroup. The insertions and deletions are indicated by black and gray bars. Gene names are given above boxes, and sizes of indels (bp) and polarity ('+' = insertion, '-' = deletion) are shown below boxes. Polarity of mutations was determined in comparisons to *L. japonica* as outgroup. The topology of the studied rice species was based on ML phylogeny reconstructed by complete chloroplast genomes.

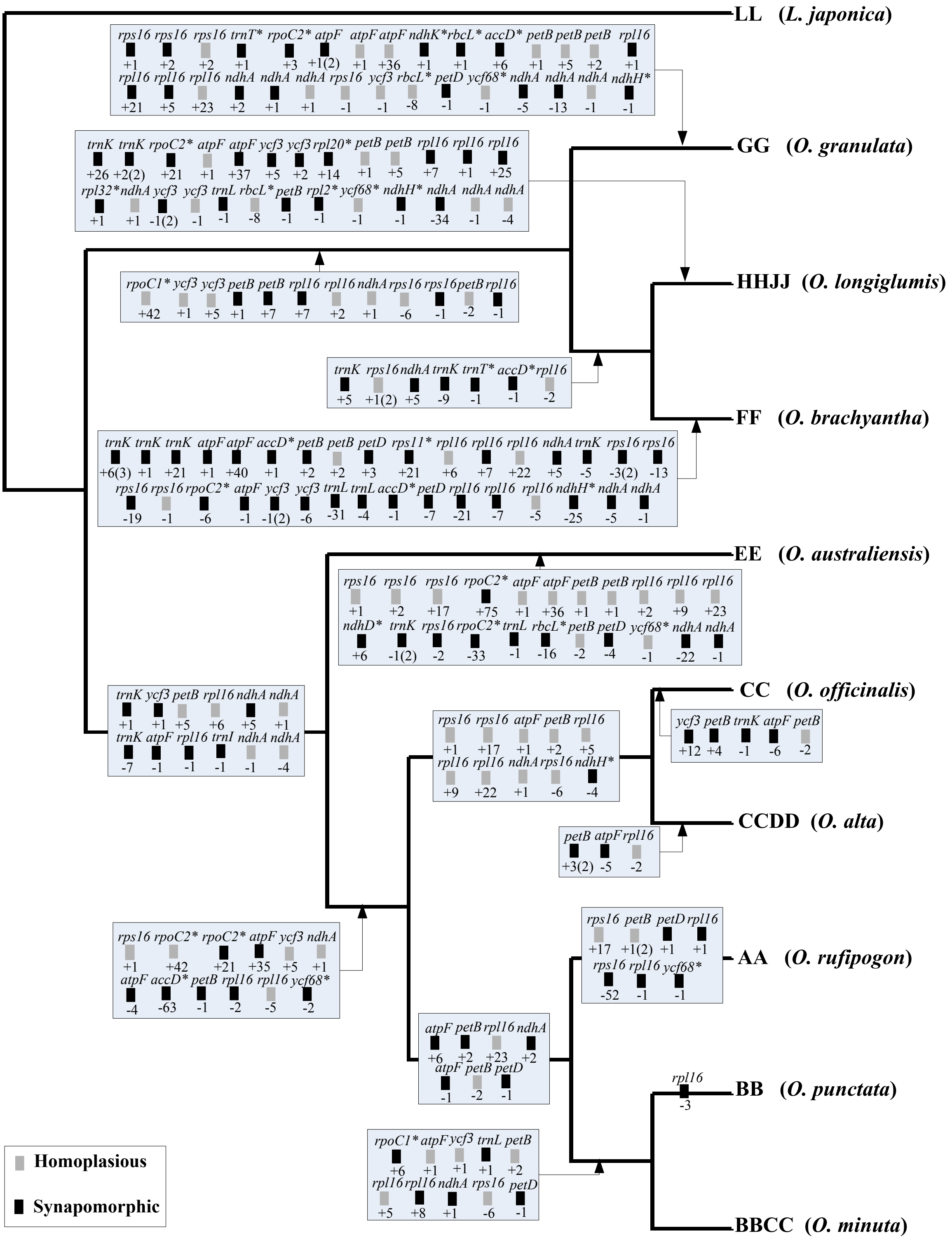

Supplementary Figure 16. Phylogenetic distribution of indels among different *Oryza* genome types using *L. japonica* as outgroup. The insertions and deletions within introns and exons (marked as *) are displayed by using black and gray bars on each branch. Gene names are given above bars, and sizes of indels (bp) and polarity ('+' = insertion, '-' = deletion) are shown below bars. Polarity of mutations was determined in comparisons to *L. japonica* as outgroup. The topology of the studied rice species was based on ML phylogeny reconstructed by complete chloroplast genomes.

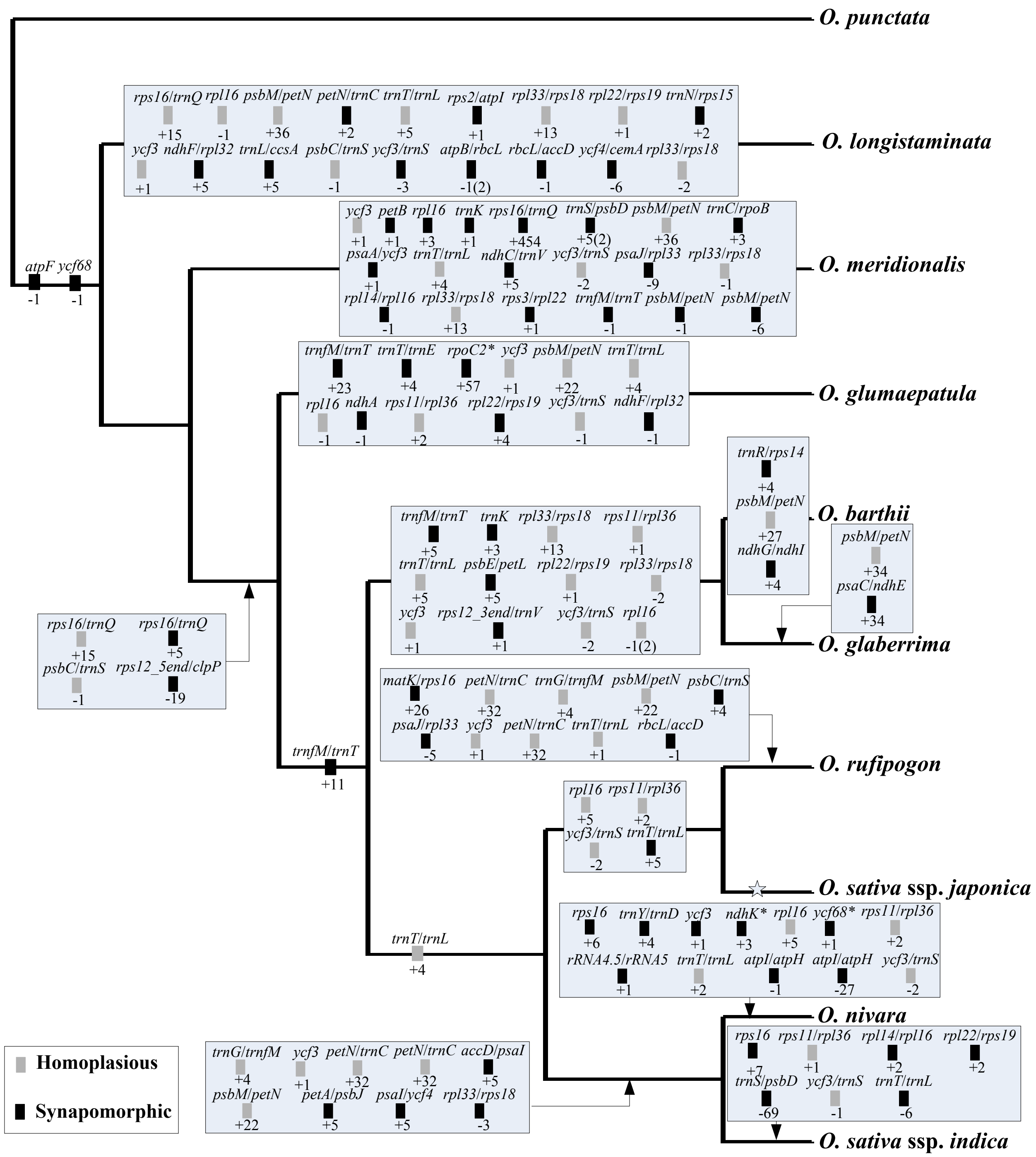

Supplementary Figure 17. Phylogenetic distribution of indels among AA- genome *Oryza* species using *O. punctata* as outgroup. The insertions and deletions of intergenic regions (with '/'), introns and exons (marked as '*') are indicated by black and gray bars on each branch. Gene names are given above bars, and sizes of indels (bp) and polarity ('+' = insertion, '-' = deletion) are shown below bars. Polarity of mutations was determined in comparisons to *O. punctata* as outgroup. The topology of the studied rice species was based on ML phylogeny reconstructed by complete chloroplast genomes.

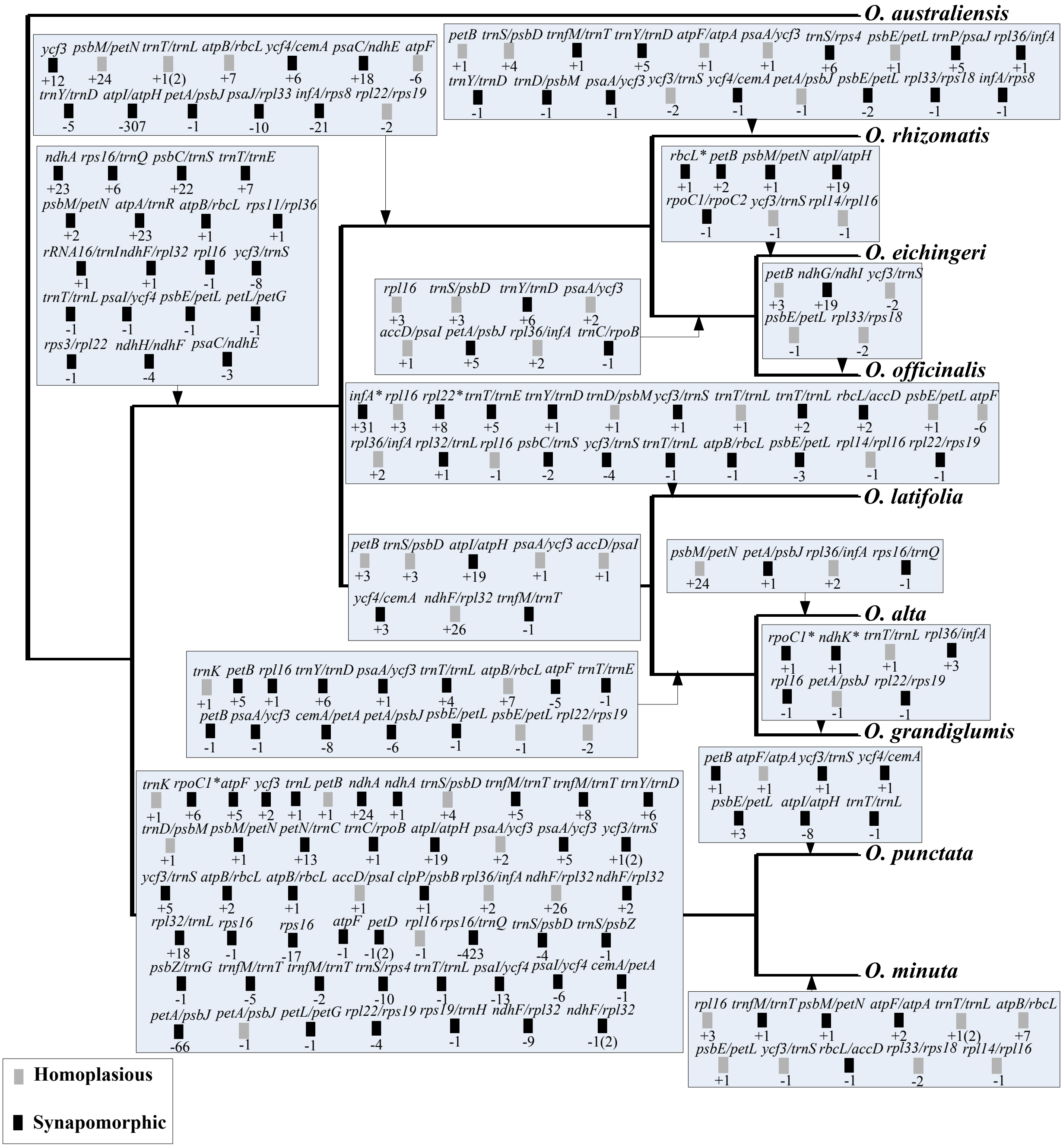

Supplementary Figure 18. Phylogenetic distribution of indels in BB-, CC- and DD-genome *Oryza* species using *O. australiensis* as outgroup. The insertions and deletions of intergenic regions (with '/'), introns and exons (marked as *) are indicated by black and gray bars on each branch. Gene names are given above bars, and sizes of indels (bp) and polarity ('+' = insertion, '-' = deletion) are shown below bars. Numbers within brackets are the frequencies of indels. Polarity of mutations was determined in comparisons to *O. australiensis* as outgroup. The topology of the studied rice species was based on ML phylogeny reconstructed by complete chloroplast genomes.

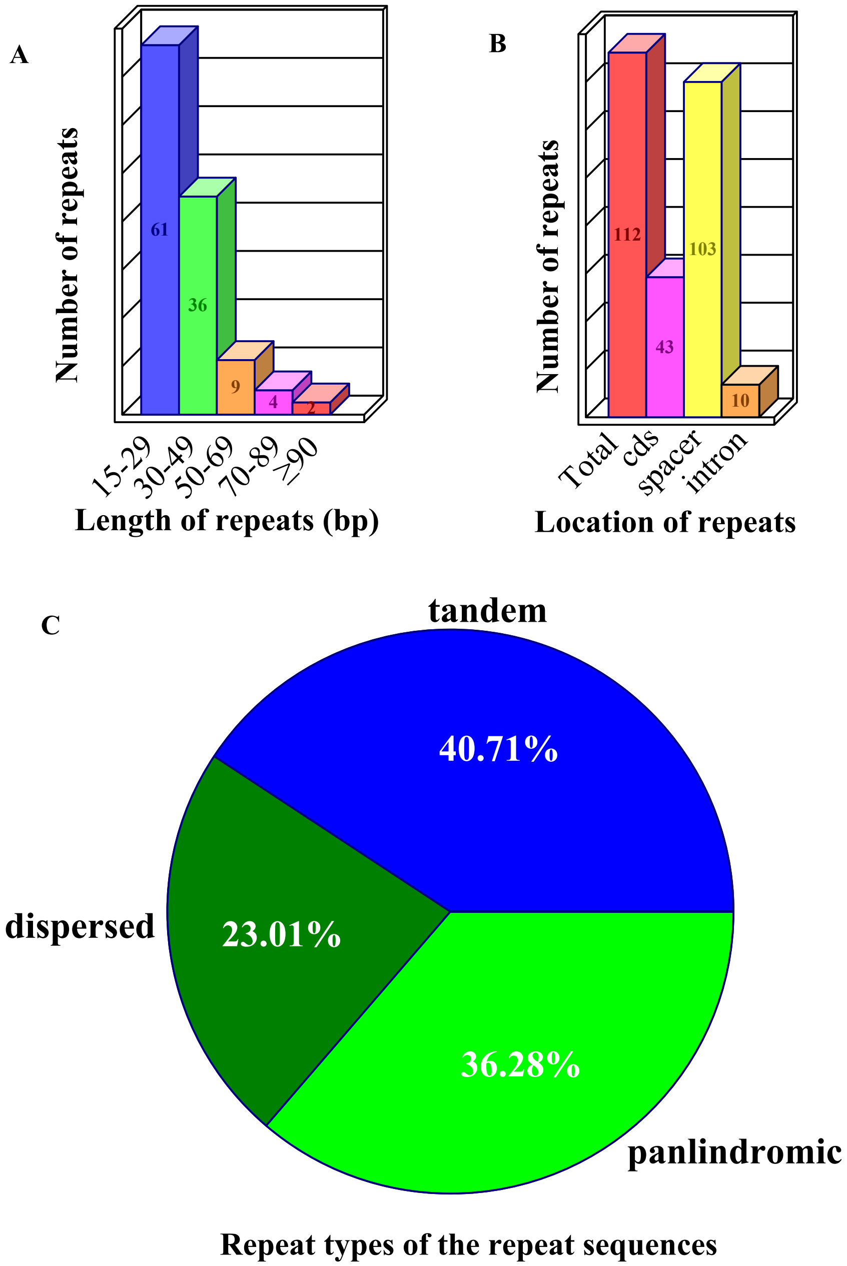

Supplementary Figure 19. Repeat sequences annotated in the 24 Oryzeae chloroplast genomes. (a) number of repeats characterized by sequence lengths; (b) number of repeats based on genomic locations; (c) percentages of the three types of repeat sequences.

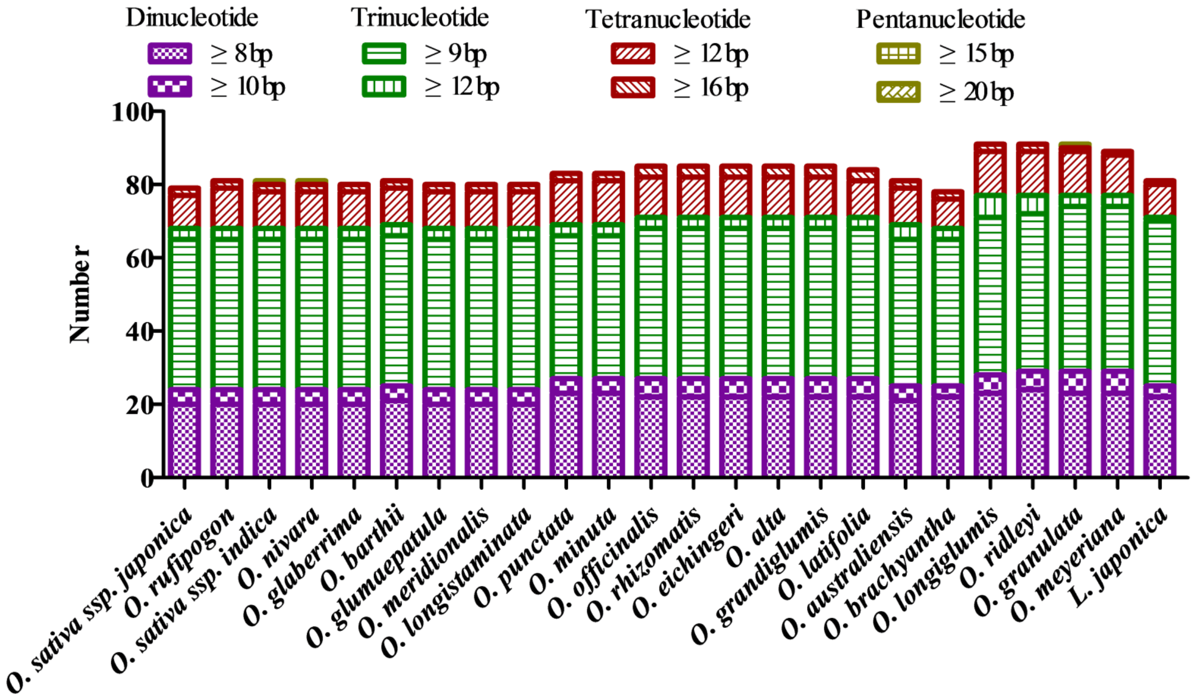

Supplementary Figure 20. Number of different types of SSRs identified among the 24 Oryzeae chloroplast genomes.

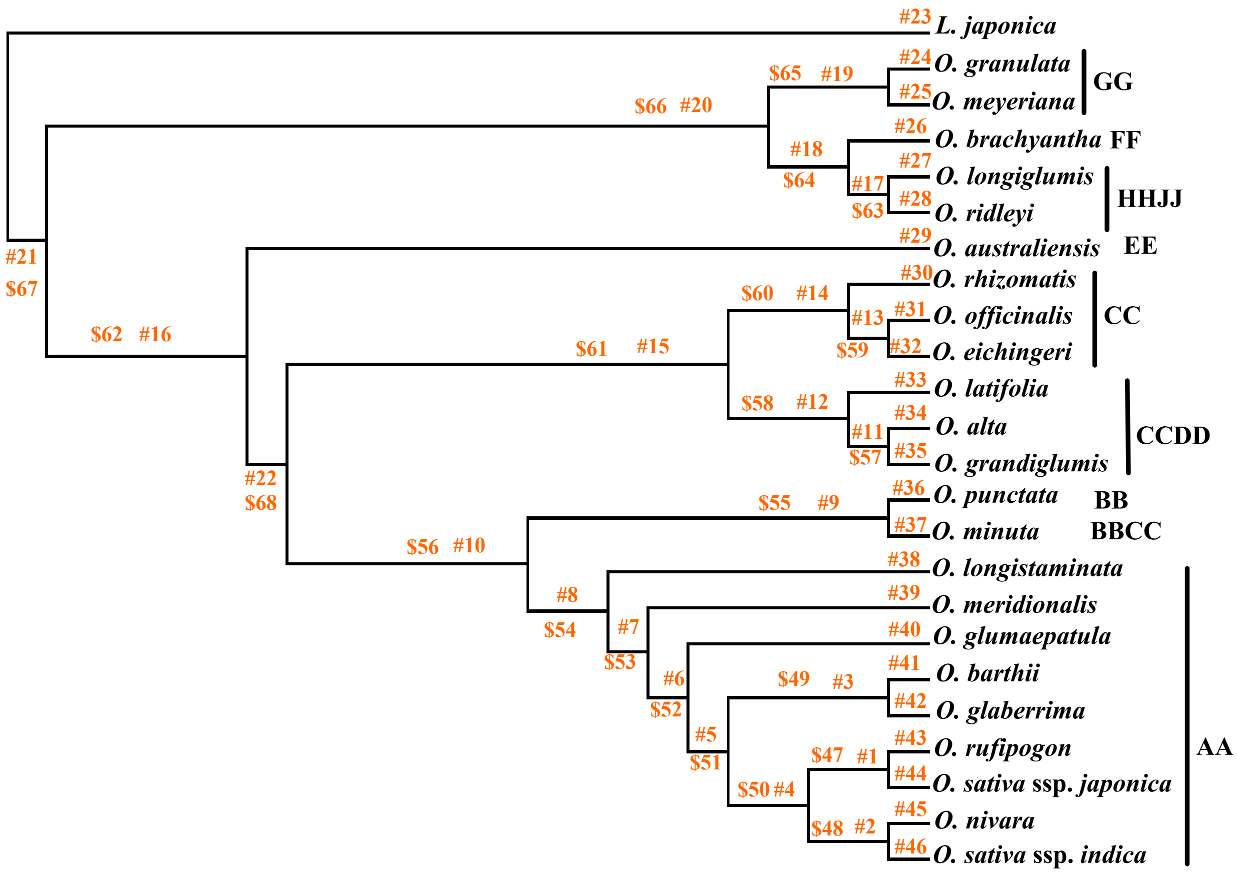

Supplementary Figure 21. The detection of genes under positive selection using the Likelihood Ratio Tests (LRTs) in the *Oryza* phylogeny. Branches under positive selection detected by lineage-specific tests were highlighted in red. The positively selected genes at a significance level of $P < 0.05$ were shown. The topology of the studied rice species was based on ML phylogeny reconstructed by complete chloroplast genomes.

Supplementary Table 1. List of the sequenced rice materials in this study.

**Species**	**Source Country**	**IRGC Acc. No.**	**Category**
O. nivara	India	106104	Wild
O. rufipogon	Yunnan, China	-*	Wild
O. glaberrima	Cameroon	104049	Cultivated
O. barthii	Senegal	101958	Wild
O. glumaepatula	Suriname	100968	Wild
O. meridionalis	Australia	105598	Wild
O. longistaminata	Nigeria	105061	Wild
O. punctata	Kenya	104974	Wild
O. minuta	Philippines	104674	Wild
O. officinalis	Yunnan, China	-*	Wild
O. rhizomatis	Sri Lanka	103417	Wild
O. eichingeri	Uganda	101422	Wild
O. alta	unknown	101395	Wild
O. grandiglumis	Brazil	105664	Wild
O. latifolia	unknown	101392	Wild
O. australiensis	Philippines	80774	Wild
O. brachyantha	Sierra leone	105151	Wild
O. longiglumis	Indonesia	105148	Wild
O. ridleyi	Malaysia	101453	Wild
O. granulata	Yunnan, China	-*	Wild
O. meyeriana	Philippines	106474	Wild
Leersia japonica	Yunnan, China	-*	Wild

*The germplasms were collected by L. Z. Gao and C. Shi.

Supplementary Table 2. Summary of chloroplast genome features of the twenty-one studied *Oryza* species and *L. japonica*.

Species	Genome Type	Genome Size (bp)	Coverage （×）	LSC Length (bp)	SSC Length (bp)	IR Length (bp)	Number of Genes	Protein-coding Genes	Structure RNAs	GC Content （%）	GenBank Accessions
O. nivara	AA	134,556	210	80,604	12,346	20,803	111	77	34	39.00	KF359901#
O. rufipogon	AA	134,587	430	80,636	12,347	20,802	111	77	34	39.00	KF359902*
O. glaberrima	AA	134,587	286	80,598	12,381	20,804	111	77	34	39.00	KF359903#
O. barthii	AA	134,581	66	80,588	12,385	20804	111	77	34	39.01	KF359904*
O. glumaepatula	AA	134,594	775	81,070	12,476	20,839	111	77	34	39.01	KF359905#
O. meridionalis	AA	134,553	407	80,600	12,347	20,803	111	77	34	39.01	KF359906#
O. longistaminata	AA	134,553	45	80,585	12,358	20,807	111	77	34	38.99	KF359907#
O. punctata	BB	134,582	393	80,601	12,387	20,797	111	77	34	38.98	KF359908*
O. minuta	BBCC	134,596	644	80,615	12,387	20,797	111	77	34	38.98	KF359909*
O. officinalis	CC	134,819	463	80,832	12,351	20,818	111	77	34	39.01	KF359910*
O. rhizomatis	CC	134,796	558	80,830	12,330	20,818	111	77	34	39.01	KF359911*
O. eichingeri	CC	134,821	302	80,853	12,330	20,819	111	77	34	39.00	KF359912*
O. alta	CCDD	135,175	2996	81,199	12,338	20,819	111	77	34	39.00	KF359913*
O. grandiglumis	CCDD	135,150	366	81,176	12,338	20,818	111	77	34	38.99	KF359914*
O. latifolia	CCDD	135,190	525	81,211	12,339	20,820	111	77	34	38.99	KF359915*
O. australiensis	EE	135,222	720	81,069	12,475	20,839	111	77	34	38.95	KF359916*
O. brachyantha	FF	134,644	56	80,451	12,529	20,832	111	77	34	38.98	KF359917$
O. longiglumis	HHJJ	135,641	256	81,622	12,485	20,767	111	77	34	38.93	KF359918*
O. ridleyi	HHJJ	135,731	344	81,603	12,494	20,817	111	77	34	38.92	KF359919*
O. granulata	GG	135,942	349	81,843	12,499	20,801	111	77	34	38.96	KF359920*
O. meyeriana	GG	136,133	3208	81,832	12,499	20,901	111	77	34	38.94	KF359921*
L. japonica	ND	134,074	358	81,225	12,449	20,200	111	77	34	38.92	KF359922$

Using high quality sequence reads extracted from whole genome Illumina repository, we assembled the five chloroplast genome sequences from the five wild rice species; * The genomes were directly sequenced by using PCR amplification products of the chloroplast DNA; $ The genomes were directly sequenced by using isolated chloroplast DNA； ND: Not Determine

Supplementary Table 3. Description of sequences and best-supported models of molecular evolution.

Characteristic	Results
Aligned length (bp)	139,206
Variable sites (%)	5,917 (4.25 %)
Informative sites (%)	2,766 (1.99%)
freqA	0.3087
freqC	0.1923
freqG	0.1934
freqT	0.3056
-ln*L*	239807.4062
Model	TVM+I+G
Ti/Tv	1.5749

Supplementary Table 4. Statistic of genomic variation events detected in the LSC, SSC and IR regions across the *Oryza* chloroplast genomes using *L. japonica* as outgroup.

**Species**	**SNP**				**Deletion**				**Insertion**			
	**LSC**	**SSC**	**IR**	**Total**	**LSC**	**SSC**	**IR**	**Total**	**LSC**	**SSC**	**IR**	**Total**
O. sativa ssp. *japonica*	1942	342	87	2371	156	21	20	197	156	22	15	193
O. rufipogon	1864	339	69	2272	134	17	10	161	135	22	9	166
O. sativa ssp. *indica*	1869	337	65	2273	134	17	10	161	136	22	9	167
O. nivara	1874	340	65	2279	135	17	10	162	137	21	10	168
O. glaberrima	1875	338	68	2281	134	17	10	161	133	22	9	164
O. barthii	1877	338	68	2283	134	17	10	161	134	23	9	166
O. glumaepatula	1855	334	68	2257	133	18	10	161	132	22	9	163
O. meridionalis	1875	340	69	2284	135	17	9	161	135	22	9	166
O. longistaminata	1867	336	68	2271	135	17	9	161	132	24	10	166
O. punctata	1820	340	69	2229	124	16	9	148	144	24	6	174
O. minuta	1836	337	73	2246	127	16	9	152	148	24	6	178
O. officinalis	1733	322	62	2117	123	16	8	147	135	24	8	167
O. rhizomatis	1765	323	63	2151	121	16	8	145	134	23	8	165
O. eichingeri	1746	320	62	2128	124	16	8	148	138	23	8	169
O. alta	1730	315	61	2106	121	16	8	145	135	22	8	165
O. grandiglumis	1730	314	61	2105	120	16	8	144	137	22	8	167
O. latifolia	1736	313	58	2107	121	16	8	145	137	23	8	168
O. australiensis	1799	342	61	2202	125	21	9	155	128	23	8	159
O. brachyantha	1936	377	67	2380	159	17	5	181	156	25	9	190
O. longiglumis	1734	328	65	2127	128	13	8	149	141	27	8	176
O. ridleyi	1720	298	65	2083	124	14	7	145	138	27	8	173
O. granulata	1554	280	60	1894	123	16	6	145	143	19	7	169
O. meyeriana	1552	281	59	1892	121	15	6	142	138	19	8	165

Supplementary Table 5. Statistic of genomic variation events of every 1 Kb detected in the LSC, SSC and IR regions across the *Oryza* chloroplast genomes using *L. japonica* as outgroup.

**Species**	**SNP**				**Deletion**				**Insertion**			
	**LSC**	**SSC**	**IR**	**Total**	**LSC**	**SSC**	**IR**	**Total**	**LSC**	**SSC**	**IR**	**Total**
O. sativa ssp. *japonica*	24.10	27.73	4.18	17.62	1.94	1.70	0.96	1.46	1.94	1.78	0.72	1.43
O. rufipogon	23.12	27.46	3.32	16.88	1.66	1.38	0.48	1.20	1.67	1.78	0.43	1.23
O. sativa ssp. *indica*	23.20	27.29	3.13	16.90	1.66	1.38	0.48	1.20	1.69	1.78	0.43	1.24
O. nivara	23.25	27.54	3.12	16.94	1.67	1.38	0.48	1.20	1.70	1.70	0.48	1.25
O. glaberrima	23.26	27.30	3.27	16.95	1.66	1.37	0.48	1.20	1.65	1.78	0.43	1.22
O. barthii	23.29	27.29	3.27	16.96	1.66	1.37	0.48	1.20	1.66	1.86	0.43	1.23
O. glumaepatula	22.88	26.77	3.26	16.69	1.64	1.44	0.48	1.19	1.63	1.76	0.43	1.21
O. meridionalis	23.26	27.54	3.32	16.97	1.67	1.38	0.43	1.20	1.67	1.78	0.43	1.23
O. longistaminata	23.17	27.19	3.27	16.88	1.68	1.38	0.43	1.20	1.64	1.94	0.48	1.23
O. punctata	22.58	27.45	3.32	16.56	1.54	1.29	0.43	1.10	1.79	1.94	0.29	1.29
O. minuta	22.77	27.21	3.51	16.69	1.58	1.29	0.43	1.13	1.84	1.94	0.29	1.32
O. officinalis	21.44	26.07	2.98	15.70	1.52	1.30	0.38	1.09	1.67	1.94	0.38	1.24
O. rhizomatis	21.84	26.20	3.03	15.96	1.50	1.30	0.38	1.08	1.66	1.87	0.38	1.22
O. eichingeri	21.59	25.95	2.98	15.78	1.53	1.30	0.38	1.10	1.71	1.87	0.38	1.25
O. alta	21.31	25.53	2.93	15.58	1.49	1.30	0.38	1.07	1.66	1.78	0.38	1.22
O. grandiglumis	21.31	25.45	2.93	15.58	1.48	1.30	0.38	1.07	1.69	1.78	0.38	1.24
O. latifolia	21.38	25.37	2.79	15.59	1.49	1.30	0.38	1.07	1.69	1.86	0.38	1.24
O. australiensis	22.19	27.41	2.93	16.28	1.54	1.68	0.43	1.15	1.58	1.84	0.38	1.18
O. brachyantha	24.06	30.09	3.22	17.67	1.98	1.36	0.24	1.34	1.94	2.00	0.43	1.41
O. longiglumis	21.24	26.27	3.13	15.68	1.57	1.04	0.39	1.10	1.73	2.16	0.39	1.30
O. ridleyi	21.08	23.85	3.12	15.35	1.52	1.12	0.34	1.07	1.69	2.16	0.38	1.27
O. granulata	18.99	22.40	2.88	13.93	1.50	1.28	0.29	1.07	1.75	1.52	0.34	1.24
O. meyeriana	18.97	22.48	2.82	13.90	1.48	1.20	0.29	1.04	1.69	1.52	0.38	1.21
Average	22.19	26.43	3.16	16.22	1.61	1.34	0.43	1.15	1.71	1.84	0.41	1.26

Supplementary Table 6a. Statistics of insertions for the twenty-three *Oryza* plastomes using the *L. japonica* genome as outgroup.

Branch*	Start**	End**	Feature	Length (bp)
O.granulata	11960	11960	insertion	1
O.granulata	17007	17007	insertion	1
O.granulata	68630	68630	insertion	1
O.granulata	72232	72236	insertion	5
O.granulata	78781	78781	insertion	1
O.granulata	83336	83336	insertion	1
O.meyeriana	4472	4472	insertion	1
O.meyeriana	15146	15146	insertion	1
O.meyeriana	57799	57799	insertion	1
O.meyeriana	81437	81437	insertion	1
O.meyeriana	84844	84844	insertion	1
O.meyeriana	90708	90807	insertion	100
O.meyeriana	137344	137443	insertion	100
O.brachyantha	3254	3259	insertion	6
O.brachyantha	3417	3417	insertion	1
O.brachyantha	3458	3463	insertion	6
O.brachyantha	3580	3600	insertion	21
O.brachyantha	3743	3748	insertion	6
O.brachyantha	7027	7068	insertion	42
O.brachyantha	7823	7836	insertion	14
O.brachyantha	9022	9028	insertion	7
O.brachyantha	9308	9314	insertion	7
O.brachyantha	9354	9358	insertion	5
O.brachyantha	12457	12458	insertion	2
O.brachyantha	13328	13331	insertion	4
O.brachyantha	14312	14322	insertion	11
O.brachyantha	16232	16237	insertion	6
O.brachyantha	18382	18385	insertion	4
O.brachyantha	18906	18907	insertion	2
O.brachyantha	19499	19499	insertion	1
O.brachyantha	19604	19608	insertion	5
O.brachyantha	19693	19694	insertion	2
O.brachyantha	19707	19713	insertion	7
O.brachyantha	19823	19829	insertion	7
O.brachyantha	21328	21332	insertion	5
O.brachyantha	34978	34981	insertion	4
O.brachyantha	36727	36727	insertion	1
O.brachyantha	37068	37071	insertion	4
O.brachyantha	39341	39347	insertion	7
O.brachyantha	39832	39836	insertion	5
O.brachyantha	47289	47289	insertion	1
O.brachyantha	47790	47790	insertion	1
O.brachyantha	48361	48381	insertion	21
O.brachyantha	49927	49927	insertion	1

O.brachyantha	50995	51000	insertion	6
O.brachyantha	51296	51302	insertion	7
O.brachyantha	54347	54347	insertion	1
O.brachyantha	55481	55486	insertion	6
O.brachyantha	58196	58200	insertion	5
O.brachyantha	58557	58557	insertion	1
O.brachyantha	60400	60404	insertion	5
O.brachyantha	60705	60709	insertion	5
O.brachyantha	61499	61499	insertion	1
O.brachyantha	66083	66086	insertion	4
O.brachyantha	66416	66432	insertion	17
O.brachyantha	68011	68015	insertion	5
O.brachyantha	68365	68368	insertion	4
O.brachyantha	69107	69112	insertion	6
O.brachyantha	69960	69964	insertion	5
O.brachyantha	70688	70688	insertion	1
O.brachyantha	71531	71531	insertion	1
O.brachyantha	73776	73779	insertion	4
O.brachyantha	74037	74037	insertion	1
O.brachyantha	74113	74118	insertion	6
O.brachyantha	78678	78680	insertion	3
O.brachyantha	79822	79823	insertion	2
O.brachyantha	81387	81407	insertion	21
O.brachyantha	82139	82139	insertion	1
O.brachyantha	84189	84197	insertion	9
O.brachyantha	86373	86378	insertion	6
O.brachyantha	96078	96083	insertion	6
O.brachyantha	96144	96144	insertion	1
O.brachyantha	99358	99358	insertion	1
O.brachyantha	107130	107163	insertion	34
O.brachyantha	110064	110070	insertion	7
O.brachyantha	111068	111068	insertion	1
O.brachyantha	111289	111313	insertion	25
O.brachyantha	111437	111437	insertion	1
O.brachyantha	116621	116649	insertion	29
O.brachyantha	116702	116712	insertion	11
O.brachyantha	116791	116817	insertion	27
O.brachyantha	116879	116885	insertion	7
O.brachyantha	118974	118974	insertion	1
O.brachyantha	120883	120916	insertion	34
O.brachyantha	128708	128708	insertion	1
O.brachyantha	131922	131922	insertion	1
O.brachyantha	131987	131992	insertion	6
O.brachyantha	141686	141691	insertion	6
O.longiglumis	13214	13214	insertion	1
O.longiglumis	13385	13389	insertion	5
O.longiglumis	29856	29876	insertion	21

O.longiglumis	34821	34821	insertion	1
O.longiglumis	36092	36092	insertion	1
O.longiglumis	50986	50986	insertion	1
O.longiglumis	51243	51243	insertion	1
O.longiglumis	60236	60236	insertion	1
O.longiglumis	61868	61868	insertion	1
O.longiglumis	65787	65792	insertion	6
O.longiglumis	67669	67669	insertion	1
O.longiglumis	68623	68623	insertion	1
O.longiglumis	69352	69353	insertion	2
O.longiglumis	84850	84851	insertion	2
O.longiglumis	110848	110848	insertion	1
O.longiglumis	110874	110874	insertion	1
O.longiglumis	113968	113984	insertion	17
O.longiglumis	114204	114219	insertion	16
O.ridleyi	5493	5493	insertion	1
O.ridleyi	22140	22140	insertion	1
O.ridleyi	34601	34601	insertion	1
O.ridleyi	40308	40308	insertion	1
O.ridleyi	44919	44920	insertion	2
O.ridleyi	54279	54283	insertion	5
O.ridleyi	70950	70950	insertion	1
O.ridleyi	110756	110761	insertion	6
O.ridleyi	116583	116588	insertion	6
O.australiensis	4468	4468	insertion	1
O.australiensis	5126	5129	insertion	4
O.australiensis	6667	6668	insertion	2
O.australiensis	8523	8523	insertion	1
O.australiensis	8733	8733	insertion	1
O.australiensis	16057	16062	insertion	6
O.australiensis	16855	16859	insertion	5
O.australiensis	18093	18094	insertion	2
O.australiensis	19963	20000	insertion	38
O.australiensis	30148	30180	insertion	33
O.australiensis	47524	47524	insertion	1
O.australiensis	47980	47985	insertion	6
O.australiensis	54069	54069	insertion	1
O.australiensis	60246	60248	insertion	3
O.australiensis	60445	60447	insertion	3
O.australiensis	61910	61910	insertion	1
O.australiensis	65622	65622	insertion	1
O.australiensis	65834	65839	insertion	6
O.australiensis	69098	69106	insertion	9
O.australiensis	70011	70017	insertion	7
O.australiensis	73862	73869	insertion	8
O.australiensis	85803	85804	insertion	2
O.australiensis	90017	90050	insertion	34

O.australiensis	111229	111231	insertion	3
O.australiensis	113139	113144	insertion	6
O.australiensis	115160	115164	insertion	5
O.australiensis	138068	138101	insertion	34
O.australiensis	141734	141735	insertion	2
O.rhizomatis	9090	9090	insertion	1
O.rhizomatis	15517	15517	insertion	1
O.rhizomatis	37666	37666	insertion	1
O.rhizomatis	48459	48464	insertion	6
O.rhizomatis	69751	69755	insertion	5
O.officinalis	60260	60266	insertion	7
O.officinalis	76864	76864	insertion	1
O.officinalis	116664	116682	insertion	19
O.eichingeri	19944	19944	insertion	1
O.eichingeri	34702	34720	insertion	19
O.eichingeri	47532	47532	insertion	1
O.eichingeri	68364	68364	insertion	1
O.latifolia	17065	17069	insertion	5
O.latifolia	18961	18961	insertion	1
O.latifolia	47767	47767	insertion	1
O.latifolia	60221	60227	insertion	7
O.latifolia	60630	60631	insertion	2
O.latifolia	76859	76859	insertion	1
O.latifolia	82154	82184	insertion	31
O.latifolia	85857	85864	insertion	8
O.latifolia	86346	86346	insertion	1
O.latifolia	111073	111073	insertion	1
O.latifolia	141725	141725	insertion	1
O.alta	19263	19286	insertion	24
O.alta	112365	112366	insertion	2
O.grandiglumis	47533	47533	insertion	1
O.grandiglumis	81789	81789	insertion	1
O.punctata	47520	47521	insertion	2
O.punctata	50705	50709	insertion	5
O.punctata	60239	60244	insertion	6
O.punctata	63189	63189	insertion	1
O.punctata	65781	65786	insertion	6
O.punctata	68628	68629	insertion	2
O.punctata	70942	70943	insertion	2
O.punctata	77254	77254	insertion	1
O.punctata	83344	83344	insertion	1
O.minuta	16046	16046	insertion	1
O.minuta	19316	19316	insertion	1
O.minuta	49921	49921	insertion	1
O.minuta	50015	50016	insertion	2
O.minuta	57882	57888	insertion	7
O.longistaminata	21108	21109	insertion	2

O.longistaminata	33637	33637	insertion	1
O.longistaminata	95596	95596	insertion	1
O.longistaminata	105869	105870	insertion	2
O.longistaminata	109934	109938	insertion	5
O.longistaminata	111778	111782	insertion	5
O.longistaminata	114055	114055	insertion	1
O.longistaminata	122194	122195	insertion	2
O.longistaminata	132469	132469	insertion	1
O.meridionalis	3618	3618	insertion	1
O.meridionalis	6506	6506	insertion	1
O.meridionalis	8499	8503	insertion	5
O.meridionalis	8710	8714	insertion	5
O.meridionalis	12043	12043	insertion	1
O.meridionalis	22188	22190	insertion	3
O.meridionalis	45032	45032	insertion	1
O.meridionalis	54747	54751	insertion	5
O.meridionalis	76858	76858	insertion	1
O.meridionalis	84078	84078	insertion	1
O.glumaepatula	4297	4302	insertion	6
O.glumaepatula	4368	4368	insertion	1
O.glumaepatula	4466	4466	insertion	1
O.glumaepatula	5036	5085	insertion	50
O.glumaepatula	5248	5248	insertion	1
O.glumaepatula	5491	5491	insertion	1
O.glumaepatula	6665	6666	insertion	2
O.glumaepatula	8520	8520	insertion	1
O.glumaepatula	8730	8731	insertion	2
O.glumaepatula	9075	9077	insertion	3
O.glumaepatula	9388	9388	insertion	1
O.glumaepatula	9409	9412	insertion	4
O.glumaepatula	13219	13219	insertion	1
O.glumaepatula	14891	15124	insertion	234
O.glumaepatula	15447	15451	insertion	5
O.glumaepatula	15514	15514	insertion	1
O.glumaepatula	16116	16125	insertion	10
O.glumaepatula	16303	16308	insertion	6
O.glumaepatula	16863	16866	insertion	4
O.glumaepatula	17613	17613	insertion	1
O.glumaepatula	18096	18097	insertion	2
O.glumaepatula	20005	20042	insertion	38
O.glumaepatula	21582	21603	insertion	22
O.glumaepatula	22106	22108	insertion	3
O.glumaepatula	27916	27916	insertion	1
O.glumaepatula	30115	30147	insertion	33
O.glumaepatula	32665	32666	insertion	2
O.glumaepatula	35036	35052	insertion	17
O.glumaepatula	36776	36776	insertion	1

O.glumaepatula	37050	37051	insertion	2
O.glumaepatula	37589	37592	insertion	4
O.glumaepatula	47685	47687	insertion	3
O.glumaepatula	47970	47975	insertion	6
O.glumaepatula	49835	49835	insertion	1
O.glumaepatula	50033	50033	insertion	1
O.glumaepatula	51084	51149	insertion	66
O.glumaepatula	51394	51426	insertion	33
O.glumaepatula	54068	54068	insertion	1
O.glumaepatula	54393	54477	insertion	85
O.glumaepatula	60055	60056	insertion	2
O.glumaepatula	60209	60213	insertion	5
O.glumaepatula	60440	60442	insertion	3
O.glumaepatula	61436	61498	insertion	63
O.glumaepatula	61917	61917	insertion	1
O.glumaepatula	62397	62398	insertion	2
O.glumaepatula	62560	62565	insertion	6
O.glumaepatula	63328	63332	insertion	5
O.glumaepatula	63567	63584	insertion	18
O.glumaepatula	65621	65621	insertion	1
O.glumaepatula	65846	65851	insertion	6
O.glumaepatula	66088	66088	insertion	1
O.glumaepatula	68620	68620	insertion	1
O.glumaepatula	68838	68838	insertion	1
O.glumaepatula	69080	69088	insertion	9
O.glumaepatula	69991	70004	insertion	14
O.glumaepatula	70204	70208	insertion	5
O.glumaepatula	70828	70845	insertion	18
O.glumaepatula	70949	70949	insertion	1
O.glumaepatula	73843	73861	insertion	19
O.glumaepatula	78668	78668	insertion	1
O.glumaepatula	82202	82202	insertion	1
O.glumaepatula	83327	83327	insertion	1
O.glumaepatula	84668	84677	insertion	10
O.glumaepatula	84875	84875	insertion	1
O.glumaepatula	89982	90015	insertion	34
O.glumaepatula	99361	99361	insertion	1
O.glumaepatula	105287	105287	insertion	1
O.glumaepatula	108883	108892	insertion	10
O.glumaepatula	109868	109868	insertion	1
O.glumaepatula	110113	110259	insertion	147
O.glumaepatula	110441	110449	insertion	9
O.glumaepatula	111220	111223	insertion	4
O.glumaepatula	111469	111472	insertion	4
O.glumaepatula	112359	112360	insertion	2
O.glumaepatula	113129	113134	insertion	6
O.glumaepatula	115154	115158	insertion	5

O.glumaepatula	115242	115245	insertion	4
O.glumaepatula	115347	115350	insertion	4
O.glumaepatula	122778	122778	insertion	1
O.glumaepatula	128712	128712	insertion	1
O.glumaepatula	138024	138057	insertion	34
O.glumaepatula	141727	141728	insertion	2
O.glaberrima	4897	4897	insertion	1
O.glaberrima	4990	5035	insertion	46
O.glaberrima	44932	44932	insertion	1
O.glaberrima	55513	55527	insertion	15
O.glaberrima	55559	55571	insertion	13
O.glaberrima	66293	66293	insertion	1
O.glaberrima	66582	66582	insertion	1
O.glaberrima	70728	70740	insertion	13
O.glaberrima	77293	77293	insertion	1
O.glaberrima	99516	99516	insertion	1
O.glaberrima	128554	128554	insertion	1
O.barthii	49828	49831	insertion	4
O.barthii	116698	116701	insertion	4
O.nivara	4376	4376	insertion	1
O.nivara	4944	4989	insertion	46
O.nivara	5251	5251	insertion	1
O.nivara	6868	6873	insertion	6
O.nivara	8729	8729	insertion	1
O.nivara	9387	9387	insertion	1
O.nivara	9405	9408	insertion	4
O.nivara	12049	12049	insertion	1
O.nivara	12118	12139	insertion	22
O.nivara	13218	13218	insertion	1
O.nivara	14656	14889	insertion	234
O.nivara	15436	15440	insertion	5
O.nivara	15519	15519	insertion	1
O.nivara	16313	16318	insertion	6
O.nivara	17080	17086	insertion	7
O.nivara	17629	17638	insertion	10
O.nivara	18136	18136	insertion	1
O.nivara	19236	19259	insertion	24
O.nivara	19500	19500	insertion	1
O.nivara	19943	19943	insertion	1
O.nivara	21560	21581	insertion	22
O.nivara	34733	34751	insertion	19
O.nivara	36774	36774	insertion	1
O.nivara	37048	37048	insertion	1
O.nivara	39264	39286	insertion	23
O.nivara	44928	44928	insertion	1
O.nivara	45826	45837	insertion	12
O.nivara	51018	51083	insertion	66

O.nivara	51361	51393	insertion	33
O.nivara	54479	54563	insertion	85
O.nivara	57796	57796	insertion	1
O.nivara	68632	68633	insertion	2
O.nivara	68840	68840	insertion	1
O.nivara	70214	70218	insertion	5
O.nivara	70354	70374	insertion	21
O.nivara	72258	72276	insertion	19
O.nivara	76854	76854	insertion	1
O.nivara	76994	76994	insertion	1
O.nivara	77208	77210	insertion	3
O.nivara	79263	79263	insertion	1
O.nivara	81434	81434	insertion	1
O.nivara	82209	82209	insertion	1
O.nivara	84660	84667	insertion	8
O.nivara	86328	86330	insertion	3
O.nivara	99513	99513	insertion	1
O.nivara	105292	105292	insertion	1
O.nivara	107441	107456	insertion	16
O.nivara	109806	109806	insertion	1
O.nivara	109863	109863	insertion	1
O.nivara	110431	110439	insertion	9
O.nivara	111332	111332	insertion	1
O.nivara	111465	111468	insertion	4
O.nivara	115001	115018	insertion	18
O.nivara	122775	122775	insertion	1
O.nivara	128553	128553	insertion	1
O.sativa_ssp.indica	5393	5399	insertion	7
O.sativa_ssp.indica	18963	18963	insertion	1
O.sativa_ssp.indica	60254	60259	insertion	6
O.sativa_ssp.indica	62505	62509	insertion	5
O.sativa_ssp.indica	65841	65845	insertion	5
O.sativa_ssp.indica	67559	67566	insertion	8
O.sativa_ssp.indica	83338	83340	insertion	3
O.rufipogon	4259	4284	insertion	26
O.rufipogon	11997	12000	insertion	4
O.rufipogon	70939	70939	insertion	1
O.sativa_ssp.japon	15531	15532	insertion	2
O.sativa_ssp.japon	16631	16631	insertion	1
O.sativa_ssp.japon	21819	21819	insertion	1
O.sativa_ssp.japon	36135	36135	insertion	1
O.sativa_ssp.japon	45972	45972	insertion	1
O.sativa_ssp.japon	46193	46193	insertion	1
O.sativa_ssp.japon	46268	46268	insertion	1
O.sativa_ssp.japon	46289	46289	insertion	1
O.sativa_ssp.japon	46293	46293	insertion	1
O.sativa_ssp.japon	46315	46315	insertion	1

O.sativa_ssp.japon	46744	46744	insertion	1
O.sativa_ssp.japon	47245	47245	insertion	1
O.sativa_ssp.japon	47261	47261	insertion	1
O.sativa_ssp.japon	47269	47269	insertion	1
O.sativa_ssp.japon	47277	47277	insertion	1
O.sativa_ssp.japon	47557	47557	insertion	1
O.sativa_ssp.japon	47743	47743	insertion	1
O.sativa_ssp.japon	49912	49912	insertion	1
O.sativa_ssp.japon	54859	54859	insertion	1
O.sativa_ssp.japon	57864	57864	insertion	1
O.sativa_ssp.japon	58214	58214	insertion	1
O.sativa_ssp.japon	58333	58333	insertion	1
O.sativa_ssp.japon	60215	60220	insertion	6
O.sativa_ssp.japon	61881	61896	insertion	16
O.sativa_ssp.japon	61912	61912	insertion	1
O.sativa_ssp.japon	61985	61985	insertion	1
O.sativa_ssp.japon	64529	64529	insertion	1
O.sativa_ssp.japon	66579	66579	insertion	1
O.sativa_ssp.japon	72248	72248	insertion	1
O.sativa_ssp.japon	73804	73804	insertion	1
O.sativa_ssp.japon	73892	73892	insertion	1
O.sativa_ssp.japon	77294	77294	insertion	1
O.sativa_ssp.japon	78364	78364	insertion	1
O.sativa_ssp.japon	79061	79061	insertion	1
O.sativa_ssp.japon	81793	81793	insertion	1
O.sativa_ssp.japon	82137	82137	insertion	1
O.sativa_ssp.japon	83852	83852	insertion	1
O.sativa_ssp.japon	85782	85782	insertion	1
O.sativa_ssp.japon	93592	93593	insertion	2
O.sativa_ssp.japon	93614	93615	insertion	2
O.sativa_ssp.japon	93623	93623	insertion	1
O.sativa_ssp.japon	93734	93734	insertion	1
O.sativa_ssp.japon	95330	95330	insertion	1
O.sativa_ssp.japon	96516	96516	insertion	1
O.sativa_ssp.japon	100490	100490	insertion	1
O.sativa_ssp.japon	103505	103505	insertion	1
O.sativa_ssp.japon	107885	107885	insertion	1
O.sativa_ssp.japon	112357	112358	insertion	2
O.sativa_ssp.japon	124561	124561	insertion	1
O.sativa_ssp.japon	127576	127576	insertion	1
O.sativa_ssp.japon	131550	131550	insertion	1
O.sativa_ssp.japon	132735	132735	insertion	1
O.sativa_ssp.japon	134331	134331	insertion	1
O.sativa_ssp.japon	134441	134441	insertion	1
O.sativa_ssp.japon	134451	134452	insertion	2
O.sativa_ssp.japon	134469	134470	insertion	2
#1#	3621	3629	insertion	9

#1#	4469	4471	insertion	3
#1#	4577	4581	insertion	5
#1#	4894	4894	insertion	1
#1#	5250	5250	insertion	1
#1#	6500	6500	insertion	1
#1#	6782	6798	insertion	17
#1#	14515	14515	insertion	1
#1#	17616	17616	insertion	1
#1#	18958	18958	insertion	1
#1#	20505	20505	insertion	1
#1#	21865	21865	insertion	1
#1#	32562	32564	insertion	3
#1#	33581	33584	insertion	4
#1#	34764	34765	insertion	2
#1#	34824	34824	insertion	1
#1#	36414	36414	insertion	1
#1#	36801	36801	insertion	1
#1#	40310	40310	insertion	1
#1#	44927	44927	insertion	1
#1#	45095	45096	insertion	2
#1#	48084	48084	insertion	1
#1#	49232	49237	insertion	6
#1#	51178	51181	insertion	4
#1#	55535	55554	insertion	20
#1#	58042	58042	insertion	1
#1#	60252	60252	insertion	1
#1#	60354	60362	insertion	9
#1#	60632	60632	insertion	1
#1#	61508	61513	insertion	6
#1#	65799	65804	insertion	6
#1#	67679	67683	insertion	5
#1#	71572	71580	insertion	9
#1#	73870	73888	insertion	19
#1#	80928	80928	insertion	1
#1#	81438	81438	insertion	1
#1#	81794	81794	insertion	1
#1#	83933	83933	insertion	1
#1#	84213	84216	insertion	4
#1#	85806	85806	insertion	1
#1#	109851	109858	insertion	8
#1#	118913	118913	insertion	1
#2#	3270	3271	insertion	2
#2#	3305	3306	insertion	2
#2#	5721	5721	insertion	1
#2#	9182	9188	insertion	7
#2#	12863	12863	insertion	1
#2#	13029	13034	insertion	6

#2#	13397	13397	insertion	1
#2#	13685	13689	insertion	5
#2#	15508	15508	insertion	1
#2#	16450	16450	insertion	1
#2#	16765	16822	insertion	58
#2#	19585	19586	insertion	2
#2#	22142	22142	insertion	1
#2#	34814	34815	insertion	2
#2#	35834	35839	insertion	6
#2#	37042	37042	insertion	1
#2#	45064	45064	insertion	1
#2#	45801	45805	insertion	5
#2#	49852	49859	insertion	8
#2#	60058	60060	insertion	3
#2#	60228	60235	insertion	8
#2#	60542	60542	insertion	1
#2#	61823	61823	insertion	1
#2#	70894	70899	insertion	6
#2#	70933	70934	insertion	2
#2#	71500	71505	insertion	6
#2#	71725	71738	insertion	14
#2#	81730	81730	insertion	1
#2#	86333	86333	insertion	1
#2#	96833	96859	insertion	27
#2#	105606	105606	insertion	1
#2#	107433	107438	insertion	6
#2#	107520	107525	insertion	6
#2#	110640	110645	insertion	6
#2#	115439	115446	insertion	8
#2#	122460	122460	insertion	1
#2#	131184	131210	insertion	27
#2#	141720	141720	insertion	1
#9#	1584	1588	insertion	5
#9#	1670	1695	insertion	26
#9#	4346	4346	insertion	1
#9#	6721	6721	insertion	1
#9#	8784	8784	insertion	1
#9#	9159	9159	insertion	1
#9#	13241	13241	insertion	1
#9#	14140	14145	insertion	6
#9#	16904	16908	insertion	5
#9#	16928	16928	insertion	1
#9#	18142	18146	insertion	5
#9#	18918	18918	insertion	1
#9#	21809	21809	insertion	1
#9#	22139	22139	insertion	1
#9#	32828	32828	insertion	1

#9#	46242	46243	insertion	2
#9#	55589	55593	insertion	5
#9#	60061	60067	insertion	7
#9#	60455	60463	insertion	9
#9#	61871	61871	insertion	1
#9#	65931	65935	insertion	5
#9#	73959	73963	insertion	5
#9#	84074	84075	insertion	2
#9#	84077	84077	insertion	1
#9#	110050	110059	insertion	10
#9#	110513	110516	insertion	4
#9#	112981	112981	insertion	1
#9#	118503	118505	insertion	3
#13#	6497	6497	insertion	1
#13#	6744	6748	insertion	5
#13#	9158	9158	insertion	1
#13#	16429	16433	insertion	5
#13#	16726	16726	insertion	1
#13#	17106	17108	insertion	3
#13#	21357	21357	insertion	1
#13#	49238	49241	insertion	4
#13#	49874	49874	insertion	1
#13#	51585	51585	insertion	1
#13#	53962	53965	insertion	4
#13#	54745	54745	insertion	1
#13#	55573	55585	insertion	13
#13#	63229	63229	insertion	1
#13#	76920	76921	insertion	2
#13#	77318	77324	insertion	7
#13#	83309	83309	insertion	1
#13#	83347	83348	insertion	2
#13#	83909	83915	insertion	7
#13#	84079	84083	insertion	5
#13#	84212	84212	insertion	1
#13#	86744	86744	insertion	1
#13#	107109	107109	insertion	1
#13#	112923	112923	insertion	1
#13#	115339	115341	insertion	3
#13#	118506	118506	insertion	1
#13#	120950	120950	insertion	1
#13#	141322	141322	insertion	1
#3#	44929	44929	insertion	1
#3#	65725	65729	insertion	5
#10#	15505	15505	insertion	1
#10#	17639	17643	insertion	5
#10#	19211	19234	insertion	24
#10#	45839	45850	insertion	12

#10#	63191	63193	insertion	3
#10#	84648	84649	insertion	2
#10#	114981	114998	insertion	18
#4#	3634	3634	insertion	1
#4#	17650	17654	insertion	5
#4#	36615	36615	insertion	1
#4#	45071	45071	insertion	1
#4#	47537	47539	insertion	3
#4#	50019	50020	insertion	2
#4#	66082	66082	insertion	1
#4#	77462	77466	insertion	5
#11#	50017	50017	insertion	1
#14#	6874	6879	insertion	6
#14#	12141	12162	insertion	22
#14#	17088	17094	insertion	7
#14#	18139	18139	insertion	1
#14#	19497	19497	insertion	1
#14#	39236	39258	insertion	23
#14#	49930	49930	insertion	1
#14#	57901	57907	insertion	7
#14#	63199	63201	insertion	3
#14#	76861	76861	insertion	1
#14#	81537	81537	insertion	1
#14#	107477	107492	insertion	16
#5#	13911	13915	insertion	5
#5#	16465	16472	insertion	8
#5#	18964	18964	insertion	1
#5#	20940	20952	insertion	13
#5#	21335	21335	insertion	1
#5#	27723	27728	insertion	6
#5#	44922	44922	insertion	1
#5#	45299	45303	insertion	5
#5#	47284	47284	insertion	1
#5#	47928	47928	insertion	1
#5#	48083	48083	insertion	1
#5#	50717	50717	insertion	1
#5#	58230	58230	insertion	1
#5#	73805	73805	insertion	1
#5#	86323	86323	insertion	1
#5#	110662	110662	insertion	1
#5#	111193	111210	insertion	18
#5#	118567	118567	insertion	1
#6#	3631	3633	insertion	3
#6#	13678	13682	insertion	5
#6#	19802	19802	insertion	1
#6#	19806	19808	insertion	3
#6#	19848	19853	insertion	6

#6#	39613	39616	insertion	4
#6#	44931	44931	insertion	1
#6#	49799	49799	insertion	1
#6#	67693	67697	insertion	5
#6#	76104	76108	insertion	5
#6#	95590	95590	insertion	1
#6#	115380	115413	insertion	34
#6#	132473	132473	insertion	1
#7#	9069	9074	insertion	6
#7#	61925	61929	insertion	5
#7#	141738	141740	insertion	3
#8#	13401	13404	insertion	4
#8#	49818	49822	insertion	5
#8#	49929	49929	insertion	1
#8#	81528	81528	insertion	1
#12#	20346	20377	insertion	32
#12#	64376	64376	insertion	1
#15#	6247	6251	insertion	5
#15#	16069	16073	insertion	5
#15#	19494	19494	insertion	1
#15#	81536	81536	insertion	1
#15#	84044	84046	insertion	3
#16#	16064	16074	insertion	11
#16#	19796	19801	insertion	6
#16#	47527	47527	insertion	1
#16#	85788	85788	insertion	1
#17#	70944	70944	insertion	1
#17#	85787	85787	insertion	1
#18#	13194	13194	insertion	1
#18#	16992	16992	insertion	1
#18#	19818	19820	insertion	3
#18#	19832	19840	insertion	9
#18#	32681	32681	insertion	1
#18#	34792	34795	insertion	4
#18#	45943	45943	insertion	1
#18#	49806	49809	insertion	4
#18#	58205	58205	insertion	1
#18#	63233	63234	insertion	2
#18#	70708	70720	insertion	13
#18#	77207	77207	insertion	1
#18#	82134	82134	insertion	1
#18#	84606	84606	insertion	1
#18#	95586	95586	insertion	1
#18#	97086	97086	insertion	1
#18#	110016	110016	insertion	1
#18#	111110	111119	insertion	10
#18#	130983	130983	insertion	1

#18#	132480	132480	insertion	1
#19#	3635	3635	insertion	1
#19#	9085	9085	insertion	1
#19#	15430	15434	insertion	5
#19#	17924	17929	insertion	6
#19#	19775	19790	insertion	16
#19#	36507	36511	insertion	5
#19#	37676	37677	insertion	2
#19#	47698	47702	insertion	5
#19#	57797	57797	insertion	1
#19#	78842	78842	insertion	1
#19#	118457	118457	insertion	1
#20#	9081	9083	insertion	3
#20#	12052	12052	insertion	1
#20#	19490	19490	insertion	1
#20#	21905	21911	insertion	7
#20#	31311	31331	insertion	21
#20#	34688	34725	insertion	38
#20#	39730	39730	insertion	1
#20#	54236	54241	insertion	6
#20#	57801	57801	insertion	1
#20#	60933	60934	insertion	2
#20#	65662	65664	insertion	3
#20#	67611	67611	insertion	1
#20#	68624	68624	insertion	1
#20#	76855	76855	insertion	1
#20#	81782	81783	insertion	2
#20#	84069	84071	insertion	3
#20#	110474	110477	insertion	4
#20#	110554	110581	insertion	28
#20#	111338	111338	insertion	1
#20#	112367	112368	insertion	2
#21#	3362	3362	insertion	1
#21#	4293	4293	insertion	1
#21#	5625	5641	insertion	17
#21#	6087	6087	insertion	1
#21#	8607	8612	insertion	6
#21#	9068	9078	insertion	11
#21#	12940	12944	insertion	5
#21#	13505	13508	insertion	4
#21#	15389	15389	insertion	1
#21#	15452	15456	insertion	5
#21#	17614	17614	insertion	1
#21#	17675	17683	insertion	9
#21#	34818	34818	insertion	1
#21#	35827	35827	insertion	1
#21#	36103	36103	insertion	1

#21#	45066	45066	insertion	1
#21#	47305	47305	insertion	1
#21#	47522	47522	insertion	1
#21#	49643	49647	insertion	5
#21#	51311	51314	insertion	4
#21#	60154	60154	insertion	1
#21#	61880	61939	insertion	60
#21#	62027	62040	insertion	14
#21#	62471	62471	insertion	1
#21#	65675	65675	insertion	1
#21#	66075	66075	insertion	1
#21#	66328	66340	insertion	13
#21#	68370	68371	insertion	2
#21#	68778	68778	insertion	1
#21#	81531	81531	insertion	1
#21#	82207	82207	insertion	1
#21#	84651	84659	insertion	9
#21#	86336	86341	insertion	6
#21#	95587	95587	insertion	1
#21#	98978	98978	insertion	1
#21#	109782	109784	insertion	3
#21#	111615	111615	insertion	1
#21#	111762	111766	insertion	5
#21#	129088	129088	insertion	1
#21#	132479	132479	insertion	1
#21#	141732	141737	insertion	6
#22#	4294	4296	insertion	3
#22#	19791	19805	insertion	15
Total				4908

**Alignment topology with node labels: ((((O.granulata,O.meyeriana)#1#,(O.brachyantha,(O.longiglumis,O.ridleyi)#2#)#9#)#13#,(O.australiensis,(((O.rhizomatis,(O.officinalis,O.eichingeri)#3#)#10#,(O.latifolia,(O.alta,O.grandiglumis)#4#)#11#)#14#,((O.punctata,O.minuta)#5#,(O.longistaminata,(O.meridionalis,(O.glumaepatula,((O.glaberrima,O.barthii)#6#,((O.nivara,O.sativa_ssp.indica)#7#,(O.rufipogon,O.sativa_ssp.japonica)#8#)#12#)#15#)#16#)#17#)#18#)#19#)#20#)#21#)#22#,L.japonica)#23#;*

*** The Starting and ending position of the feature in the Prank+F alignment*

Supplementary Table 6b. Statistics of deletions for the twenty-three *Oryza* plastomes using the *L. japonica* genome as outgroup.

Branch*	Start**	End**	Feature	Length (bp)
O.granulata	12047	12047	deletion	1
O.granulata	20834	20834	deletion	1
O.granulata	49282	49282	deletion	1
O.granulata	49295	49295	deletion	1
O.meyeriana	9091	9091	deletion	1
O.meyeriana	54608	54608	deletion	1
O.meyeriana	66079	66079	deletion	1
O.meyeriana	84212	84216	deletion	5
O.brachyantha	1371	1377	deletion	7
O.brachyantha	1665	1695	deletion	31
O.brachyantha	4151	4151	deletion	1
O.brachyantha	4179	4196	deletion	18
O.brachyantha	4200	4239	deletion	40
O.brachyantha	4294	4309	deletion	16
O.brachyantha	4890	4892	deletion	3
O.brachyantha	5243	5245	deletion	3
O.brachyantha	5332	5344	deletion	13
O.brachyantha	5623	5658	deletion	36
O.brachyantha	6172	6172	deletion	1
O.brachyantha	6497	6498	deletion	2
O.brachyantha	6632	6645	deletion	14
O.brachyantha	6690	6722	deletion	33
O.brachyantha	6806	6810	deletion	5
O.brachyantha	7177	7177	deletion	1
O.brachyantha	7471	7476	deletion	6
O.brachyantha	8019	8027	deletion	9
O.brachyantha	9158	9159	deletion	2
O.brachyantha	9385	9385	deletion	1
O.brachyantha	11962	11962	deletion	1
O.brachyantha	12475	12479	deletion	5
O.brachyantha	14140	14146	deletion	7
O.brachyantha	15383	15445	deletion	63
O.brachyantha	15718	15916	deletion	199
O.brachyantha	15974	16221	deletion	248
O.brachyantha	16311	16360	deletion	50
O.brachyantha	16871	16931	deletion	61
O.brachyantha	17602	17674	deletion	73
O.brachyantha	18092	18095	deletion	4
O.brachyantha	19738	19754	deletion	17
O.brachyantha	20835	20835	deletion	1
O.brachyantha	21021	21084	deletion	64
O.brachyantha	21375	21402	deletion	28
O.brachyantha	21550	21550	deletion	1

O.brachyantha	21796	21866	deletion	71
O.brachyantha	32544	32549	deletion	6
O.brachyantha	32649	32663	deletion	15
O.brachyantha	35856	35856	deletion	1
O.brachyantha	36773	36773	deletion	1
O.brachyantha	37092	37092	deletion	1
O.brachyantha	39506	39580	deletion	75
O.brachyantha	45152	45152	deletion	1
O.brachyantha	45312	45312	deletion	1
O.brachyantha	46242	46244	deletion	3
O.brachyantha	46277	46277	deletion	1
O.brachyantha	47309	47314	deletion	6
O.brachyantha	48000	48010	deletion	11
O.brachyantha	48265	48265	deletion	1
O.brachyantha	49238	49242	deletion	5
O.brachyantha	49517	49523	deletion	7
O.brachyantha	49618	49624	deletion	7
O.brachyantha	49900	49900	deletion	1
O.brachyantha	50026	50026	deletion	1
O.brachyantha	50454	50484	deletion	31
O.brachyantha	50774	50777	deletion	4
O.brachyantha	50979	50979	deletion	1
O.brachyantha	54579	54607	deletion	29
O.brachyantha	55593	55611	deletion	19
O.brachyantha	58039	58039	deletion	1
O.brachyantha	60484	60510	deletion	27
O.brachyantha	61060	61194	deletion	135
O.brachyantha	61359	61359	deletion	1
O.brachyantha	61854	61871	deletion	18
O.brachyantha	63156	63156	deletion	1
O.brachyantha	63544	63561	deletion	18
O.brachyantha	64320	64329	deletion	10
O.brachyantha	64354	64354	deletion	1
O.brachyantha	64378	64386	deletion	9
O.brachyantha	65889	65916	deletion	28
O.brachyantha	66204	66225	deletion	22
O.brachyantha	67910	67914	deletion	5
O.brachyantha	68428	68493	deletion	66
O.brachyantha	68626	68626	deletion	1
O.brachyantha	69089	69094	deletion	6
O.brachyantha	69132	69137	deletion	6
O.brachyantha	70952	70955	deletion	4
O.brachyantha	72546	72546	deletion	1
O.brachyantha	73906	73922	deletion	17
O.brachyantha	73953	73989	deletion	37
O.brachyantha	76865	76865	deletion	1
O.brachyantha	76944	76946	deletion	3

O.brachyantha	78782	78788	deletion	7
O.brachyantha	83880	83900	deletion	21
O.brachyantha	84049	84054	deletion	6
O.brachyantha	84077	84083	deletion	7
O.brachyantha	84200	84203	deletion	4
O.brachyantha	84207	84212	deletion	6
O.brachyantha	84848	84848	deletion	1
O.brachyantha	86678	86678	deletion	1
O.brachyantha	107465	107498	deletion	34
O.brachyantha	109797	109797	deletion	1
O.brachyantha	110280	110287	deletion	8
O.brachyantha	110326	110346	deletion	21
O.brachyantha	110390	110391	deletion	2
O.brachyantha	111315	111335	deletion	21
O.brachyantha	112824	112833	deletion	10
O.brachyantha	112923	112923	deletion	1
O.brachyantha	115294	115294	deletion	1
O.brachyantha	116606	116606	deletion	1
O.brachyantha	118877	118881	deletion	5
O.brachyantha	141386	141386	deletion	1
O.longiglumis	17728	17729	deletion	2
O.longiglumis	46786	46786	deletion	1
O.longiglumis	49120	49120	deletion	1
O.longiglumis	49620	49620	deletion	1
O.longiglumis	49920	49920	deletion	1
O.longiglumis	51789	51793	deletion	5
O.longiglumis	65793	65798	deletion	6
O.longiglumis	70023	70023	deletion	1
O.longiglumis	90624	90673	deletion	50
O.longiglumis	113987	114003	deletion	17
O.longiglumis	114185	114200	deletion	16
O.longiglumis	137288	137337	deletion	50
O.ridleyi	9093	9093	deletion	1
O.ridleyi	17206	17206	deletion	1
O.ridleyi	17656	17656	deletion	1
O.ridleyi	37671	37671	deletion	1
O.ridleyi	109916	109916	deletion	1
O.australiensis	1308	1316	deletion	9
O.australiensis	1454	1454	deletion	1
O.australiensis	4293	4296	deletion	4
O.australiensis	6409	6439	deletion	31
O.australiensis	7299	7299	deletion	1
O.australiensis	15972	15972	deletion	1
O.australiensis	17655	17655	deletion	1
O.australiensis	18202	18202	deletion	1
O.australiensis	19791	19805	deletion	15
O.australiensis	19867	19933	deletion	67

O.australiensis	21811	21811	deletion	1
O.australiensis	30240	30272	deletion	33
O.australiensis	35409	35409	deletion	1
O.australiensis	44934	44934	deletion	1
O.australiensis	45033	45033	deletion	1
O.australiensis	46318	46322	deletion	5
O.australiensis	47748	47757	deletion	10
O.australiensis	49699	49699	deletion	1
O.australiensis	50035	50035	deletion	1
O.australiensis	50796	50796	deletion	1
O.australiensis	50985	50985	deletion	1
O.australiensis	51791	51795	deletion	5
O.australiensis	54608	54608	deletion	1
O.australiensis	60068	60078	deletion	11
O.australiensis	60083	60088	deletion	6
O.australiensis	61872	61873	deletion	2
O.australiensis	69089	69097	deletion	9
O.australiensis	70383	70403	deletion	21
O.australiensis	72546	72546	deletion	1
O.australiensis	73807	73807	deletion	1
O.australiensis	73891	73896	deletion	6
O.australiensis	76998	76998	deletion	1
O.australiensis	78782	78785	deletion	4
O.australiensis	81443	81443	deletion	1
O.australiensis	98978	98979	deletion	2
O.australiensis	109803	109803	deletion	1
O.australiensis	110665	110665	deletion	1
O.australiensis	111410	111410	deletion	1
O.australiensis	115332	115337	deletion	6
O.australiensis	118434	118456	deletion	23
O.australiensis	118531	118531	deletion	1
O.australiensis	119054	119054	deletion	1
O.australiensis	129087	129088	deletion	2
O.rhizomatis	17614	17614	deletion	1
O.rhizomatis	17655	17655	deletion	1
O.rhizomatis	18091	18091	deletion	1
O.rhizomatis	45101	45101	deletion	1
O.rhizomatis	61862	61862	deletion	1
O.rhizomatis	63157	63157	deletion	1
O.rhizomatis	66075	66075	deletion	1
O.rhizomatis	68377	68377	deletion	1
O.rhizomatis	70946	70946	deletion	1
O.rhizomatis	76861	76861	deletion	1
O.rhizomatis	81782	81782	deletion	1
O.rhizomatis	82208	82208	deletion	1
O.rhizomatis	84065	84067	deletion	3
O.officinalis	60237	60253	deletion	17

O.officinalis	70946	70947	deletion	2
O.eichingeri	27847	27847	deletion	1
O.eichingeri	83337	83337	deletion	1
O.latifolia	6984	6984	deletion	1
O.latifolia	12041	12042	deletion	2
O.latifolia	50251	50251	deletion	1
O.latifolia	57901	57907	deletion	7
O.latifolia	58489	58489	deletion	1
O.latifolia	60237	60253	deletion	17
O.latifolia	68373	68374	deletion	2
O.latifolia	83335	83335	deletion	1
O.latifolia	84651	84651	deletion	1
O.alta	6167	6167	deletion	1
O.alta	49930	49930	deletion	1
O.alta	112361	112362	deletion	2
O.grandiglumis	66082	66087	deletion	6
O.grandiglumis	84876	84876	deletion	1
O.grandiglumis	86332	86332	deletion	1
O.grandiglumis	141722	141722	deletion	1
O.punctata	35011	35018	deletion	8
O.punctata	37677	37677	deletion	1
O.punctata	50711	50715	deletion	5
O.punctata	60245	60253	deletion	9
O.punctata	65793	65798	deletion	6
O.punctata	84042	84047	deletion	6
O.punctata	112367	112368	deletion	2
O.minuta	112361	112362	deletion	2
O.longistaminata	47522	47522	deletion	1
O.longistaminata	58426	58426	deletion	1
O.longistaminata	58484	58484	deletion	1
O.longistaminata	60582	60582	deletion	1
O.longistaminata	63262	63267	deletion	6
O.longistaminata	84047	84065	deletion	19
O.longistaminata	86338	86340	deletion	3
O.longistaminata	112367	112368	deletion	2
O.longistaminata	141736	141736	deletion	1
O.meridionalis	14326	14326	deletion	1
O.meridionalis	19775	19780	deletion	6
O.meridionalis	69988	70010	deletion	23
O.meridionalis	83330	83330	deletion	1
O.meridionalis	86337	86340	deletion	4
O.meridionalis	141733	141736	deletion	4
O.glumaepatula	1308	1316	deletion	9
O.glumaepatula	1453	1453	deletion	1
O.glumaepatula	3617	3617	deletion	1
O.glumaepatula	4290	4295	deletion	6
O.glumaepatula	6411	6441	deletion	31

O.glumaepatula	7301	7301	deletion	1
O.glumaepatula	13199	13199	deletion	1
O.glumaepatula	15430	15434	deletion	5
O.glumaepatula	15972	15972	deletion	1
O.glumaepatula	16993	16993	deletion	1
O.glumaepatula	17655	17655	deletion	1
O.glumaepatula	17923	17928	deletion	6
O.glumaepatula	18198	18198	deletion	1
O.glumaepatula	19777	19810	deletion	34
O.glumaepatula	19866	19932	deletion	67
O.glumaepatula	21811	21811	deletion	1
O.glumaepatula	21906	21912	deletion	7
O.glumaepatula	30238	30270	deletion	33
O.glumaepatula	31311	31331	deletion	21
O.glumaepatula	32683	32683	deletion	1
O.glumaepatula	34793	34796	deletion	4
O.glumaepatula	35409	35409	deletion	1
O.glumaepatula	36506	36510	deletion	5
O.glumaepatula	37677	37677	deletion	1
O.glumaepatula	39731	39731	deletion	1
O.glumaepatula	44934	44934	deletion	1
O.glumaepatula	45033	45033	deletion	1
O.glumaepatula	45945	45945	deletion	1
O.glumaepatula	46319	46323	deletion	5
O.glumaepatula	47696	47700	deletion	5
O.glumaepatula	47748	47757	deletion	10
O.glumaepatula	47807	47809	deletion	3
O.glumaepatula	49699	49699	deletion	1
O.glumaepatula	49808	49811	deletion	4
O.glumaepatula	50796	50796	deletion	1
O.glumaepatula	50985	50985	deletion	1
O.glumaepatula	51784	51787	deletion	4
O.glumaepatula	54235	54240	deletion	6
O.glumaepatula	54608	54608	deletion	1
O.glumaepatula	57798	57798	deletion	1
O.glumaepatula	58205	58205	deletion	1
O.glumaepatula	60070	60088	deletion	19
O.glumaepatula	60933	60934	deletion	2
O.glumaepatula	61864	61864	deletion	1
O.glumaepatula	63232	63233	deletion	2
O.glumaepatula	65662	65664	deletion	3
O.glumaepatula	67612	67612	deletion	1
O.glumaepatula	69089	69097	deletion	9
O.glumaepatula	70006	70019	deletion	14
O.glumaepatula	70209	70213	deletion	5
O.glumaepatula	72544	72544	deletion	1
O.glumaepatula	73806	73806	deletion	1

O.glumaepatula	73890	73906	deletion	17
O.glumaepatula	76853	76853	deletion	1
O.glumaepatula	77207	77207	deletion	1
O.glumaepatula	78779	78783	deletion	5
O.glumaepatula	78841	78841	deletion	1
O.glumaepatula	81781	81782	deletion	2
O.glumaepatula	82136	82136	deletion	1
O.glumaepatula	84047	84064	deletion	18
O.glumaepatula	84608	84608	deletion	1
O.glumaepatula	95597	95597	deletion	1
O.glumaepatula	97087	97087	deletion	1
O.glumaepatula	98982	98983	deletion	2
O.glumaepatula	108893	108902	deletion	10
O.glumaepatula	110019	110019	deletion	1
O.glumaepatula	110471	110474	deletion	4
O.glumaepatula	110554	110555	deletion	2
O.glumaepatula	110666	110666	deletion	1
O.glumaepatula	111098	111107	deletion	10
O.glumaepatula	111416	111416	deletion	1
O.glumaepatula	112367	112368	deletion	2
O.glumaepatula	115248	115251	deletion	4
O.glumaepatula	115332	115337	deletion	6
O.glumaepatula	118434	118457	deletion	24
O.glumaepatula	118531	118531	deletion	1
O.glumaepatula	119054	119054	deletion	1
O.glumaepatula	129087	129088	deletion	2
O.glumaepatula	130979	130979	deletion	1
O.glumaepatula	132480	132480	deletion	1
O.glaberrima	47523	47523	deletion	1
O.glaberrima	55508	55510	deletion	3
O.glaberrima	66537	66537	deletion	1
O.glaberrima	66590	66590	deletion	1
O.glaberrima	77351	77351	deletion	1
O.glaberrima	77375	77380	deletion	6
O.glaberrima	86343	86345	deletion	3
O.glaberrima	141731	141733	deletion	3
O.barthii	19798	19810	deletion	13
O.nivara	3612	3612	deletion	1
O.nivara	6247	6251	deletion	5
O.nivara	13197	13197	deletion	1
O.nivara	15430	15434	deletion	5
O.nivara	16051	16067	deletion	17
O.nivara	16990	16990	deletion	1
O.nivara	17618	17627	deletion	10
O.nivara	17917	17922	deletion	6
O.nivara	18154	18154	deletion	1
O.nivara	19747	19785	deletion	39

O.nivara	20322	20353	deletion	32
O.nivara	21647	21647	deletion	1
O.nivara	27847	27847	deletion	1
O.nivara	32683	32683	deletion	1
O.nivara	34795	34798	deletion	4
O.nivara	34924	35283	deletion	360
O.nivara	36509	36513	deletion	5
O.nivara	36612	36618	deletion	7
O.nivara	37677	37677	deletion	1
O.nivara	45944	45944	deletion	1
O.nivara	47475	47482	deletion	8
O.nivara	47530	47530	deletion	1
O.nivara	47696	47700	deletion	5
O.nivara	49305	49305	deletion	1
O.nivara	49627	49627	deletion	1
O.nivara	53883	53883	deletion	1
O.nivara	66475	66475	deletion	1
O.nivara	68779	68779	deletion	1
O.nivara	69940	69949	deletion	10
O.nivara	70202	70211	deletion	10
O.nivara	77191	77194	deletion	4
O.nivara	78839	78839	deletion	1
O.nivara	79283	79283	deletion	1
O.nivara	82135	82135	deletion	1
O.nivara	82279	82299	deletion	21
O.nivara	84607	84607	deletion	1
O.nivara	85799	85799	deletion	1
O.nivara	95592	95592	deletion	1
O.nivara	97086	97086	deletion	1
O.nivara	98982	98982	deletion	1
O.nivara	107458	107493	deletion	36
O.nivara	111033	111036	deletion	4
O.nivara	111099	111108	deletion	10
O.nivara	115422	115424	deletion	3
O.nivara	118460	118460	deletion	1
O.nivara	129089	129089	deletion	1
O.nivara	130983	130983	deletion	1
O.nivara	132479	132479	deletion	1
O.sativa_ssp.indic	6645	6645	deletion	1
O.sativa_ssp.indic	9068	9153	deletion	86
O.sativa_ssp.indic	50300	50305	deletion	6
O.sativa_ssp.indic	60238	60253	deletion	16
O.sativa_ssp.indic	64375	64375	deletion	1
O.sativa_ssp.indic	67551	67558	deletion	8
O.sativa_ssp.indic	70938	70940	deletion	3
O.sativa_ssp.indic	84040	84040	deletion	1
O.sativa_ssp.indic	141738	141746	deletion	9

O.rufipogon	19797	19805	deletion	9
O.rufipogon	60652	60652	deletion	1
O.rufipogon	64376	64376	deletion	1
O.rufipogon	70177	70181	deletion	5
O.sativa_ssp.japo	5565	5565	deletion	1
O.sativa_ssp.japo	9225	9225	deletion	1
O.sativa_ssp.japo	14253	14253	deletion	1
O.sativa_ssp.japo	15387	15387	deletion	1
O.sativa_ssp.japo	19821	19822	deletion	2
O.sativa_ssp.japo	20346	20377	deletion	32
O.sativa_ssp.japo	37583	37583	deletion	1
O.sativa_ssp.japo	45942	45942	deletion	1
O.sativa_ssp.japo	47223	47224	deletion	2
O.sativa_ssp.japo	47531	47531	deletion	1
O.sativa_ssp.japo	50601	50601	deletion	1
O.sativa_ssp.japo	50956	50956	deletion	1
O.sativa_ssp.japo	50985	50985	deletion	1
O.sativa_ssp.japo	53884	53884	deletion	1
O.sativa_ssp.japo	55034	55035	deletion	2
O.sativa_ssp.japo	58163	58163	deletion	1
O.sativa_ssp.japo	58375	58389	deletion	15
O.sativa_ssp.japo	60238	60253	deletion	16
O.sativa_ssp.japo	61072	61072	deletion	1
O.sativa_ssp.japo	61778	61778	deletion	1
O.sativa_ssp.japo	63385	63385	deletion	1
O.sativa_ssp.japo	63489	63489	deletion	1
O.sativa_ssp.japo	73978	73978	deletion	1
O.sativa_ssp.japo	74081	74082	deletion	2
O.sativa_ssp.japo	77350	77350	deletion	1
O.sativa_ssp.japo	77383	77383	deletion	1
O.sativa_ssp.japo	77431	77431	deletion	1
O.sativa_ssp.japo	78428	78428	deletion	1
O.sativa_ssp.japo	78481	78481	deletion	1
O.sativa_ssp.japo	78546	78546	deletion	1
O.sativa_ssp.japo	78621	78621	deletion	1
O.sativa_ssp.japo	82299	82299	deletion	1
O.sativa_ssp.japo	84545	84545	deletion	1
O.sativa_ssp.japo	93722	93722	deletion	1
O.sativa_ssp.japo	96014	96014	deletion	1
O.sativa_ssp.japo	96249	96249	deletion	1
O.sativa_ssp.japo	96400	96400	deletion	1
O.sativa_ssp.japo	102851	102851	deletion	1
O.sativa_ssp.japo	103141	103141	deletion	1
O.sativa_ssp.japo	103435	103436	deletion	2
O.sativa_ssp.japo	104001	104001	deletion	1
O.sativa_ssp.japo	104860	104861	deletion	2
O.sativa_ssp.japo	105001	105001	deletion	1

O.sativa_ssp.japoi	106109	106109	deletion	1
O.sativa_ssp.japoi	107840	107840	deletion	1
O.sativa_ssp.japoi	110619	110619	deletion	1
O.sativa_ssp.japoi	111024	111027	deletion	4
O.sativa_ssp.japoi	112367	112368	deletion	2
O.sativa_ssp.japoi	117389	117394	deletion	6
O.sativa_ssp.japoi	118501	118501	deletion	1
O.sativa_ssp.japoi	121957	121957	deletion	1
O.sativa_ssp.japoi	123066	123066	deletion	1
O.sativa_ssp.japoi	123204	123205	deletion	2
O.sativa_ssp.japoi	124065	124065	deletion	1
O.sativa_ssp.japoi	124631	124632	deletion	2
O.sativa_ssp.japoi	124925	124925	deletion	1
O.sativa_ssp.japoi	125215	125215	deletion	1
O.sativa_ssp.japoi	131666	131666	deletion	1
O.sativa_ssp.japoi	131817	131817	deletion	1
O.sativa_ssp.japoi	132052	132052	deletion	1
O.sativa_ssp.japoi	134343	134343	deletion	1
L.japonica	1633	1641	deletion	9
L.japonica	3276	3276	deletion	1
L.japonica	4254	4258	deletion	5
L.japonica	5123	5139	deletion	17
L.japonica	5253	5253	deletion	1
L.japonica	5484	5485	deletion	2
L.japonica	6429	6429	deletion	1
L.japonica	6484	7091	deletion	608
L.japonica	8892	8892	deletion	1
L.japonica	8937	8946	deletion	10
L.japonica	9062	9062	deletion	1
L.japonica	9065	9080	deletion	16
L.japonica	12097	12097	deletion	1
L.japonica	12478	12478	deletion	1
L.japonica	13370	13371	deletion	2
L.japonica	13440	13448	deletion	9
L.japonica	13485	13485	deletion	1
L.japonica	14430	14430	deletion	1
L.japonica	14460	14464	deletion	5
L.japonica	15387	15391	deletion	5
L.japonica	15509	15510	deletion	2
L.japonica	16077	16081	deletion	5
L.japonica	16912	16913	deletion	2
L.japonica	16930	16932	deletion	3
L.japonica	17116	17116	deletion	1
L.japonica	17128	17133	deletion	6
L.japonica	17627	17628	deletion	2
L.japonica	17853	17855	deletion	3
L.japonica	18128	18132	deletion	5

L.japonica	18183	18183	deletion	1
L.japonica	18412	18417	deletion	6
L.japonica	18483	18483	deletion	1
L.japonica	19404	19408	deletion	5
L.japonica	19831	19845	deletion	15
L.japonica	19926	19935	deletion	10
L.japonica	20443	20443	deletion	1
L.japonica	20549	20567	deletion	19
L.japonica	20578	20578	deletion	1
L.japonica	20791	20803	deletion	13
L.japonica	21017	21019	deletion	3
L.japonica	21811	21811	deletion	1
L.japonica	21881	21881	deletion	1
L.japonica	22348	22348	deletion	1
L.japonica	27370	27389	deletion	20
L.japonica	27913	27913	deletion	1
L.japonica	30046	30087	deletion	42
L.japonica	34656	34656	deletion	1
L.japonica	34842	34844	deletion	3
L.japonica	34908	34908	deletion	1
L.japonica	35807	35859	deletion	53
L.japonica	36773	36773	deletion	1
L.japonica	37049	37090	deletion	42
L.japonica	39365	39365	deletion	1
L.japonica	39501	39505	deletion	5
L.japonica	44934	44934	deletion	1
L.japonica	45025	45145	deletion	121
L.japonica	45397	45397	deletion	1
L.japonica	45763	45763	deletion	1
L.japonica	46320	46324	deletion	5
L.japonica	47530	47531	deletion	2
L.japonica	48250	48254	deletion	5
L.japonica	48444	48449	deletion	6
L.japonica	49230	49230	deletion	1
L.japonica	49414	49414	deletion	1
L.japonica	49618	49622	deletion	5
L.japonica	49800	49877	deletion	78
L.japonica	49904	49904	deletion	1
L.japonica	50246	50246	deletion	1
L.japonica	50763	50763	deletion	1
L.japonica	51339	51339	deletion	1
L.japonica	51656	51658	deletion	3
L.japonica	51681	51686	deletion	6
L.japonica	51737	51740	deletion	4
L.japonica	51889	51893	deletion	5
L.japonica	54135	54155	deletion	21
L.japonica	54629	54633	deletion	5

L.japonica	55587	55587	deletion	1
L.japonica	58264	58264	deletion	1
L.japonica	58434	58451	deletion	18
L.japonica	58533	58533	deletion	1
L.japonica	60075	60075	deletion	1
L.japonica	60237	60253	deletion	17
L.japonica	60584	60584	deletion	1
L.japonica	61806	61879	deletion	74
L.japonica	62384	62388	deletion	5
L.japonica	62400	62400	deletion	1
L.japonica	65793	65798	deletion	6
L.japonica	66079	66079	deletion	1
L.japonica	66305	66323	deletion	19
L.japonica	66476	66476	deletion	1
L.japonica	68158	68158	deletion	1
L.japonica	68338	68338	deletion	1
L.japonica	68626	68626	deletion	1
L.japonica	68844	68851	deletion	8
L.japonica	69270	69270	deletion	1
L.japonica	69667	69667	deletion	1
L.japonica	70383	70403	deletion	21
L.japonica	71693	71693	deletion	1
L.japonica	72238	72242	deletion	5
L.japonica	72654	72654	deletion	1
L.japonica	72769	72782	deletion	14
L.japonica	76865	76865	deletion	1
L.japonica	76943	76947	deletion	5
L.japonica	76997	76998	deletion	2
L.japonica	79799	79805	deletion	7
L.japonica	81443	81443	deletion	1
L.japonica	81498	81540	deletion	43
L.japonica	82142	82193	deletion	52
L.japonica	82275	82295	deletion	21
L.japonica	84024	84047	deletion	24
L.japonica	84823	84846	deletion	24
L.japonica	84978	84978	deletion	1
L.japonica	85540	85584	deletion	45
L.japonica	86678	86678	deletion	1
L.japonica	89046	89062	deletion	17
L.japonica	89193	89735	deletion	543
L.japonica	102777	102841	deletion	65
L.japonica	109865	109865	deletion	1
L.japonica	109891	109897	deletion	7
L.japonica	110015	110015	deletion	1
L.japonica	110280	110287	deletion	8
L.japonica	110528	110528	deletion	1
L.japonica	110541	110546	deletion	6

L.japonica	110601	110610	deletion	10
L.japonica	111023	111026	deletion	4
L.japonica	111330	111330	deletion	1
L.japonica	111407	111409	deletion	3
L.japonica	112329	112378	deletion	50
L.japonica	115336	115337	deletion	2
L.japonica	115823	115826	deletion	4
L.japonica	117441	117441	deletion	1
L.japonica	118160	118164	deletion	5
L.japonica	118301	118305	deletion	5
L.japonica	118460	118460	deletion	1
L.japonica	118500	118500	deletion	1
L.japonica	118528	118528	deletion	1
L.japonica	125222	125286	deletion	65
L.japonica	138320	138862	deletion	543
L.japonica	139018	139034	deletion	17
L.japonica	141386	141386	deletion	1
#1#	4239	4239	deletion	1
#1#	4294	4305	deletion	12
#1#	4387	4387	deletion	1
#1#	6413	6417	deletion	5
#1#	14430	14430	deletion	1
#1#	15387	15391	deletion	5
#1#	15509	15510	deletion	2
#1#	15792	15792	deletion	1
#1#	15806	15806	deletion	1
#1#	16215	16242	deletion	28
#1#	16861	16872	deletion	12
#1#	17206	17206	deletion	1
#1#	18646	18646	deletion	1
#1#	19305	19309	deletion	5
#1#	19769	19805	deletion	37
#1#	32649	32662	deletion	14
#1#	33800	33800	deletion	1
#1#	35124	35150	deletion	27
#1#	47531	47531	deletion	1
#1#	49255	49255	deletion	1
#1#	49618	49623	deletion	6
#1#	49920	49920	deletion	1
#1#	51789	51793	deletion	5
#1#	57794	57795	deletion	2
#1#	58193	58202	deletion	10
#1#	58531	58531	deletion	1
#1#	60057	60057	deletion	1
#1#	60353	60353	deletion	1
#1#	65793	65798	deletion	6
#1#	68911	68911	deletion	1

#1#	69591	69636	deletion	46
#1#	70947	70948	deletion	2
#1#	72686	72686	deletion	1
#1#	78782	78783	deletion	2
#1#	84076	84076	deletion	1
#1#	110394	110395	deletion	2
#1#	112830	112830	deletion	1
#1#	112920	112920	deletion	1
#1#	118297	118306	deletion	10
#1#	118811	118823	deletion	13
#2#	6362	6374	deletion	13
#2#	6882	6882	deletion	1
#2#	9065	9084	deletion	20
#2#	12955	12956	deletion	2
#2#	13150	13150	deletion	1
#2#	13418	13420	deletion	3
#2#	16705	16764	deletion	60
#2#	17767	17773	deletion	7
#2#	18130	18130	deletion	1
#2#	18157	18201	deletion	45
#2#	18280	18280	deletion	1
#2#	19791	19805	deletion	15
#2#	21881	21881	deletion	1
#2#	27915	27915	deletion	1
#2#	33578	33599	deletion	22
#2#	46086	46086	deletion	1
#2#	47530	47531	deletion	2
#2#	47958	47958	deletion	1
#2#	50802	50802	deletion	1
#2#	51259	51273	deletion	15
#2#	51335	51335	deletion	1
#2#	51590	51590	deletion	1
#2#	60061	60079	deletion	19
#2#	60237	60253	deletion	17
#2#	60568	60622	deletion	55
#2#	63388	63388	deletion	1
#2#	66532	66532	deletion	1
#2#	67935	67938	deletion	4
#2#	69629	69739	deletion	111
#2#	70309	70309	deletion	1
#2#	76913	76913	deletion	1
#2#	76997	76998	deletion	2
#2#	81787	81787	deletion	1
#2#	83335	83341	deletion	7
#2#	84067	84075	deletion	9
#2#	87674	87674	deletion	1
#2#	107109	107109	deletion	1

#2#	118382	118420	deletion	39
#2#	118503	118506	deletion	4
#2#	119031	119034	deletion	4
#2#	120950	120950	deletion	1
#2#	140392	140392	deletion	1
#9#	5486	5486	deletion	1
#9#	13251	13312	deletion	62
#9#	16725	16726	deletion	2
#9#	18155	18155	deletion	1
#9#	19127	19128	deletion	2
#9#	36094	36094	deletion	1
#9#	53943	53992	deletion	50
#9#	60583	60583	deletion	1
#9#	65603	65603	deletion	1
#9#	84032	84048	deletion	17
#9#	89046	89062	deletion	17
#9#	107440	107462	deletion	23
#9#	111079	111079	deletion	1
#9#	111587	111601	deletion	15
#9#	118460	118460	deletion	1
#9#	119032	119032	deletion	1
#9#	139018	139034	deletion	17
#13#	3613	3613	deletion	1
#13#	4290	4292	deletion	3
#13#	5728	5729	deletion	2
#13#	6190	6211	deletion	22
#13#	6315	6335	deletion	21
#13#	17811	17814	deletion	4
#13#	34599	34599	deletion	1
#13#	37672	37672	deletion	1
#13#	50032	50035	deletion	4
#13#	51339	51340	deletion	2
#13#	55505	55512	deletion	8
#13#	60068	60075	deletion	8
#13#	60786	60786	deletion	1
#13#	61373	61373	deletion	1
#13#	65604	65604	deletion	1
#13#	68019	68306	deletion	288
#13#	70945	70946	deletion	2
#13#	82247	82294	deletion	48
#13#	84254	84254	deletion	1
#3#	21646	21646	deletion	1
#3#	68624	68624	deletion	1
#10#	17622	17626	deletion	5
#10#	34677	34695	deletion	19
#10#	34920	35279	deletion	360
#10#	66477	66477	deletion	1

#10#	69940	69949	deletion	10
#10#	82286	82306	deletion	21
#10#	84653	84654	deletion	2
#10#	110538	110563	deletion	26
#10#	112361	112362	deletion	2
#4#	17208	17208	deletion	1
#4#	45026	45026	deletion	1
#4#	64363	64370	deletion	8
#4#	65956	65961	deletion	6
#4#	68156	68156	deletion	1
#4#	68631	68631	deletion	1
#4#	84067	84068	deletion	2
#11#	47534	47535	deletion	2
#11#	112367	112368	deletion	2
#14#	6645	6645	deletion	1
#14#	18157	18157	deletion	1
#14#	19821	19844	deletion	24
#14#	36620	36625	deletion	6
#14#	47475	47482	deletion	8
#14#	47522	47522	deletion	1
#14#	49627	49627	deletion	1
#14#	62401	62401	deletion	1
#14#	68375	68376	deletion	2
#14#	68778	68778	deletion	1
#14#	84845	84845	deletion	1
#14#	85802	85802	deletion	1
#14#	86340	86341	deletion	2
#14#	98977	98977	deletion	1
#14#	107457	107476	deletion	20
#14#	115432	115434	deletion	3
#14#	129089	129089	deletion	1
#5#	5626	5642	deletion	17
#5#	6488	6982	deletion	495
#5#	12482	12482	deletion	1
#5#	12855	12855	deletion	1
#5#	15516	15516	deletion	1
#5#	17655	17655	deletion	1
#5#	19775	19805	deletion	31
#5#	48270	48279	deletion	10
#5#	62319	62331	deletion	13
#5#	64425	64425	deletion	1
#5#	65549	65615	deletion	67
#5#	78841	78842	deletion	2
#5#	86337	86341	deletion	5
#5#	86679	86679	deletion	1
#5#	98977	98978	deletion	2
#5#	110519	110519	deletion	1

#5#	129088	129089	deletion	2
#5#	141387	141387	deletion	1
#5#	141733	141737	deletion	5
#6#	70940	70940	deletion	1
#6#	76099	76103	deletion	5
#6#	84041	84046	deletion	6
#6#	84659	84659	deletion	1
#6#	86340	86340	deletion	1
#6#	141736	141736	deletion	1
#7#	19821	19844	deletion	24
#8#	86340	86341	deletion	2
#8#	141736	141737	deletion	2
#15#	72277	72295	deletion	19
#15#	85787	85788	deletion	2
#15#	86331	86332	deletion	2
#15#	99511	99511	deletion	1
#15#	128551	128551	deletion	1
#16#	19818	19820	deletion	3
#16#	19832	19840	deletion	9
#16#	70708	70720	deletion	13
#17#	19490	19490	deletion	1
#17#	36771	36771	deletion	1
#17#	37676	37676	deletion	1
#17#	57797	57797	deletion	1
#17#	112361	112362	deletion	2
#18#	4254	4257	deletion	4
#18#	4373	4373	deletion	1
#18#	5086	5154	deletion	69
#18#	8732	8732	deletion	1
#18#	9078	9084	deletion	7
#18#	9380	9380	deletion	1
#18#	12052	12052	deletion	1
#18#	13216	13216	deletion	1
#18#	15127	15382	deletion	256
#18#	16322	16327	deletion	6
#18#	17614	17614	deletion	1
#18#	21604	21625	deletion	22
#18#	34688	34725	deletion	38
#18#	35016	35032	deletion	17
#18#	51150	51219	deletion	70
#18#	51427	51459	deletion	33
#18#	54306	54391	deletion	86
#18#	61873	61873	deletion	1
#18#	62401	62402	deletion	2
#18#	63542	63559	deletion	18
#18#	68624	68625	deletion	2
#18#	70383	70403	deletion	21

#18#	76998	76998	deletion	1
#18#	81443	81443	deletion	1
#18#	82207	82207	deletion	1
#18#	84071	84071	deletion	1
#18#	84651	84657	deletion	7
#18#	84876	84876	deletion	1
#18#	105294	105294	deletion	1
#18#	109803	109803	deletion	1
#18#	110556	110581	deletion	26
#18#	111334	111334	deletion	1
#18#	111473	111476	deletion	4
#18#	122771	122771	deletion	1
#19#	5255	5255	deletion	1
#19#	9413	9416	deletion	4
#19#	15388	15396	deletion	9
#19#	15452	15456	deletion	5
#19#	37039	37039	deletion	1
#19#	49836	49836	deletion	1
#19#	50034	50035	deletion	2
#19#	60237	60237	deletion	1
#19#	62566	62571	deletion	6
#19#	66075	66075	deletion	1
#19#	68844	68844	deletion	1
#19#	70945	70946	deletion	2
#19#	78670	78670	deletion	1
#19#	83335	83335	deletion	1
#19#	84658	84658	deletion	1
#19#	109865	109865	deletion	1
#19#	110450	110458	deletion	9
#20#	5485	5485	deletion	1
#20#	15512	15512	deletion	1
#20#	16126	16135	deletion	10
#20#	22102	22104	deletion	3
#20#	27913	27913	deletion	1
#20#	32671	32672	deletion	2
#20#	37052	37052	deletion	1
#20#	37593	37596	deletion	4
#20#	61500	61568	deletion	69
#20#	63333	63337	deletion	5
#20#	70847	70864	deletion	18
#20#	84641	84642	deletion	2
#20#	99364	99364	deletion	1
#20#	110261	110408	deletion	148
#20#	115351	115354	deletion	4
#20#	128710	128710	deletion	1
#21#	4576	4576	deletion	1
#21#	11962	11962	deletion	1

#21#	12872	12881	deletion	10
#21#	15176	15180	deletion	5
#21#	15360	15360	deletion	1
#21#	15441	15445	deletion	5
#21#	16894	16897	deletion	4
#21#	18251	18783	deletion	533
#21#	19419	19444	deletion	26
#21#	19756	19772	deletion	17
#21#	20421	20489	deletion	69
#21#	20495	20935	deletion	441
#21#	21281	21281	deletion	1
#21#	21479	21479	deletion	1
#21#	32667	32670	deletion	4
#21#	36629	36629	deletion	1
#21#	49119	49122	deletion	4
#21#	49617	49624	deletion	8
#21#	49680	49680	deletion	1
#21#	49710	49714	deletion	5
#21#	51909	51918	deletion	10
#21#	51958	51958	deletion	1
#21#	54299	54299	deletion	1
#21#	60469	60469	deletion	1
#21#	63154	63154	deletion	1
#21#	66286	66286	deletion	1
#21#	68334	68355	deletion	22
#21#	68810	68820	deletion	11
#21#	69666	69669	deletion	4
#21#	72304	72304	deletion	1
#21#	83928	83928	deletion	1
#21#	84048	84063	deletion	16
#21#	96499	96499	deletion	1
#21#	99938	99938	deletion	1
#21#	109788	109790	deletion	3
#21#	110394	110394	deletion	1
#21#	112985	112988	deletion	4
#21#	115367	115376	deletion	10
#21#	119031	119034	deletion	4
#21#	128132	128132	deletion	1
#21#	131567	131567	deletion	1
#22#	1202	1208	deletion	7
#22#	3643	3650	deletion	8
#22#	4173	4174	deletion	2
#22#	4643	4647	deletion	5
#22#	5103	5121	deletion	19
#22#	5769	5769	deletion	1
#22#	6310	6314	deletion	5
#22#	7732	7738	deletion	7

#22#	7988	7989	deletion	2
#22#	8758	8758	deletion	1
#22#	11945	11955	deletion	11
#22#	12376	12415	deletion	40
#22#	12445	12449	deletion	5
#22#	12459	12459	deletion	1
#22#	15147	15151	deletion	5
#22#	15245	15263	deletion	19
#22#	15770	15771	deletion	2
#22#	16448	16448	deletion	1
#22#	16531	16533	deletion	3
#22#	16899	16902	deletion	4
#22#	17095	17102	deletion	8
#22#	17620	17620	deletion	1
#22#	17911	17915	deletion	5
#22#	18102	18102	deletion	1
#22#	18653	18656	deletion	4
#22#	18913	18913	deletion	1
#22#	19287	19291	deletion	5
#22#	19690	19691	deletion	2
#22#	19813	19813	deletion	1
#22#	20434	20434	deletion	1
#22#	20742	20742	deletion	1
#22#	20885	20889	deletion	5
#22#	21232	21234	deletion	3
#22#	27363	27367	deletion	5
#22#	32651	32659	deletion	9
#22#	32738	32750	deletion	13
#22#	35745	35751	deletion	7
#22#	36138	36138	deletion	1
#22#	36403	36403	deletion	1
#22#	36979	36984	deletion	6
#22#	37668	37668	deletion	1
#22#	39581	39586	deletion	6
#22#	45155	45155	deletion	1
#22#	45662	45667	deletion	6
#22#	45946	45946	deletion	1
#22#	46285	46286	deletion	2
#22#	47062	47062	deletion	1
#22#	47835	47839	deletion	5
#22#	47988	47993	deletion	6
#22#	48352	48356	deletion	5
#22#	50021	50024	deletion	4
#22#	50688	50688	deletion	1
#22#	50990	50990	deletion	1
#22#	51010	51013	deletion	4
#22#	51592	51594	deletion	3

#22#	54100	54120	deletion	21
#22#	55151	55151	deletion	1
#22#	57861	57861	deletion	1
#22#	57982	57987	deletion	6
#22#	58170	58175	deletion	6
#22#	58206	58207	deletion	2
#22#	58241	58250	deletion	10
#22#	60267	60275	deletion	9
#22#	60405	60405	deletion	1
#22#	60476	60479	deletion	4
#22#	60647	60649	deletion	3
#22#	61404	61404	deletion	1
#22#	61669	61732	deletion	64
#22#	63219	63223	deletion	5
#22#	65556	65556	deletion	1
#22#	65805	65810	deletion	6
#22#	65999	65999	deletion	1
#22#	66289	66290	deletion	2
#22#	67619	67620	deletion	2
#22#	67858	67858	deletion	1
#22#	68070	68070	deletion	1
#22#	68307	68327	deletion	21
#22#	68499	68503	deletion	5
#22#	69683	69687	deletion	5
#22#	70277	70278	deletion	2
#22#	70303	70304	deletion	2
#22#	72552	72554	deletion	3
#22#	76912	76912	deletion	1
#22#	77243	77245	deletion	3
#22#	77412	77412	deletion	1
#22#	77456	77457	deletion	2
#22#	79827	79827	deletion	1
#22#	81790	81791	deletion	2
#22#	82128	82132	deletion	5
#22#	83257	83261	deletion	5
#22#	84092	84093	deletion	2
#22#	85790	85797	deletion	8
#22#	90137	90137	deletion	1
#22#	90921	90932	deletion	12
#22#	94665	94665	deletion	1
#22#	95609	95620	deletion	12
#22#	99354	99354	deletion	1
#22#	107425	107429	deletion	5
#22#	109800	109801	deletion	2
#22#	114974	114978	deletion	5
#22#	115425	115429	deletion	5
#22#	115945	115947	deletion	3

#22#	116864	116871	deletion	8
#22#	117494	117494	deletion	1
#22#	118386	118390	deletion	5
#22#	118832	118833	deletion	2
#22#	118975	118975	deletion	1
#22#	119036	119039	deletion	4
#22#	128713	128713	deletion	1
#22#	132434	132445	deletion	12
#22#	133405	133405	deletion	1
#22#	137124	137135	deletion	12
#22#	137929	137929	deletion	1
Total				12592

**Alignment topology with node labels: ((((O.granulata,O.meyeriana)#1#,(O.brachyantha,(O.longiglumis,O.ridleyi)#2#)#9#)#13#,(O.australiensis,(((O.rhizomatis,(O.officinalis,O.eichingeri)#3#)#10#,(O.latifolia,(O.alta,O.grandiglumis)#4#)#11#)#14#,((O.punctata,O.minuta)#5#,(O.longistaminata,(O.meridionalis,(O.glumaepatula,((O.glaberrima,O.barthii)#6#,((O.nivara,O.sativa_ssp.indica)#7#,(O.rufipogon,O.sativa_ssp.japonica)#8#)#12#)#15#)#16#)#17#)#18#)#19#)#20#)#21#)#22#,L.japonica)#23#;*

*** The Starting and ending position of the feature in the Prank+F alignment*

Supplementary Table 7. Statistics of genomic structural variation for the twenty-three *Oryza* plastomes. Putative indels were extracted from the refined sequence alignments with *L. japonica* as outgroup.

	Insertion				Deletion			
**Species**	**Count**	**Sum (bp)**	**Max (bp)**	**Avg (bp)**	**Count**	**Sum (bp)**	**Max (bp)**	**Avg (bp)**
O. granulata	172	7623	926	44	144	3727	748	26
O. meyeriana	171	7810	926	46	142	3723	748	26
O. brachyantha	178	5613	1309	32	166	3367	948	20
O. longiglumis	179	6910	1309	39	149	3222	510	22
O. ridleyi	177	8371	1309	47	148	4604	548	31
O. australiensis	162	8423	1720	52	152	4710	1575	31
O. rhizomatis	167	9599	1721	57	152	6898	1575	45
O. officinalis	170	9681	1720	57	154	6928	1575	45
O. eichingeri	171	9689	1720	57	155	6934	1575	45
O. latifolia	175	9921	1719	57	151	6746	1575	45
O. alta	173	9673	1719	56	152	6540	1575	43
O. grandiglumis	174	9578	1719	55	154	6469	1575	42
O. punctata	176	8005	1309	45	156	5701	758	37
O. minuta	180	8015	1309	45	158	5697	758	36
O. longistaminata	184	9864	1485	54	167	7738	1575	46
O. meridionalis	176	9139	1309	52	161	6919	1382	43
O. glumaepatula	163	8427	1720	52	153	4712	1575	31
O. glaberrima	180	9466	1583	53	162	7155	1668	44
O. barthii	177	9428	1583	53	162	7200	1668	44
O. nivara	170	9572	1720	56	162	6855	1575	42
O. sativa. ssp. *indica*	176	9344	1485	53	162	7200	1575	44
O. rufipogon	173	9237	1485	53	161	7134	1575	44
O. sativa. ssp. *japonica*	210	9447	2088	45	199	7213	1575	36

Supplementary Table 8. Statistics of the detected indels across the twenty-three *Oryza* chloroplast genomes using *L. japonica* as outgroup.

Feature	Insertion		Deletion		Percentage (%)
	No.	Total length (bp)	No.	Total length (bp)	
**Intergenic regions**	574	4051	773	11152	78
**Exon**	32	327	44	610	4
**Intron**	90	358	145	759	14
**RNA Gene**	32	154	39	311	4
**Total**	728	4890	1001	12832	100

Supplementary Table 9. List of the detected indels within exons of 16 genes in the *Oryza* chloroplast genomes.

Gene	Homoplasious	Synapomorphic	Total
accD	0	5	5
atpF	0	1	1
ccsA	2	1	3
ndhD	0	5	5
ndhF	0	2	2
ndhH	1	3	4
ndhI	0	1	1
ndhK	0	1	1
rbcL	0	1	1
rpl22	0	1	1
rpl32	0	2	2
rpoC1	0	2	2
rpoC2	0	8	8
rps3	0	1	1
ycf68	0	4	4
rrn23	0	6	6
**Total**	3	44	47

Supplementary Table 10. The insertion (base) and deletion (-) of the chloroplast genome in the *O. sativa* ssp. *japonica* using *O. punctata* as outgroup.

Size	*O. punctata*	*O. sativa* ssp. *japonica*	Location
2	--	ac	*trnfM-CAU/trnT-GGU*
1	-	c	*trnfM-CAU/trnT-GGU*
1	-	c	*trnC-GCA/rpoB*
1	-	a	*atpH/atpF*
1	-	t	*ycf3*
1	-	c	*ycf3*
1	-	c	*ycf3*
2	--	ca	*ycf3*
1	-	c	*ycf3*
1	-	a	*ycf3*
1	-	t	*ycf3*
3	---	tcg	*ycf3*
1	-	c	*ycf3/trnS-GGA*
1	-	a	*ycf3/trnS-GGA*
1	-	t	*trnV-UAC*
1	-	a	*atpB/rbcL*
1	-	t	*atpB/rbcL*
16	----------------	actttttttttagaat	*accD/psaI*
1	-	a	*accD/psaI*
1	-	g	*accD/psaI*
1	-	c	*cemA/petA*
1	-	a	*cemA/petA*
1	-	g	*petA/psbJ*
1	-	c	*rps12_5end/clpP*
1	-	g	*clpP/psbB*
1	-	c	*petB*
1	-	a	*petB/petD*
1	-	t	*petD*
1	-	a	*rpl36/infA*
1	-	c	*rpl16*
2	--	ga	*ndhB/rps7*
3	---	att	*ndhB/rps7*
1	-	t	*rps12_3end/trnV-GAC*
1	-	a	*rps12_3end/trnV-GAC*
1	-	g	*trnA-UGC*
1	-	a	*rrn23*
1	-	c	*ndhF*
1	t	-	*rps16*
1	c	-	*trnS-GCU/psbD*
1	g	-	*trnfM-CAU/trnT-GGU*
1	t	-	*trnfM-CAU/trnT-GGU*

1	g	-	*atpF*
2	gt	--	*ycf3*
1	t	-	*trnL-UAA/trnF-GAA*
1	g	-	*ndhC/trnV-UAC*
2	gc	--	*trnV-UAC*
1	g	-	*atpB/rbcL*
15	cgaattcctatagta	---------------	*atpB/rbcL*
1	g	-	*rbcL/accD*
1	g	-	*accD/psaI*
1	g	-	*ycf4/cemA*
1	a	-	*ycf4/cemA*
1	g	-	*clpP/psbB*
2	at	--	*clpP/psbB*
1	t	-	*petB*
1	g	-	*petB*
1	g	-	*petB*
1	t	-	*petD*
1	g	-	*petD*
1	g	-	*petD*
1	g	-	*petD*
1	g	-	*rps8*
1	g	-	*rpl16*
1	c	-	*rps12_3end/trnV-GAC*
1	g	-	*rps12_3end/trnV-GAC*
1	a	-	*rps12_3end/trnV-GAC*
1	c	-	*rrn23*
1	g	-	*rrn23*
2	cc	--	*rrn23*
1	c	-	*rrn23*
2	gg	--	*rrn4.5/trnR-ACG*
1	g	-	*trnR-ACG/trnN-GUU*
1	c	-	*trnN-GUU/rps15*
1	c	-	*ndhF*
1	t	-	*ndhF/rpl32*
4	aaca	----	*rpl32*
6	ataact	------	*ndhI*
1	c	-	*ndhA*
1	c	-	*rps7/ndhB*

Supplementary Table 11. Summary of repeat sequences detected in the 24 *Oryzeae* chloroplast genomes.

Repeat Type	Length (bp)	Repeat Sequence	Location	Shared Chloroplast Genomes
tandem	18	TATATGAATGAAACATA	*atpB*/*rbcL*	*O. sativa* ssp. *japonica, O. rufipogon, O. sativa* ssp. *indica, O. nivara, O. glaberrima, O. barthii, O. glumaepatula, O. meridionalis, O. longistaminata, O. punctata, O. minuta, O. officinalis, O. eichingeri, O. alta, O. grandiglumis, O. latifolia, O. australiensis,*
tandem	16	CTTTTTTTTTAGAATA	*accD*/*psaI*	*O. sativa* ssp. *japonica,*
tandem	21	TAAATCCAAGCAAACTTTTCG	*rps18**	*O. sativa* ssp. *japonica, O. rufipogon, O. sativa* ssp. *indica, O. nivara, O. glaberrima, O. barthii, O. glumaepatula, O. meridionalis, O. longistaminata, O. punctata, O. minuta, O. officinalis, O. rhizomatis, O. eichingeri, O. alta, O. grandiglumis, O. latifolia, O. australiensis, O. longiglumis, O. ridleyi,*
tandem	26	ATTTTTTTAGCATTTTTATTTAAATA	*matK*/*rps16*	*O. rufipogon*
tandem	32	TTAACAAATTCTTAGAGTATTTCTGGTAGAAT	*petN*/*trnC*	*O. rufipogon, O. sativa* ssp. *indica, O. nivara*
tandem	34	TCAAATAATAAATAGTATTCATGTTAATATTTCA	*psaC*/*ndhE*	*O. glaberrima, O. barthii*
tandem	23	GAGAAGAGGTACCTGCTGAAAAT	*trnfM*/*trnT*	*O. glumaepatula*
tandem	57	AGAGGAAGACTCAGAGGACGAACATGGGACTTTAGAGGAAGACTCAGAGGAAGACTC	*rpoC2**	*O. glumaepatula*
tandem	20	TATATAGAAATAGAAAGAAT	*psbM*/*petN*	*O. meridionalis*
tandem	19	TATGAATATACAATCTAGA	*atpI*/*atpH*	*O. punctata, O. minuta, O. eichingeri, O. latifolia, O.grandiglumis, O. alta*
tandem	15	AGTATTCTAAAAAAA	*accD*/*psaI*	*O. punctata*
tandem	21	TTTCATAAATCCAAGCAAACT	*rps18**	*O. punctata, O. minuta, O. officinalis, O. rhizomatis, O. eichingeri, O. alta, O. grandiglumis, O. latifolia, O. australiensis,*

				O. brachyantha, O. longiglumis, O. ridleyi, L. japonica
tandem	26	ATATCTGTATATGGAGAGTATACTTG	*ndhF*/*rpl32*	*O. punctata, O. minuta, O. alta, O. grandiglumis, O. latifolia*
tandem	22	AGAGAGAGGAAAGCAAAAGGAG	*psbC*/*trnS**	*O. officinalis, O. rhizomatis, O. eichingeri, O. alta, O. grandiglumis, O. latifolia*
tandem	24	ACTATATGTAGTTTTTTCTACCAC	*psbM*/*petN*	*O. officinalis, O. rhizomatis, O. eichingeri*
tandem	23	CACGTCTACTCTTGTTAGTAGAA	*atpA*/*trnR*	*O. officinalis, O. rhizomatis, O. eichingeri, O. alta, O. grandiglumis, O. latifolia*
tandem	18	ATTAGTACTATATCAATC	*psaC*/*ndhE*	*O. officinalis, O. rhizomatis, O. eichingeri*
tandem	19	ATTTTTTTGTATATTGTAT	*ndhG*/*ndhI*	*O. officinalis*
tandem	24	TATGTAGTTTTTTCTACCACACTT	*psbM*/*petN*	*O. alta*
tandem	31	TCTCCTATTTTTCTTTTCTGTCATATTTTTT	*infA**/*rps8*	*O. latifolia*
tandem	17	TTTCGATTTCTATATTA	*rps16* intron	*O. australiensis*
tandem	19	TTAATGCAAAATGGTCAAT	*psbM*/*petN*	*O. australiensis*
tandem	33	GAGGACGAATATAGGACTCGAGAGGAGGAATCC	*rpoC2**	*O. australiensis*
tandem	20	ATTCTATATGAATACTATAG	*clpP*/*psbB*	*O. australiensis*
tandem	34	AACGAATAAAACCCCTTGTCGAAGCCCTTTATGA	*trnI*/*trnL*	*O. australiensis*
tandem	34	TCATAAAGGGCTTCGACAAGGGGTTTTATTCGTT	*trnL*/*trnI*	*O. australiensis*
tandem	21	TTAATCCAAAATCAAGAATAA	*trnK* intron	*O. brachyantha*
tandem	21	ATACAATACGATTTCATCTTT	*rps16*/*trnQ*	*O. brachyantha*
tandem	15	ATAGAAATAGAAAGA	*psbM*/*petN*	*O. brachyantha*
tandem	17	TTTATTATTTTTACAAA	*petA*/*psbJ*	*O. brachyantha*
tandem	21	ATATTTTATTATCTCATAAAT	*rps11*/*rpl36*	*O. brachyantha*

tandem	34	TTTTATTAGTAGTCTTATAGTAGTCTTAGATTTT	*rps15/ndhH*	*O. brachyantha*
tandem	27	GCAGTAGGTATTCGTTTTTCTTTCTAG	*ndhG/ndhI*	*O. brachyantha*
tandem	34	AAAATCTAAGACTACTATAAGACTACTAATAAAA	*ndhH/rps15*	*O. brachyantha*
tandem	21	ATATAGGACCCTAGAGGACGA	*rpoC2**	*O. longiglumis*
tandem	26	AGTTTTGTTTAATGATTAATTAAGAG	*trnK* intron	*O. longiglumis, O. ridleyi*
tandem	17	TCTAATCGCTCTTTCTA	*psbM/petN*	*O. longiglumis, O. ridleyi, O. granulata, O. meyeriana, L. japonica*
tandem	27	AATTTTTCCCATTATTTGACTATCCAT	*rps12_3end/trnV*	*O. longiglumis, O. ridleyi*
tandem	27	ATTATGGATAGTCAAATAATGGGAAAA	*trnV/rps12_3end*	*O. longiglumis, O. ridleyi*
tandem	17	TATCTCTATCATAAATC	*rps16/trnQ*	*O. granulata, O. meyeriana*
tandem	19	AATACTATAGATTCTATTA	*clpP/psbB*	*O. granulata, O. meyeriana*
tandem	20	ATAGTAGAGGATATCTGTTT	*trnV/trnM*	*O. granulata, O. meyeriana*
tandem	100	ATCTTGAAAGTGAATCATATTCCATGAATATGATATCTATCTAATGTGATATCTTGAAAGTGAATCGTATTCCATGAATATGATATCTATCTAATGTGAT	*trnL/ndhB*	*O. meyeriana*
tandem	100	ATATCACATTAGATAGATATCATATTCATGGAATACGATTCACTTTCAAGATATCACATTAGATAGATATCATATTCATGGAATA	*ndhB/trnL*	*O. meyeriana*

		TGATTCACTTTCAAG		
tandem	30	GTATACTGTATCCATGTATACAGGATCCGC	*trnS*/*psbZ*	*L. japonica*
tandem	19	AATGATCATTATTTGCTTT	*trnfM*/*trnT*	*L. japonica*
dispersed	43	CCTCTACCATGTCTAGACAAATAGAATAGTTATTTTATACAGA	*trnG*/*trnfM*, *trnfM*/*trnT*	*O. sativa* ssp. *japonica*
dispersed	32	ATGGAATCAAATACGCAGTATTTACAGAAAAA	*rbcL*/*accD*, *rpl23**	*O. sativa* ssp.*japonica, O. rufipogon, O. sativa* ssp. *indica, O. nivara, O. glaberrima, O. barthii, O. glumaepatula, O. meridionalis, O. longistaminata, O. officinalis, O. rhizomatis, O. eichingeri, O. alta, O. grandiglumis, O. latifolia, O. australiensis, O. granulata*
dispersed	38	GACATACAATGCATTACAGACGTATGATCATTACCCTT	*rbcL*/*accD*, *rpl23**	*O. sativa* ssp.*japonica, O. rufipogon, O. sativa* ssp. *indica, O. nivara, O. glaberrima, O. barthii, O. glumaepatula, O. meridionalis, O. longistaminata*
dispersed	52	AACCGGGTTATTCTATTCCACTTCTAGATAGAGAAAAAAACTAAAGGAGAAT	*rbcL*/*accD*, *rpl23**	*O. sativa* ssp.*japonica, O. rufipogon, O. sativa* ssp. *indica, O. nivara, O. glaberrima, O. barthii, O. glumaepatula, O. meridionalis*
dispersed	46	CTTAGGTTTCCGAAATAGTGTAATGGAAAAAGAAGTGCTTCGAATC	*psbE*/*petL*, *rps7*/*rps12_3end*	*O. sativa* ssp.*japonica, O. rufipogon, O. sativa* ssp. *indica, O. nivara, O. glaberrima, O. barthii, O. glumaepatula, O. meridionalis, O. longistaminata, O. punctata, O. minuta, O. officinalis, O. rhizomatis, O. eichingeri, O. alta, O. grandiglumis, O. latifolia*
dispersed	35	TATTCCATGAATATGATATCTATCTAATGTGATAT	*trnL*/*ndhB*	*O. sativa* ssp.*japonica, O. rufipogon, O. sativa* ssp. *indica, O. nivara, O. glaberrima, O. barthii, O. glumaepatula, O. meridionalis, O. longistaminata, O. punctata, O. minuta, O. officinalis, O. rhizomatis, O. eichingeri, O. alta, O. grandiglumis, O. latifolia, O. australiensis, O. brachyantha, O. ridleyi, O. granulata, O. meyeriana, L. japonica*
dispersed	30	TTCTTTTTCGAAATCGATTTCGAAAAAGAA	*ndhB* intron	*O. sativa* ssp.*japonica, O. rufipogon, O. sativa* ssp. *indica, O. nivara, O. glaberrima, O. barthii, O. glumaepatula, O. meridionalis, O. longistaminata, O. officinalis, O. rhizomatis, O. eichingeri, O. alta, O. grandiglumis, O. latifolia, O. australiensis, O. brachyantha, O. longiglumis, O. ridleyi, O. granulata, O. meyeriana, L. japonica*
dispersed	35	ATATCACATTAGATAGATATCATAT	*ndhB*/*trnL*	*O. sativa* ssp.*japonica, O. rufipogon, O. sativa* ssp. *indica, O. nivara, O. glaberrima, O. barthii, O. glumaepatula, O.*

		TCATGGAATA		*meridionalis, O. longistaminata, O. punctata, O. minuta, O. officinalis, O. rhizomatis, O. eichingeri, O. alta, O. grandiglumis, O. latifolia, O. australiensis, O. brachyantha, O. ridleyi, O. granulata, O. meyeriana, L. japonica*
dispersed	34	AGAGGAAGACTCAGAGGAAGACTCAGAGGACGAA	*rpoC2**	*O. glumaepatula*
dispersed	39	AACCGGGTTATTCTATTCCACTTCTAGATAGAGAAAAAA	*rbcL*/*accD, rpl23**	*O. longistaminata*
dispersed	36	AACCGTACATGAGATTTTCACCTCATACGGCTCCTC	*ycf3, rps12_3end*/*trnV*	*O. punctata, O. minuta*
dispersed	72	AACCGGGTTATTCTATTCCACTTCTAGATAGAGAAAAAAACTAAAGGAGAATACGTATGATCATTACCCTTC	*rbcL*/*accD, rpl23**	*O. punctata, O. minuta, O. officinalis, O. rhizomatis, O. eichingeri, O. alta, O. grandiglumis, O. latifolia,*
dispersed	30	GCAATAGCTAAATGATGGTGCGCAATATCG	*psaB*, psaA**	*O. australiensis*
dispersed	69	AACCGGGTTATTCTATTCCACTTCTAGATAGAGAAAAAAACTAAAGGAGAATTATGATCATTACCCTTC	*rbcL*/*accD, rpl23**	*O. australiensis*
dispersed	31	AGTGTAATGGAAAAAGAAGTGCTTCGAATCA	*psbE*/*petL, rps7*/*rps12_3end*	*O. australiensis*
dispersed	30	CCTAGAGGACGAATATAGGACTCGAGAGGA	*rpoC2**	*O. brachyantha, O. longiglumis, O. ridleyi, O. granulata, O. meyeriana*
dispersed	38	ACGTATGATCATTACCCTTCAACCGGGTTATTCTATTC	*rbcL*/*accD, rpl23**	*O. brachyantha*
disperse	55	AACCGGGTTATT	*rbcL*/*acc*	*O. granulata, O. meyeriana*

d		CTATTCCACTTC TAGATAGAGAA AAAAACTAAAG GAGAATTTC	*D, rpl23**	
disperse d	49	TATTCCATGAAT ATGATATCTATCT AATGTGATATCT TGAAAGTGAAT C	*trnL/ndh B*	*O. meyeriana*
disperse d	49	TATTCCATGAAT ATGATATCTATCT AATGTGATATCT TGAAAGTGAAT C	*trnL/ndh B*	*O. meyeriana*
disperse d	49	ATATCACATTAG ATAGATATCATAT TCATGGAATAGA TTCACTTTCAAG	*ndhB/trn L*	*O. meyeriana*
disperse d	49	ATATCACATTAG ATAGATATCATAT TCATGGAATAGA TTCACTTTCAAG	*ndhB/trn L*	*O. meyeriana*
disperse d	31	AATATGGGACTT TAGAGGAAGAC TCAGAGGA	*rpoC2**	*L. japonica*
disperse d	53	CGAGTCCTTAG GTTTCCGAAATA GTGTAATGGAA AAAAGAAGTGC TTCGAATC	*psbE/pet L, rps7/rps1 2_3end*	*L. japonica*
disperse d	77	GACATACAATGC ATTACAAACGTA TGATCATTACCC TTCAACCGGGTT ATTTTATTCCAC TTCTAGATAGAG AAAAA	*rbcL/acc D, rpl23**	*L. japonica*
panlindr omic	20	TTCTTATTCTTTT TTCTTTC	*rps16* intron, *ndhF**	*O. sativa* ssp.*japonica*, *O. rufipogon*, *O. sativa* ssp. *indica*, *O. nivara*, *O. glaberrima*, *O. barthii*, *O. glumaepatula*, *O. meridionalis*, *O. longistaminata*, *O. punctata*, *O. minuta*, *O. officinalis*, *O. rhizomatis*, *O. eichingeri*, *O. alta*, *O. grandiglumis*, *O. latifolia*, *O. longiglumis*, *O. ridleyi*, *O. granulata*, *O. meyeriana*
panlindr omic	29	AGAGAGGGATT CGAACCCTCGC	*trnS**	*O. sativa* ssp.*japonica*, *O. rufipogon*, *O. sativa* ssp. *indica*, *O. nivara*, *O. glaberrima*, *O. barthii*, *O. glumaepatula*, *O.*

		GGAAGTA		*meridionalis, O. longistaminata, O. punctata, O. minuta, O. officinalis, O. rhizomatis, O. eichingeri, O. alta, O. grandiglumis, O. latifolia, O. australiensis, O. brachyantha, O. longiglumis, O. ridleyi, O. granulata, O. meyeriana, L. japonica*
panlindromic	21	AGAGAGGGATTCGAACCCTCG	*trnS**	*O. sativa* ssp.*japonica, O. rufipogon, O. sativa* ssp. *indica, O. nivara, O. glaberrima, O. barthii, O. glumaepatula, O. meridionalis, O. longistaminata, O. punctata, O. minuta, O. officinalis, O. rhizomatis, O. eichingeri, O. alta, O. grandiglumis, O. latifolia, O. australiensis, O. brachyantha, O. longiglumis, O. ridleyi, O. granulata, O. meyeriana, L. japonica*
panlindromic	21	GTCATCGGTTCAAATCCGATA	*trnT**	*O. sativa* ssp.*japonica, O. rufipogon, O. sativa* ssp. *indica, O. nivara, O. glaberrima, O. barthii, O. glumaepatula, O. meridionalis, O. longistaminata, O. punctata, O. minuta, O. officinalis, O. rhizomatis, O. eichingeri, O. alta, O. grandiglumis, O. latifolia, O. australiensis, O. longiglumis, O. ridleyi, O. granulata, O. meyeriana, L. japonica*
panlindromic	22	TAAAGTGTGGTAGAAAAAACTA	*psbM*/*petN*	*O. sativa* ssp.*japonica, O. rufipogon, O. sativa* ssp. *indica, O. nivara, O. glaberrima, O. barthii, O. glumaepatula, O. meridionalis, O. longistaminata, O. punctata, O. minuta, O. officinalis, O. rhizomatis, O. eichingeri, O. alta, O. grandiglumis, O. latifolia*
panlindromic	23	TTTCTATTCTTTCTATTTCTATT	*psbM*/*petN*	*O. sativa* ssp. *japonica, O. glaberrima*
panlindromic	22	TTTCTATTCTTTCTATTTCTAT	*psbM*/*petN*	*O. sativa* ssp. *japonica, O. rufipogon, O. sativa* ssp. *indica, O. nivara, O. glaberrima, O. glumaepatula,*
panlindromic	32	ATGGAATCAAATACGCAGTATTTACAGAAAAA	*rbcL*/*accD, rpl23**	*O. sativa* ssp.*japonica, O. rufipogon, O. sativa* ssp. *indica, O. nivara, O. glaberrima, O. barthii, O. glumaepatula, O. meridionalis, O. longistaminata, O. officinalis, O. rhizomatis, O. eichingeri, O. alta, O. grandiglumis, O. latifolia, O. granulata, O. meyeriana*
panlindromic	20	TTTATTGGGAAAGAATCAAT	*rbcL*/*accD, rpl23**	*O. sativa* ssp.*japonica, O. rufipogon, O. sativa* ssp. *indica, O. nivara, O. glaberrima, O. barthii, O. glumaepatula, O. meridionalis, O. longistaminata, O. punctata, O. minuta, O. australiensis, O. longiglumis, O. ridleyi, O. granulata, O. meyeriana*
panlindromic	38	GACATACAATGCATTACAGACGTATGATCATTACCCTT	*rbcL*/*accD, rpl23**	*O. sativa* ssp.*japonica, O. rufipogon, O. sativa* ssp. *indica, O. nivara, O. glaberrima, O. barthii, O. glumaepatula, O. meridionalis, O. longistaminata,*
panlindromic	52	AACCGGGTTATTCTATTCCACTTCTAGATAGAGAAAAAAACTAAAGGAGAAT	*rbcL*/*accD, rpl23**	*O. sativa* ssp.*japonica, O. rufipogon, O. sativa* ssp. *indica, O. nivara, O. glaberrima, O. barthii, O. glumaepatula, O. meridionalis, O. punctata, O. minuta, O. officinalis, O. rhizomatis, O. eichingeri, O. alta, O. grandiglumis, O. latifolia, O. australiensis, O. granulata, O. meyeriana*

panlindromic	20	CTTTTTTTTTAGAATACTTT	*accD*/*psaI*,	*O. sativa* ssp. *japonica*
panlindromic	25	TAGAATACTTTTTTTTTAGAATACT	*accD*/*psaI*,	*O. sativa* ssp. *japonica*
panlindromic	46	CTTAGGTTTCCGAAATAGTGTAATGGAAAAAGAAGTGCTTCGAATC	*psbE*/*petL*, *rps12_3end*/*rps7*	*O. sativa* ssp.*japonica, O. rufipogon, O. sativa* ssp. *indica, O. nivara, O. glaberrima, O. barthii, O. glumaepatula, O. meridionalis, O. longistaminata, O. punctata, O. minuta, O. officinalis, O. rhizomatis, O. eichingeri, O. alta, O. grandiglumis, O. latifolia*
panlindromic	22	TAATTGAAGTAAGAAGTCTCCC	*psbT*/*psbN*	*O. sativa* ssp.*japonica, O. rufipogon, O. sativa* ssp. *indica, O. nivara, O. glaberrima, O. barthii, O. glumaepatula, O. meridionalis, O. longistaminata, O. punctata, O. minuta, O. officinalis, O. rhizomatis, O. eichingeri, O. alta, O. grandiglumis, O. latifolia, O. australiensis, O. brachyantha, O. longiglumis, O. ridleyi, O. granulata, O. meyeriana, L. japonica*
panlindromic	25	ATTCTTTTATTTTAGATAGAAGAAA	*trnN*/*rps15*	*O. sativa* ssp.*japonica, O. rufipogon, O. sativa* ssp. *indica, O. nivara, O. glaberrima, O. barthii, O. glumaepatula, O. meridionalis, O. longistaminata, O. punctata, O. minuta, O. officinalis, O. rhizomatis, O. eichingeri, O. alta, O. grandiglumis, O. latifolia, O. australiensis, O. brachyantha, O. longiglumis, O. ridleyi, O. granulata, O. meyeriana, L. japonica*
panlindromic	22	TTTTCACCTCATACGGCTCCTC	*ycf3* intron, *trnV*/*rps12_3end*	*O. rufipogon, O. sativa* ssp. *indica, O. nivara, O. glaberrima, O. barthii, O. glumaepatula, O. meridionalis, O. longistaminata, O. punctata, O. minuta, O. officinalis, O. rhizomatis, O. eichingeri, O. alta, O. grandiglumis, O. latifolia, O. australiensis*
panlindromic	28	TTTCTATTCTTTCTATTTCTATTTCTAT	*psbM*/*petN*	*O. glaberrima*
panlindromic	39	AACCGGGTTATTCTATTCCACTTCTAGATAGAGAAAAAA	*rbcL*/*accD, rpl23**	*O. sativa* ssp.*japonica, O. rufipogon, O. sativa* ssp. *indica, O. nivara, O. glaberrima, O. barthii, O. glumaepatula, O. meridionalis, O. longistaminata, O. punctata, O. minuta, O. officinalis, O. rhizomatis, O. eichingeri, O. alta, O. grandiglumis, O. latifolia, O. australiensis, O. granulata, O. meyeriana*
panlindromic	36	AACCGTACATGAGATTTTCACCTCATACGGCTCCTC	*ycf3* intron, *trnV*/*rps12_3end*	*O. punctata, O. minuta*
panlindromic	20	CTAGTAAAAAAATCCTGGA	*ycf3* intron, *rps4**	*O. punctata, O. minuta*
panlindromic	72	ACGTATGATCATTACCCTTCAACCGGGTTATTCTAT	*rbcL*/*accD*, *rpl2*/*rpl2*	*O. punctata, O. minuta, O. officinalis, O. rhizomatis, O. eichingeri, O. alta, O. grandiglumis, O. latifolia*

		TCCACTTCTAGATAGAGAAAAAAACTAAAGGAGAAT	*3**	
panlindromic	24	TAAAGTGTGGTAGAAAAAACTACA	*psbM*/*petN*	*O. alta*
panlindromic	21	GACATACAATGCATTACAAAC	*rbcL*/*accD, rpl23**	*O. australiensis, L. japonica*
panlindromic	69	TATGATCATTACCCTTCAACCGGGTTATTCTATTCCACTTCTAGATAGAGAAAAAAACTAAAGGAGAAT	*rbcL*/*accD, rpl2*/*rpl23**	*O. punctata, O. minuta, O. officinalis, O. rhizomatis, O. eichingeri, O. alta, O. grandiglumis, O. latifolia, O. australiensis*
panlindromic	31	AGTGTAATGGAAAAAGAAGTGCTTCGAATCA	*psbE*/*petL, rps12_3end*/*rps7*	*O. australiensis*
panlindromic	24	TAAAGTGTGGTAGAAAGAACTACA	*psbM*/*petN*	*O. brachyantha, O. longiglumis, O. ridleyi, O. granulata, O. meyeriana,*
panlindromic	23	ATGGAATCAAATACGCAGTATTT	*rbcL*/*accD, rpl23**	*O. sativa* ssp.*japonica, O. rufipogon, O. sativa* ssp. *indica, O. nivara, O. glaberrima, O. barthii, O. glumaepatula, O. meridionalis, O. longistaminata, O. officinalis, O. rhizomatis, O. eichingeri, O. alta, O. grandiglumis, O. latifolia, O. brachyantha, O. longiglumis, O. ridleyi, O. granulata, O. meyeriana*
panlindromic	25	GCAGTATTTACAGAAAAAAGTCTTC	*rbcL*/*accD, rpl23**	*O. brachyantha, O. longiglumis, O. ridleyi*
panlindromic	38	ACGTATGATCATTACCCTTCAACCGGGTTATTCTATTC	*rbcL*/*accD, rpl23**	*O. punctata, O. minuta, O. officinalis, O. rhizomatis, O. eichingeri, O. alta, O. grandiglumis, O. latifolia, O. brachyantha*
panlindromic	20	ACTTCTAGATAGAGAAAAAA	*rbcL*/*accD, rpl23**	*O. sativa* ssp.*japonica, O. rufipogon, O. sativa* ssp. *indica, O. nivara, O. glaberrima, O. barthii, O. glumaepatula, O. meridionalis, O. longistaminata, O. punctata, O. minuta, O. officinalis, O. rhizomatis, O. eichingeri, O. alta, O. grandiglumis, O. latifolia, O. australiensis, O. brachyantha, O. granulata, O. meyeriana*
panlindromic	20	AACCGTACATGAGATTTTCA	*ycf3* intron, *trnV*/*rps12_3end*	*O. punctata, O. minuta, O. longiglumis, O. ridleyi*

panlindromic	26	TTCAACCGGGTTATTCTATTCCACTT	*rbcL*/*accD*, *rpl23**	*O. punctata, O. minuta, O. officinalis, O. rhizomatis, O. eichingeri, O. alta, O. grandiglumis, O. latifolia, O. australiensis, O. longiglumis, O. ridleyi, O. granulata, O. meyeriana*
panlindromic	20	CCAGTCACTACGGAATCTCG	*ndhD**	*O. longiglumis*
panlindromic	26	TCTAAAGTGTGGTAGAAAGAACTACA	*psbM*/*petN*	*O. granulata, O. meyeriana*
panlindromic	35	ATGGAATCAAATACGCAGTATTTACAGAAAAAAAGT	*rbcL*/*accD*, *rpl23**	*O. granulata, O. meyeriana*
panlindromic	55	TTCAACCGGGTTATTCTATTCCACTTCTAGATAGAGAAAAAAACTAAAGGAGAAT	*rbcL*/*accD*, *rpl23**	*O. punctata, O. minuta, O. officinalis, O. rhizomatis, O. eichingeri, O. alta, O. grandiglumis, O. latifolia, O. australiensis, O. granulata, O. meyeriana*
panlindromic	21	TGTTTCTTTATTTGTTTTTTT	*psbC*/*trnS*	*L. japonica*
panlindromic	22	TAAAGTGTGGTAGAAAGAACTA	*psbM*/*petN*	*O. brachyantha, O. longiglumis, O. ridleyi, O. granulata, O. meyeriana, L. japonica*
panlindromic	77	GACATACAATGCATTACAAACGTATGATCATTACCCTTCAACCGGGTTATTTTATTCCACTTCTAGATAGAGAAAAA(N26665)	*rbcL*/*accD*, *rpl23**	*L. japonica*
panlindromic	53	CGAGTCCTTAGGTTTCCGAAATAGTGTAATGGAAAAAAGAAGTGCTTCGAATC(N62739)	*psbE*/*petL*, *rps12_3end*/*rps7*	*L. japonica*

Note: Locations of repeat sequences within intergenic regions (with '/'), exons (marked as *) and introns.

Supplementary Table 12. Comparisons of repeat sequences across the *Oryza* plastomes using *L. japonica* as outgroup.

No.	Repeat Type	Length (bp)	Repeat Sequence	Location
Rt2	tandem	16	CTTTTTTTTTAGAATA	*accD*/*psaI*
Rd2	dispersed	43	CCTCTACCATGTCTAGACAAATAGAATAGTTATTTT ATACAGA	*trnG*/*trnfM*, *trnfM*/*trnT*
Rp14	panlindromic	20	CTTTTTTTTTAGAATACTTT	*accD*/*psaI*,
Rp15	panlindromic	25	TAGAATACTTTTTTTTTAGAATACT	*accD*/*psaI*,
Rt4	tandem	26	ATTTTTTTAGCATTTTTATTTAAATA	*matK*/*rps16*
Rp8	panlindromic	28	TTTCTATTCTTTCTATTTCTATTTCTAT	*psbM*/*petN*
Rt7	tandem	23	GAGAAGAGGTACCTGCTGAAAAT	*trnfM*/*trnT*
Rt8	tandem	57	AGAGGAAGACTCAGAGGACGAACATGGGACTTTA GAGGAAGACTCAGAGGAAGACTC	*rpoC2**
Rd10	dispersed	34	AGAGGAAGACTCAGAGGAAGACTCAGAGGACGAA	*rpoC2**
Rt9	tandem	20	TATATAGAAATAGAAAGAAT	*psbM*/*petN*
Rp11	panlindromic	39	AACCGGGTTATTCTATTCCACTTCTAGATAGAGAA AAAA	*rbcL*/*accD*, *rpl23**
Rt11	tandem	15	AGTATTCTAAAAAAA	*accD*/*psaI*
Rt19	tandem	24	TATGTAGTTTTTTCTACCACACTT	*psbM*/*petN*
Rt20	tandem	31	TCTCCTATTTTTCTTTTCTGTCATATTTTTT	*infA**/*rps8*
Rt18	tandem	19	ATTTTTTTGTATATTGTAT	*ndhG*/*ndhI*
Rt21	tandem	17	TTTCGATTTCTATATTA	*rps16*
Rt22	tandem	19	TTAATGCAAAATGGTCAAT	*psbM*/*petN*
Rt23	tandem	33	GAGGACGAATATAGGACTCGAGAGGAGGAATCC	*rpoC2**
Rt24	tandem	20	ATTCTATATGAATACTATAG	*clpP*/*psbB*
Rt25	tandem	34	AACGAATAAAACCCCTTGTCGAAGCCCTTTATGA	*trnI*/*trnL*
Rt26	tandem	34	TCATAAAGGGCTTCGACAAGGGGTTTTATTCGTT	*trnL*/*trnI*
Rd13	dispersed	30	GCAATAGCTAAATGATGGTGCGCAATATCG	*psaB**, *psaA**
Rd14	dispersed	31	AGTGTAATGGAAAAAGAAGTGCTTCGAATCA	*psbE*/*petL*, *rps7*/*rps12_3end*
Rp12	panlindromic	69	AACCGGGTTATTCTATTCCACTTCTAGATAGAGAA AAAAACTAAAGGAGAATTATGATCATTACCCTTC	*rbcL*/*accD*, *rpl23**
Rt35	tandem	21	ATATAGGACCCTAGAGGACGA	*rpoC2**
Rp26	panlindromic	20	CCAGTCACTACGGAATCTCG	*ndhD**
Rt27	tandem	21	TTAATCCAAAATCAAGAATAA	*trnK*
Rt28	tandem	21	ATACAATACGATTTCATCTTT	*rps16*/*trnQ*
Rt29	tandem	15	ATAGAAATAGAAAGA	*psbM*/*petN*
Rt30	tandem	17	TTTATTATTTTTACAAA	*petA*/*psbJ*
Rt31	tandem	21	ATATTTTATTATCTCATAAAT	*rps11*/*rpl36*
Rt32	tandem	34	TTTTATTAGTAGTCTTATAGTAGTCTTAGATTTT	*rps15*/*ndhH*
Rt33	tandem	27	GCAGTAGGTATTCGTTTTTCTTTCTAG	*ndhG*/*ndhI*

Rt34	tandem	34	AAAATCTAAGACTACTATAAGACTACTAATAAAA	*ndhH*/*rps15*
Rd16	dispersed	38	ACGTATGATCATTACCCTTCAACCGGGTTATTCTATTC	*rbcL*/*accD*, *rpl23**
Rt43	tandem	100	ATCTTGAAAGTGAATCATATTCCATGAATATGATATCTATCTAATGTGATATCTTGAAAGTGAATCGTATTCCATGAATATGATATCTATCTAATGTGAT	*trnL*/*ndhB*
Rt44	tandem	100	ATATCACATTAGATAGATATCATATTCATGGAATACGATTCACTTTCAAGATATCACATTAGATAGATATCATATTCATGGAATATGATTCACTTTCAAG	*ndhB*/*trnL*
Rd1	dispersed	49	TATTCCATGAATATGATATCTATCTAATGTGATATCTTGAAAGTGAATC	*trnL*/*ndhB*
Rt45	tandem	30	GTATACTGTATCCATGTATACAGGATCCGC	*trnS*/*psbZ*
Rt46	tandem	19	AATGATCATTATTTGCTTT	*trnfM*/*trnT*
Rd6	dispersed	53	CGAGTCCTTAGGTTTCCGAAATAGTGTAATGGAAAAAAGAAGTGCTTCGAATC	*psbE*/*petL*, *rps7*/*rps12_3end*
Rd17	dispersed	31	AATATGGGACTTTAGAGGAAGACTCAGAGGA	*rpoC2**
Rp27	panlindromic	77	GACATACAATGCATTACAAACGTATGATCATTACCCTTCAACCGGGTTATTTTATTCCACTTCTAGATAGAGAAAAA	*rbcL*/*accD*, *rpl23**
Rp28	panlindromic	21	TGTTTCTTTATTTGTTTTTTT	*psbC*/*trnS*
Rt6	tandem	34	TCAAATAATAAATAGTATTCATGTTAATATTTCA	*psaC*/*ndhE*
Rt5	tandem	32	TTAACAAATTCTTAGAGTATTTCTGGTAGAAT	*petN*/*trnC*
Rp7	panlindromic	22	TTTCTATTCTTTCTATTTCTAT	*psbM*/*petN*
Rp9	panlindromic	52	AACCGGGTTATTCTATTCCACTTCTAGATAGAGAAAAAAACTAAAGGAGAAT	*rbcL*/*accD*, *rpl23**
Rd4	dispersed	38	GACATACAATGCATTACAGACGTATGATCATTACCCTT	*rbcL*/*accD*, *rpl23**
Rd11	dispersed	36	AACCGTACATGAGATTTTCACCTCATACGGCTCCTC	*ycf3*, *trnV*/*rps12_3end*
Rp16	panlindromic	20	CTAGTAAAAAAAATCCTGGA	*ycf3*, *rps4**
Rt15	tandem	24	ACTATATGTAGTTTTTTCTACCAC	*psbM*/*petN*
Rt17	tandem	18	ATTAGTACTATATCAATC	*psaC*/*ndhE*
Rt14	tandem	22	AGAGAGAGGAAAGCAAAAGGAG	*psbC*/*trnS**
Rt16	tandem	23	CACGTCTACTCTTGTTAGTAGAA	*atpA*/*trnR*
Rp10	panlindromic	72	AACCGGGTTATTCTATTCCACTTCTAGATAGAGAAAAAAACTAAAGGAGAATACGTATGATCATTACCCTTC	*rbcL*/*accD*, *rpl23**
Rt37	tandem	26	AGTTTTGTTTAATGATTAATTAAGAG	*trnK*
Rt38	tandem	27	AATTTTTCCCATTATTTGACTATCCAT	*rps12_3end*/*trnV*
Rt39	tandem	27	ATTATGGATAGTCAAATAATGGGAAAA	*trnV*/*rps12_3end*

Rp22	panlindromic	25	GCAGTATTTACAGAAAAAAGTCTTC	*rbcL/accD, rpl23**
Rt40	tandem	17	TATCTCTATCATAAATC	*rps16/trnQ*
Rt41	tandem	19	AATACTATAGATTCTATTA	*clpP/psbB*
Rt42	tandem	20	ATAGTAGAGGATATCTGTTT	*trnV/trnM*
Rp13	panlindromic	55	AACCGGGTTATTCTATTCCACTTCTAGATAGAGAAAAAAACTAAAGGAGAATTTC	*rbcL/accD, rpl23**
Rp19	panlindromic	26	TCTAAAGTGTGGTAGAAAGAACTACA	*psbM/petN*
Rd15	dispersed	30	CCTAGAGGACGAATATAGGACTCGAGAGGA	*rpoC2**
Rp18	panlindromic	24	TAAAGTGTGGTAGAAAGAACTACA	*psbM/petN*
Rt36	tandem	17	TCTAATCGCTCTTTCTA	*psbM/petN*
Rp29	panlindromic	22	TAAAGTGTGGTAGAAAGAACTA	*psbM/petN*
Rp25	panlindromic	26	TTCAACCGGGTTATTCTATTCCACTT	*rbcL/accD, rpl23**
Rt12	tandem	21	TTTCATAAATCCAAGCAAACT	*rps18**
Rd5	dispersed	46	CTTAGGTTTCCGAAATAGTGTAATGGAAAAAGAAGTGCTTCGAATC	*psbE/petL, rps7/rps12_3end*
Rp5	panlindromic	22	TAAAGTGTGGTAGAAAAAAACTA	*psbM/petN*
Rt1	tandem	18	TATATGAATGAAACATAT	*atpB/rbcL*
Rd12	dispersed	22	TTTTCACCTCATACGGCTCCTC	*ycf3, trnV/rps12_3end*
Rp20	panlindromic	35	ATGGAATCAAATACGCAGTATTTACAGAAAAAAGT	*rbcL/accD, rpl23**
Rt3	tandem	21	TAAATCCAAGCAAACTTTTCG	*rps18**
Rp1	panlindromic	20	TTCTTATTCTTTTTTCTTTC	*rps16, ndhF**
Rp24	panlindromic	20	ACTTCTAGATAGAGAAAAAA	*rbcL/accD, rpl23**
Rd9	dispersed	30	TTCTTTTTCGAAATCGATTTCGAAAAAGAA	*ndhB*
Rp4	panlindromic	21	GTCATCGGTTCAAATCCGATA	*trnT**
Rd7	dispersed	35	TATTCCATGAATATGATATCTATCTAATGTGATAT	*trnL/ndhB*
Rd8	dispersed	35	ATATCACATTAGATAGATATCATATTCATGGAATA	*ndhB/trnL*
Rd18	dispersed	25	ATTCTTTTATTTTAGATAGAAGAAA	*trnN/rps15*
Rp2	panlindromic	22	TAATTGAAGTAAGAAGTCTCCC	*psbT/psbN*
Rp3	panlindromic	21	AGAGAGGGATTCGAACCCTCG	*trnS**
Rp17	panlindromic	21	GACATACAATGCATTACAAAC	*rbcL/accD, rpl23**
Rt10	tandem	19	TATGAATATACAATCTAGA	*atpI/atpH*
Rt13	tandem	26	ATATCTGTATATGGAGAGTATACTTG	*ndhF/rpl32*
Rp6	panlindromic	23	TTTCTATTCTTTCTATTTCTATT	*psbM/petN*
Rp23	panlindromic	38	ACGTATGATCATTACCCTTCAACCGGGTTATTCTATTC	*rbcL/accD, rpl23**

Rd3	dispersed	32	ATGGAATCAAATACGCAGTATTTACAGAAAAA	*rbcL*/*accD, rpl23**
Rp21	panlindromic	23	ATGGAATCAAATACGCAGTATTT	*rbcL*/*accD, rpl23**

Supplementary Table 13. Summary of SSRs identified in the 24 *Oryzeae* chloroplast genomes.

Species	Dinucleotide		Trinucleotide		Tetranucleotide		Pentanucleotide		Total	
	≥8 bp	≥10 bp	≥9 bp	≥12 bp	≥12 bp	≥16 bp	≥15 bp	≥20 bp	A	B
O. sativa ssp. *japonica*	20	4	41	3	9	2	0	0	70	9
O. rufipogon	20	4	41	3	11	2	0	0	72	9
O. sativa ssp. *indica*	20	4	41	3	10	2	1	0	72	9
O. nivara	20	4	41	3	10	2	1	0	72	9
O. glaberrima	20	4	41	3	10	2	0	0	71	9
O. barthii	21	4	41	3	10	2	0	0	72	9
O. glumaepatula	20	4	41	3	10	2	0	0	71	9
O. meridionalis	20	4	41	3	10	2	0	0	71	9
O. longistaminata	20	4	41	3	10	2	0	0	71	9
O. punctata	23	4	39	3	12	2	0	0	74	9
O. minuta	23	4	39	3	12	2	0	0	74	9
O. officinalis	22	5	41	3	11	3	0	0	74	11
O. rhizomatis	22	5	41	3	11	3	0	0	74	11
O. eichingeri	22	5	41	3	11	3	0	0	74	11
O. alta	22	5	41	3	11	3	0	0	74	11
O. grandiglumis	22	5	41	3	11	3	0	0	74	11
O. latifolia	22	5	41	3	10	3	0	0	73	11
O. australiensis	21	4	40	4	10	2	0	0	71	10
O. brachyantha	22	3	40	3	8	2	0	0	70	8
O. longiglumis	23	5	43	6	12	2	0	0	78	13
O. ridleyi	24	5	43	5	12	2	0	0	79	12
O. granulata	23	6	45	3	12	1	1	0	81	10
O. meyeriana	23	6	45	3	11	1	0	0	79	10
L. japonica	22	3	45	1	9	1	0	0	76	5
Mean	21.54	4.42	41.42	3.17	10.54	2.12	0.13	0	73.63	9.71

Note: A: The total number of dinucleotide ≥8 bp, trinucleotide ≥9 bp, tetranucleotide ≥12 bp, and pentanucleotide ≥15 bp. B: The total number of dinucleotide ≥10 bp, trinucleotide ≥12 bp, tetranucleotide ≥16 bp, and pentanucleotide ≥20 bp.

Supplementary Table 14. Comparisons of s across the *Oryza* plastomes using *L. japonica* as outgroup.

SSR No.	nex	Start	End	Number of species	SSR motifs	Repeat Count	Mutation Type	Species
11	nex	3267	3279	2	$(AG)_6$		Count	*O. ridleyi, O. longiglumis*
	nex	3267	3275	2	$(AG)_3$		Count	*L. japonica, O. brachyanth*
	nex	3267	3279	20	$(AG)_5$		Count	*O. rhizomatis, O. officinalis, O. sativa ssp. indica, O. alta, O. latifolia, O. minuta, O. meyeriana, O. australiensis, O. longistaminata, O. glumaepatula, O. meridionalis, O. rufipogon, O. barthii, O. grandiglumis, O. glaberrima, O. sativa ssp. japonica, O. granulata, O. punctata, O. eichingeri, O. nivara*
12	nex	4251	4304	1	$(TTTA)_3 + (AAAT)_2$		Count	*L. japonica*
	nex	4252	4306	2	$(TTTA)_3 + (AAAT)_1$		Count	*O. granulata, O. meyeriana*
	nex	4252	4306	2	(TTTA)3 + (AAAT)3		Count	*O. longiglumis, O. ridleyi*
	nex	4252	4294	1	(TTTA)4 + (AAAT)2		Count	*O. australiensis*
	nex	4252	4310	1	(TTTA)3 + (AAAT)0		Count	*O. brachyantha*
	nex	4252	4304	8	(TTTA)4 + (AAAT)3		Count	*O. officinalis, O. eichingeri, O. minuta, O. rhizomatis, O. punctata, O. latifolia, O. alta, O. grandiglumis*
	nex	4252	4294	9	(TTTA)2 + (AAAT)4		Count	*O. sativa ssp. indica, O. nivara, O. longistaminata, O. glaberrima, O. rufipogon, O. sativa ssp. japonica, O. meridionalis, O. barthii, O. glumaepatula*
14	nex	5111	5140	1	$(TAT)_4$		indel	*L. japonica*
	nex	5134	5146	1	$(ATAG)_3$		indel	*O. australiensis*
34	nex	12110	12142	7	(GA)5		indel	*O. rhizomatis, O. latifolia, O. eichingeri, O. officinalis, O. alta, O. grandiglumis, O. nivara*
49	nex	15380	15397	3	(CTTT)3		indel	*L. japonica, O. meyeriana, O. ridleyi*
				2	(CTTT)4		indel	*O. granulata, O. longiglumis*

				7	(CTTT)2C(CTTT)2	indel	*O. officinalis, O. rhizomatis, O. eichingeri, O. alta, O. grandiglumis, O. latifolia, O. australiensis,*
58	nex	17726	17734	14	$(TC)_4$	Count	*O. rhizomatis, O. latifolia, O. australiensis, O. alta, O. punctata, O. ridleyi, O. minuta, O. eichingeri, O. nivara, O. glumaepatula, O. granulata, O. meyeriana, O. officinalis, O. grandiglumis*
	nex	17728	17734	1	$(TC)_3$	Count	*O. longiglumis*
				1	TTTCTCTC	SNP	*O. brachyantha*
				7	TCTCTTTC	SNP	*O. sativa ssp. indica, O. longistaminata, O. glaberrima, O. rufipogon, O. sativa ssp. japonica, O. meridionalis, O. barthii*
66	nex	19788	19812	1	(TTTCTA)3	indel	*O. glaberrima*
67	nex	19831	19858	2	(AATAGA)3	Count	*O. barthii, O. glaberrima*
87	nex	27362	27391	1	(AAT)3	indel	*L. japonica*
	nex	27379	27391	23	(AAT)4	indel	*O. glaberrima, O. brachyantha, O. minuta, O. eichingeri, O. granulata, O. ridleyi, O. longiglumis, O. sativa ssp. indica, O. latifolia, O. meyeriana, O. rufipogon, O. australiensis, O. glumaepatula, O. grandiglumis, O. punctata, O. alta, O. sativa ssp. japonica, O. barthii, O. meridionalis, O. longistaminata, O. officinalis, O. rhizomatis, O. nivara*
96	nex	29889	29898	1	(AGA)3	SNP	*L. japonica*
103	nex	32749	32758	1	(ATT)3	indel	*L. japonica*
107	nex	33551	33559	1	(CT)4	SNP	*O. brachyantha*
	nex	33552	33558	22	TT(CT)3	SNP	*O. sativa ssp. indica, O. ridleyi, O. eichingeri, O. sativa ssp. japonica, O. alta, O. latifolia, O. granulata, O. nivara, O. glaberrima, O. meridionalis, O. glumaepatula, O. australiensis, O. officinalis, O. grandiglumis, O. barthii, O. minuta, O. punctata, O. longiglumis, O. rufipogon, O. meyeriana, O. longistaminata, O. rhizomatis*
	nex	33558	33564	1	TTCC(CT)2	SNP	*L. japonica*
115	nex	34759	34769	2	(GA)5	Count	*O. granulata, O. meyeriana*

				3	(GA)4	Count	*O. brachyantha, O. longiglumis, O. ridleyi*
	nex	34760	34768	19	AA(GA)3	SNP	*O. grandiglumis, O. latifolia, O. punctata, O. nivara, O. sativa ssp. japonica, O. officinalis, O. longistaminata, O. glumaepatula, O. glaberrima, O. minuta, O. alta, L. japonica, O. sativa ssp. indica, O. eichingeri, O. australiensis, O. rufipogon, O. meridionalis, O. barthii, O. rhizomatis*
128	nex	40141	40151	16	(CT)5	SNP	*O. meridionalis, O. rufipogon, O. longistaminata, O. nivara, O. minuta, O. latifolia, O. grandiglumis, O. barthii, O. punctata, O. eichingeri, O. glaberrima, O. alta, O. sativa ssp. japonica, O. officinalis, O. sativa ssp. indica, O. rhizomatis*
	nex	40142	40150	8	TT(CT)4	SNP	*O. glumaepatula, O. meyeriana, O. granulata, O. ridleyi, O. australiensis, O. brachyantha, O. longiglumis, L. japonica*
153	nex	48262	48284	2	(TTTA)3	indel	*O. punctata, O. minuta*
156	nex	49285	49295	1	$(TA)_5$TTA	indel	*O. meyeriana*
	nex	49285	49298	1	$(TA)_6$	SNP+indel	*O. granulata*
	nex	49287	49295	22	TG$(TA)_4$TTA	SNP+indel	*O. meridionalis, O. ridleyi, L. japonica, O. longiglumis, O. officinalis, O. brachyantha, O. alta, O. rufipogon, O. australiensis, O. longistaminata, O. barthii, O. nivara, O. rhizomatis, O. sativa ssp. japonica, O. minuta, O. sativa ssp. indica, O. latifolia, O. grandiglumis, O. punctata, O. glumaepatula, O. glaberrima, O. eichingeri*
158	nex	49812	49827	7	(AT)5	SNP	*O. granulata, O. ridleyi, O. meyeriana, O. australiensis, O. longiglumis, O. brachyantha, O. glumaepatula*
	nex	49812	49825	14	(AT)4AC	SNP	*O. longistaminata, O. meridionalis, O. sativa ssp. indica, O. eichingeri, O. alta, O. punctata, O. rhizomatis, O. grandiglumis, O. latifolia, O. barthii, O. nivara, O. minuta, O. officinalis, O. glaberrima*
	nex	49812	49820	2	(AT)4(TA)3	indel	*O. rufipogon, O. sativa ssp. japonica*

197				21	(GAA)3	SNP	*O. glaberrima, O. brachyantha, O. minuta, O. eichingeri, O. granulata, O. ridleyi, O. longiglumis, O. sativa ssp. indica, O. latifolia, O. meyeriana, O. rufipogon, O. grandiglumis, O. punctata, O. alta, O. sativa ssp. japonica, O. barthii, O. meridionalis, O. longistaminata, O. officinalis, O. rhizomatis, O. nivara*
				2	(GAA)2GAG	SNP	*O. australiensis, O. glumaepatula*
				1	GCA(GAA)2	SNP	*L. japonica*
198	nex	63189	63204	18	(GAA)3	Count	*O. punctata, O. nivara, O. australiensis, O. rufipogon, O. glaberrima, O. meyeriana, O. granulata, L. japonica, O. meridionalis, O. minuta, O. ridleyi, O. glumaepatula, O. longiglumis, O. brachyantha, O. sativa ssp. indica, O. barthii, O. longistaminata, O. sativa ssp. japonica*
	nex	63190	63205	3	(GAA)4	Count	*O. alta, O. grandiglumis, O. latifolia*
				3	(GAA)5	Count	*O. officinalis, O. rhizomatis, O. eichingeri*
234	nex	73767	73783	22	(AGAA)3	Count	*L. japonica, O. alta, O. australiensis, O. barthii, O. brachyantha, O. eichingeri, O. glaberrima, O. glumaepatula, O. grandiglumis, O. granulata, O. longiglumis, O. longistaminata, O. meyeriana, O. minuta, O. nivara, O. officinalis, O. punctata, O. rhizomatis, O. ridleyi, O. rufipogon, O. sativa ssp. indica, O. sativa ssp. japonica*
				1	(AGAA)4	Count	*O. meridionalis*
244	nex	77191	77211	7	(TTTA)4	Count	*O. latifolia, O. grandiglumis, O. alta, O. rhizomatis, O. officinalis, O. eichingeri, O. nivara*
	nex	77195	77207	15	(TTTA)3	Count	*L. japonica, O. longiglumis, O. meyeriana, O. granulata, O. minuta, O. brachyantha, O. rufipogon, O. longistaminata, O. meridionalis, O. barthii, O. punctata, O. ridleyi, O. sativa ssp. indica, O. glaberrima, O. sativa ssp. japonica*
	nex	77195	77211	2	TTTT(TTTA)2	Count+SNP	*O. australiensis, O. glumaepatula*

331	nex	122529	122535	23	CT(AT)3	SNP	*O. meyeriana, O. officinalis, O. granulata, O. latifolia, O. glaberrima, O. longistaminata, O. alta, O. brachyantha, O. punctata, O. barthii, O. glumaepatula, O. eichingeri, O. minuta, O. ridleyi, O. meridionalis, O. rhizomatis, O. nivara, O. grandiglumis, O. sativa ssp. indica, O. longiglumis, O. sativa ssp. japonica, O. australiensis, O. rufipogon*
	nex	122529	122537	1	(AT)4	SNP	*L. japonica*
345				2	(ATT)4	Count	*O. longiglumis, O. ridleyi*
				21	(ATT)2	Count	*others*
347	nex	110664	110672	6	(AT)4	indel	*O. longiglumis, O. ridleyi, O. brachyantha, O. granulata, O. meyeriana, L. japonica*
	nex	110666	110672	17	(AT)3	indel	*O. glumaepatula, O. grandiglumis, O. sativa ssp. japonica, O. barthii, O. officinalis, O. glaberrima, O. punctata, O. minuta, O. alta, O. rhizomatis, O. rufipogon, O. sativa ssp. indica, O. australiensis, O. nivara, O. latifolia, O. longistaminata, O. eichingeri*
	nex	110665	110672	1	(AT)2AG	indel+SNP	*O. meridionalis*
349	nex	111021	111033	21	(CAAA)3	indel	*O. glaberrima, O. brachyantha, O. minuta, O. eichingeri, O. granulata, O. ridleyi, O. longiglumis, O. sativa ssp. indica, O. latifolia, O. meyeriana, O. rufipogon, O. australiensis, O. glumaepatula, O. grandiglumis, O. punctata, O. alta, O. barthii, O. meridionalis, O. longistaminata, O. officinalis, O. rhizomatis*
	nex	111021	111037	1	(CAAA)2CAA	indel	*O. nivara*
	nex	111021	111033	2	(CAAA)2	indel	*O. sativa ssp. japonica, L. japonica*
360	nex	115335	115353	1	(TAA)3	indel	*L. japonica*
	nex	115336	115345	1	(TAA)4	indel	*O. longiglumis*
	nex	115336	115352	3	(TAA)3GAA	SNP	*O. granulata, O. meyeriana, O. brachyantha*
				1	(TAA)3AAA	SNP	*O. ridleyi*
	nex	115336	115345	2	(TAA)2GAA	SNP	*O. australiensis, O. glumaepatula*

				16	(TAA)2		indel	*O. sativa ssp. japonica, O. sativa ssp. indica, O. nivara, O. rufipogon, O. glaberrima, O. barthii, O. meridionalis, O. longistaminata, O. punctata, O. minuta, O. officinalis, O. rhizomatis, O. eichingeri, O. alta, O. grandiglumis, O. latifolia*
361	nex	115422	115447	7	(TAT)3		Count	*O. rhizomatis, O. eichingeri, O. latifolia, O. alta, O. officinalis, O. grandiglumis, O. nivara*
	nex	115422	115439	16	(TAT)4		Count	*O. longiglumis, O. ridleyi, O. australiensis, O. minuta, O. longistaminata, O. brachyantha, O. rufipogon, O. sativa ssp. japonica, O. glaberrima, O. glumaepatula, O. granulata, O. punctata, O. meridionalis, O. meyeriana, O. barthii, O. sativa ssp. indica*
	nex	115429	115438	1	(TTA)3		indel	*L. japonica*
1	nex	42	54	1	(TTCC)$_3$		SNP	*O. granulata*
13	nex	5065	5077	1	(ATAG)$_3$			*O. glumaepatula*
29	nex	11067	11076	1	(GGA)3			*L. japonica*
32	nex	11997	12009	1	(CATT)3			*O. rufipogon*
88	nex	27593	27602	1	TAG	3		*L. japonica*
152	nex	48229	48238	1	AAT	3		*L. japonica*
155	nex	48446	48464	1	AAGGGG	3		*O. rhizomatis*
157	nex	49524	49532	1	AT	4		*L. japonica*
174	nex	54812	54821	1	ATT	3		*O. brachyantha*
195	nex	62503	62518	1	AAAGT	3		*O. sativa ssp. indica*
228	nex	72143	72152	1	TAA	3		*L. japonica*
230	nex	72223	72238	1	TTCTA	3		*O. granulata*
267	nex	82124	82135	1	AT	5		*L. japonica*
348	nex	110755	110773	1	TAAATA	3		*O. ridleyi*
363	nex	116570	116588	1	ACTAAA	3		*O. ridleyi*
371	nex	119637	119646	1	ATA	3		*L. japonica*
18	nex	6727	6736	2	TAA	3		*O. granulata, O. meyeriana*
120	nex	35829	35841	2	TTA	4		*O. longiglumis, O. ridleyi*
126	nex	39611	39619	2	AT	4		*O. barthii, O. glaberrima*

185	nex	59876	59885	2	GCA	3	*O. granulata, O. meyeriana*
205	nex	65829	65852	2	TTC	4	*O. australiensis, O. glumaepatula*
235	nex	73927	73937	2	TA	5	*O. longiglumis, O. ridleyi*
236	nex	74002	74010	2	AG	4	*O. minuta, O. punctata*
346	nex	110634	110646	2	ATT	4	*O. longiglumis, O. ridleyi*
97	nex	30231	30240	5	AGA	3	*O. brachyantha, O. granulata, O. longiglumis, O. meyeriana, O. ridleyi*
35	nex	12154	12162	6	GA	4	*O. alta, O. eichingeri, O. grandiglumis, O. latifolia, O. officinalis, O. rhizomatis*
369	nex	119122	119131	8	AGC	3	*L. japonica, O. australiensis, O. brachyantha, O. glumaepatula, O. granulata, O. longiglumis, O. meyeriana, O. ridleyi*
226	nex	71558	71567	15	GAA	3	*L. japonica, O. alta, O. australiensis, O. brachyantha, O. eichingeri, O. glumaepatula, O. grandiglumis, O. granulata, O. latifolia, O. longiglumis, O. meyeriana, O. nivara, O. officinalis, O. rhizomatis, O. ridleyi*
179	nex	56528	56537	17	TCC	3	*L. japonica, O. australiensis, O. barthii, O. brachyantha, O. glaberrima, O. glumaepatula, O. granulata, O. longiglumis, O. longistaminata, O. meridionalis, O. meyeriana, O. minuta, O. punctata, O. ridleyi, O. rufipogon, O. sativa ssp. indica, O. sativa ssp. japonica*
9	nex	2677	2686	18	AGT	3	*O. alta, O. australiensis, O. barthii, O. eichingeri, O. glaberrima, O. glumaepatula, O. grandiglumis, O. latifolia, O. longistaminata, O. meridionalis, O. minuta, O. nivara, O. officinalis, O. punctata, O. rhizomatis, O. rufipogon, O. sativa ssp. indica, O. sativa ssp. japonica*
17	nex	6707	6716	18	AAG	3	*O. alta, O. barthii, O. eichingeri, O. glaberrima, O. grandiglumis, O. granulata, O. latifolia, O. longiglumis, O. longistaminata, O. meridionalis, O. meyeriana, O. nivara, O. officinalis, O. rhizomatis, O. ridleyi, O. rufipogon, O. sativa ssp. indica, O. sativa ssp. japonica*

95	nex	29493	29502	18	TGA	3	*O. alta, O. australiensis, O. barthii, O. eichingeri, O. glaberrima, O. glumaepatula, O. grandiglumis, O. latifolia, O. longistaminata, O. meridionalis, O. minuta, O. nivara, O. officinalis, O. punctata, O. rhizomatis, O. rufipogon, O. sativa ssp. indica, O. sativa ssp. japonica*
197	nex	63177	63186	21	GAA	3	*O. alta, O. barthii, O. brachyantha, O. eichingeri, O. glaberrima, O. grandiglumis, O. granulata, O. latifolia, O. longiglumis, O. longistaminata, O. meridionalis, O. meyeriana, O. minuta, O. nivara, O. officinalis, O. punctata, O. rhizomatis, O. ridleyi, O. rufipogon, O. sativa ssp. indica, O. sativa ssp. japonica*
43	nex	13810	13819	22	ATT	3	*L. japonica, O. alta, O. barthii, O. brachyantha, O. eichingeri, O. glaberrima, O. grandiglumis, O. granulata, O. latifolia, O. longiglumis, O. longistaminata, O. meridionalis, O. meyeriana, O. minuta, O. nivara, O. officinalis, O. punctata, O. rhizomatis, O. ridleyi, O. rufipogon, O. sativa ssp. indica, O. sativa ssp. japonica*
73	nex	21930	21939	22	CTA	3	*L. japonica, O. alta, O. australiensis, O. barthii, O. brachyantha, O. eichingeri, O. glaberrima, O. glumaepatula, O. grandiglumis, O. granulata, O. latifolia, O. longiglumis, O. longistaminata, O. meridionalis, O. meyeriana, O. nivara, O. officinalis, O. rhizomatis, O. ridleyi, O. rufipogon, O. sativa ssp. indica, O. sativa ssp. japonica*
132	nex	41696	41705	22	ATG	3	*L. japonica, O. alta, O. barthii, O. brachyantha, O. eichingeri, O. glaberrima, O. grandiglumis, O. granulata, O. latifolia, O. longiglumis, O. longistaminata, O. meridionalis, O. meyeriana, O. minuta, O. nivara, O. officinalis, O. punctata, O. rhizomatis, O. ridleyi, O. rufipogon, O. sativa ssp. indica, O. sativa ssp. japonica*

133	nex	43301	43310	22	TTG	3	*L. japonica, O. alta, O. australiensis, O. barthii, O. brachyantha, O. eichingeri, O. glaberrima, O. glumaepatula, O. grandiglumis, O. granulata, O. latifolia, O. longistaminata, O. meridionalis, O. meyeriana, O. minuta, O. nivara, O. officinalis, O. punctata, O. rhizomatis, O. rufipogon, O. sativa ssp. indica, O. sativa ssp. japonica*
7	nex	2343	2352	23	TTC	3	*O. alta, O. australiensis, O. barthii, O. brachyantha, O. eichingeri, O. glaberrima, O. glumaepatula, O. grandiglumis, O. granulata, O. latifolia, O. longiglumis, O. longistaminata, O. meridionalis, O. meyeriana, O. minuta, O. nivara, O. officinalis, O. punctata, O. rhizomatis, O. ridleyi, O. rufipogon, O. sativa ssp. indica, O. sativa ssp. japonica*
147	nex	47703	47712	23	CAA	3	*L. japonica, O. alta, O. australiensis, O. barthii, O. eichingeri, O. glaberrima, O. glumaepatula, O. grandiglumis, O. granulata, O. latifolia, O. longiglumis, O. longistaminata, O. meridionalis, O. meyeriana, O. minuta, O. nivara, O. officinalis, O. punctata, O. rhizomatis, O. ridleyi, O. rufipogon, O. sativa ssp. indica, O. sativa ssp. japonica*
168	nex	53210	53219	23	AAC	3	*L. japonica, O. alta, O. australiensis, O. barthii, O. eichingeri, O. glaberrima, O. glumaepatula, O. grandiglumis, O. granulata, O. latifolia, O. longiglumis, O. longistaminata, O. meridionalis, O. meyeriana, O. minuta, O. nivara, O. officinalis, O. punctata, O. rhizomatis, O. ridleyi, O. rufipogon, O. sativa ssp. indica, O. sativa ssp. japonica*
187	nex	60384	60396	23	AATA	3	*L. japonica, O. alta, O. australiensis, O. barthii, O. eichingeri, O. glaberrima, O. glumaepatula, O. grandiglumis, O. granulata, O. latifolia, O. longiglumis, O. longistaminata, O. meridionalis, O. meyeriana, O. minuta, O. nivara, O. officinalis, O. punctata, O. rhizomatis, O. ridleyi, O. rufipogon, O.*

							sativa ssp. indica, O. sativa ssp. japonica
217	nex	70324	70333	23	TTC	3	*O. alta, O. australiensis, O. barthii, O. brachyantha, O. eichingeri, O. glaberrima, O. glumaepatula, O. grandiglumis, O. granulata, O. latifolia, O. longiglumis, O. longistaminata, O. meridionalis, O. meyeriana, O. minuta, O. nivara, O. officinalis, O. punctata, O. rhizomatis, O. ridleyi, O. rufipogon, O. sativa ssp. indica, O. sativa ssp. japonica*
286	nex	86245	86257	23	TCT	4	*O. alta, O. australiensis, O. barthii, O. brachyantha, O. eichingeri, O. glaberrima, O. glumaepatula, O. grandiglumis, O. granulata, O. latifolia, O. longiglumis, O. longistaminata, O. meridionalis, O. meyeriana, O. minuta, O. nivara, O. officinalis, O. punctata, O. rhizomatis, O. ridleyi, O. rufipogon, O. sativa ssp. indica, O. sativa ssp. japonica*

Supplementary Table 15. The *dS*, *dN* and *ω* (*dN/dS*) of chloroplast protein-coding genes in *Oryza* species using *L. japonica* as outgroup.

Grp Description	*dN*	*dS*	*ω*
O. meyeriana	0.0077	0.0421	0.1826
O. granulata	0.0077	0.0420	0.1843
O. ridleyi	0.0082	0.0470	0.1736
O. longiglumis	0.0086	0.0485	0.1784
O. brachyantha	0.0071	0.0504	0.1417
O. australiensis	0.0085	0.0497	0.1713
O. latifolia	0.0077	0.0471	0.1636
O. grandiglumis	0.0077	0.0468	0.1644
O. alta	0.0077	0.0469	0.1634
O. eichingeri	0.0078	0.0472	0.1657
O. rhizomatis	0.0078	0.0480	0.1626
O. officinalis	0.0078	0.0471	0.1665
O. minuta	0.0079	0.0501	0.1581
O. punctata	0.0079	0.0499	0.1575
O. longistaminata	0.0079	0.0511	0.1555
O. meridionalis	0.0080	0.0512	0.1558
O. glumaepatula	0.0077	0.0503	0.1533
O. barthii	0.0078	0.0509	0.1540
O. glaberrima	0.0078	0.0508	0.1538
O. rufipogon	0.0078	0.0506	0.1532
O. sativa* ssp. *japonica	0.0090	0.0521	0.1722
O. nivara	0.0078	0.0506	0.1537
O. sativa* ssp. *indica	0.0078	0.0505	0.1541

Supplementary Table 16. Summary of the positively selected genes detected in the *Oryza* plastomes.

	PSGs	
	***P*-value < 0.05**	**FDR < 0.05**
**Site models**	11 (*accD*, *atpE*, *matK*, *ndhF*, *ndhH*, *psbB*, *psbH*, *rbcL*, *rpoA*, *rpoC2*, *ycf68*)	8 (*accD*, *matK*, *ndhF*, *ndhH*, *psbH*, *rbcL*, *rpoC2*, *ycf68*)
**Branch-site models**	22 (*accD*, *atpE*, *matK*, *ndhD*, *ndhF*, *ndhH*, *psaA*, *psaJ*, *psbB*, *psbD*, *psbH*, *rbcL*, *rpl16*, *rpl23*, *rpl32*, *rpoA*, *rpoB*, *rpoC2*, *rps14*, *rps2*, *ycf3*, *ycf68*)	14 (*accD*, *matK*, *ndhD*, *ndhF*, *ndhH*, *psaA*, *psbB*, *psbD*, *psbH*, *rbcL*, *rpl16*, *rpoA*, *rpoC2*, *ycf68*)*
**Total** (no-redundancy)	22 (*accD*, *atpE*, *matK*, *ndhD*, *ndhF*, *ndhH*, *psaA*, *psaJ*, *psbB*, *psbD*, *psbH*, *rbcL*, *rpl16*, *rpl23*, *rpl32*, *rpoA*, *rpoB*, *rpoC2*, *rps14*, *rps2*, *ycf3*, *ycf68*)	14 (*accD*, *matK*, *ndhD*, *ndhF*, *ndhH*, *psaA*, *psbB*, *psbD*, *psbH*, *rbcL*, *rpl16*, *rpoA*, *rpoC2*, *ycf68*)

Supplementary Table 17. Genomic locations of the positively selected genes in the *Oryza* chloroplast genomes tested by both site model and branch-site model for all branches.

Gene location	The positively selected genes	Total
**LSC**	*rpoC2*, *rpl16*, *rbcL*, *psbH*, *psbB*, *rpoA*, *matK*, *accD*, *psaA*, *psbD*	10
**SSC**	*ndhF*, *ndhD*	2
**IR**	*ndhH*, *ycf68*,	2
**Total**		14

Supplementary Table 18. Functional categories of the positively selected genes in the *Oryza* chloroplast genomes by both site model and branch-site model for all branches.

Category for genes	The positively selected genes	Total
**Self replication**	*rpoC2, rpl16, rpoA*	3
**Photosynthesis**	*rbcL, ndhH, psbH, ndhF, psbB, ndhD, psaA, psbD*	8
**Other genes**	*matK*	1
**Genes with unknown functions**	*ycf68, accD*	2
**Total**		14

Supplementary Table 19. Functional annotation of chloroplast genes of the *Oryza* and *L. japonica* plastomes.

Categories	Gene annotation	Name of genes
**Self replication**	Large subunit of ribosome	*rpl2**, *rpl14*, *rpl16**, *rpl20*, *rpl22*, *rpl23*, *rpl32*, *rpl33*, *rpl36*
	Small subunit of ribosome	*rps2*, *rps3*, *rps4*, *rps7**, *rps8*, *rps11*, *rps12**, *rps14*, *rps15*, *rps16**, *rps18*, *rps19*
	DNA dependent RNA polymerase	*rpoA*, *rpoB*, *rpoC1**, *rpoC2*
	rRNA genes	*rrn4.5*, *rrn5*, *rrn16*, *rrn23*
	tRNA genes	*trnA-UGC**, *trnC-GCA*, *trnD-GUC*, *trnE-UUC*, *trnF-GAA*, *trnG-UCC**, *trnH-GUG*, *trnI-CAU*, *trnI-GAU**, *trnK-UUU**, *trnL-UAG*, *trnL-CAA*, *trnL-UAA**, *trnM-CAU*, *trnfM-CAU*, *trnN-GUU*, *trnP-UGG*, *trnQ-UUG*, *trnR-ACG*, *trnR-UCU*, *trnS-GGA*, *trnS-GCU*, *trnS-UGA*, *trnT-GGU*, *trnT-UGU*, *trnV-UAC**, *trnV-GAC*, *trnW-CCA*, *trnY-GUA*
**Photosynthesis**	Subunits of photosystem I	*psaA*, *psaB*, *psaC*, *psaI*, *psaJ*, *ycf3***, *ycf4*
	Subunits of photosystem II	*psbA*, *psbB*, *psbC*, *psbD*, *psbE*, *psbF*, *psbH*, *psbI*, *psbJ*, *psbK*, *psbL*, *psbM*, *psbN*, *psbT*, *psbZ*
	Subunits of NADH-dehydrogenase	*ndhA**, *ndhB**, *ndhC*, *ndhD*, *ndhE*, *ndhF*, *ndhG*, *ndhH*, *ndhI*, *ndhJ*, *ndhK*
	Subunits of cytochrome b/f complex	*petA*, *petB**, *petD**, *petG*, *petL*, *petN*
	Subunits of ATP synthase	*atpA*, *atpB*, *atpE*, *atpF**, *atpH*, *atpI*
	Large subunit of rubisco	*rbcL*
**Other genes**	Translational initiation factor	*infA*
	Maturase	*matK*
	Protease	*clpP*
	Envelop membrane protein	*cemA*
	c-type cytochrom synthesis gene	*ccsA*
**Unknown function**	Conserved Open Reading Frames (ORF, *ycf*)	*accD*, *ycf68*

One (*) and two (**) asterisks indicate one- and two- introns containing genes, respectively. The *rps12* gene is divided into two parts: the *rps12*_5end is located in the LSC region while the *rps12*_3end is located in the IR region.

Supplementary Table 20. Branch or clade-based genes under positive selection in the *Oryza* phylogeny using *L. japonica* as outgroup.

**Branch/Clade**[a]	**Number of PSGs**[b]	**The positively selected genes**			
		**Self replication**	**Photosynthesis**	**Other genes**	**unknown function**
**#10**	1		*ndhF*		
**#14**	1		*psaA*		
**#16**	1				*accD*
**#27**	1		*ndhD*		
**#29**	1	*rpoC2*			
**#33**	1		*rbcL*		
**#36**	1		*psaA*		
**#44**	3		*ndhF, psbB, psaA*		
**$47**	3		*ndhF, psbB, psaA*		
**$50**	5	*rpl16, rpoC2*	*ndhF, psbB, psaA*		
**$51**	3	*rpl16*	*ndhF, psbB*		
**$52**	3	*rpl16*	*ndhF, psbB*		
**$53**	4	*rpl16, rpoC2*	*ndhF, psbB*		
**$54**	3		*ndhF, psbB, psaA*		
**$55**	1		*psaA*		
**$56**	4		*ndhF, psbB, psaA*	*matK*	
**$58**	1		*rbcL*		
**$60**	1		*psbD*		
**$61**	1		*psbH*		
**$62**	5	*rpoC2*	*psbH, rbcL, ndhF*		*ycf68*
**$63**	1		*ndhD*		
**$64**	1		*ndhD*		
**$66**	4		*ndhD, ndhH, rbcL,*		*accD*
**$67**	9	*rpoC2,rpoA*	*rbcL, ndhH, psbH, ndhF*	*matK*	*ycf68, accD*
**$68**	4	*rpoC2*	*ndhF, psbH, psbB*		
**Total**	14	3	8	1	2

[a] #/$ of each branch/clade is consistent with that labeled in the phylogenic tree shown in **Supplementary Figure 21** ;

[b] Number of non-redundant PSGs.

Supplementary Table 21. Summary of species-specific PSGs detected in the *Oryza* plastomes. The Identified sites under positive selection were obtained by using the Bayes empirical Bayes (BEB) (Yang et al. 2005) to calculate the posterior probabilities. * P < 0.05; ** P < 0.01.

Genes	Branch	Model	lnL	*P*-value	FDR	Positively selected sites
ndhF	Ancestral AA/BB/BBCC	Model A	-4019.93	8.87E-14	6.58E-11	
		Model A1	-4047.73			
ndhF	*O. sativa* ssp. *japonica*	Model A	-3960.4	1.05E-27	2.34E-24	267A0.799
		Model A1	-4019.9			614A0.823
						615Y0.996**
						616M0.989*
						617F0.822
						618Y0.982*
						619G0.810
						620S0.979*
						621A0.985*
						622Y0.978*
						623S0.983*
						626Q0.979*
						627N0.905
						628L0.781
ndhD	*O. longiglumis*	Model A	-2609.17	1.18E-14	1.32E-11	109R0.970*
		Model A1	-2638.95			110N0.986*
						113L0.972*
						114F0.996**
						175S0.988*
						176I0.896
						177F0.826
						178F0.979*
						179L0.856
						180I0.803
psbB	*O. sativa* ssp. *japonica*	Model A	-2373.47	1.66E-06	7.41E-04	476R0.987*
		Model A1	-2384.94			494T0.818
						495F0.827
						498V0.987*
						504R0.822
rpoC2	*O. autraliensis*	Model A	-8199.63	9.20E-07	5.12E-04	712N0.896
		Model A1	-8211.67			714Y0.524
						720T0.536
						721L0.801
						723E0.681
						724D0.906
						725S0.900
						925N0.522

Supplementary Table 22. The primer pairs for PCR amplification of chloroplast genomes. The locations of primer pairs and estimated product sizes are shown using the chloroplast genome sequence of *O. nivara* (NC_005973) as a reference.

NO.	Primer	Sequence of the Primers	Location (bp)	Production (bp)
P1	cp1F	5′-ACTGAATAGGGAACCGCCGAATACACCA-3′	508	10080
	cp1R	5′-CGCCAAATACACAAATGAAACCCAACCA-3′	10587	
P2	cp2F	5′-AGTACTTCATCTAATTTCCTCCGCAGTCTT-3′	10190	9434
	cp2R	5′-GCTAATTCCGATCTTCCTCCCCAATCTG-3	19623	
P3	cp3F	5′-GGAGGAAGATCGGAATTAGCAATTGAT-3′	19604	6813
	cp3R	5′-GATGATACTGTAATGGAATGGCGAACC-3′	26416	
P4	cp4F	5′-GTCATTGGGTTAAGAGAAGTTCCGATTG-3′	24252	7822
	cp4R	5′-TTGTCCTGCAGCAGTACCTTGACCAACT-3′	32074	
P5	cp5F	5′-GCAGTTCGGAGAATTCAGATTATCGTTTC-3′	31839	9141
	cp5R	5′-AAGTCTTTAGTGCTCATTTCGGTCAACTCT-3′	40979	
P6	cp6F	5′-CAAGAAAGAGACCCAAGTCCTAATAACCC-3′	40556	10859
	cp6R	5′-GTACGAAAGAATTGGTTGAAGCGAAGGTAG-3′	51414	
P7	cp7F	5′-GGGCTGGATATTTACCTTATCAACTAGCAA-3′	50713	11155
	cp7R	5′-GAACTATGACACAATCAAACCCGAATG-3′	61868	
P8	cp8F	5′-TGATCCTTTCTTTCTCCTCGCTTCAT-3′	61159	10015
	cp8R	5′-CACACACTCATATTCCAGAGATACCGAAAC-3′	71173	
P9	cp9F	5′-GGACCAAGACAAACTCGCGTAGGTA-3′	70848	9641
	cp9R	5′-TAGGATAAAGAAGAAGAAGAGTGGGCTAAG-3′	80488	
P10	cp10F	5′-ATAGTACTCCTACTGACTTCGGCTTTAGTG-3′	80174	12587
	cp10R	5′-GCATAAGCATTAGCTCTCCCTGAAAAGGAG-3′	92760	
P11	cp11F	5′-GATTGCAGGCTGCAACTCGCCTGCATGAAG-3′	92498	9502
	cp11R	5′-GTCTATCCTCATGAACCGGGAAATACTATG-3′	101999	
P12	cp12F	5′-GACACCCTTGTCGAAAAACTGCGTTAGT-3′	101532	10718
	cp12R	5′-ATTGGTCTTCTTATGGCAGGATATAGCTCA-3′	112249	
P13	cp13F	5′-CCCGCTTCAAGATATGATGACTAATC-3′	111869	10673
	cp11F	5′-GATTGCAGGCTGCAACTCGCCTGCATGAAG-3′	122541	
P14	cp10R	5′-GCATAAGCATTAGCTCTCCCTGAAAAGGAG-3′	122279	13235
	cp14R	5′-TGACCGCAACTTCTGTATTTATTATCGC-3′	1019	

Supplementary Table 23. The primer pairs for PCR amplification and sequencing for the chloroplast genome sequence validation and gap closure.

Species	No. of Gap	Primer	Primer sequence (5’-3’)
O. nivara	25	15g1F	GCCGAGTACTCTACCATTGA
		9g1R	CGTATTGTATTCTGCACGAGA
		15g9F	GATTACTTCCGGATTTAGGTG
		15g11R	TATGTAGTCTAGCGGTTCGTC
		8g8F	GGAGCTCCCGTGTCAATCACT
		8g12R	GAAGCCACCGGTATAACACCT
		8g13F	GATCGTGCTCGCGGTATCTT
		8g15R	CAATAATGCGCGGTAAAGG
		8g16F	CCTTTACCGCGCATTATTG
		8g20R	GATTGGATATCCCGCATAAG
		8g21F	GCGGGATATCCAATCAATTAG
		8g27R	GGGAACAGGGCCTATCACAA
		8g28F	GTGCTTCAGGATCCATTACGC
		cp7R	GAACTATGACACAATCAAACCCGAATG
		15g17F	CGCCATTCCTGAGATTAAC
		cp8R	CACACACTCATATTCCAGAGATACCGAAAC
		1g7F	GGCCTGTTATCTCTATCAAGA
		13g19R	CCCCTCAATATTGCAAGTTT
		8g34F	GACCTTGCGGATCTGTAACA
		8g35R	CTTGGTTTCAACTTTGCGACA
		11g6F	CCACCACGACGTGCATATC
		9g6R	TGAGAAATTGACTCGGAAAG
		13g23F	AGGTTTGAATCTCGCAATGG
		13g23R	AGAAGAAGAGTGGGCTAAGGA
		9g15F	GTCCAAACCCCATTGACATAA
		9g15R	CGCCAGAAAATGATCTTGAT
		13g25F	TCTTCTCTCCATCGGAACAA
		15g20R	TTATTCGAGGAGCCCTAGAT
O. rufipogon	9	15g5F	GCGATATGTTGGGAATAGCTC
		15g5R	GGGCTGCTTTAATGGTAGTCT
		9g2F	GTATGTGGAGTTCCCGAGAA
		9g2R	CGTCAAGTATACCCGTTTCAA
		15g9F	GATTACTTCCGGATTTAGGTG
		15g11R	TATGTAGTCTAGCGGTTCGTC
		9g3F	CCGGTAGATAAACTAGATAGC
		9g4R	CGCCAACTAGATACTCCT
		15g17F	CGCCATTCCTGAGATTAAC
		cp8R	CACACACTCATATTCCAGAGATACCGAAAC
		9g6F	GTTTCGGTCGGTCTTATTGTA
		9g6R	TGAGAAATTGACTCGGAAAG
		15g23F	AAGTCCAAACCCCATTGACAT
		15g23R	ATTCTTGCTTTGCCCAATAG
O. glaberrima	1	21g1F	TCCCAGCCCTGATACCAAT
		21g1R	GGCCATCTCTCCTCCATAATC
O. barthii	30	15g1F	GCCGAGTACTCTACCATTGA
		9g1R	CGTATTGTATTCTGCACGAGA
		15g3F	CCGCACAATACCTAACCTA
		15g4R	CTTGCGTGGTCTAAGTCTAAG
		15g5F	GCGATATGTTGGGAATAGCTC
		15g5R	GGGCTGCTTTAATGGTAGTCT
		9g2F	GTATGTGGAGTTCCCGAGAA
		9g2R	CGTCAAGTATACCCGTTTCAA
		15g7F	CATAGCCCAAAGTCGTATCTC
		15g7R	AATGACAACGAGCCCTTATT

		15g8F	GTTCGGAGAGTTCAGATTATC
		15g8R	TAATTCGTCGAGATCGTG
		15g9F	GATTACTTCCGGATTTAGGTG
		15g11R	TATGTAGTCTAGCGGTTCGTC
		15g12F	CGTATAGATACTGGCATAGCA
		15g12R	TATCAATACCTATTCGGAAGA
		9g3F	CCGGTAGATAAACTAGATAGC
		9g4R	CGCCAACTAGATACTCCT
		cp8F	TGATCCTTTCTTTCTCCTCGCTTCAT
		15g15R	AGTCCCTAATTCCGAATCAAG
		15g16F	GATAGATTGGGTTGGACCTTA
		9g5R	TGCCGTCACTGTATGGATAA
		15g17F	CGCCATTCCTGAGATTAAC
		cp8R	CACACACTCATATTCCAGAGATACCGAAAC
		9g6F	GTTTCGGTCGGTCTTATTGTA
		9g6R	TGAGAAATTGACTCGGAAAG
		15g19F	CGGGACCACGTTACTATTGT
		15g19R	AGAGGCGAATTGAAAGCTAAG
		15g20F	CTCCAAGCCGTACATACAACT
		15g20R	TTATTCGAGGAGCCCTAGAT
		cp11F	GATTGCAGGCTGCAACTCGCCTGCATGAAG
		cp10R	GCATAAGCATTAGCTCTCCCTGAAAAGGAG
		15g22F	TCTTACGCTCTGACCCGAGTA
		15g22R	GTTACCCAAGCCGACATTCT
		15g23F	AAGTCCAAACCCCATTGACAT
		15g23R	ATTCTTGCTTTGCCCAATAG
		9g16F	GCTCGTACACATTGAGTGCAT
		9g16R	CGGGTGGAATTTGTCTATT
		15g25F	GGGCTTCCACTCTTGCATAAA
		15g25R	GGATATGAGTCTACCGCTTAC
O. glumaepatula	0	-	-
O. meridionalis	1	19g2F	CGAGAGGATAAATATGGCAC
		19g2R	CTTCTTGATGTTGGCTAAGTG
O. longistaminata	24	9g1F	TGACAAAGTTGGCCTAA
		9g1R	CGTATTGTATTCTGCACGAGA
		9g2F	GTATGTGGAGTTCCCGAGAA
		9g2R	CGTCAAGTATACCCGTTTCAA
		cp4F	GTCATTGGGTTAAGAGAAGTTCCGATTG
		cp3R	GATGATACTGTAATGGAATGGCGAACC
		9g3F	CCGGTAGATAAACTAGATAGC
		9g4R	CGCCAACTAGATACTCCT
		9g5F	TTCTTCTTCCTCTTCGGTTTC
		9g5R	TGCCGTCACTGTATGGATAA
		9g6F	GTTTCGGTCGGTCTTATTGTA
		9g6R	TGAGAAATTGACTCGGAAAG
		cp10F	ATAGTACTCCTACTGACTTCGGCTTTAGTG
		9g8R	GTAACGGCAGCAAGTGATTGA
		9g9F	GGGCGTGGAAACAGATAGAA
		9g9R	GTTTACGGCTAGGACTACTGG
		9g10F	ATGCAAAGCGAAGAACCTTAC
		9g11R	GGTCTGTGAAGATGCGTTGTT
		9g12F	GGGTGAGTCAGGGCCTAAGAT
		9g13R	ATAGAGTCCGACCGCAACGAC
		9g14F	ATGAGTCTACCGCTTACAAGA
		cp11R	GTCTATCCTCATGAACCGGGAAATACTATG
		9g15F	GTCCAAACCCCATTGACATAA
		9g15R	CGCCAGAAAATGATCTTGAT

		9g16F	GCTCGTACACATTGAGTGCAT
		9g16R	CGGGTGGAATTTGTCTATT
O. punctata	2	9g1F	TGACAAAGTTGGCCTAA
		3g1R	CGTATTCTATTCTGCACGAGA
		8g16F	CCTTTACCGCGCATTATTG
		8g20R	GATTGGATATCCCGCATAAG
O. minuta	1	9g1F	TGACAAAGTTGGCCTAA
		9g1R	CGTATTGTATTCTGCACGAGA
O. officinalis	1	8g16F	CCTTTACCGCGCATTATTG
		8g20R	GATTGGATATCCCGCATAAG
O. rhizomatis	1	7g1F	GCGGATGTATTAGTCGTTGTT
		7g1R	ACGGAAGCAGCAGCAATTAGT
O. eichingeri	40	15g1F	GCCGAGTACTCTACCATTGA
		9g1R	CGTATTGTATTCTGCACGAGA
		15g9F	GATTACTTCCGGATTTAGGTG
		15g11R	TATGTAGTCTAGCGGTTCGTC
		8g5F	TCGTTGAGGAGGCGTATTCTC
		8g7R	AGTAGGGGAACGGACTCGTGA
		8g8F	GGAGCTCCCGTGTCAATCACT
		8g12R	GAAGCCACCGGTATAACACCT
		8g13F	GATCGTGCTCGCGGTATCTT
		8g15R	CAATAATGCGCGGTAAAGG
		8g16F	CCTTTACCGCGCATTATTG
		8g20R	GATTGGATATCCCGCATAAG
		8g21F	GCGGGATATCCAATCAATTAG
		8g27R	GGGAACAGGGCCTATCACAA
		8g28F	GTGCTTCAGGATCCATTACGC
		cp7R	GAACTATGACACAATCAAACCCGAATG
		1g7F	GGCCTGTTATCTCTATCAAGA
		1g7R	TGCCTGAATCTCAAGACGTT
		8g34F	GACCTTGCGGATCTGTAACA
		8g35R	CTTGGTTTCAACTTTGCGACA
		8g34F	GACCTTGCGGATCTGTAACA
		9g6R	TGAGAAATTGACTCGGAAAG
		15g23F	AAGTCCAAACCCCATTGACAT
		15g23R	ATTCTTGCTTTGCCCAATAG
		9g16F	GCTCGTACACATTGAGTGCAT
		9g16R	CGGGTGGAATTTGTCTATT
O. alta	0	-	-
O. grandiglumis	21	15g1F	GCCGAGTACTCTACCATTGA
		9g1R	CGTATTGTATTCTGCACGAGA
		9g1F	TGACAAAGTTGGCCTAA
		9g1R	CGTATTGTATTCTGCACGAGA
		15g3F	CCGCACAATACCTAACCTA
		10g6R	GTAACTCTACCAAGGGCTACA
		10g7F	TTTCTGTTCTGGCTCGGTT
		10g10R	CGTCTCCGCACTTAGCAGT
		10g11F	CGGACGCCTTAAGTATATCA
		10g13R	TCGCCTGTCCTACTCTAAGA
		10g14F	GCGAATATTCTTATGGTTCC
		15g5R	GGGCTGCTTTAATGGTAGTCT
		10g17F	GCAAGTATTCATCCACGTTTC
		9g2R	CGTCAAGTATACCCGTTTCAA
		8g16F	CCTTTACCGCGCATTATTG
		8g20R	GATTGGATATCCCGCATAAG
		15g23F	AAGTCCAAACCCCATTGACAT
		15g23R	ATTCTTGCTTTGCCCAATAG

O. latifolia	1	9g15F	GTCCAAACCCCATTGACATAA
		9g15R	CGCCAGAAAATGATCTTGAT
O. australiensis	2	15g5F	GCGATATGTTGGGAATAGCTC
		15g5R	GGGCTGCTTTAATGGTAGTCT
		9g15F	GTCCAAACCCCATTGACATAA
		9g15R	CGCCAGAAAATGATCTTGAT
O. brachyantha	9	20g1F	CGCGACTCTGTACTCATAATC
		20g1R	GGGCTTCTTATTCAATTCCA
		20g2F	GGCAGGAAGCAAGATCAAATA
		20g3R	AACGTGGATGAATACTTGCTC
		20g4F	GATTCGCGATTTCCTAAGA
		15g7R	AATGACAACGAGCCCTTATT
		15g9F	GATTACTTCCGGATTTAGGTG
		15g11R	TATGTAGTCTAGCGGTTCGTC
		8g21F	GCGGGATATCCAATCAATTAG
		8g27R	GGGAACAGGGCCTATCACAA
		8g28F	GTGCTTCAGGATCCATTACGC
		cp7R	GAACTATGACACAATCAAACCCGAATG
		cp10F	ATAGTACTCCTACTGACTTCGGCTTTAGTG
		9g8R	GTAACGGCAGCAAGTGATTGA
O. longiglumis	38	15g3F	CCGCACAATACCTAACCTA
		1g1R	GGGCTATAGTCATAGTGATCC
		15g5F	GCGATATGTTGGGAATAGCTC
		1g2R	TAATAATGCTGTTCGGACC
		1g3F	TATAGCTCTTCGGGAATGT
		1g3R	CAATAGAAGTGTTGGCCTACT
		21g1F	TCCCAGCCCTGATACCAAT
		21g1R	GGCCATCTCTCCTCCATAATC
		1g5F	CCAACTATTTGTCAGGCGACT
		8g7R	AGTAGGGGAACGGACTCGTGA
		1g6F	GCTAATGTCATCCGGTATTT
		1g6R	ATGCATTTATGCTCGTAGCCT
		1g7F	GGCCTGTTATCTCTATCAAGA
		1g7R	TGCCTGAATCTCAAGACGTT
		1g8F	TTGGATATTAGGGAGCGGTAA
		cp11R	GTCTATCCTCATGAACCGGGAAATACTATG
		cp12F	GACACCCTTGTCGAAAAACTGCGTTAGT
		1g11R	GGGGTTGGTTACTAGTTCC
		11g8F	AGCCCATATGCGACGAAGAT
		1g16R	TGGGTAATGTAAATGAACCAT
		1g17F	CCAAATTGGAAGGGTACGAAG
		1g21R	ACTGGAGATTGGGAATCGATG
		1g22F	GGATTCGCATCTCTTACAACC
		1g26R	TTTCTGCTTTTTCGCTAGGA
		1g27F	TGCCACAGCTACAAAGTAGGA
		cp12R	ATTGGTCTTCTTATGGCAGGATATAGCTCA
O. ridleyi	11	15g9F	GATTACTTCCGGATTTAGGTG
		15g11R	TATGTAGTCTAGCGGTTCGTC
		1g6F	GCTAATGTCATCCGGTATTT
		1g6R	ATGCATTTATGCTCGTAGCCT
		11g4F	CGGCTTCGGAATAGTAGAAT
		11g4R	GCCAATAAATAGAGCTAGGGT
		11g11F	CCTGTGACATTTTGTACCC
		11g11R	ACGAGCCTGTTTTATAGCA
		11g5F	CGCCATTCCTGAGATTAGCC
		11g5R	CCAGCCCAACCAGAAACTAG
		11g6F	CCACCACGACGTGCATATC

		9g6R	TGAGAAATTGACTCGGAAAG
		9g12F	GGGTGAGTCAGGGCCTAAGAT
		11g7R	ACAGCTCAAGGTGGCGAAGA
		11g8F	AGCCCATATGCGACGAAGAT
		9g15R	CGCCAGAAAATGATCTTGAT
		9g16F	GCTCGTACACATTGAGTGCAT
		9g16R	CGGGTGGAATTTGTCTATT
O. granulata	11	15g9F	GATTACTTCCGGATTTAGGTG
		15g11R	TATGTAGTCTAGCGGTTCGTC
		5g3F	TATCCACGCTGGTACAGTAG
		5g3R	CGTCGGTTCTAGACAATCTTA
		9g15F	GTCCAAACCCCATTGACATAA
		5g6R	CTCTTGCTTTGCCCAATAGA
		5g7F	GAGCTATATCCTGCCATAAGA
		5g11R	GCGGTAATTTCATCGTTC
O. meyeriana	0	-	-
L. japonica	5	9g1F	TGACAAAGTTGGCCTAA
		23g1R	ATATTGTACCCCACACGAGAC
		23g2F	CTTGAGAAATGGCCGGGTC
		23g2R	TATAGCGCTTACTCCGGGAAA
		1g5F	CCAACTATTTGTCAGGCGACT
		8g7R	AGTAGGGGAACGGACTCGTGA
		23g4F	TGGGTTGCGCTATATCTATC
		23g4R	CTGAGGAGTTACTCGGAATGC
		9g15F	GTCCAAACCCCATTGACATAA
		23g8R	CTTGCTTTGCCCAATAGAA
