# Supplementary material for: Evolution of Oryza chloroplast genomes promoted adaptation to diverse ecological habitats
Source: Commun Biol. 2019 Jul 26;2:278. doi: 10.1038/s42003-019-0531-2 (PMC6659635; doi:10.1038/s42003-019-0531-2)
Supplement: Supplementary file 2 — Reporting Summary [file 42003_2019_531_MOESM2_ESM.pdf]

nature research

Corresponding author(s): Li-zhi Gao

Reporting Summary

Nature Research wishes to improve the reproducibility of the work that we publish. This form provides structure for consistency and transparency in reporting. For further information on Nature Research policies, see Authors & Referees and the Editorial Policy Checklist.

Statistical parameters

When statistical analyses are reported, confirm that the following items are present in the relevant location (e.g. figure legend, table legend, main text, or Methods section).

n/a	Confirmed	
☒	☐	The exact sample size (n) for each experimental group/condition, given as a discrete number and unit of measurement
☒	☐	An indication of whether measurements were taken from distinct samples or whether the same sample was measured repeatedly
☐	☒	The statistical test(s) used AND whether they are one- or two-sided<br>*Only common tests should be described solely by name; describe more complex techniques in the Methods section.*
☐	☒	A description of all covariates tested
☒	☐	A description of any assumptions or corrections, such as tests of normality and adjustment for multiple comparisons
☒	☐	A full description of the statistics including central tendency (e.g. means) or other basic estimates (e.g. regression coefficient) AND variation (e.g. standard deviation) or associated estimates of uncertainty (e.g. confidence intervals)
☐	☒	For null hypothesis testing, the test statistic (e.g. F, t, r) with confidence intervals, effect sizes, degrees of freedom and P value noted<br>*Give P values as exact values whenever suitable.*
☒	☐	For Bayesian analysis, information on the choice of priors and Markov chain Monte Carlo settings
☒	☐	For hierarchical and complex designs, identification of the appropriate level for tests and full reporting of outcomes
☐	☒	Estimates of effect sizes (e.g. Cohen's d, Pearson's r), indicating how they were calculated
☐	☒	Clearly defined error bars<br>*State explicitly what error bars represent (e.g. SD, SE, CI)*

Our web collection on statistics for biologists may be useful.

Software and code

Policy information about availability of computer code

Data collection	BLAST (http://blast.ncbi.nlm.nih.gov/)
Data analysis	Bowtie (Shimada et al., 1991) ;SOAPdenovo (Li et al., 1995);ClustalW 1.83 program (Thompson et al., 1994) ;DOGMA (Dual Organellar GenoMe Annotator (Wyman et al., 2004);OGDRAW v1.1 (Lohse et al., 2007); MAFFT v5 (Katoh et al., 2005);Circos-0.64 (Krzywinski et al., 2009);PAUP*4.0b10 (Swofford et al., 2003); RAxML version 7.2.6 (Stamatakis, 2006);MrBayes version 3.1.2 (Ronquist et al., 2003);ClustalW1.83 program (Thompson et al., 1994);PRANK+F (Löytynoja et al., 2008);SSRHunter software (http:// www.biosoft.net) ;DNAMAN Version 6.0.3.99 (Lynnon Biosoft, Vaudreuil, Que¬bec, Canada)

For manuscripts utilizing custom algorithms or software that are central to the research but not yet described in published literature, software must be made available to editors/reviewers upon request. We strongly encourage code deposition in a community repository (e.g. GitHub). See the Nature Research guidelines for submitting code & software for further information.

Data

March 2018

Policy information about availability of data

All manuscripts must include a data availability statement. This statement should provide the following information, where applicable:

- Accession codes, unique identifiers, or web links for publicly available datasets
- A list of figures that have associated raw data
- A description of any restrictions on data availability

All genome sequences and their annotation have been submitted to NCBI and BIG databases. The data that support the findings of this study are available from the corresponding author upon reasonable request.

Field-specific reporting

Please select the best fit for your research. If you are not sure, read the appropriate sections before making your selection.

☒ Life sciences ☐ Behavioural & social sciences

For a reference copy of the document with all sections, see nature.com/authors/policies/ReportingSummary-flat.pdf

Life sciences

Study design

All studies must disclose on these points even when the disclosure is negative.

Sample size	We selected more than 22 cp genome for comparisons which are sufficient for our analysis.
Data exclusions	No data were excluded from the analysis.
Replication	All attempts at replication were successful.
Randomization	This is not relevant to our study, because it is not needed for us to perform comparative analysis of cp genomes.
Blinding	This is not relevant to our study.

Materials & experimental systems

Policy information about availability of materials

n/a	Involved in the study
☒	☐ Unique materials
☒	☐ Antibodies
☒	☐ Eukaryotic cell lines
☒	☐ Research animals
☒	☐ Human research participants

Method-specific reporting

n/a	Involved in the study
☒	☐ ChIP-seq
☒	☐ Flow cytometry
☒	☐ Magnetic resonance imaging
